# Preparation of corrosion inhibitor from natural plant for mild stil immersed in an acidic environmental: experimental and theoretical study

**DOI:** 10.1038/s41598-024-58637-z

**Published:** 2024-04-04

**Authors:** Maryam Pourmohseni, Alimorad Rashidi, Mehrnoosh Karimkhani

**Affiliations:** 1 Department of Chemistry Central Tehran Branch, Islamic Azad University, Tehran, Iran; 2 Nanotechnology Research Center, Research Institute of Petroleum Industry (RIPI), Tehran, Iran

**Keywords:** Marjoram plant, Corrosion, Density functional theory, Green corrosion inhibitor, EIS, Plant sciences, Environmental sciences, Chemistry, Mathematics and computing

## Abstract

In the present study, the inhibition performance of some medicinal plants (i.e. Yarrow, Wormwood, Maurorum, Marjoram, and *Ribes rubrum*) was theoretically and experimentally investigated for mild steel immersed in 1M HCl. In this way, the obtained extracts characterized by Fourier transform infrared spectroscopy (FT-IR) and the electrochemical and theoretical techniques were used to study the inhibition mechanisms of the extracts for the immersed electrode in the acidic solution. In addition, the microstructure of the electrode surface immersed in the blank and inhibitor-containing solutions characterized by field emission scanning electron microscopy (FE-SEM), and Violet-visible (UV–Vis) spectroscopy was used to confirm the adsorption of the compounds on the electrode surface. The obtained electrochemical results revealed that the inhibition performance of the green inhibitors increased by increasing their dosage in the electrolyte. In addition, it was proved that Marjoram plant extract possessed the most inhibition efficiency (up to 92%) among the under-studied herbal extracts. Marjoram extract behaved as a mixed-type inhibitor in the hydrochloric acid solution, and the adsorption process of the extract on the steel surface followed the Langmuir adsorption model. Adsorption of the compounds on the steel surface was also studied using density functional theory (DFT), and it was found that the protonated organic compounds in the extract have a high affinity for adsorption on the electrode surface in the acidic solution.

## Introduction

It is asserted that natural disasters, including tornadoes, lightning, floods, fires, and earthquakes, have lower yearly costs than corrosion^1^. Therefore, safeguarding alloys from the phenomena is an alluring topic in both the industrial and academic spheres^2–4^. Acid solutions are frequently used in industrial cleaning, acid pickling, and oil-well acidizing^5,6^. Corrosion inhibitor compounds are one of the most important ways to avoid alloys submerged in corrosive solutions from degrading^7,8^.

It is well known that inhibitors’ electronic structure and spatial orientation have been linked to their efficacy^9^. Generally, inhibitors containing π-bonds and heteroatoms (P, S, N, and O) with lone pair electrons are the most potent and efficient^10^. Because both inorganic and organic inhibitors are usually hazardous, researchers are looking for more environmentally friendly substances, often known as green or natural inhibitors (NIs)^11,12^. NIs, which comprise medication and plant extracts, are regarded as safe substances devoid of heavy metals^13^. The use of medicinal plants as NIs for submerged alloys in aggressive media has been experimentally described previously, in addition to their conventional use as medicines^14,15^. Antimalarial, analgesic, antibacterial, antiviral, antifungal, and antineoplastic medications have all been reported to be employed as NIs for submerged alloys under different conditions^16,17^.

For hundreds of years, people have used *Urtica dioica* L. extract as an effective ancient medicine to treat anemia, eczema, arthritis, gout, and painful muscles and joints. Today, it is utilized to address urinary issues when prostate enlargement is still in its early stages^18^. Nasibi et al.^19^ investigated the corrosion inhibition performance of the extract for mild steel (MS) immersed in 1M HCl. The obtained electrochemical analysis results revealed that the maximum inhibition efficiency of the extract was 92.24% in the presence of 300 ppm of the NI at 40 °C. *Malva sylvestris* L. is another ancient medicine with antimicrobial, hepatoprotective, anti-inflammatory, and antioxidant properties^20^. Naghi Tehrani et al.^15^ used the herbal medicine as NI for MS and found that the inhibition efficiency of the extract was about 91% at the concentration of 2000 ppm in a saline media. *Mentha suaveolens* L. extract has many health advantages, including lowering fevers and easing asthma and depression. Mint is frequently used as a tea as a home remedy to help relieve stomach discomfort. In addition, the extract was originally used as a medicinal herb to treat stomach and chest aches^21^. Salhi et al.^14^ used the plant’s essential oil and aqueous extract as a potent NI for MS in 0.5M H_2_SO_4_. The obtained EIS results showed that 2000 ppm of the essential oil and the aqueous extract provided inhibition efficiencies of 81.9% and 91.3% in the corrosive media, respectively. *Foeniculum vulgare* M., which is usually known as fennel, has been used for respiratory, reproductive, endocrine, and digestive systems. It is additionally utilized by breastfeeding women as a galactagogue agent^22^. Lahhit et al.^23^ and Bouoidina et al.^24^ used essential oil of the herbal medicine as an NI for carbon steel in an HCl electrolyte. Both studies proved that the essential oil's inhibition performance increased in the presence of more inhibitor dosages but decreased with the increase in temperature.

Yarrow is a helpful plant to keep on hand for various illnesses since it possesses antiseptic, astringent, antibacterial, and anti-inflammatory qualities. To aid in stopping bleeding, Yarrow leaves can be applied topically or powdered into a styptic powder^25^. Wormwood has historically been considered a helpful treatment for issues with the liver and gallbladder. Absinthin and Anabsinthin, two potent bitter substances in Wormwood, stimulate the gallbladder and digestive systems. According to popular belief, Wormwood helps to ease intestinal spasms and stimulate digestion^26^. In folk medicine, Maurorum treats piles, migraine, warts, and rheumatism as purgative, diaphoretic, expectorant, and diuretic^27^. Marjoram has a long history of usage in both traditional medicine and cookery. It may reduce inflammation, ease gastric discomfort, and regulate the menstrual cycle, among other potential advantages^28^. Lycopene, an anti-oxidant carotenoid, is found in Ribes Rubrum. Heart disease and cancer risk are both decreased by lycopene, particularly prostate cancer. Furthermore, it shields the body from the effects of free radical stress, which can harm DNA and other cellular structures^29^.

In the present study, extracts of five different herbal medicines (i.e., Yarrow, Wormwood, Maurorum, Marjoram, and *Ribes rubrum*) were used as NIs for MS exposed to 1M HCl solution, and their inhibition performance was investigated using electrochemical techniques. The results of this research can open a window to using of traditional herbal medicines as new and powerful NIs to prevent corrosion damage.

## Materials and methods

### Materials

**Figure 1:**
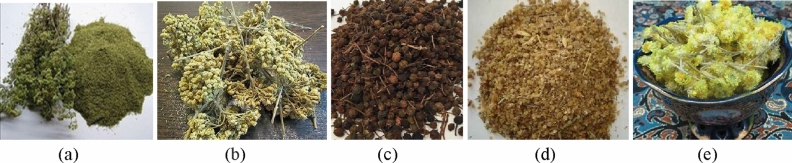
Picture of the plants (**a**) Marjoram, (**b**) Yarrow, (**c**) *Ribes rubrum*, (**d**) Maurorum, (**e**) Wormwood.

HCl solution (1M) was prepared using hydrochloric acid (37% Merck) and distilled water. Steel panels (St-12) in the dimension of 0.5 × 5 × 15 cm were obtained from Foolad Mobarakeh Company (Iran) with the following chemical composition: C (0.190 wt%), Si (0.288 wt%), Mn (1.388 wt%), Cr (0.03 wt%), Mo (0.016 wt%), Co (0.386 wt%), Cu (0.299 wt%), Nb (0.354%), and Fe (balance). Ribes rubrum (collected from Arsbaran forests, Iran), Wormwood (collected from Mazandaran, Iran), Marjoram (collected from Astara, Iran), Yarrow (collected from Esfahan, Iran), Maurorum (collected from Tabriz, Iran). Picture of the plants are shown in Fig. 1.

### Preparation of the extracts

At first, the medicinal plants, including Yarrow, Wormwood, Maurorum, Marjoram, and *Ribes rubrum* were powdered, and then 50 g of powders was mixed with 500 ml of distilled water, followed by heating and stirring on a magnetic stirrer at 70 °C for 5 h. afterward, the mixtures were centrifuged at 5000 rpm for 5 min to remove undissolved particles. Then, the solvent (water) was evaporated by incubating the filtered solution in an oven at 500 °C for 48 h and the obtained dry powders were used as the NIs for the electrochemical analyses.

### Steel surface preparation

To prepare the surface of mild steel (MS) panels for the electrochemical tests, the sheets were degreased with acetone to remove surface contamination and then polished with 400, 600, 800, and 1200 sandpapers to remove the oxidized layers. Then, they were rewashed with acetone and dried at 50 °C.

### Characterization methods

To investigate the chemical compounds in the used extracts, FT-IR analysis was performed in the range of 400–4000 cm^−1^ using a Perkin-Elmer spectrometer. UV–VIS spectra were carried out using a UV–Vis spectrophotometer (Hitachi U-3010) in the solution medium. Using an emission scanning electron microscope (FE-SEM) model Mitra3, the surface of the immersed electrodes was studied in the presence and absence of the NIs.

### Electrochemical tests

The corrosion inhibition of the extracts for MS immersed in the 1M HCl was evaluated in the presence of different dosages of the NIs (0, 200, 400, 600, and 800 ppm) at different immersion times (0.25, 2, 4 6, and 24 h).

Electrochemical analyses (EIS and PP) were done using a 3-electrode system including a saturated calomel reference electrode, platinum as an auxiliary electrode, and the prepared steel panels with a diameter of 1 cm^2^ as the working electrodes by an Ivium Compactstat instrument. Before performing the electrochemical analyses, the steel electrodes were submerged in the corrosive solutions for 15 min to reach steady state conditions. EIS measurements were performed in the frequency range of 10 kHz to 10 mHz with a sinusoidal voltage of ± 10 mV. PP tests were also carried out in the potential range of + 250 mV to – 250 mV with a 0.001 V s^−1^ scan rate.

### Theoretical studies

The ORCA program package, module version 4.0, was used to carry out the quantum-based calculations. Prior to completing geometry optimization, the calculations began with no geometry constraints. The global minimum for geometry optimizations has been found after careful consideration. The hybrid B3LYP functional level with a higher basis set, indicated as 6-311G(d,p), was used for all calculations with complete geometry optimization^30^. Self-Consistent Reaction Field (SCRF) theory and the Polarized Continuum Model (PCM) have been used to analyze the effect of solvent^31^. Koopman’s theory states that the energies of the HOMO and LUMO orbitals were used to calculate the ionization energy and the electronic affinity^32,33^.

### Ethical statement

The plant collection and use was in accordance with all the relevant guidelines.

## Results and discussion

### FT-IR investigations

**Figure 2:**
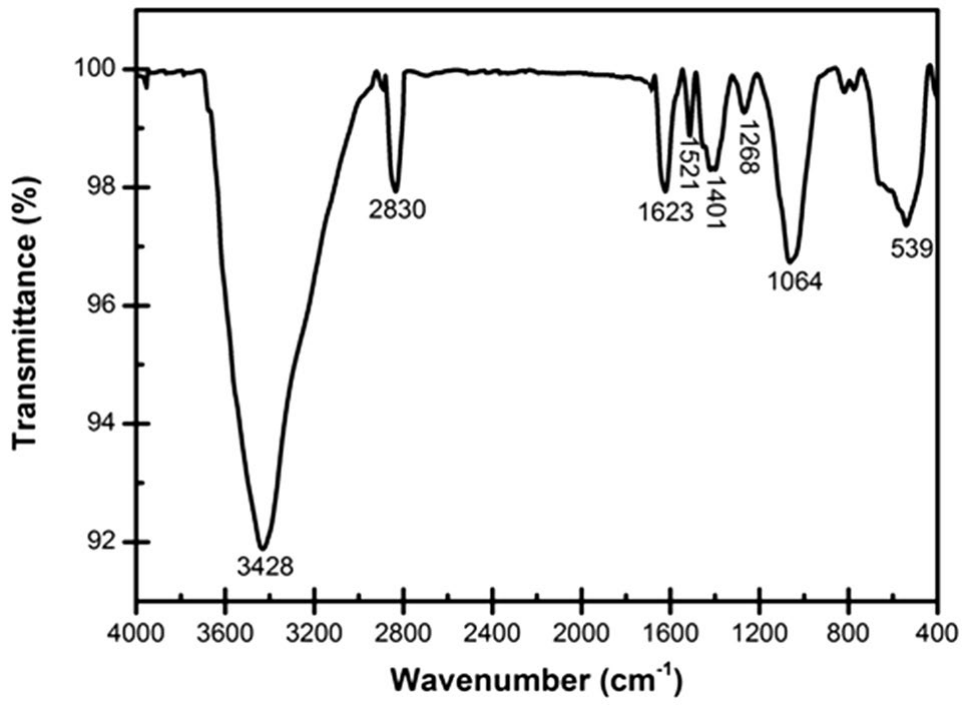
FT-IR spectrum of the Marjoram extract used in this research.

**Figure 3:**
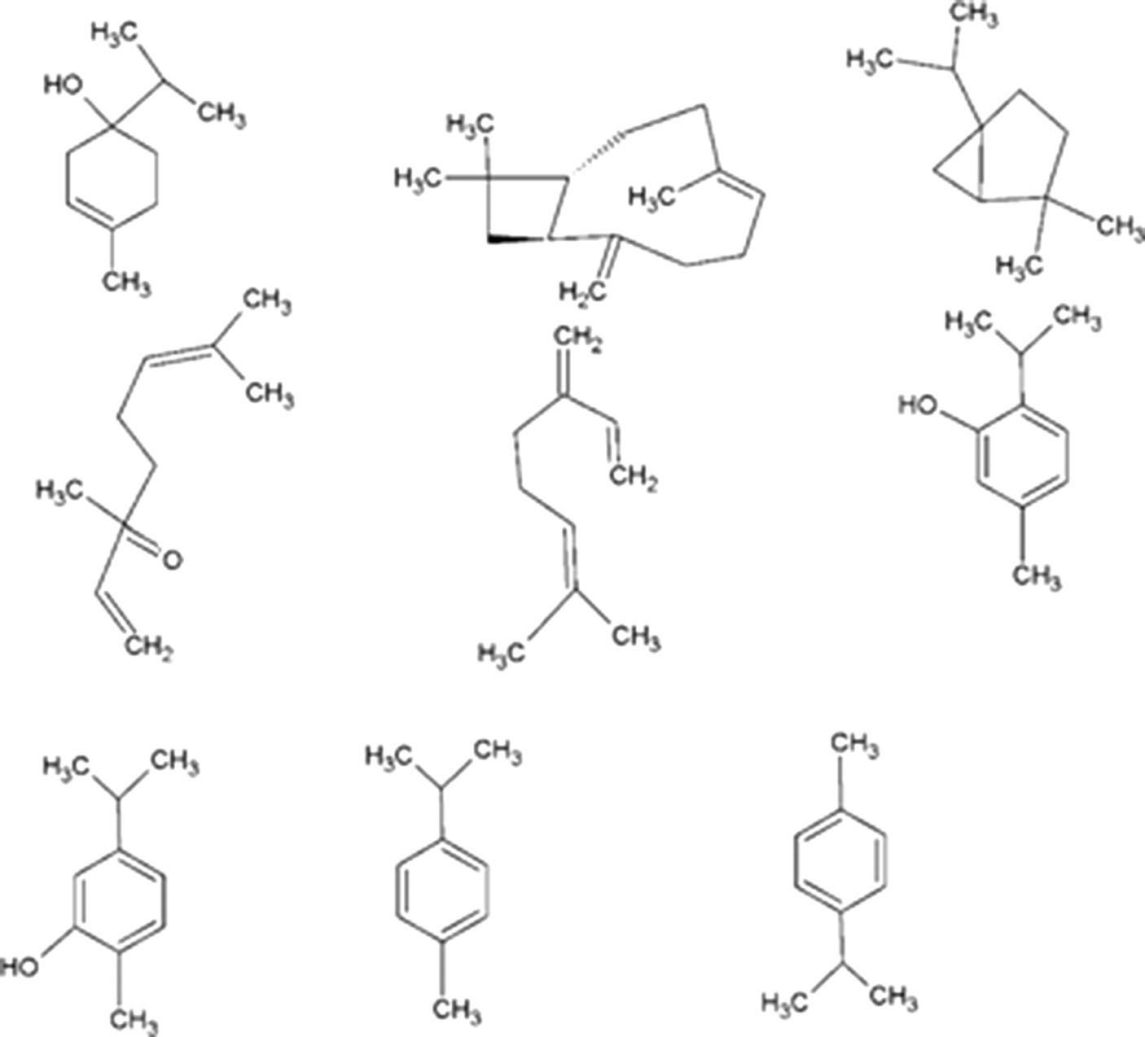
The chemical compounds found in the extract of Marjoram medicinal plant described by Lopez et al.^34^.

FT-IR spectroscopy was used to investigate the chemical compounds in the under-studied extracts. Other plant extracts are presented in the supplementary information except for the FT-IR result of the Marjoram extract. The FT-IR spectrum of herbal extract from Marjoram and the present chemical compounds in the extract (described by Lopez et al.^34^) are shown in Figs. 2 and 3, respectively.

As demonstrated in Fig. 2, the marjoram extract contains chemical compounds with C–H, C–OH, O–H, and aromatic rings. The broad band located at 3428 cm^−1^ and the sharp peak centered at 1623 cm^−1^ belong to the stretching and bending vibrations of O–H bonds in the hydroxyl groups and surface adsorbed water, respectively^35^. The stretching vibrations peak of C–H bonds in aliphatic groups is located at 2830 cm^−1^^36^. Absorption peaks at 1521 cm^−1^ and 1401 cm^−1^ are related to the stretching vibration of C=C bonds in aromatic rings and the bending vibration of C–H bonds, respectively^37^. The peak of stretching vibration of C–O–C and C–OH bonds appeared at 1064 cm^−1^^38^. The peak centered at 539 cm^−1^ is related to the bending vibration of the C–H bonds connected to the aromatic rings and the bending vibration of the rings^37^.

### EIS measurements

Nyquist and Bode curves of MS electrodes immersed in the 1M HCl electrolyte with different dosages of the NIs for various exposing times are presented in Figs. S6 and S7, respectively, in supplementary information. The charge transfer resistance of the samples can be directly related to the diameter of the Nyquist semicircles. The Nyquist curves clearly show the increased diameter of the semicircles in the presence of the herbal extracts compared to the inhibitor-free samples, indicating the effect of the medicinal plant extracts on the anodic and cathodic reactions of MS corrosion and showing their inhibition effect. The increased diameter of the semicircles is also observed with increasing time, suggesting that the adsorption of the NI’s compounds on the immersed MS and the formation of the protective layer on the electrode surface is a time consuming process. In addition, it can be seen that the largest diameter of the semicircles in all concentrations and all immersion times belongs to the sample containing Marjoram extract, indicating that the herbal extract has a more significant effect on the corrosion reactions of the immersed MS in the acidic environment than other extracts.

The impedance at the lowest frequency (|Z_10mHz_|) can be considered an index for a system's total corrosion resistance^39,40^. A comparison of the |Z_10mHz_| values observable from the Bode curves (Fig. S7) revealed that the index values for the NI-containing samples were more than those obtained for the blank samples. Furthermore, the total corrosion resistance values increased with the immersion times and the concentration of the NIs. It is also observed that the immersed MS in the solution containing the Marjoram extract possessed more |Z_10mHz_|values than the other samples.

Due to the fact that only one peak can be seen in the phase angle curves, it can be concluded that the under-studied samples can be electrochemically modeled with a simple Randels equivalent electrical circuit. In this circuit, Rs is solution resistance, R_ct_ is charge transfer resistance, and Q_dl_ is a constant phase element (CPE) of the double layer, which is used instead of an ideal capacitor to better modeling of the EIS data. According to Figs. S6 and S7, the modeled data fitted well with the experimental results, suggesting the accuracy of the obtained electrochemical data reported in Table S1 (supplementary information). In this table, Y_0_ and n are the admittance and power values of Q_dl,_ respectively. The “n” value can represent the degree of heterogeneity of the metal surface (0 ≤ n ≤ 1). According to literature^41,42^, the higher n values in the inhibitor**-**containing solutions proves the creation of a protecting film on the steel surface.

In Table S1, the C_dl_ value, which corresponds to the capacity of the double-layer capacitor, is estimated with Eq. (1)^43^.

1 $$ {\text{C}}_{{{\text{dl}}}} = \, \left( {{\text{Y}}_{{0,{\text{dl}}}} } \right)^{{({1}/{\text{n}})}} \times \, \left( {\frac{{{\text{Rct }} \times {\text{Rs}}}}{{{\text{Rct}} + {\text{Rs}}}}} \right)^{{\left( {\left( {{1} - {\text{n}}} \right)/{\text{n}}} \right)}} $$

As the concentration of the extract increases, the C_dl_ value decreases. The decline can be attributed to a decrease in the dielectric constant due to substituting the extract’s chemical compounds with water molecules on the MS surface. Indeed, since the value of the water dielectric coefficient is high than organic molecules, the adsorption of the organic molecules on the MS surface and desorption of water molecules results in the increment of the capacitance values. In addition, the capacitance of a capacitor has an inverse relationship with the distance between the plates^44^. Thus, replacing of the large inhibitor molecules instead of water molecules on the metal surface increases the thickness of the electric double layer, causing a decrease in the C_dl_ value.

According to the results reported in Table 1, the C_dl_ values were significantly reduced by increasing the dosage of the NIs and exposing time. In other words, due to the adsorption of the natural compounds on the immersed surface, the formed layer leads to a decrease in the C_dl_ values. Among the herbal extracts, it can be again seen that the lowest values of the C_dl_ belong to the Marjoram sample, which also confirms the formation of a more protective layer on the steel surface in the presence of this inhibitor.

**Table 1:** Calculated quantum chemical parameters for compounds (eV), μ in Debye, σ and ε in (eV^−1^).

Compound	E_HOMO_	E_LUMO_	ΔE	EA	IP	χ	μ	η	σ	ΔN
Thymol	− 8.673	− 4.377	4.296	4.377	8.673	6.525	1.487	2.148	0.466	0.111
Carvacrol	− 8.688	− 4.363	4.325	4.363	8.688	6.526	1.578	2.163	0.462	0.110
β_Myrcene	− 9.350	− 5.096	4.254	5.096	9.350	7.223	0.527	2.127	0.470	− 0.052
Linalool	− 9.415	− 4.274	5.141	4.274	9.415	6.845	1.547	2.571	0.389	0.030
Caryophyllene	− 9.417	− 4.166	5.251	4.166	9.417	6.791	0.726	2.625	0.381	0.040
Cymene	− 9.450	− 4.482	4.968	4.482	9.450	6.966	0.051	2.484	0.403	0.007
Terpinene	− 8.760	− 5.149	3.611	5.149	8.760	6.954	0.731	1.805	0.554	0.013
Sabinene	− 7.409	− 1.079	6.330	1.079	7.409	4.244	0.157	3.165	0.316	0.435

**Figure 4:**
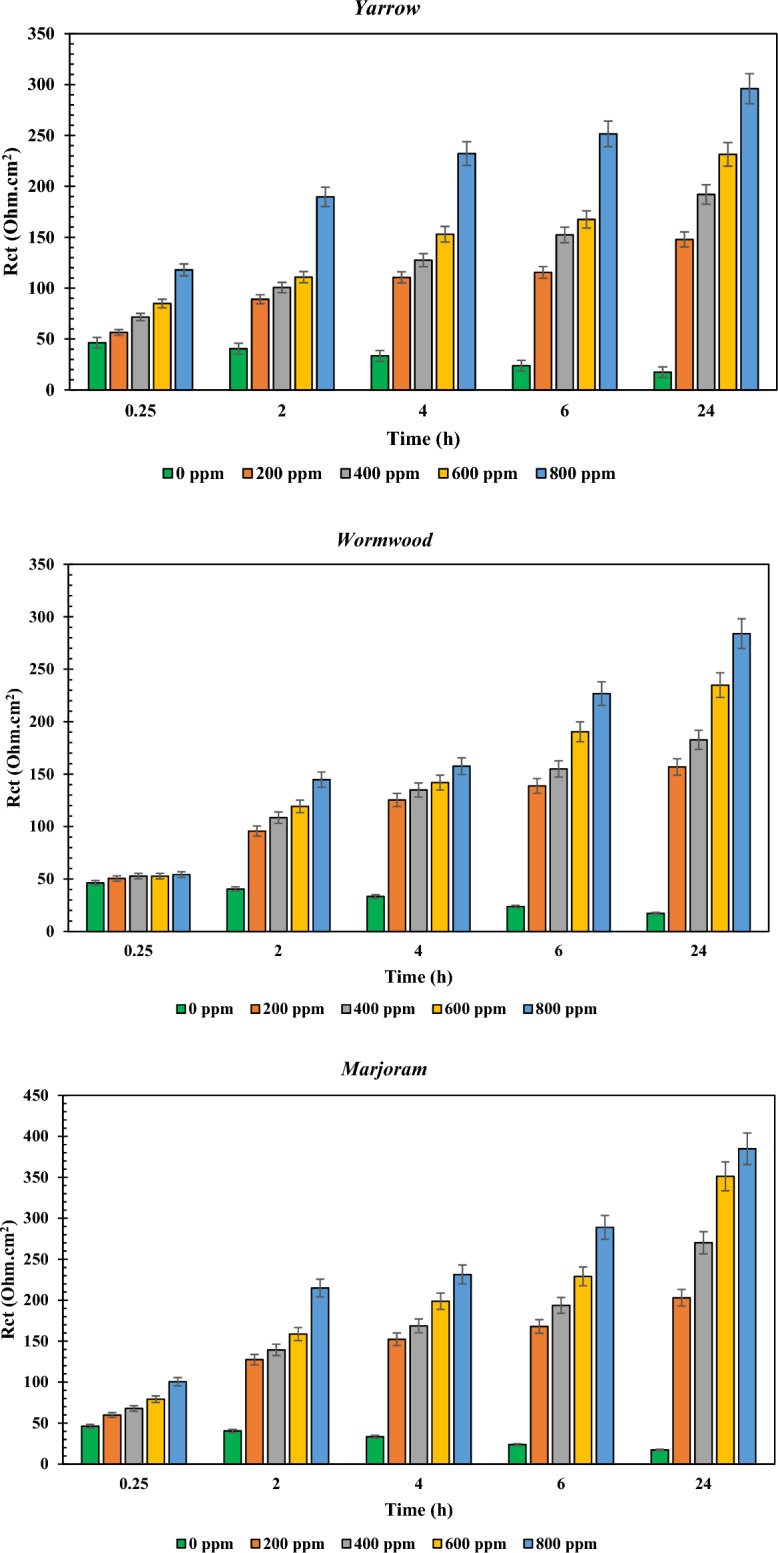

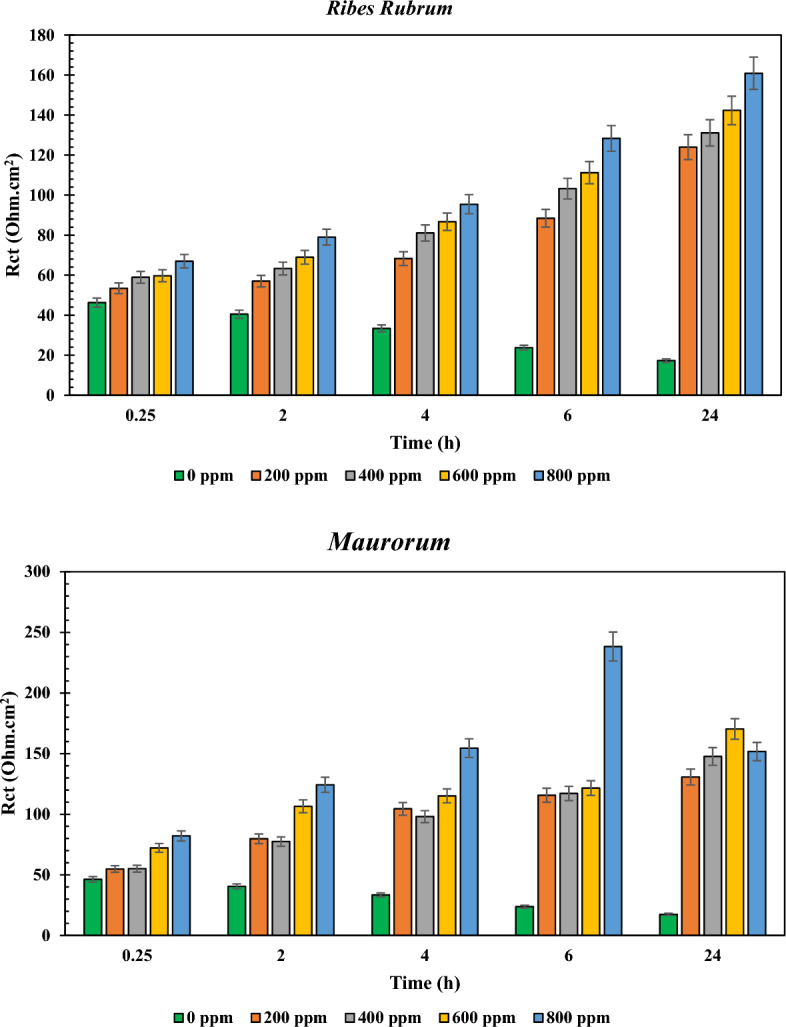
Column diagrams of the reported R_ct_ values in Table S1 for the under-studied samples in various dosages of the NIs and different immersion times.

Since the obtained R_ct_ values in Table S1 can be considered as the electrochemical resistance against corrosion reactions^45^, the column diagrams of the parameter are shown in Fig. 4 for a better comparison of the results.

From the graphs shown in Fig. 4, it can be seen that the charge transfer values increased by increasing the dosage of the NIs, indicating that the adsorption of the chemical compounds on the MS surface is the main mechanism for the inhibition behavior of the used NIs. In addition, unlike the blank samples, the R_ct_ values for the NI-containing samples increased by increasing the immersion time, as seen in Nyquist and Bode diagrams. Furthermore, the higher charge transfer resistance of the sample containing Marjoram extract can be clearly observed, followed by the sample containing Yarrow extract, which confirms the results of the previous results. Moreover, the lowest corrosion resistance values among the NI-containing samples belonged to those containing the extracts of *R. Rubrum* and Maurorum.

The value of inhibition efficiency (%IE) in Table S1 is obtained from Eq. (2).

2 $$ \% {\text{IE}}_{{}} = { 1}00 \, \times \, \left( {{\text{R}}_{{{\text{ct}}}}^{{{\text{in}}}} {-}{\text{ R}}_{{{\text{ct}}}}^{0} } \right)/{\text{R}}_{{{\text{ct}}}}^{0} $$

where, R_ct_^in^ and R_ct_^0^ are the charge transfer resistance in the presence and absence of the NIs, respectively. The obtained results confirm the higher inhibition performance of the Marjoram-containing sample, followed by the samples containing Yarrow, Wormwood, *Ribes rubrum*, and Maurorum herbal extracts.

### PP measurements

PP curves of the samples containing different amounts of the herbal extracts are represented in Fig. S11. Electrochemical parameters obtained from Tafel extrapolation of the PP curves are reported in Table S2 (in supplementary information). The PP curves show that by increasing the concentration of the NIs the corrosion current density (corrosion rate) decreases, confirming the obtained data from EIS analysis. In fact, in the presence of more dosages of the NIs, and the formation of a thicker adsorbed layer on the immersed electrodes, the electron transfer process from the anode regions to the cathode regions disrupts, which can inhibit the corrosion reactions^46^.

In Table S2, the slopes of the anodic and cathodic branches are represented by βa and βc. E_corr_ is the corrosion potential and i_corr_ is the corrosion current density of the electrodes. R_p_ is the polarization resistance and %IE is the inhibition efficiency, which are respectively calculated using Eqs. (3) and (4)^47^.

3 $$ {\text{R}}_{{\text{p}}} = \, (\beta_{{\text{a}}} \times \beta_{{\text{c}}} )/({2}.{3}0{31 } \times {\text{ i}}_{{{\text{corr}}}} \times \left( {\beta_{{\text{a}}} + \beta_{{\text{c}}} )} \right) $$

4 $$ \% {\text{IE }} = {1}00 \, \times \, \theta $$

**Figure 5:**
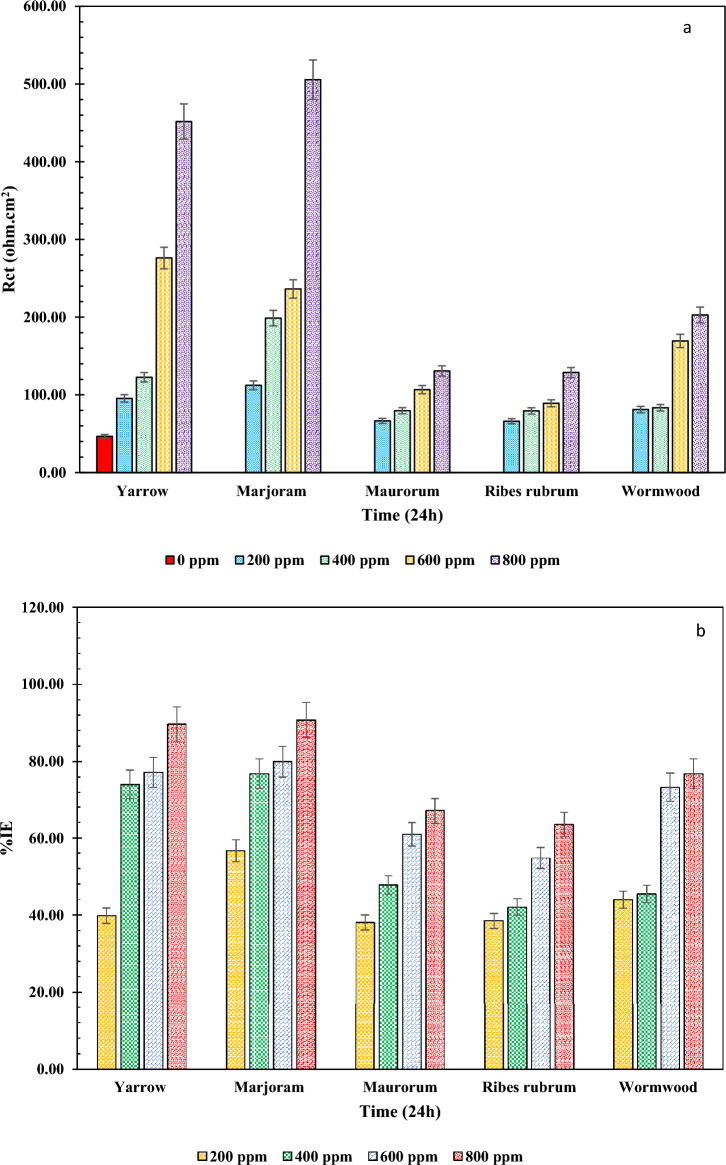
Column graphs of (**a**) R_p_ and (**b**) %IE values for the under-studied samples at different dosages obtained from the PP curves.

In Eq. (4), θ is the coverage degree of the NIs obtained from Eq. (5).

5 $$ \theta \, = \, \left( {{\text{i}}^{0}_{{{\text{corr}}}} {-}{\text{ i}}_{{{\text{corr}}}} } \right) \, /{\text{ i}}^{0}_{{{\text{corr}}}} $$

where i^0^_corr_ and i_corr_ are the corrosion current densities of the samples without and with the Nis, respectively. Column graphs of the obtained polarization resistance and inhibition efficiency values from the Tafel extrapolation of the PP curves are shown in Fig. 5.

It can be seen from Fig. 5 that the samples with the extracts of Marjoram and Yarrow have the highest polarization resistance and inhibition efficiency values. Accordingly, the highest inhibition efficiency (about 90%) and the lowest corrosion rate among the studied samples were obtained for the sample with 800 ppm of Marjoram. These results confirm those obtained in the EIS test and indicate that the inhibition performance of Marjoram extract is optimal compared to the other samples. Indeed, the formation of a more protective layer in the presence of the NI on the surface of the MS electrode led to providing a higher polarization resistance value than other herbal extracts.

At low concentrations, the surface coverage of the NIs is inadequate. So, with the increase of the NI dosages and increase of the surface coverage, the inhibition efficiency of the NIs arises (Fig. 5b). Despite the higher i_corr_ value in the blank solution than the NI-containing samples, the Tafel slopes remained almost unchanged, indicating that the extracts had no significant effect on the reaction kinetics of the hydrogen gas evolution and the mechanism of iron dissolution. Therefore, the main mechanism of corrosion inhibition is simple adsorption of the compounds in the extracts on the electrode surface.

Due to the negligible alterations in the values of β_a_ and β_c_ in the presence of different dosages of the NIs, as well as the slight shift of the corrosion potential in these samples (less than ± 85 mV compared to the blank sample), it can be concluded that the NIs acted as mixed-type inhibitors^16,48^.

By adding the compounds shown in Fig. 5 to the corrosive electrolyte, the protonation of these compounds in the acidic electrolyte causes them to be absorbed on the steel surface because the steel surface has gained a negative charge due to the absorption of chloride anion on it. On the other hand, the formation of a complex between the iron cation produced by the anodic reaction with the organic compounds in the extract causes the formation of insoluble compounds, which create a protective layer by depositing on the surface of the immersed MS. In addition, creating a covalent bond between lone pair electrons of heteroatoms and multiple bonds in the compounds of the extract with the empty orbital of iron atoms on the surface of the electrode leads to the formation of a chemical bond between the electrode and the compounds. So, the inhibition mechanism of the extracts can be considered physical, chemical, or physical–chemical. In this regard, investigating adsorption isotherm models can help identify the inhibition mechanism.

### Adsorption isotherms

The mechanism of surface adsorption and the interaction between molecules of a NI and the metal surface can be characterized using models called adsorption isotherms^49,50^. Langmuir (Eq. 6) and Freundlich (Eq. 7) adsorption isotherms are the main models widely employed to investigate the adsorption of an inhibitor on an electrode surface^51^.

6 $$ {\text{C}}/\theta \, = { 1}/{\text{K}}_{{{\text{ads}}}} + {\text{ C}} $$

7 $$ {\text{ln }}\left( \theta \right) \, = {\text{ ln K}}_{{\text{F}}} + { 1}/{\text{n }} \times {\text{ ln C}} $$

where C is the concentration of the NI, and θ is the coverage degree. Langmuir and Freundlich absorption constants are respectively represented by K_ads_ ads and K_F_. The affinity of a NI adsorption can be achieved by the coefficient 1/n in the Freundlich isotherm equation^51^. According to Eq. (6), if C/θ is plotted versus C, and the linear regression (R^2^) is close to one, it can be concluded that the NI followed the Langmuir isotherm model. Likewise, a linear regression near to one for plotting ln (θ) versus ln C shows that the inhibitor followed the Freundlich model.

**Figure 6:**
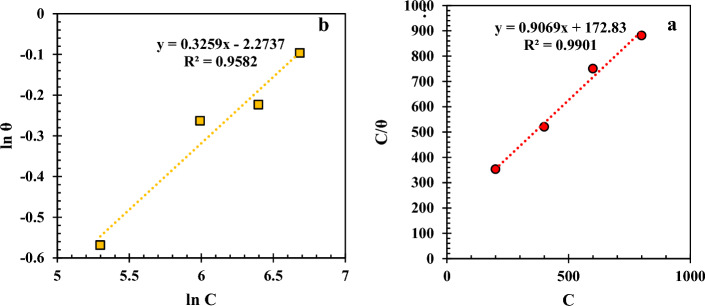
(**a**) Langmuir and (**b**) Freundlich adsorption isotherm plots for the MS immersed in the electrolyte with 800 ppm Marjoram extract.

According to Fig. 6, the immersed MS in the electrolyte solution with 800 ppm Marjoram extract (the optimum sample based on the electrochemical analyses) follows the Langmuir adsorption model with R^2^ > 0.99, indicating that the adsorption is in a monolayer form and all active adsorption sites have the same tendency to adsorb the inhibitor compounds^49,50^.

The values of K_ads_ and ΔG_ads_ (obtained from Eq. 8) are equal to 5786.03 L mol^−1^ and − 31.309 kJ mol^−1^, respectively.

8 $$ \Delta {\text{G}}_{{{\text{ads}}}} = \, - {\text{RT ln }}\left( {{55}.{\text{5 K}}_{{{\text{ads}}}} } \right) $$

In Eq. (8), R is the universal gas constant (3.314 kJ mol^−1^) and T is the absolute temperature (K).

The negative value of ΔG_ads_ indicates the spontaneity of the inhibitor adsorption on the MS surface in the corrosive media. Additionally, the high value of K_ads_ suggests that the NI has a high thermodynamical affinity to adsorb on the surface of the immersed metal^52,53^. Generally, the adsorption mechanism can be determined by the absolute value of ΔG_ads._ When ΔG_ads_ < 20 kJ mol^−1^, the adsorption mechanism is physisorption, and when ΔG_ads_ > 40 kJ mol^−1^, chemisorption can be considered the main adsorption mechanism. 20 kJ mol^−1^ < ΔG_ads_ < 40 kJ mol^−1^ indicates that the physicochemical adsorption is the main interaction between the NI and the immersed metal^54,55^. So, for the under-studied sample, it can be concluded that the adsorption of the NI on the MS surface is in the form of the physico-chemical model.

### Microscopic studies

**Figure 7:**
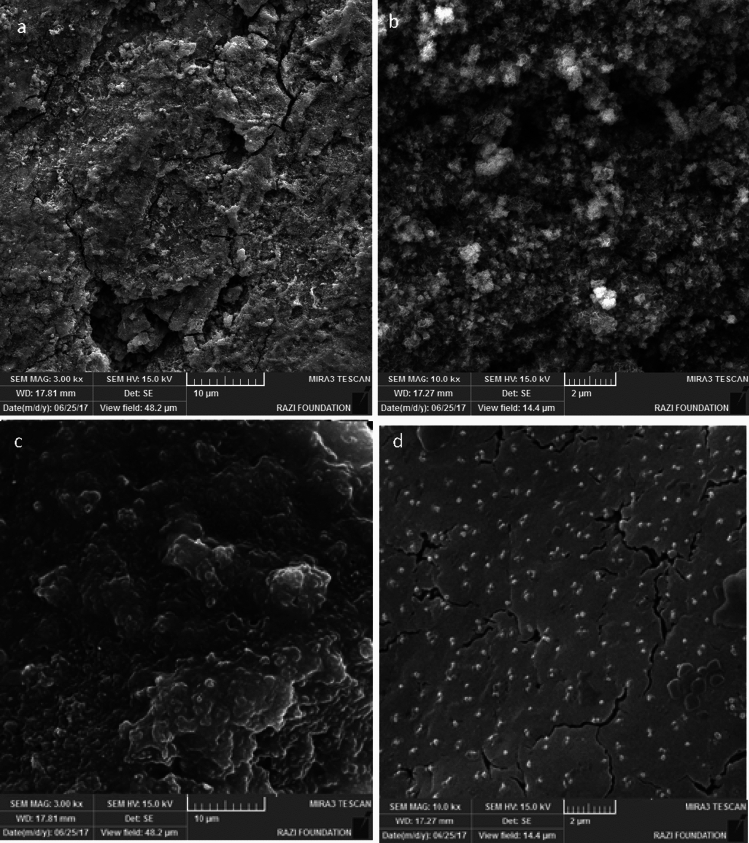
FE-SEM micrographs of MS surface immersed for 24 h in the corrosive electrolyte (**a**, **b**) without and (**c**, **d**) with 800 ppm Marjoram extract.

Figure 7 displays the morphology and microstructure of the MS surface exposed to the acid solution with and without the optimum NI (800 ppm Marjoram extract). The highly rough surface of the soaked MS in the blank solution indicates the surface degradation of the electrode due to the dissolution of iron atoms as Fe^2+^ and Fe^3+^ cations into the electrolyte. From the high magnification micrograph (Fig. 7b), it can be clearly seen that the surface was strongly damaged by the direct attack of the acid solution.

**Figure 8:**
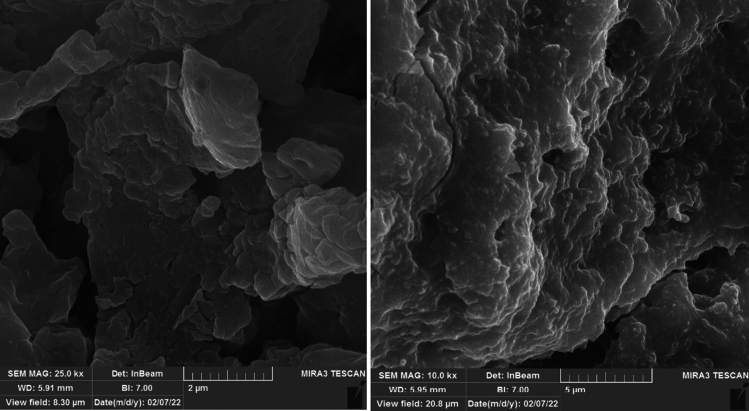
FE-SEM micrographs of MS surface immersed for 24 h in the corrosive electrolyte with 800 ppm Yarrow extract.

In the presence of the NI, a compact adsorbed layer can be seen on the surface of the exposed MS. The protection layer led to the reduction of Fe dissolution in the acidic environment and created a smoother surface. In fact, the formation of covalent bonds between the iron atoms and the extract’s compounds reduced the iron dissolution in the HCl electrolyte. Therefore, it can be concluded that the presence of the NI affects the corrosion reactions of the MS and reduces the rate of anodic and cathodic reactions, confirming the electrochemical analyses. SEM image of yarrow plant extract as an organic inhibitor in the sample containing 800 ppm inhibitor is provided for a comparison in Fig. 8.

### UV–VIS spectroscopy

**Figure 9:**
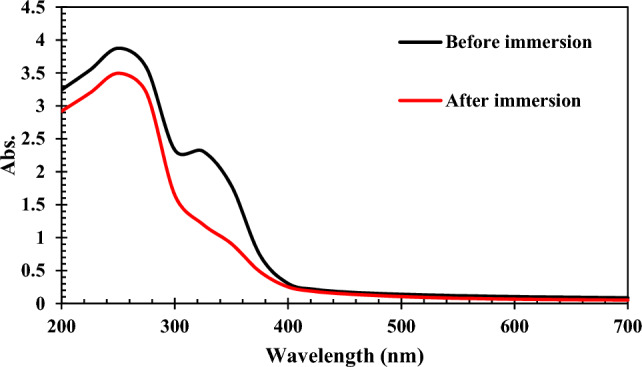
UV–Vis spectra of the HCl solution with 800 ppm Marjoram extract before and after immersion of the MS electrode.

The interaction between the immersed MS surface in the NI-containing medium and the inhibitor molecules in an acidic medium can be studied using UV–Vis spectroscopy. To confirm the formation of an adsorbed inhibitor-based protect layer on the immersed metal surface, UV–Vis analysis was performed before and after exposing the MS electrodes to the 1M hydrochloric acid solution with 800 ppm Marjoram extract (Fig. 9). Generally, the appeared peaks at 250 and 325 nm in the spectrum of the MS-free electrolyte can be related to π–π* and n–π* electron transition of C=C and C=O bonds, respectively^56^. It is clear from Fig. 9 that the intensity of the peaks decreased significantly after 24 h immersion of the MS electrode in the solution, indicating the adsorption of the extract’s compounds on the surface of the electrode, which decreased the concentration of organic compounds in the solution. These observations indicate the formation of a bond between inhibitor molecules and the surface of the steel sample, which reduces the opening of organic compounds in the electrolyte to absorb on the surface of the electrode. The lone pair electrons of heteroatoms, as well as ring π electrons, have been successfully shared with d orbitals of Fe atoms located on the steel surface. These reactions create a single nanometer layer on the surface, which can absorb and reduce the speed reactions of the steel in the active sites of the metal.

**Figure 10:**
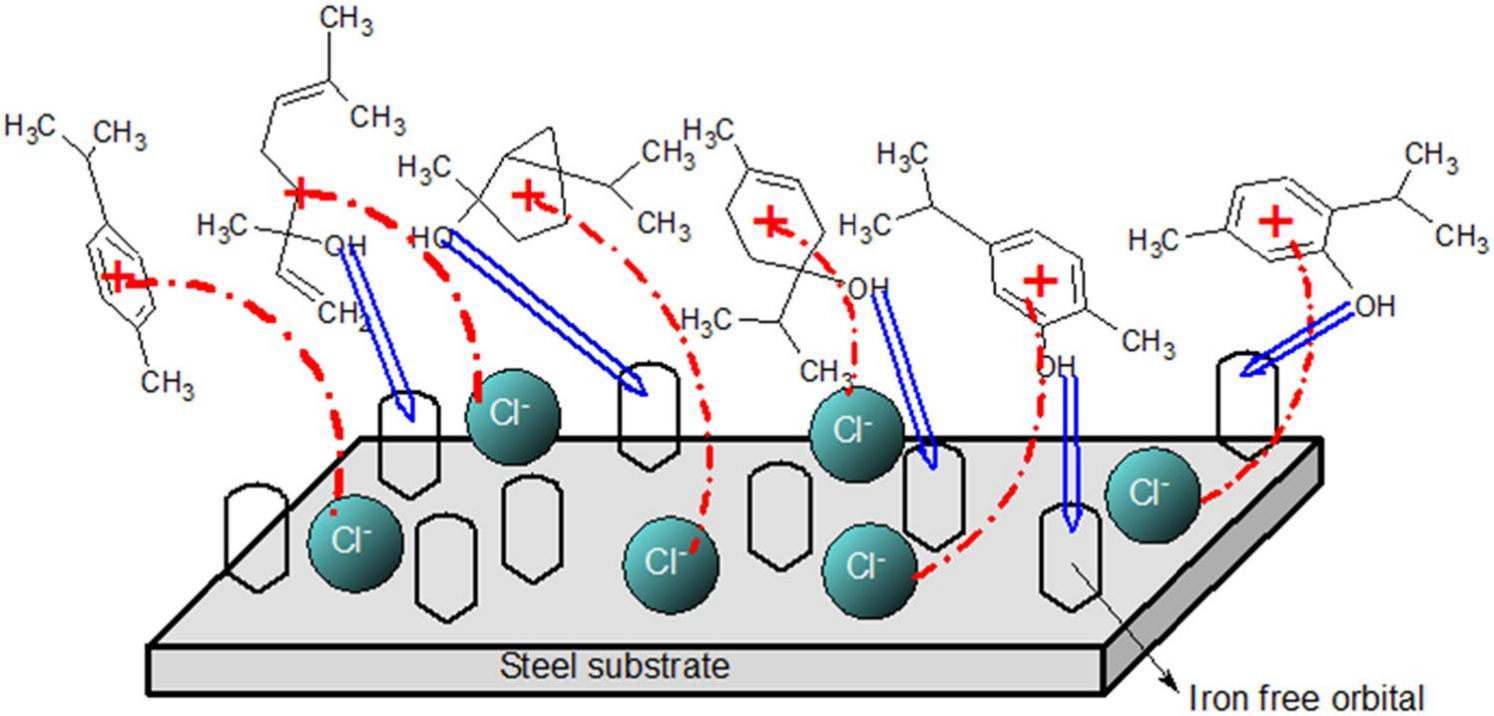
A schematic of absorption mechanisms of the Marjoram extract compounds on the surface of an MS electrode immersed in an HCl environment.

### Schematic of the adsorption process

A schematic of the adsorption mechanisms of the Marjoram extract compounds on the MS surface immersed in an acidic environment is shown in Fig. 10.

In the first stage, the anodic reaction of the corrosion produces a positively charged region near the immersed electrode surface due to the presence of Fe^2+^ and Fe^3+^ in this region. The positively charged region causes the attraction of Cl^−^ from the electrolyte toward the metal surface and form a negatively charged layer on the surface of the immersed electrode^41^. At the next stage, the protonated organic compounds in the solution adsorb on the MS surface through an electrostatic attraction and forms a compact layer on the electrode/electrolyte interface^57,58^. Finally, unsaturated pair electrons on oxygen atoms and π-bonds in the chemical structure of the Marjoram extract compounds can be shared with vacant iron orbitals on the electrode surface and lead to chemical adsorption. Therefore, the physicochemical adsorbed layer on the surface of the MS prevents direct contact of the electrode surface with the corrosive environment and reduces the corrosion rate of the metal^47,59^.

### Theoretical investigations

DFT is one of the most powerful tools in quantum chemistry. It is the shooting star in theoretical modeling. The optimized chemical structures of the present compounds in the optimum extract (Marjoram) are presented in Table S3 (in supplementary information). In the literature^60,61^, quantum calculations for the main substances present in the extract are performed to predict the mechanism of the compounds to protect the working electrode surface from corrosion reactions. In this study, DFT calculations were performed for 8 active substances in the Marjoram called Thymol, Carvacrol, β_Myrcene, Linalool, Caryophyllene, Cymene, Terpinene, and Sabinene as described by Lopez et al.^34^. Calculated parameters such as the energy of the highest orbital of the occupied E_HOMO_ molecule, the lowest of the non-occupied molecular orbital of E_LUMO_, the energy distance between HOMO and LUMO (ΔE), the ionization potential (IP), the electron affinity (E_a_), the electronegativity (χ), hardness (σ), and ΔN (the fraction of electrons that move from the molecule to the metal surface) for the molecules are given in Table 1. Parameters IP, E_A_, χ, σ, and ΔN are obtained using the following equations^62,63^.

9 $$ {\text{IP }} = \, - {\text{E}}_{{{\text{HOMO}}}} $$

10 $$ {\text{E}}_{{\text{A}}} = \, - {\text{E}}_{{{\text{LUMO}}}} $$

11 $$ \chi \, = \, \left( {{\text{I}} + {\text{Ea}}} \right)/{2} $$

12 $$ \sigma \, = \, \left( {{\text{I}} - {\text{Ea}}} \right)/{2} $$

13 $$ \eta \, = { 1}/\sigma $$

14 $$ \Delta {\text{N}} = \left( { \, \chi_{{{\text{Fe}}}} - \, \chi_{{{\text{inh}}}} } \right)/{2}\left( {\sigma_{{{\text{Fe}}}} + \sigma_{{{\text{inh}}}} } \right) $$

In the last equation, χ_Fe_ is 7 eV, and σ_Fe_ is equal to zero.

Large amounts of E_HOMO_ indicate that the molecule tends to give electrons to the low-energy empty orbitals of the using electrode^64^. The increase in values of E_HOMO_ facilitates the adsorption process by affecting the transfer process between the adsorption layers. Therefore, the effectiveness of an inhibitor can be improved by enhancing the transferring process. From Table 1, it is clear that the E_HOMO_ values for the under-studied inhibitors decrease in the order; Sabinene > Thymol > Carvacrol > Terpinene > β_Myrcene > Linalool > Caryophyllene > Cymene. However, inhibiting molecules does not only give electrons to the empty orbital of the Fe atom, but electrons are also accepted from the occupied orbitals of the electrode. This process leads to the creation of the feedback bond. Thus, E_LUMO_ indicates the ability of an inhibitor to accept electrons from the electrode surface, which would definitely improve the adsorption and inhibition efficiency of the anti-corrosion agent on the steel surface^65^. The E_LUMO_ for the under-studied molecules followed the order: Sabinene > Linalool > Caryophyllene > Carvacrol > Thymol > Cymene > β_Myrcene > Terpinene, indicating that the Terpinene has a higher affinity to electron accept from the immersed electrode. ΔE = E_HOMO_−E_LUMO_ is another parameter whose low value indicates higher inhibition efficiency of the corrosion inhibitor^66^. The obtained results (Table 1) revealed that the Cymene molecule has a fewer ΔE than the other molecules. Therefore, the Cymene molecule has a greater effect on protecting the immersed electrode from corrosion in the acidic environment. ΔN is another important quantum parameter in the study of anti-corrosion agents. Generally, positive and negative ΔN values indicate electron transfer from the anti-corrosion agent to the electrode surface and from the electrode surface to the anti-corrosion molecule, respectively^65,67^. It has also been reported that values of less than 3.6 indicate a higher electron-donating power of the molecule resulting in better molecular inhibition performance. Based on the results in Table 1, the values of ΔN are positive for all molecules (except for β_Myrcene), and their value is less than 3.6 (except for Sabinene). Thus, all molecules (except for β_Myrcene and Sabinene) are capable of forming covalent bonds to the immersed electrode surface and act as an effective protection agent for the used mild steel against corrosion reactions.

In an attempt to further evaluate the molecular reactivity and stability of the inhibitors, the absolute hardness (η) and softness (σ) were determined. The absolute softness and hardness are related to soft and hard solutions through the theory of HSAB^68,69^. Chemical hardness indicates the polarization resistance of the electron cloud of molecules, atoms, or ions with minor perturbations of the chemical reaction. The absolute hardness for the under-studied molecules was reduced in the following order: Sabinene > Caryophyllene > Linalool > Cymene > Carvacrol > Thymol > β_Myrcene > Terpinene. Terpinene, with the lowest hardness value (1.805 eV) compared with other compounds, had the lowest ∆E, while Sabinene with the highest hardness value (3.165 eV), had the highest ∆E. The softness followed a reverse trend of hardness values. This result was consistent with the general belief that hard compounds should have a large ∆E and soft molecules should have small ∆E. Therefore, Terpinene is expected to have higher inhibition performance than other compounds since the lowest global hardness value (i.e., the highest global softness) was likely the highest inhibition performance.

The dipole moment (µ) of compounds gives information about the polarity in the bond of a molecule and the distribution of electrons in the molecules. It is well known that a higher dipole moment value of an inhibitor tends to more adsorption tendency of the compound on the immersed electrode surface^70–72^. The µ value for the under-studied compounds was reduced in the following order: Carvacrol > Linalool > Thymol > Terpinene > Caryophyllene > β_Myrcene > Sabinene > Cymene. Although there is inconsistency with the use of the parameter to predict the direction of a corrosion inhibition reaction in literature; however, it is well known that the adsorption of polar molecules with more values of µ on the surface of the electrode should enhance inhibition performance.

The distribution of HOMO and LUMO orbitals can also be used to investigate how the chemical structure is adsorbed on the immersed electrode surface. The distribution of the HOMO and LUMO orbitals for the under-studied molecules is shown in Table S3. From the distribution of the HOMO and LUMO orbitals of all molecules, it can be concluded that the HOMO orbitals are more focused on double-bonded carbon atoms (C=C) and oxygen atoms. As a result, this portion of the molecule is adsorbed to the immersed electrode surface.

## Conclusion

1. Electrochemical analyses revealed that aqueous extracts of Wormwood, Maurorum, Yarrow, R. rubrum, and Marjoram herbal plants are suitable NIs for MS in 1M HCl electrolyte and their efficiencies increase with dosage in the order of Marjoram > Yarrow > Wormwood > Maurorum > R. rubrum.
2. The highest inhibition performance belonged to the Marjoram extract with an inhibition efficiency of about 92%.
3. The obtained results from the adsorption isotherm investigations proved that the adsorption of the organic compounds in the Marjoram extract acted as a mixed-type NI and obeyed the Langmuir model.
4. It was demonstrated that the organic compounds formed a protective layer on the electrode surface via physicochemical interactions.
5. UV–Vis and FE-SEM results confirmed the adsorption of the NI from the corrosive electrolyte on the MS surface.
6. DFT computation showed that the oxygen atoms and π-bonds are suitable sites for sharing their lone pair electrons with empty iron orbitals on the soaked MS surface.

## Supplementary Information

Supplementary Information.

## Data Availability

All data generated or analysed during this study are included in this published article [and its supplementary information files].
