# Supplementary material for: Preparation of corrosion inhibitor from natural plant for mild stil immersed in an acidic environmental: experimental and theoretical study
Source: Sci Rep. 2024 Apr 4;14:7937. doi: 10.1038/s41598-024-58637-z (PMC11371809; doi:10.1038/s41598-024-58637-z)
Supplement: Supplementary file 1 — Supplementary Information. [file 41598_2024_58637_MOESM1_ESM.docx]

Supporting Information

**Preparation of Corrosion Inhibitor from Natural Plant for Mild Step Immersed in an Acidic Environmental: Experimental and Theoretical Study**

Maryam Pourmohseni^a^, Alimorad Rashidi^b^*, Mehrnoosh Karimkhani^a^

^a^Department of Chemistry Central Tehran Branch, Islamic Azad University, Tehran, Iran

^b^*Nanotechnology Research Center, Research Institute of Petroleum Industry (RIPI), Tehran, Iran

Corresponding author.

E-mail addresses: ^b^*[rashidiam@ripi.ir](mailto:rashidiam@ripi.ir)

Fig S1 shows the FT-IR spectrum of the yarrow plant extract used in this study. Fig S2 represents some chemical compounds that can be found in this plant obtained by Khasneh et al. [1].





Fig S1. FT-IR spectrum of the Yarrow plant extract


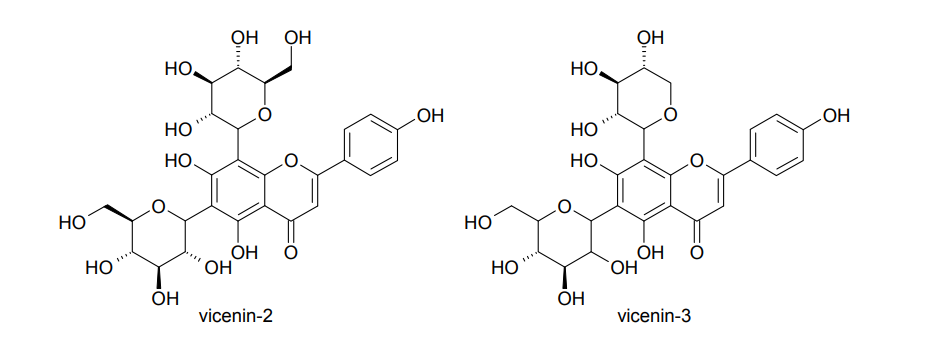


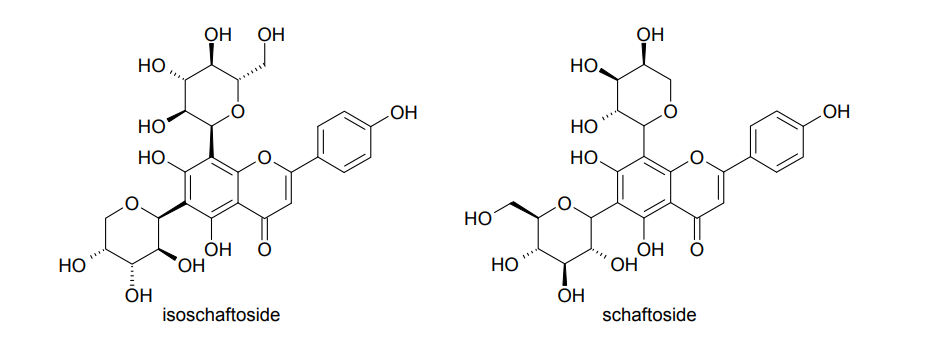


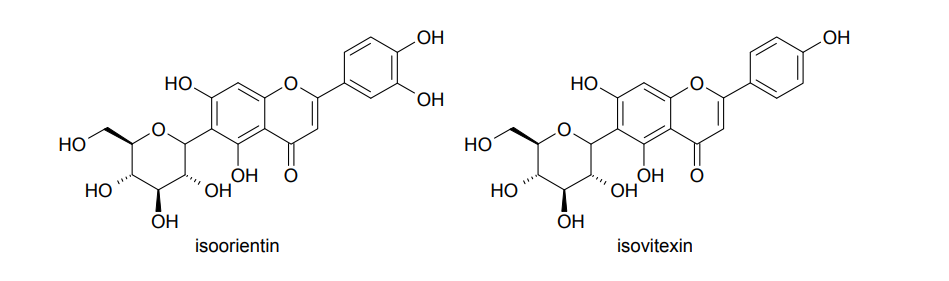


Fig S2. Some of the most essential main chemical compounds in Yarrow extract [1]

According to Fig S2, the presence of C-H, C-OH, C-O-C, and C=O bonds as well as numerous aromatic rings (C=C bonds), can be seen in the extract of this medicinal plant. In the FT-IR spectrum of this sample, the peak located in the wavenumber range of 3000 to 3800 cm^-1^ is related to the stretching vibration of O-H bonds [2], arises from the hydroxyl groups present in the compounds or adsorbed moisture on the sample. The peak of O-H bending vibration can be seen at 1624 cm^-1^ [3]. The asymmetric and symmetric stretching vibrations of C-H bonds in alkyl groups cause the peaks at 2927 and 2873 cm^-1^, respectively [4,5]. The absorption peak at 1723 cm^-1^ can be related to the stretching vibration of the C=O bonds in the chemical compounds [6]. The appeared peaks at 1068, 1396, and 1511 cm^-1^ are due to the stretching vibration of C-O bonds (in the structure of C-O-C and C-OH functional groups), the bending vibration of C-H bonds, and the stretching vibrations of C=C bonds in aromatic rings, respectively [7,8]. The absorption peak at 517 cm^-1^ is related to the bending vibration of the C-H bonds connected to the aromatic rings in the structure of the extract [8].

The FT-IR spectrum of the Maurorum herbal extract, and the compounds that can be detected in this stract (reviewed by Ahmed et al. [9]), are shown in Fig S3 and Fig S4, respectively.





Fig S3. FT-IR spectrum of the Maurorum plant extract


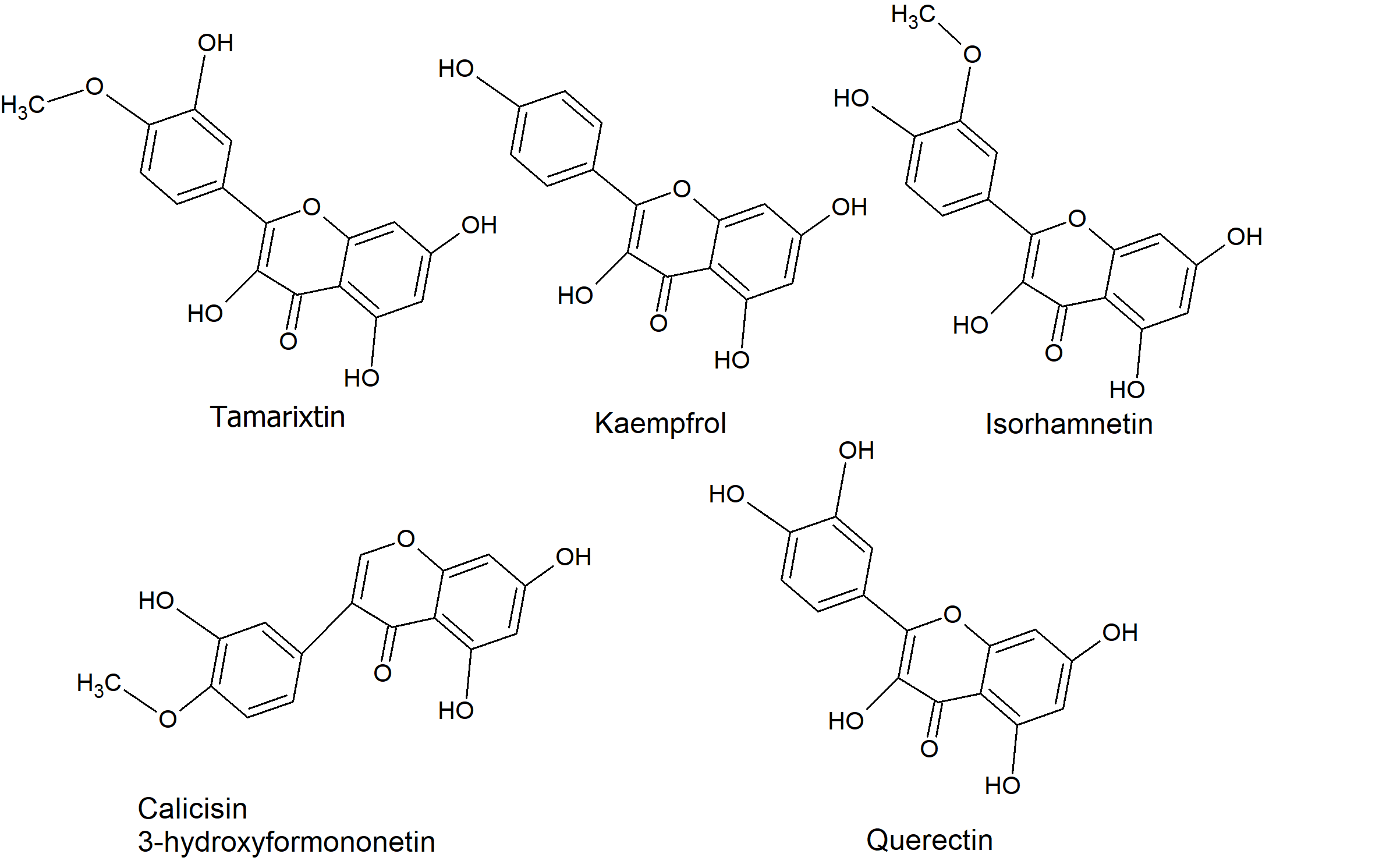


Fig S4. Detected compounds in the Maurorum extract [9]

From Fig S4, the presence of C-H, C-O-C, C-OH, C=O, C=C, and C-OH bonds can be proved in the Maurorum extract. As can be seen in the FT-IR spectrum for this extract, the appeared peaks at 3367 and 1646 cm^-1^ are due to the stretching and bending vibrations of O-H bonds [10,11]. The absorption peak at 2927 cm^-1^ is related to the stretching vibration of C-H bond in alkyl compounds [6]. The absorption peak at 1731 cm^-1^ arises from the stretching vibration of C=O bonds in the carboxylic and carbonyl structures [12]. The peaks at 1419 and 1646 cm^-1^ are due to the bending and stretching vibrations of C-H and C=C bonds, respectively. Absorption peaks at 1145 and 1064 cm^-1^ can be attributed to the stretching vibrations of C-OH and C-O-C bonds, respectively [13]. The absorption peaks at 991 and 925 cm^-1^ are due to the bending vibration of C-H bonds in CH_2_ and CH_3_ groups [8]. Furthermore, the peaks at the wavenumbers < 900 cm^-1^ resulting from the bending vibration of aromatic rings in the extract compounds and the bending vibration of C-H bonds in aromatic rings [10].

The FT-IR spectrum of the R. Rubrum, as well as the compounds that can be isolated from the medicinal plant, obtained by Stefaniou et al. [14], are shown in Fig S5 and Fig S6, respectively.





Fig S5. FT-IR spectrum of the Ribes Rubrum extract


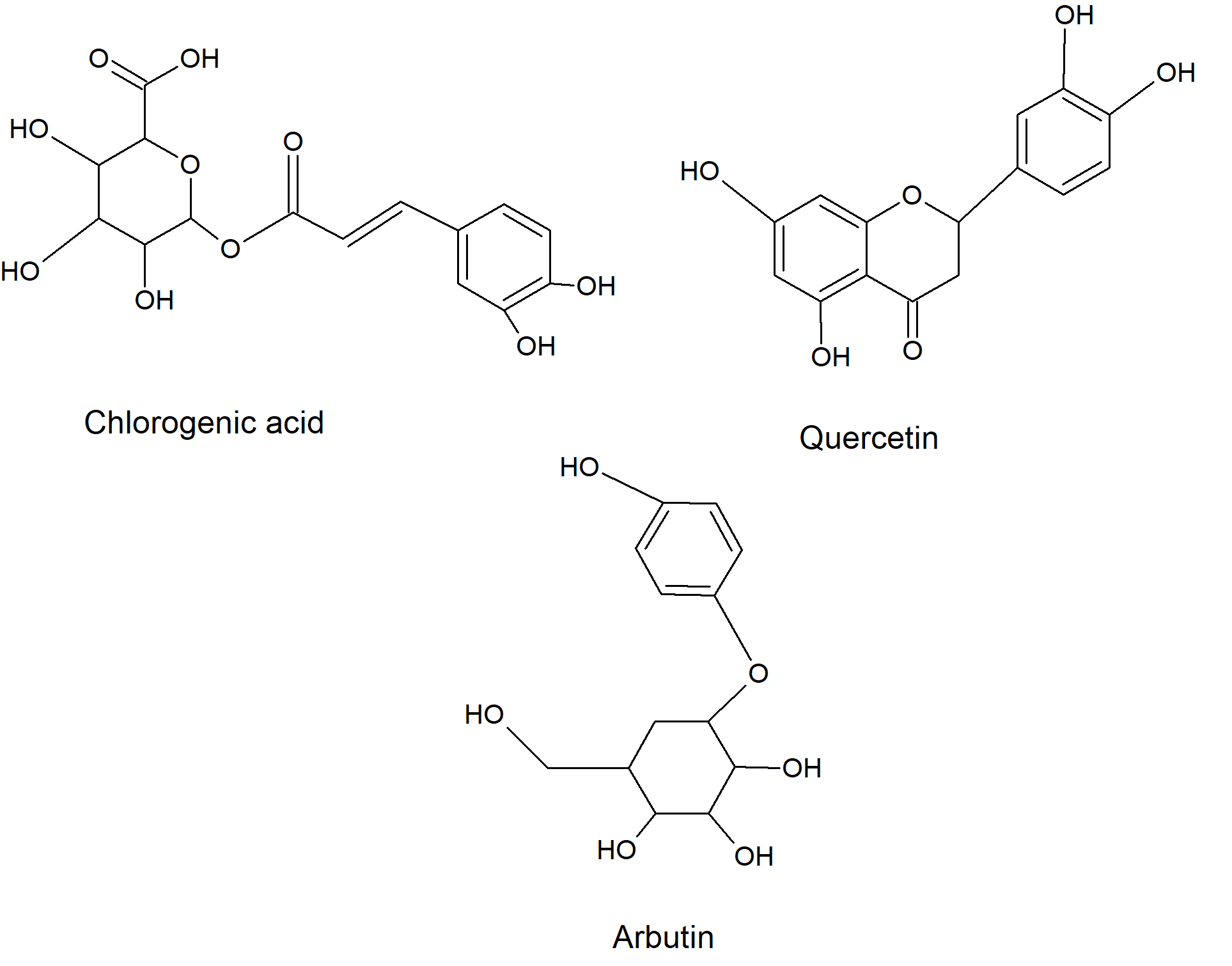


Fig S6. The isolated compounds from the Ribes rubrum extract [14]

The presence of C-C, C-H, C-O-C, C-OH, C=O, and C-OH bonds can be seen in the isolated compounds from the R. rubrum extract. In the FT-IR spectrum of the R. rubrum extract, the located peaks at 3432 and 1623 cm^-1^ are respectively due to the stretching and bending vibrations of the O-H bonds in the hydroxyl groups and adsorbed moisture [15]. The peaks of symmetric and asymmetric stretching vibrations of C-H bonds can be seen at 2857 and 2923 cm^-1^, respectively [16]. the absorption peak at 1454 cm^-1^can be related to the stretching vibration of C=C bonds in aromatic rings and the bending vibration of C-H bonds [8]. According to Fig S6, two types of carboxyl groups can be seen in the compounds, one is attached to the aromatic ring, and the other is in an aliphatic structure. These carboxyl groups caused the formation of two peaks at 1731 and 1793 cm^-1^ [17]. The C-OH and C-O-C bonds in the structure of the compounds have also shown absorption peaks at 1241 and 1072 cm^-1^ [18]. Additionally, the peaks in the wavenumber range of 400- 900 cm^-1^ belong to the bending vibration of aromatic rings in the extract compounds and the bending vibration of C-H bonds in aromatic rings [8].

The FT-IR spectrum of the Wormwood herbal extract and its chemical compounds obtained by Eszopa et al.[19], are shown in Fig S7 and Fig S8, respectively.





Fig S7. FT-IR spectrum of Wormwood plant extract


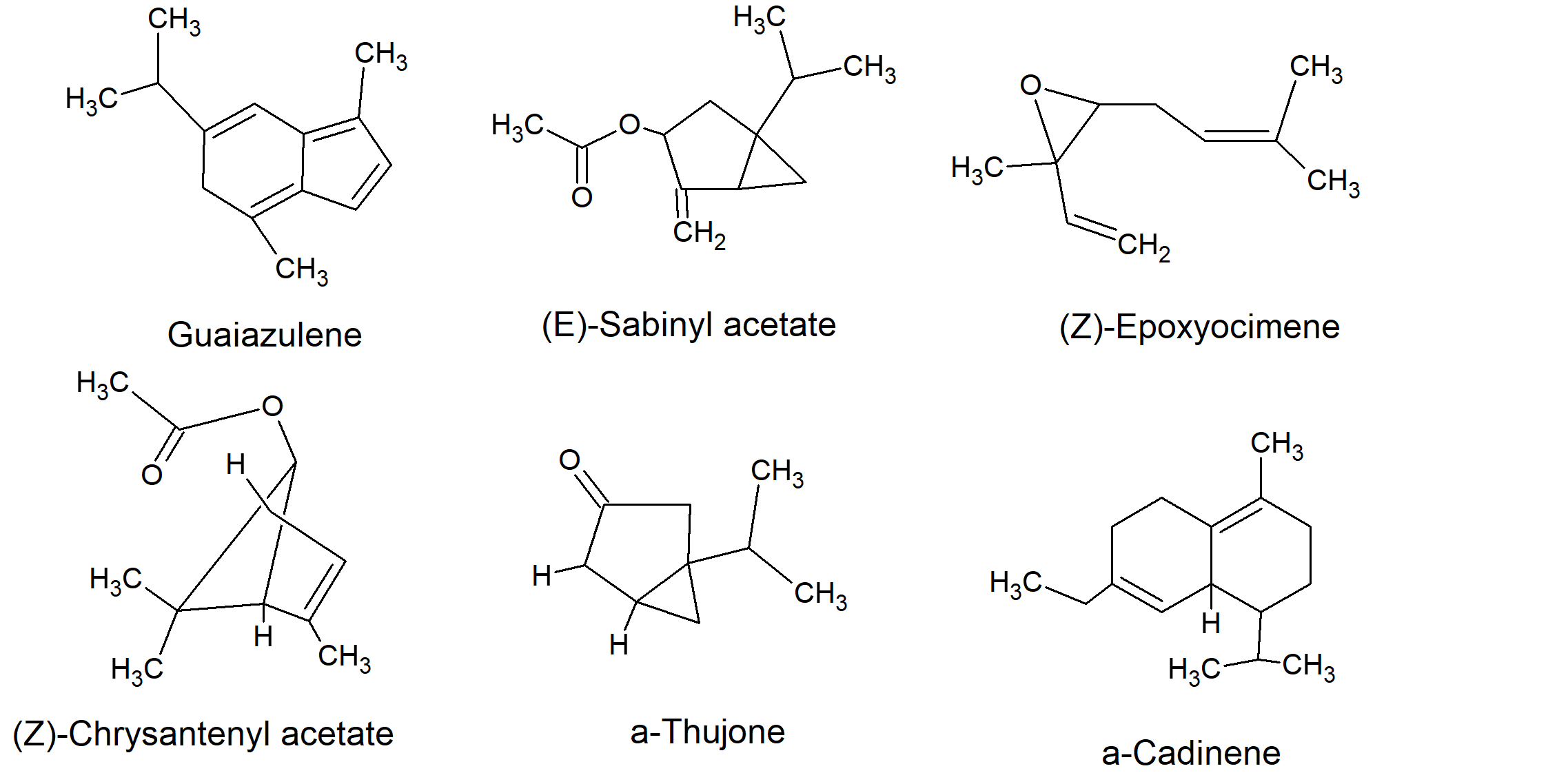


Fig S8. The isolated compounds from the Wormwood herbal extract [19]

According to Fig S8, the presence of C-H, C-O-C, C-OH, C=O, C=C, and C-OH bonds can be seen in the isolated chemical compounds from the Wormwood extract. In the FT-IR spectrum (Fig S7), the appeared peaks at 3424 and 1619 cm^-1^ are due to the stretching and bending vibrations of O-H bonds, respectively. The stretching vibration of C-H bonds in alkyl compounds also showed an absorption peak at 2850 cm^-1^ [6]. The stretching vibration of C=O bonds in the carboxylic structure has caused an absorption peak at 1739 cm^-1^ [6]. The peaks at 1396 and 1511 cm^-1^ are due to the bending and stretching vibrations of C-H and C=C bonds in aromatic rings, respectively. The peaks of stretching vibrations of C-OH and C-O-C bonds appeared at 1265 cm^-1^ and 1060 cm^-1^, respectively [8,13]. Moreover, the peaks at wavenumbers less than 900 cm^-1^ belong to the bending vibrations of C-H bonds and aromatic rings in the Wormwood extract [8].

a

Fig S9. Nyquist curves of (a) the blank sample and samples with the Yarrow extract at concentrations of (b) 200, (c) 400, (d) 600, and (e) 800 ppm, the *Wormwood* extract at concentrations of (f) 200, (g) 400, (h) 600 and (i) 800 ppm, the Ribes rubrum extract at concentrations of (j) 200, (k) 400, (l) 600 and (m) 800 ppm, the Marjoram extract at the concentrations of (n) 200, (o) 400, (p) 600 and (q) 800 ppm, and the Maurorum extract at concentrations of (r) 200, (s) 400, (t) 600 and (w) 800 ppm. Markers and solid lines represent experimental and fitted data, respectively.

a

Fig S10. Bode curves of (a) the blank sample and samples with the Yarrow extract at concentrations of (b) 200, (c) 400, (d) 600, and (e) 800 ppm, the *Wormwood* extract at concentrations of (f) 200, (g) 400, (h) 600 and (i) 800 ppm, the Ribes rubrum extract at concentrations of (j) 200, (k) 400, (l) 600 and (m) 800 ppm, the Marjoram extract at the concentrations of (n) 200, (o) 400, (p) 600 and (q) 800 ppm, and the Maurorum extract at concentrations of (r) 200, (s) 400, (t) 600 and (w) 800 ppm. Markers and solid lines represent experimental and fitted data, respectively.

Table S1. Electrochemical parameters resulting from modeling impedance test results on the electrochemical equivalent circuit

| *%IE* | *Cdl* (μF/cm2) | *R_ct_* (ohm.cm^2^) | *n* | *CPE, Y0* (S.sec^n^/cm^2^ ( | *R_s_* (ohm.cm^2^) | Immersion time | Concentration (ppm) | Sample |
| --- | --- | --- | --- | --- | --- | --- | --- | --- |
|  | 1251.74 | 46.24 | 0.88 | 3.37E-03 | 0.209 | 15 min |  | No inhibitor |
|  | 1764.21 | 40.48 | 0.89 | 4.13E-03 | 0.25 | 2 h |  |  |
|  | 1771.31 | 33.41 | 0.89 | 4.14E-03 | 0.253 | 4 h |  |  |
|  | 2048.84 | 23.77 | 0.88 | 5.22E-03 | 0.203 | 6 h |  |  |
|  | 2157.71 | 17.33 | 0.89 | 5.16E-03 | 0.169 | 24 h |  |  |
| 18.04 | 371.71 | 56.42 | 0.76 | 3.23E-03 | 0.331 | 15 min | 200 | Yarrow |
| 54.56 | 228.75 | 89.08 | 0.82 | 1.34E-03 | 0.238 | 2 h |  |  |
| 69.77 | 246.52 | 110.53 | 0.84 | 1.17E-03 | 0.241 | 4 h |  |  |
| 79.41 | 238.36 | 115.44 | 0.84 | 1.13E-03 | 0.251 | 6 h |  |  |
| 88.28 | 146.14 | 147.85 | 0.81 | 1.05E-03 | 0.213 | 24 h |  |  |
| 35.40 | 521.78 | 71.58 | 0.79 | 3.13E-03 | 0.38 | 15 min | 400 |  |
| 59.78 | 339.06 | 100.64 | 0.87 | 1.11E-03 | 0.323 | 2 h |  |  |
| 73.78 | 533.51 | 127.41 | 0.88 | 1.57E-03 | 0.233 | 4 h |  |  |
| 84.39 | 605.14 | 152.27 | 0.89 | 1.59E-03 | 0.254 | 6 h |  |  |
| 90.97 | 172.84 | 191.98 | 0.82 | 1.03E-03 | 0.286 | 24 h |  |  |
| 45.52 | 321.74 | 84.88 | 0.75 | 3.03E-03 | 0.397 | 15 min | 600 |  |
| 63.45 | 237.27 | 110.74 | 0.86 | 9.13E-04 | 0.279 | 2 h |  |  |
| 78.15 | 236.64 | 152.93 | 0.88 | 7.51E-04 | 0.28 | 4 h |  |  |
| 85.81 | 224.98 | 167.52 | 0.88 | 7.18E-04 | 0.281 | 6 h |  |  |
| 92.51 | 190.35 | 231.41 | 0.82 | 1.05E-03 | 0.399 | 24 h |  |  |
| 60.77 | 420.14 | 117.88 | 0.76 | 3.23E-03 | 0.487 | 15 min | 800 |  |
| 78.66 | 350.25 | 189.66 | 0.87 | 1.11E-03 | 0.401 | 2 h |  |  |
| 85.61 | 306.17 | 232.22 | 0.88 | 9.07E-04 | 0.384 | 4 h |  |  |
| 90.55 | 353.51 | 251.55 | 0.87 | 1.12E-03 | 0.398 | 6 h |  |  |
| 94.14 | 249.57 | 295.93 | 0.82 | 1.28E-03 | 0.456 | 24 h |  |  |
| 8.45 | 437.98 | 50.51 | 0.75 | 3.35E-03 | 0.676 | 15 min | 200 | Wormwood |
| 57.70 | 369.45 | 95.7 | 0.87 | 1.17E-03 | 0.383 | 2 h |  |  |
| 73.34 | 338.40 | 125.33 | 0.88 | 1.02E-03 | 0.301 | 4 h |  |  |
| 82.87 | 4694.70 | 138.77 | 0.89 | 9.61E-03 | 0.317 | 6 h |  |  |
| 88.95 | 206.50 | 156.84 | 0.83 | 1.08E-03 | 0.288 | 24 h |  |  |
| 12.47 | 368.01 | 52.83 | 0.75 | 3.03E-03 | 0.598 | 15 min | 400 |  |
| 62.70 | 252.21 | 108.53 | 0.86 | 9.05E-04 | 0.433 | 2 h |  |  |
| 75.22 | 251.63 | 134.85 | 0.85 | 1.03E-03 | 0.331 | 4 h |  |  |
| 84.66 | 1857.54 | 154.94 | 0.8 | 7.84E-03 | 0.403 | 6 h |  |  |
| 90.52 | 432.21 | 182.73 | 0.75 | 3.32E-03 | 0.667 | 24 h |  |  |
| 12.41 | 561.73 | 52.79 | 0.76 | 3.76E-03 | 0.654 | 15 min | 600 |  |
| 66.05 | 325.05 | 119.22 | 0.86 | 1.16E-03 | 0.349 | 2 h |  |  |
| 76.46 | 443.83 | 141.93 | 0.88 | 1.27E-03 | 0.354 | 4 h |  |  |
| 87.51 | 342.64 | 190.33 | 0.89 | 9.12E-04 | 0.399 | 6 h |  |  |
| 92.62 | 305.04 | 234.8 | 0.83 | 1.48E-03 | 0.303 | 24 h |  |  |
| 14.72 | 631.23 | 54.22 | 0.75 | 4.43E-03 | 0.661 | 15 min | 800 |  |
| 72.04 | 286.41 | 144.8 | 0.86 | 9.65E-04 | 0.598 | 2 h |  |  |
| 78.81 | 323.60 | 157.65 | 0.89 | 8.36E-04 | 0.555 | 4 h |  |  |
| 89.52 | 272.24 | 226.75 | 0.88 | 7.83E-04 | 0.553 | 6 h |  |  |
| 93.90 | 309.24 | 283.92 | 0.84 | 1.34E-03 | 0.339 | 24 h |  |  |
| 13.46 | 261.17 | 53.43 | 0.8 | 1.82E-03 | 0.234 | 15 min | 200 | Ribes rubrum |
| 28.96 | 312.04 | 56.98 | 0.82 | 1.60E-03 | 0.367 | 2 h |  |  |
| 51.05 | 356.26 | 68.26 | 0.87 | 1.16E-03 | 0.321 | 4 h |  |  |
| 73.12 | 345.13 | 88.42 | 0.88 | 1.03E-03 | 0.321 | 6 h |  |  |
| 86.02 | 207.53 | 123.98 | 0.85 | 9.04E-04 | 0.265 | 24 h |  |  |
| 21.49 | 360.91 | 58.9 | 0.82 | 1.87E-03 | 0.299 | 15 min | 400 |  |
| 36.08 | 262.46 | 63.33 | 0.84 | 1.19E-03 | 0.302 | 2 h |  |  |
| 58.80 | 462.79 | 81.09 | 0.86 | 1.56E-03 | 0.369 | 4 h |  |  |
| 76.97 | 312.67 | 103.21 | 0.85 | 1.22E-03 | 0.367 | 6 h |  |  |
| 86.78 | 240.32 | 131.11 | 0.84 | 1.11E-03 | 0.293 | 24 h |  |  |
| 22.51 | 636.83 | 59.67 | 0.75 | 4.47E-03 | 0.654 | 15 min | 600 |  |
| 41.25 | 1006.89 | 68.9 | 0.91 | 1.97E-03 | 0.578 | 2 h |  |  |
| 61.46 | 717.97 | 86.68 | 0.9 | 1.56E-03 | 0.598 | 4 h |  |  |
| 78.63 | 581.98 | 111.25 | 0.89 | 1.41E-03 | 0.554 | 6 h |  |  |
| 87.82 | 495.54 | 142.33 | 0.83 | 2.37E-03 | 0.203 | 24 h |  |  |
| 30.94 | 666.25 | 66.96 | 0.74 | 4.62E-03 | 0.886 | 15 min | 800 |  |
| 48.73 | 433.91 | 78.96 | 0.88 | 1.17E-03 | 0.597 | 2 h |  |  |
| 64.99 | 405.83 | 95.42 | 0.89 | 1.03E-03 | 0.521 | 4 h |  |  |
| 81.48 | 653.64 | 128.34 | 0.9 | 1.43E-03 | 0.612 | 6 h |  |  |
| 89.23 | 542.55 | 160.89 | 0.85 | 1.94E-03 | 0.378 | 24 h |  |  |
| 22.78 | 98.19 | 59.88 | 0.73 | 1.33E-03 | 0.662 | 15 min | 200 | Marjoram |
| 68.23 | 354.01 | 127.43 | 0.84 | 1.39E-03 | 0.55 | 2 h |  |  |
| 78.07 | 338.41 | 152.35 | 0.87 | 1.05E-03 | 0.489 | 4 h |  |  |
| 85.85 | 337.08 | 167.97 | 0.88 | 9.54E-04 | 0.511 | 6 h |  |  |
| 91.46 | 188.85 | 202.99 | 0.83 | 1.01E-03 | 0.276 | 24 h |  |  |
| 31.89 | 4.25 | 67.89 | 0.74 | 1.10E-04 | 0.877 | 15 min | 400 |  |
| 70.95 | 347.40 | 139.33 | 0.86 | 1.12E-03 | 0.676 | 2 h |  |  |
| 80.19 | 342.19 | 168.66 | 0.89 | 8.58E-04 | 0.689 | 4 h |  |  |
| 87.73 | 319.45 | 193.65 | 0.89 | 7.90E-04 | 0.837 | 6 h |  |  |
| 93.58 | 187.74 | 270.11 | 0.85 | 7.25E-04 | 0.654 | 24 h |  |  |
| 41.65 | 773.23 | 79.24 | 0.75 | 4.11E-03 | 1.654 | 15 min | 600 |  |
| 74.50 | 369.52 | 158.77 | 0.86 | 1.12E-03 | 0.989 | 2 h |  |  |
| 83.19 | 357.67 | 198.76 | 0.89 | 8.59E-04 | 0.976 | 4 h |  |  |
| 89.63 | 356.33 | 229.11 | 0.9 | 7.89E-04 | 0.995 | 6 h |  |  |
| 95.07 | 198.07 | 351.19 | 0.85 | 7.25E-04 | 0.886 | 24 h |  |  |
| 53.99 | 451.94 | 100.5 | 0.82 | 1.84E-03 | 0.915 | 15 min | 800 |  |
| 81.16 | 282.59 | 214.85 | 0.87 | 8.14E-04 | 1.039 | 2 h |  |  |
| 85.56 | 382.51 | 231.43 | 0.91 | 7.73E-04 | 1.058 | 4 h |  |  |
| 91.77 | 361.98 | 288.94 | 0.9 | 7.95E-04 | 1.062 | 6 h |  |  |
| 95.50 | 288.43 | 384.77 | 0.9 | 6.42E-04 | 1.165 | 24 h |  |  |
| 15.59 | 623.30 | 54.78 | 0.79 | 3.32E-03 | 0.563 | 15 min | 200 | Maurorum |
| 49.24 | 302.07 | 79.75 | 0.85 | 1.21E-03 | 0.319 | 2 h |  |  |
| 68.01 | 373.62 | 104.44 | 0.86 | 1.37E-03 | 0.25 | 4 h |  |  |
| 79.44 | 2980.46 | 115.64 | 0.87 | 7.55E-03 | 0.264 | 6 h |  |  |
| 86.74 | 256.60 | 130.7 | 0.84 | 1.21E-03 | 0.241 | 24 h |  |  |
| 16.02 | 353.76 | 55.06 | 0.8 | 2.22E-03 | 0.292 | 15 min | 400 |  |
| 47.71 | 184.69 | 77.41 | 0.83 | 1.01E-03 | 0.248 | 2 h |  |  |
| 65.91 | 435.79 | 98.01 | 0.84 | 1.98E-03 | 0.179 | 4 h |  |  |
| 79.71 | 217.02 | 117.13 | 0.82 | 1.33E-03 | 0.195 | 6 h |  |  |
| 88.26 | 353.61 | 147.67 | 0.87 | 1.21E-03 | 0.22 | 24 h |  |  |
| 35.98 | 921.00 | 72.23 | 0.76 | 4.71E-03 | 1.23 | 15 min | 600 |  |
| 62.01 | 13173.24 | 106.55 | 0.84 | 2.03E-02 | 5.343 | 2 h |  |  |
| 70.98 | 4149.24 | 115.12 | 0.87 | 7.30E-03 | 3.211 | 4 h |  |  |
| 80.44 | 3911.33 | 121.55 | 0.88 | 6.79E-03 | 2.635 | 6 h |  |  |
| 89.83 | 2976.41 | 170.33 | 0.88 | 5.78E-03 | 1.342 | 24 h |  |  |
| 43.67 | 771.88 | 82.09 | 0.73 | 5.20E-03 | 1.122 | 15 min | 800 |  |
| 67.43 | 358.33 | 124.3 | 0.87 | 1.16E-03 | 0.333 | 2 h |  |  |
| 78.38 | 432.34 | 154.55 | 0.88 | 1.15E-03 | 0.669 | 4 h |  |  |
| 90.03 | 518.19 | 238.32 | 0.87 | 1.16E-03 | 3.987 | 6 h |  |  |
| 88.57 | 313.21 | 151.67 | 0.85 | 1.29E-03 | 0.255 | 24 h |  |  |

a

Fig S11. PP curves of the samples containing (a) the Yarrow extract, (b) the Marjoram extract, (c) the Maurorum extract, (d) the R. rubrum extract, and (e) the Wormwood extract after 24 hours of immersion in 1 M HCl solution

Table S2. Electrochemical parameters obtained from Tafel extrapolation of the PP curves

| *%IE* | *R_p_* (Ohm.cm^2^) | *i_corr_* (μA/cm^2^) | *E_corr_* SCE (V) | -*β_c_* (v.dec^-1^) | *β_a_* (v.dec^-1^) | Concentration (ppm) | Sample |
| --- | --- | --- | --- | --- | --- | --- | --- |
|  | 46.63 | 347.64 | -0.44 | 0.08 | 0.07 | 0 | Blank |
| 39.83 | 95.47 | 209.18 | -0.44 | 0.10 | 0.09 | 200 | Yarrow |
| 74.07 | 122.66 | 90.16 | -0.42 | 0.12 | 0.03 | 400 |
| 77.19 | 276.15 | 79.28 | -0.42 | 0.12 | 0.09 | 600 |
| 89.67 | 451.76 | 35.92 | -0.37 | 0.16 | 0.05 | 800 |
| 56.62 | 112.29 | 150.79 | -0.43 | 0.09 | 0.07 | 200 | Marjoram |
| 76.86 | 198.81 | 80.45 | -0.44 | 0.09 | 0.06 | 400 |
| 79.96 | 236.28 | 69.66 | -0.43 | 0.09 | 0.07 | 600 |
| 90.79 | 505.63 | 32.03 | -0.44 | 0.09 | 0.06 | 800 |
| 38.06 | 66.45 | 215.32 | -0.46 | 0.08 | 0.06 | 200 | Maurorum |
| 47.77 | 79.62 | 181.56 | -0.45 | 0.08 | 0.06 | 400 |
| 60.89 | 106.68 | 135.95 | -0.45 | 0.08 | 0.06 | 600 |
| 67.04 | 130.81 | 114.58 | -0.44 | 0.09 | 0.06 | 800 |
| 38.48 | 66.10 | 213.87 | -0.44 | 0.08 | 0.06 | 200 | Ribes Rubrum |
| 42.04 | 79.38 | 201.49 | -0.42 | 0.09 | 0.06 | 400 |
| 54.73 | 89.22 | 157.39 | -0.44 | 0.09 | 0.05 | 600 |
| 63.45 | 128.75 | 127.05 | -0.43 | 0.09 | 0.06 | 800 |
| 43.93 | 81.10 | 194.93 | -0.42 | 0.09 | 0.06 | 200 | Wormwood |
| 45.41 | 83.39 | 189.77 | -0.44 | 0.09 | 0.06 | 400 |
| 73.32 | 169.38 | 92.74 | -0.43 | 0.09 | 0.06 | 600 |
| 76.85 | 202.83 | 80.49 | -0.44 | 0.09 | 0.06 | 800 |

Table S3. From left to right: Compound name, optimized geometry, HOMO and LUMO orbitals for main constituent of Marjoram extract.

| Compound | Optimized geometry | HOMO | LUMO |
| --- | --- | --- | --- |
| Thymol | 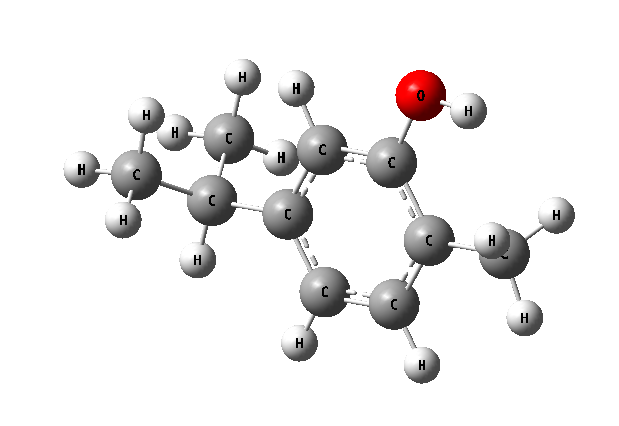 | 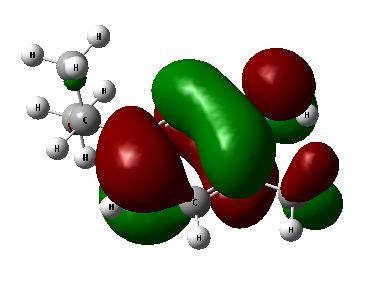 | 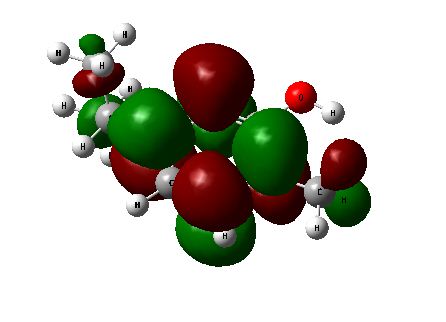 |
| β_Myrcene | 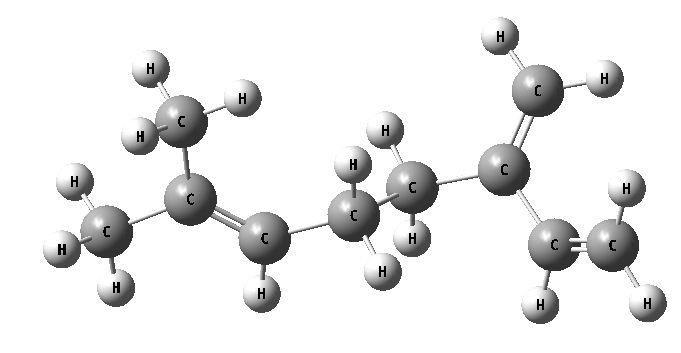 | 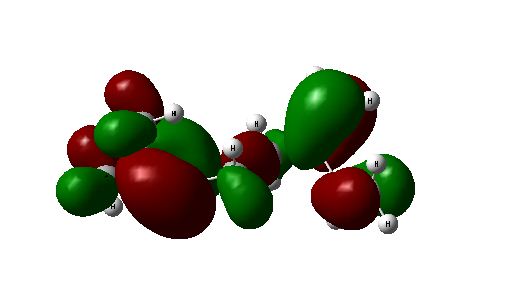 | 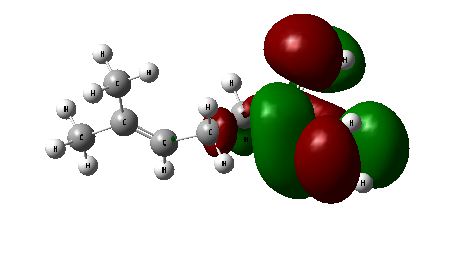 |
| Carvacrol | 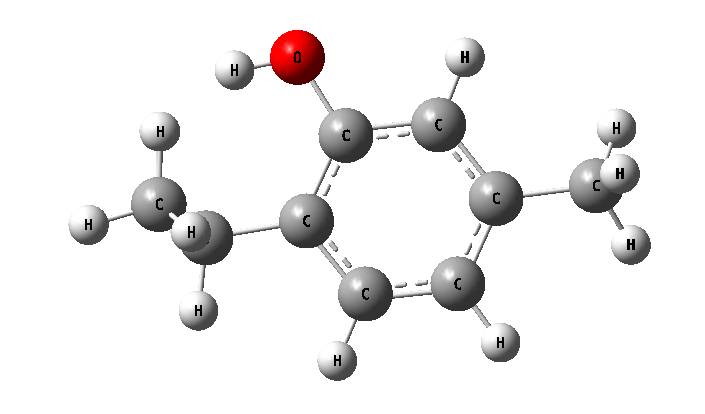 | 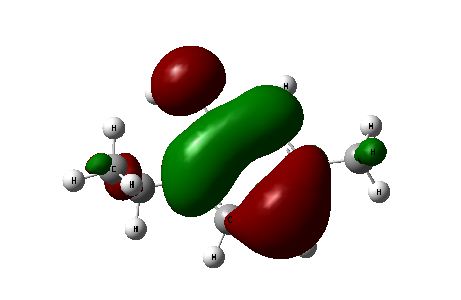 | 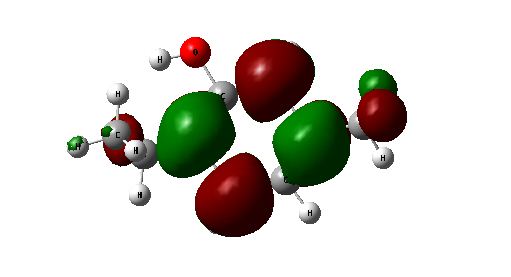 |
| Linalool | 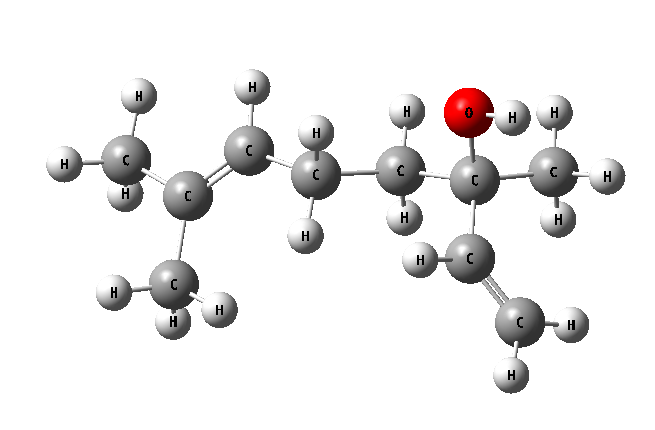 | 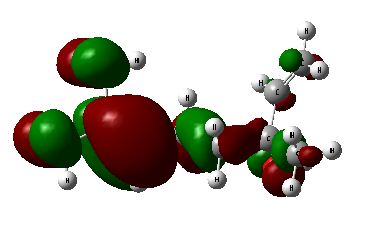 | 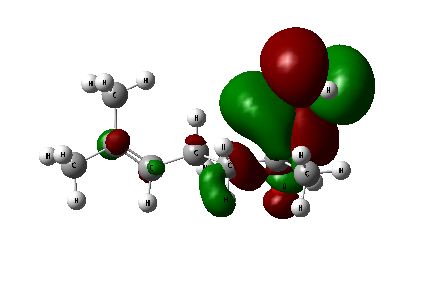 |
| Terpinene | 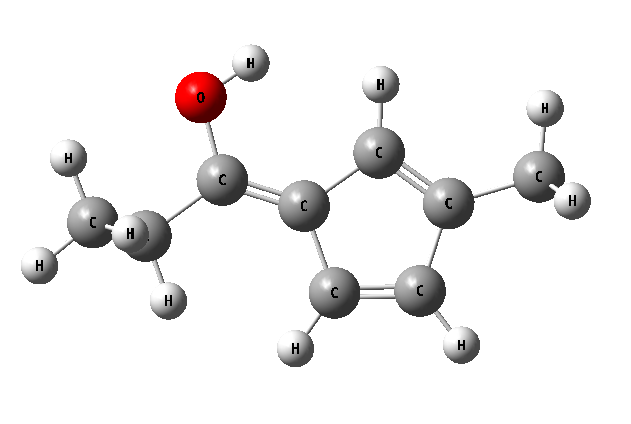 | 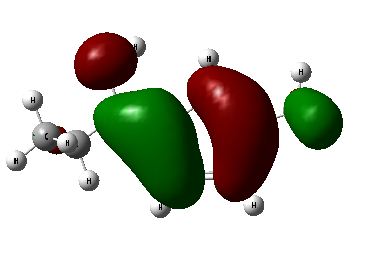 | 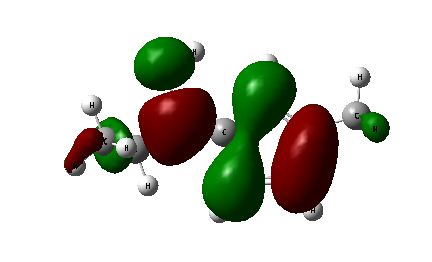 |
| Cymene | 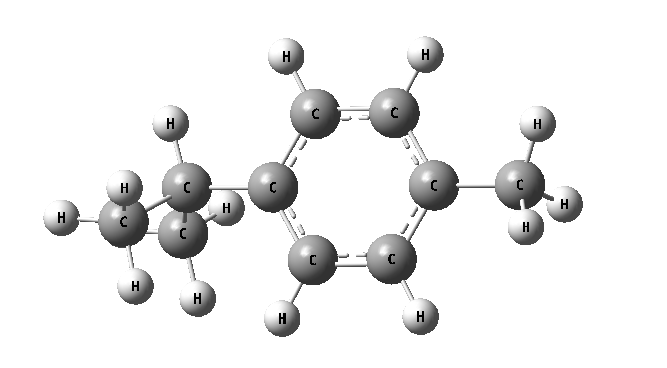 | 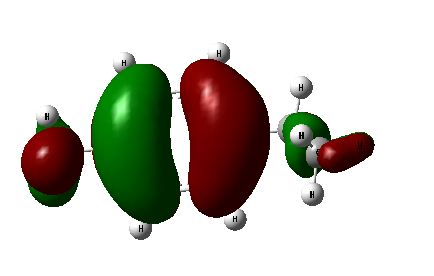 | 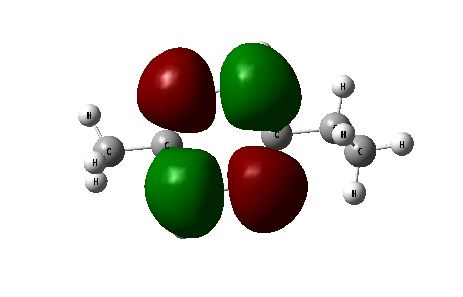 |
| Caryophyllene | 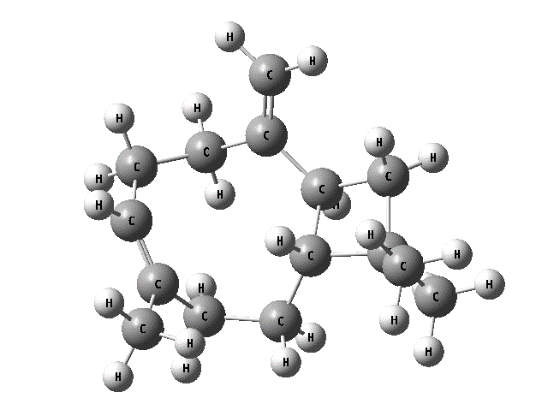 | 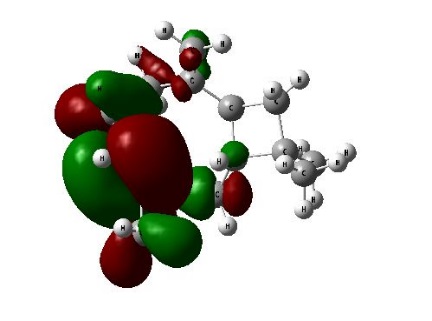 | 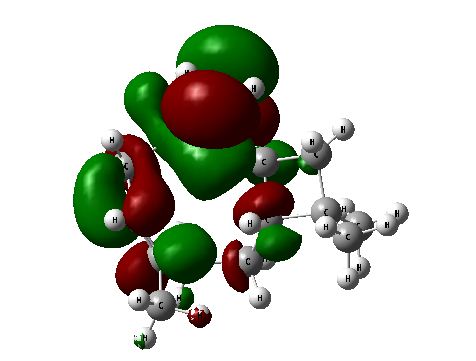 |
| Sabinene | 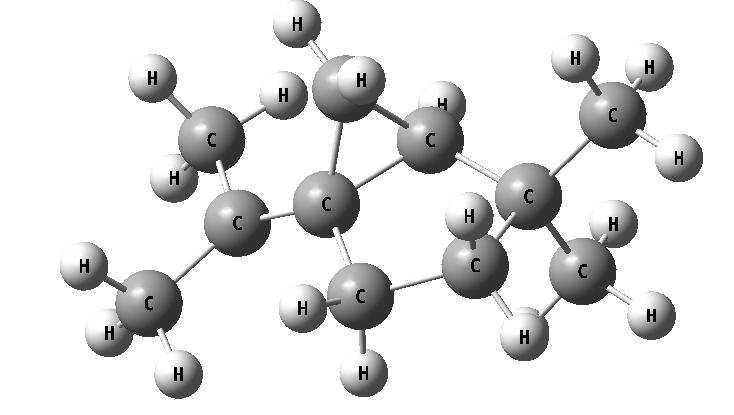 | 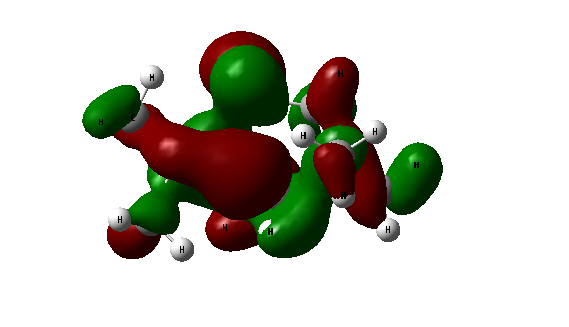 | 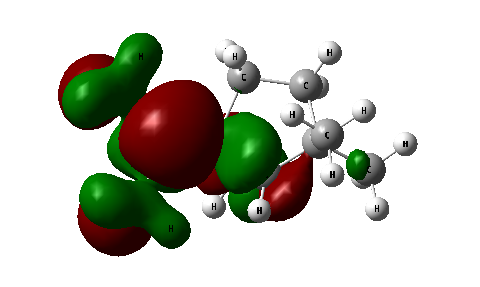 |

**Table S3: Raw data from polarization and impedance test**

A: polarization test

Sample: Blank (no inhibitor), Immersion time: 24h

| Potential applied(V) | Time (s) | WE(1).  Current (A) | WE(1).  Potential (V) | Index |  |  |
| --- | --- | --- | --- | --- | --- | --- |
| -0.70679 | 68.54436 | -9.3E-05 | -0.70468 | 1 |  |  |
| -0.70572 | 69.61248 | -0.00093 | -0.70364 | 2 |  |  |
| -0.70465 | 70.68059 | -0.00768 | -0.70294 | 3 |  |  |
| -0.70358 | 71.74871 | -0.00764 | -0.70184 | 4 |  |  |
| -0.70251 | 72.81682 | -0.00761 | -0.70084 | 5 |  |  |
| -0.70145 | 73.88494 | -0.00758 | -0.69977 | 6 |  |  |
| -0.70038 | 74.95305 | -0.00755 | -0.69873 | 7 |  |  |
| -0.69931 | 76.02117 | -0.00753 | -0.69769 | 8 |  |  |
| -0.69824 | 77.08928 | -0.0075 | -0.69662 | 9 |  |  |
| -0.69717 | 78.1574 | -0.00748 | -0.6955 | 10 |  |  |
| -0.69611 | 79.22551 | -0.00746 | -0.69449 | 11 |  |  |
| -0.69504 | 80.29363 | -0.00743 | -0.69342 | 12 |  |  |
| -0.69397 | 81.36174 | -0.00741 | -0.69229 | 13 |  |  |
| -0.6929 | 82.42986 | -0.00739 | -0.69128 | 14 |  |  |
| -0.69183 | 83.49797 | -0.00737 | -0.69022 | 15 |  |  |
| -0.69077 | 84.56609 | -0.00735 | -0.68912 | 16 |  |  |
| -0.6897 | 85.6342 | -0.00733 | -0.68805 | 17 |  |  |
| -0.68863 | 86.70232 | -0.00731 | -0.68701 | 18 |  |  |
| -0.68756 | 87.77043 | -0.00729 | -0.68597 | 19 |  |  |
| -0.68649 | 88.83855 | -0.00727 | -0.68491 | 20 |  |  |
| -0.68542 | 89.90666 | -0.00725 | -0.68387 | 21 |  |  |
| -0.68436 | 90.97478 | -0.00723 | -0.6828 | 22 |  |  |
| -0.68329 | 92.04289 | -0.00721 | -0.68173 | 23 |  |  |
| -0.68222 | 93.11101 | -0.0072 | -0.68054 | 24 |  |  |
| -0.68115 | 94.17912 | -0.00718 | -0.67957 | 25 |  |  |
| -0.68008 | 95.24724 | -0.00716 | -0.67859 | 26 |  |  |
| -0.67902 | 96.31535 | -0.00714 | -0.67749 | 27 |  |  |
| -0.67795 | 97.38347 | -0.00712 | -0.67639 | 28 |  |  |
| -0.67688 | 98.45158 | -0.0071 | -0.67523 | 29 |  |  |
| -0.67581 | 99.5197 | -0.00709 | -0.67435 | 30 |  |  |
| -0.67474 | 100.5878 | -0.00707 | -0.67322 | 31 |  |  |
| -0.67368 | 101.6559 | -0.00705 | -0.67215 | 32 |  |  |
| -0.67261 | 102.724 | -0.00703 | -0.67102 | 33 |  |  |
| -0.67154 | 103.7922 | -0.00701 | -0.67001 | 34 |  |  |
| -0.67047 | 104.8603 | -0.00699 | -0.66898 | 35 |  |  |
| -0.6694 | 105.9284 | -0.00698 | -0.66794 | 36 |  |  |
| -0.66833 | 106.9965 | -0.00696 | -0.66687 | 37 |  |  |
| -0.66727 | 108.0646 | -0.00694 | -0.66586 | 38 |  |  |
| -0.6662 | 109.1327 | -0.00692 | -0.66467 | 39 |  |  |
| -0.66513 | 110.2008 | -0.0069 | -0.6637 | 40 |  |  |
| -0.66406 | 111.269 | -0.00688 | -0.66257 | 41 |  |  |
| -0.66299 | 112.3371 | -0.00687 | -0.6615 | 42 |  |  |
| -0.66193 | 113.4052 | -0.00685 | -0.66055 | 43 |  |  |
| -0.66086 | 114.4733 | -0.00683 | -0.65936 | 44 |  |  |
| -0.65979 | 115.5414 | -0.00681 | -0.65839 | 45 |  |  |
| -0.65872 | 116.6095 | -0.00679 | -0.65723 | 46 |  |  |
| -0.65765 | 117.6777 | -0.00677 | -0.65634 | 47 |  |  |
| -0.65659 | 118.7458 | -0.00676 | -0.65518 | 48 |  |  |
| -0.65552 | 119.8139 | -0.00674 | -0.65414 | 49 |  |  |
| -0.65445 | 120.882 | -0.00672 | -0.65302 | 50 |  |  |
| -0.65338 | 121.9501 | -0.0067 | -0.65198 | 51 |  |  |
| -0.65231 | 123.0182 | -0.00668 | -0.65094 | 52 |  |  |
| -0.65125 | 124.0863 | -0.00666 | -0.64984 | 53 |  |  |
| -0.65018 | 125.1545 | -0.00665 | -0.64883 | 54 |  |  |
| -0.64911 | 126.2226 | -0.00663 | -0.64764 | 55 |  |  |
| -0.64804 | 127.2907 | -0.00661 | -0.64664 | 56 |  |  |
| -0.64697 | 128.3588 | -0.00659 | -0.64563 | 57 |  |  |
| -0.6459 | 129.4269 | -0.00657 | -0.64444 | 58 |  |  |
| -0.64484 | 130.495 | -0.00655 | -0.64346 | 59 |  |  |
| -0.64377 | 131.5631 | -0.00654 | -0.64252 | 60 |  |  |
| -0.6427 | 132.6313 | -0.00652 | -0.64139 | 61 |  |  |
| -0.64163 | 133.6994 | -0.0065 | -0.64029 | 62 |  |  |
| -0.64056 | 134.7675 | -0.00648 | -0.63919 | 63 |  |  |
| -0.6395 | 135.8356 | -0.00646 | -0.63809 | 64 |  |  |
| -0.63843 | 136.9037 | -0.00644 | -0.63712 | 65 |  |  |
| -0.63736 | 137.9718 | -0.00642 | -0.63611 | 66 |  |  |
| -0.63629 | 139.04 | -0.0064 | -0.63495 | 67 |  |  |
| -0.63522 | 140.1081 | -0.00639 | -0.63391 | 68 |  |  |
| -0.63416 | 141.1762 | -0.00637 | -0.63293 | 69 |  |  |
| -0.63309 | 142.2443 | -0.00635 | -0.63187 | 70 |  |  |
| -0.63202 | 143.3124 | -0.00633 | -0.6308 | 71 |  |  |
| -0.63095 | 144.3805 | -0.00631 | -0.62967 | 72 |  |  |
| -0.62988 | 145.4486 | -0.00629 | -0.62857 | 73 |  |  |
| -0.62881 | 146.5168 | -0.00627 | -0.6275 | 74 |  |  |
| -0.62775 | 147.5849 | -0.00625 | -0.62656 | 75 |  |  |
| -0.62668 | 148.653 | -0.00623 | -0.62537 | 76 |  |  |
| -0.62561 | 149.7211 | -0.00621 | -0.62436 | 77 |  |  |
| -0.62454 | 150.7892 | -0.00619 | -0.62332 | 78 |  |  |
| -0.62347 | 151.8573 | -0.00618 | -0.62225 | 79 |  |  |
| -0.62241 | 152.9254 | -0.00616 | -0.62119 | 80 |  |  |
| -0.62134 | 153.9936 | -0.00614 | -0.62015 | 81 |  |  |
| -0.62027 | 155.0617 | -0.00612 | -0.61902 | 82 |  |  |
| -0.6192 | 156.1298 | -0.0061 | -0.61795 | 83 |  |  |
| -0.61813 | 157.1979 | -0.00608 | -0.61691 | 84 |  |  |
| -0.61707 | 158.266 | -0.00606 | -0.61584 | 85 |  |  |
| -0.616 | 159.3341 | -0.00604 | -0.61472 | 86 |  |  |
| -0.61493 | 160.4023 | -0.00602 | -0.61374 | 87 |  |  |
| -0.61386 | 161.4704 | -0.006 | -0.61264 | 88 |  |  |
| -0.61279 | 162.5385 | -0.00598 | -0.61157 | 89 |  |  |
| -0.61172 | 163.6066 | -0.00596 | -0.61053 | 90 |  |  |
| -0.61066 | 164.6747 | -0.00594 | -0.60959 | 91 |  |  |
| -0.60959 | 165.7428 | -0.00592 | -0.60834 | 92 |  |  |
| -0.60852 | 166.8109 | -0.00589 | -0.60736 | 93 |  |  |
| -0.60745 | 167.8791 | -0.00587 | -0.60626 | 94 |  |  |
| -0.60638 | 168.9472 | -0.00585 | -0.60526 | 95 |  |  |
| -0.60532 | 170.0153 | -0.00583 | -0.60416 | 96 |  |  |
| -0.60425 | 171.0834 | -0.00581 | -0.60312 | 97 |  |  |
| -0.60318 | 172.1515 | -0.00579 | -0.60211 | 98 |  |  |
| -0.60211 | 173.2196 | -0.00577 | -0.60101 | 99 |  |  |
| -0.60104 | 174.2877 | -0.00575 | -0.59988 | 100 |  |  |
| -0.59998 | 175.3559 | -0.00572 | -0.59885 | 101 |  |  |
| -0.59891 | 176.424 | -0.0057 | -0.59775 | 102 |  |  |
| -0.59784 | 177.4921 | -0.00568 | -0.59665 | 103 |  |  |
| -0.59677 | 178.5602 | -0.00566 | -0.59573 | 104 |  |  |
| -0.5957 | 179.6283 | -0.00563 | -0.5946 | 105 |  |  |
| -0.59464 | 180.6964 | -0.00561 | -0.59354 | 106 |  |  |
| -0.59357 | 181.7646 | -0.00559 | -0.59244 | 107 |  |  |
| -0.5925 | 182.8327 | -0.00557 | -0.59146 | 108 |  |  |
| -0.59143 | 183.9008 | -0.00554 | -0.59036 | 109 |  |  |
| -0.59036 | 184.9689 | -0.00552 | -0.58929 | 110 |  |  |
| -0.58929 | 186.037 | -0.00549 | -0.5881 | 111 |  |  |
| -0.58823 | 187.1051 | -0.00547 | -0.58713 | 112 |  |  |
| -0.58716 | 188.1732 | -0.00544 | -0.58606 | 113 |  |  |
| -0.58609 | 189.2414 | -0.00542 | -0.58496 | 114 |  |  |
| -0.58502 | 190.3095 | -0.00539 | -0.58395 | 115 |  |  |
| -0.58395 | 191.3776 | -0.00536 | -0.58289 | 116 |  |  |
| -0.58289 | 192.4457 | -0.00534 | -0.58179 | 117 |  |  |
| -0.58182 | 193.5138 | -0.00531 | -0.58078 | 118 |  |  |
| -0.58075 | 194.5819 | -0.00528 | -0.57977 | 119 |  |  |
| -0.57968 | 195.65 | -0.00525 | -0.5787 | 120 |  |  |
| -0.57861 | 196.7182 | -0.00523 | -0.57764 | 121 |  |  |
| -0.57755 | 197.7863 | -0.0052 | -0.57651 | 122 |  |  |
| -0.57648 | 198.8544 | -0.00517 | -0.57541 | 123 |  |  |
| -0.57541 | 199.9225 | -0.00513 | -0.5744 | 124 |  |  |
| -0.57434 | 200.9906 | -0.0051 | -0.57333 | 125 |  |  |
| -0.57327 | 202.0587 | -0.00507 | -0.5723 | 126 |  |  |
| -0.5722 | 203.1269 | -0.00504 | -0.57123 | 127 |  |  |
| -0.57114 | 204.195 | -0.005 | -0.57016 | 128 |  |  |
| -0.57007 | 205.2631 | -0.00496 | -0.56915 | 129 |  |  |
| -0.569 | 206.3312 | -0.00493 | -0.56799 | 130 |  |  |
| -0.56793 | 207.3993 | -0.00489 | -0.56699 | 131 |  |  |
| -0.56686 | 208.4674 | -0.00485 | -0.56601 | 132 |  |  |
| -0.5658 | 209.5355 | -0.00481 | -0.56491 | 133 |  |  |
| -0.56473 | 210.6037 | -0.00476 | -0.56381 | 134 |  |  |
| -0.56366 | 211.6718 | -0.00472 | -0.56271 | 135 |  |  |
| -0.56259 | 212.7399 | -0.00467 | -0.56168 | 136 |  |  |
| -0.56152 | 213.808 | -0.00462 | -0.56067 | 137 |  |  |
| -0.56046 | 214.8761 | -0.00457 | -0.55963 | 138 |  |  |
| -0.55939 | 215.9442 | -0.00451 | -0.5585 | 139 |  |  |
| -0.55832 | 217.0123 | -0.00445 | -0.55746 | 140 |  |  |
| -0.55725 | 218.0805 | -0.00439 | -0.55643 | 141 |  |  |
| -0.55618 | 219.1486 | -0.00432 | -0.55542 | 142 |  |  |
| -0.55511 | 220.2167 | -0.00425 | -0.55432 | 143 |  |  |
| -0.55405 | 221.2848 | -0.00418 | -0.55325 | 144 |  |  |
| -0.55298 | 222.3529 | -0.0041 | -0.55225 | 145 |  |  |
| -0.55191 | 223.421 | -0.00402 | -0.55121 | 146 |  |  |
| -0.55084 | 224.4892 | -0.00394 | -0.55011 | 147 |  |  |
| -0.54977 | 225.5573 | -0.00386 | -0.54907 | 148 |  |  |
| -0.54871 | 226.6254 | -0.00377 | -0.5481 | 149 |  |  |
| -0.54764 | 227.6935 | -0.00369 | -0.54703 | 150 |  |  |
| -0.54657 | 228.7616 | -0.0036 | -0.54602 | 151 |  |  |
| -0.5455 | 229.8297 | -0.00351 | -0.54492 | 152 |  |  |
| -0.54443 | 230.8978 | -0.00343 | -0.54391 | 153 |  |  |
| -0.54337 | 231.966 | -0.00335 | -0.54279 | 154 |  |  |
| -0.5423 | 233.0341 | -0.00326 | -0.54172 | 155 |  |  |
| -0.54123 | 234.1022 | -0.00318 | -0.54071 | 156 |  |  |
| -0.54016 | 235.1703 | -0.0031 | -0.53964 | 157 |  |  |
| -0.53909 | 236.2384 | -0.00302 | -0.53857 | 158 |  |  |
| -0.53802 | 237.3065 | -0.00295 | -0.53751 | 159 |  |  |
| -0.53696 | 238.3746 | -0.00287 | -0.53653 | 160 |  |  |
| -0.53589 | 239.4428 | -0.0028 | -0.5354 | 161 |  |  |
| -0.53482 | 240.5109 | -0.00273 | -0.53439 | 162 |  |  |
| -0.53375 | 241.579 | -0.00266 | -0.53333 | 163 |  |  |
| -0.53268 | 242.6471 | -0.00259 | -0.53226 | 164 |  |  |
| -0.53162 | 243.7152 | -0.00253 | -0.53125 | 165 |  |  |
| -0.53055 | 244.7833 | -0.00246 | -0.53024 | 166 |  |  |
| -0.52948 | 245.8515 | -0.0024 | -0.52917 | 167 |  |  |
| -0.52841 | 246.9196 | -0.00234 | -0.52814 | 168 |  |  |
| -0.52734 | 247.9877 | -0.00228 | -0.52701 | 169 |  |  |
| -0.52628 | 249.0558 | -0.00222 | -0.52588 | 170 |  |  |
| -0.52521 | 250.1239 | -0.00216 | -0.52487 | 171 |  |  |
| -0.52414 | 251.192 | -0.0021 | -0.52383 | 172 |  |  |
| -0.52307 | 252.2601 | -0.00205 | -0.52277 | 173 |  |  |
| -0.522 | 253.3283 | -0.002 | -0.5217 | 174 |  |  |
| -0.52094 | 254.3964 | -0.00194 | -0.52072 | 175 |  |  |
| -0.51987 | 255.4645 | -0.00189 | -0.51968 | 176 |  |  |
| -0.5188 | 256.5326 | -0.00184 | -0.51865 | 177 |  |  |
| -0.51773 | 257.6007 | -0.0018 | -0.51761 | 178 |  |  |
| -0.51666 | 258.6688 | -0.00175 | -0.51648 | 179 |  |  |
| -0.51559 | 259.7369 | -0.0017 | -0.51547 | 180 |  |  |
| -0.51453 | 260.8051 | -0.00166 | -0.51434 | 181 |  |  |
| -0.51346 | 261.8732 | -0.00161 | -0.51331 | 182 |  |  |
| -0.51239 | 262.9413 | -0.00157 | -0.51224 | 183 |  |  |
| -0.51132 | 264.0094 | -0.00153 | -0.51117 | 184 |  |  |
| -0.51025 | 265.0775 | -0.00149 | -0.5101 | 185 |  |  |
| -0.50919 | 266.1456 | -0.00145 | -0.50906 | 186 |  |  |
| -0.50812 | 267.2138 | -0.00141 | -0.50803 | 187 |  |  |
| -0.50705 | 268.2819 | -0.00137 | -0.50699 | 188 |  |  |
| -0.50598 | 269.35 | -0.00133 | -0.50583 | 189 |  |  |
| -0.50491 | 270.4181 | -0.0013 | -0.50479 | 190 |  |  |
| -0.50385 | 271.4862 | -0.00126 | -0.50375 | 191 |  |  |
| -0.50278 | 272.5543 | -0.00123 | -0.50262 | 192 |  |  |
| -0.50171 | 273.6224 | -0.00119 | -0.50156 | 193 |  |  |
| -0.50064 | 274.6906 | -0.00116 | -0.50043 | 194 |  |  |
| -0.49957 | 275.7587 | -0.00113 | -0.49942 | 195 |  |  |
| -0.4985 | 276.8268 | -0.0011 | -0.4985 | 196 |  |  |
| -0.49744 | 277.8949 | -0.00106 | -0.49741 | 197 |  |  |
| -0.49637 | 278.963 | -0.00103 | -0.49634 | 198 |  |  |
| -0.4953 | 280.0311 | -0.001 | -0.49527 | 199 |  |  |
| -0.49423 | 281.0992 | -0.00098 | -0.49423 | 200 |  |  |
| -0.49316 | 282.1674 | -0.00095 | -0.49313 | 201 |  |  |
| -0.4921 | 283.2355 | -0.00092 | -0.49213 | 202 |  |  |
| -0.49103 | 284.3036 | -0.00089 | -0.49103 | 203 |  |  |
| -0.48996 | 285.3717 | -0.00086 | -0.48999 | 204 |  |  |
| -0.48889 | 286.4398 | -0.00084 | -0.48889 | 205 |  |  |
| -0.48782 | 287.5079 | -0.00081 | -0.48773 | 206 |  |  |
| -0.48676 | 288.5761 | -0.00079 | -0.48672 | 207 |  |  |
| -0.48569 | 289.6442 | -0.00076 | -0.48569 | 208 |  |  |
| -0.48462 | 290.7123 | -0.00074 | -0.48471 | 209 |  |  |
| -0.48355 | 291.7804 | -0.00072 | -0.48355 | 210 |  |  |
| -0.48248 | 292.8485 | -0.00069 | -0.48248 | 211 |  |  |
| -0.48141 | 293.9166 | -0.00067 | -0.48148 | 212 |  |  |
| -0.48035 | 294.9847 | -0.00065 | -0.48038 | 213 |  |  |
| -0.47928 | 296.0529 | -0.00063 | -0.47931 | 214 |  |  |
| -0.47821 | 297.121 | -0.00061 | -0.47827 | 215 |  |  |
| -0.47714 | 298.1891 | -0.00058 | -0.47714 | 216 |  |  |
| -0.47607 | 299.2572 | -0.00056 | -0.47614 | 217 |  |  |
| -0.47501 | 300.3253 | -0.00054 | -0.47507 | 218 |  |  |
| -0.47394 | 301.3934 | -0.00052 | -0.47394 | 219 |  |  |
| -0.47287 | 302.4615 | -0.0005 | -0.47296 | 220 |  |  |
| -0.4718 | 303.5297 | -0.00048 | -0.47189 | 221 |  |  |
| -0.47073 | 304.5978 | -0.00046 | -0.47076 | 222 |  |  |
| -0.46967 | 305.6659 | -0.00045 | -0.46973 | 223 |  |  |
| -0.4686 | 306.734 | -0.00043 | -0.46857 | 224 |  |  |
| -0.46753 | 307.8021 | -0.00041 | -0.46756 | 225 |  |  |
| -0.46646 | 308.8702 | -0.00039 | -0.46652 | 226 |  |  |
| -0.46539 | 309.9384 | -0.00037 | -0.46533 | 227 |  |  |
| -0.46432 | 311.0065 | -0.00036 | -0.46439 | 228 |  |  |
| -0.46326 | 312.0746 | -0.00034 | -0.46332 | 229 |  |  |
| -0.46219 | 313.1427 | -0.00032 | -0.46219 | 230 |  |  |
| -0.46112 | 314.2108 | -0.00031 | -0.46115 | 231 |  |  |
| -0.46005 | 315.2789 | -0.00029 | -0.46005 | 232 |  |  |
| -0.45898 | 316.347 | -0.00027 | -0.45911 | 233 |  |  |
| -0.45792 | 317.4152 | -0.00026 | -0.45795 | 234 |  |  |
| -0.45685 | 318.4833 | -0.00024 | -0.45691 | 235 |  |  |
| -0.45578 | 319.5514 | -0.00022 | -0.45578 | 236 |  |  |
| -0.45471 | 320.6195 | -0.00021 | -0.45474 | 237 |  |  |
| -0.45364 | 321.6876 | -0.00019 | -0.4537 | 238 |  |  |
| -0.45258 | 322.7557 | -0.00018 | -0.45267 | 239 |  |  |
| -0.45151 | 323.8238 | -0.00016 | -0.45154 | 240 |  |  |
| -0.45044 | 324.892 | -0.00014 | -0.45047 | 241 |  |  |
| -0.44937 | 325.9601 | -0.00013 | -0.4494 | 242 |  |  |
| -0.4483 | 327.0282 | -0.00011 | -0.44833 | 243 |  |  |
| -0.44724 | 328.0963 | -9.7E-05 | -0.44727 | 244 |  |  |
| -0.44617 | 329.1644 | -8.1E-05 | -0.4462 | 245 |  |  |
| -0.4451 | 330.2325 | -6.6E-05 | -0.44519 | 246 |  |  |
| -0.44403 | 331.3007 | -5E-05 | -0.44409 | 247 |  |  |
| -0.44296 | 332.3688 | -3.4E-05 | -0.44305 | 248 |  |  |
| -0.44189 | 333.4369 | -1.7E-05 | -0.4418 | 249 |  |  |
| -0.44083 | 334.505 | -2E-06 | -0.44086 | 250 |  |  |
| -0.43976 | 335.5731 | 1.37E-05 | -0.44003 | 251 |  |  |
| -0.43869 | 336.6412 | 2.89E-05 | -0.43878 | 252 |  |  |
| -0.43762 | 337.7093 | 4.64E-05 | -0.43781 | 253 |  |  |
| -0.43655 | 338.7775 | 6.36E-05 | -0.43671 | 254 |  |  |
| -0.43549 | 339.8456 | 8.04E-05 | -0.43573 | 255 |  |  |
| -0.43442 | 340.9137 | 9.77E-05 | -0.43472 | 256 |  |  |
| -0.43335 | 341.9818 | 0.000115 | -0.43369 | 257 |  |  |
| -0.43228 | 343.0499 | 0.000133 | -0.43262 | 258 |  |  |
| -0.43121 | 344.118 | 0.000151 | -0.43149 | 259 |  |  |
| -0.43015 | 345.1861 | 0.000169 | -0.43048 | 260 |  |  |
| -0.42908 | 346.2543 | 0.000188 | -0.42935 | 261 |  |  |
| -0.42801 | 347.3224 | 0.000207 | -0.42828 | 262 |  |  |
| -0.42694 | 348.3905 | 0.000226 | -0.42737 | 263 |  |  |
| -0.42587 | 349.4586 | 0.000246 | -0.4263 | 264 |  |  |
| -0.4248 | 350.5267 | 0.000266 | -0.42526 | 265 |  |  |
| -0.42374 | 351.5948 | 0.000288 | -0.42416 | 266 |  |  |
| -0.42267 | 352.663 | 0.000309 | -0.42316 | 267 |  |  |
| -0.4216 | 353.7311 | 0.000331 | -0.42181 | 268 |  |  |
| -0.42053 | 354.7992 | 0.000354 | -0.42065 | 269 |  |  |
| -0.41946 | 355.8673 | 0.000377 | -0.41962 | 270 |  |  |
| -0.4184 | 356.9354 | 0.000401 | -0.41849 | 271 |  |  |
| -0.41733 | 358.0035 | 0.000426 | -0.41751 | 272 |  |  |
| -0.41626 | 359.0716 | 0.000452 | -0.41638 | 273 |  |  |
| -0.41519 | 360.1398 | 0.000478 | -0.41534 | 274 |  |  |
| -0.41412 | 361.2079 | 0.000505 | -0.41418 | 275 |  |  |
| -0.41306 | 362.276 | 0.000534 | -0.41324 | 276 |  |  |
| -0.41199 | 363.3441 | 0.000563 | -0.41211 | 277 |  |  |
| -0.41092 | 364.4122 | 0.000594 | -0.41113 | 278 |  |  |
| -0.40985 | 365.4803 | 0.000625 | -0.40994 | 279 |  |  |
| -0.40878 | 366.5484 | 0.000657 | -0.40897 | 280 |  |  |
| -0.40771 | 367.6166 | 0.000691 | -0.40793 | 281 |  |  |
| -0.40665 | 368.6847 | 0.000726 | -0.40692 | 282 |  |  |
| -0.40558 | 369.7528 | 0.000762 | -0.40573 | 283 |  |  |
| -0.40451 | 370.8209 | 0.0008 | -0.40472 | 284 |  |  |
| -0.40344 | 371.889 | 0.000839 | -0.40366 | 285 |  |  |
| -0.40237 | 372.9571 | 0.00088 | -0.40259 | 286 |  |  |
| -0.40131 | 374.0253 | 0.000923 | -0.40158 | 287 |  |  |
| -0.40024 | 375.0934 | 0.000967 | -0.40051 | 288 |  |  |
| -0.39917 | 376.1615 | 0.001013 | -0.39948 | 289 |  |  |
| -0.3981 | 377.2296 | 0.00106 | -0.39838 | 290 |  |  |
| -0.39703 | 378.2977 | 0.001111 | -0.39737 | 291 |  |  |
| -0.39597 | 379.3658 | 0.001163 | -0.39636 | 292 |  |  |
| -0.3949 | 380.4339 | 0.001218 | -0.39517 | 293 |  |  |
| -0.39383 | 381.5021 | 0.001275 | -0.39417 | 294 |  |  |
| -0.39276 | 382.5702 | 0.001335 | -0.39294 | 295 |  |  |
| -0.39169 | 383.6383 | 0.001397 | -0.39203 | 296 |  |  |
| -0.39063 | 384.7064 | 0.001462 | -0.3909 | 297 |  |  |
| -0.38956 | 385.7745 | 0.00153 | -0.38989 | 298 |  |  |
| -0.38849 | 386.8426 | 0.001602 | -0.38876 | 299 |  |  |
| -0.38742 | 387.9107 | 0.001677 | -0.38766 | 300 |  |  |
| -0.38635 | 388.9789 | 0.001755 | -0.38666 | 301 |  |  |
| -0.38528 | 390.047 | 0.001837 | -0.38568 | 302 |  |  |
| -0.38422 | 391.1151 | 0.001924 | -0.38449 | 303 |  |  |
| -0.38315 | 392.1832 | 0.002014 | -0.38345 | 304 |  |  |
| -0.38208 | 393.2513 | 0.002109 | -0.38242 | 305 |  |  |
| -0.38101 | 394.3194 | 0.002208 | -0.38135 | 306 |  |  |
| -0.37994 | 395.3876 | 0.002313 | -0.38028 | 307 |  |  |
| -0.37888 | 396.4557 | 0.002423 | -0.37933 | 308 |  |  |
| -0.37781 | 397.5238 | 0.002538 | -0.3782 | 309 |  |  |
| -0.37674 | 398.5919 | 0.002658 | -0.3772 | 310 |  |  |
| -0.37567 | 399.66 | 0.002785 | -0.37613 | 311 |  |  |
| -0.3746 | 400.7281 | 0.00292 | -0.375 | 312 |  |  |
| -0.37354 | 401.7962 | 0.003059 | -0.37405 | 313 |  |  |
| -0.37247 | 402.8644 | 0.003205 | -0.37308 | 314 |  |  |
| -0.3714 | 403.9325 | 0.003357 | -0.37201 | 315 |  |  |
| -0.37033 | 405.0006 | 0.003513 | -0.37088 | 316 |  |  |
| -0.36926 | 406.0687 | 0.003672 | -0.36981 | 317 |  |  |
| -0.36819 | 407.1368 | 0.003833 | -0.3689 | 318 |  |  |
| -0.36713 | 408.2049 | 0.00399 | -0.36783 | 319 |  |  |
| -0.36606 | 409.273 | 0.00414 | -0.36676 | 320 |  |  |
| -0.36499 | 410.3412 | 0.004277 | -0.36584 | 321 |  |  |
| -0.36392 | 411.4093 | 0.004402 | -0.36475 | 322 |  |  |
| -0.36285 | 412.4774 | 0.004516 | -0.36365 | 323 |  |  |
| -0.36179 | 413.5455 | 0.00462 | -0.36261 | 324 |  |  |
| -0.36072 | 414.6136 | 0.004714 | -0.36154 | 325 |  |  |
| -0.35965 | 415.6817 | 0.0048 | -0.3606 | 326 |  |  |
| -0.35858 | 416.7499 | 0.004879 | -0.35947 | 327 |  |  |
| -0.35751 | 417.818 | 0.004952 | -0.35843 | 328 |  |  |
| -0.35645 | 418.8861 | 0.00502 | -0.35739 | 329 |  |  |
| -0.35538 | 419.9542 | 0.005085 | -0.35632 | 330 |  |  |
| -0.35431 | 421.0223 | 0.005147 | -0.35529 | 331 |  |  |
| -0.35324 | 422.0904 | 0.005207 | -0.35422 | 332 |  |  |
| -0.35217 | 423.1585 | 0.005264 | -0.35312 | 333 |  |  |
| -0.3511 | 424.2267 | 0.005319 | -0.35217 | 334 |  |  |
| -0.35004 | 425.2948 | 0.005372 | -0.35107 | 335 |  |  |
| -0.34897 | 426.3629 | 0.005424 | -0.35007 | 336 |  |  |
| -0.3479 | 427.431 | 0.005474 | -0.349 | 337 |  |  |
| -0.34683 | 428.4991 | 0.005523 | -0.34787 | 338 |  |  |
| -0.34576 | 429.5672 | 0.005571 | -0.34692 | 339 |  |  |
| -0.3447 | 430.6353 | 0.005618 | -0.34579 | 340 |  |  |
| -0.34363 | 431.7035 | 0.005665 | -0.34473 | 341 |  |  |
| -0.34256 | 432.7716 | 0.00571 | -0.34369 | 342 |  |  |
| -0.34149 | 433.8397 | 0.005755 | -0.34268 | 343 |  |  |
| -0.34042 | 434.9078 | 0.0058 | -0.34155 | 344 |  |  |
| -0.33936 | 435.9759 | 0.005844 | -0.34055 | 345 |  |  |
| -0.33829 | 437.044 | 0.005888 | -0.33945 | 346 |  |  |
| -0.33722 | 438.1122 | 0.005932 | -0.33847 | 347 |  |  |
| -0.33615 | 439.1803 | 0.005975 | -0.33731 | 348 |  |  |
| -0.33508 | 440.2484 | 0.006018 | -0.33627 | 349 |  |  |
| -0.33401 | 441.3165 | 0.006062 | -0.33517 | 350 |  |  |
| -0.33295 | 442.3846 | 0.006104 | -0.33414 | 351 |  |  |
| -0.33188 | 443.4527 | 0.006147 | -0.33316 | 352 |  |  |
| -0.33081 | 444.5208 | 0.00619 | -0.33206 | 353 |  |  |
| -0.32974 | 445.589 | 0.006234 | -0.33093 | 354 |  |  |
| -0.32867 | 446.6571 | 0.006277 | -0.32993 | 355 |  |  |
| -0.32761 | 447.7252 | 0.006319 | -0.32886 | 356 |  |  |
| -0.32654 | 448.7933 | 0.006362 | -0.32785 | 357 |  |  |
| -0.32547 | 449.8614 | 0.006405 | -0.32681 | 358 |  |  |
| -0.3244 | 450.9295 | 0.006448 | -0.32571 | 359 |  |  |
| -0.32333 | 451.9976 | 0.00649 | -0.32458 | 360 |  |  |
| -0.32227 | 453.0658 | 0.006533 | -0.32355 | 361 |  |  |
| -0.3212 | 454.1339 | 0.006575 | -0.3226 | 362 |  |  |
| -0.32013 | 455.202 | 0.006617 | -0.3215 | 363 |  |  |
| -0.31906 | 456.2701 | 0.006659 | -0.3204 | 364 |  |  |
| -0.31799 | 457.3382 | 0.0067 | -0.31934 | 365 |  |  |
| -0.31693 | 458.4063 | 0.00674 | -0.31824 | 366 |  |  |
| -0.31586 | 459.4745 | 0.006781 | -0.3172 | 367 |  |  |
| -0.31479 | 460.5426 | 0.006821 | -0.31613 | 368 |  |  |
| -0.31372 | 461.6107 | 0.00686 | -0.31512 | 369 |  |  |
| -0.31265 | 462.6788 | 0.006898 | -0.31406 | 370 |  |  |
| -0.31158 | 463.7469 | 0.006937 | -0.31296 | 371 |  |  |
| -0.31052 | 464.815 | 0.006973 | -0.31207 | 372 |  |  |
| -0.30945 | 465.8831 | 0.007009 | -0.31088 | 373 |  |  |
| -0.30838 | 466.9513 | 0.007045 | -0.30975 | 374 |  |  |
| -0.30731 | 468.0194 | 0.007079 | -0.30878 | 375 |  |  |
| -0.30624 | 469.0875 | 0.007113 | -0.30774 | 376 |  |  |
| -0.30518 | 470.1556 | 0.007146 | -0.30658 | 377 |  |  |
| -0.30411 | 471.2237 | 0.007177 | -0.30557 | 378 |  |  |
| -0.30304 | 472.2918 | 0.007209 | -0.30447 | 379 |  |  |
| -0.30197 | 473.3599 | 0.007239 | -0.3035 | 380 |  |  |
| -0.3009 | 474.4281 | 0.007269 | -0.30246 | 381 |  |  |
| -0.29984 | 475.4962 | 0.007298 | -0.30124 | 382 |  |  |
| -0.29877 | 476.5643 | 0.007327 | -0.30023 | 383 |  |  |
| -0.2977 | 477.6324 | 0.007354 | -0.29919 | 384 |  |  |
| -0.29663 | 478.7005 | 0.007382 | -0.29816 | 385 |  |  |
| -0.29556 | 479.7686 | 0.007409 | -0.29709 | 386 |  |  |
| -0.29449 | 480.8368 | 0.007435 | -0.29605 | 387 |  |  |
| -0.29343 | 481.9049 | 0.007461 | -0.29504 | 388 |  |  |
| -0.29236 | 482.973 | 0.007487 | -0.29388 | 389 |  |  |
| -0.29129 | 484.0411 | 0.007512 | -0.29288 | 390 |  |  |
| -0.29022 | 485.1092 | 0.007536 | -0.29169 | 391 |  |  |
| -0.28915 | 486.1773 | 0.00756 | -0.29068 | 392 |  |  |
| -0.28809 | 487.2454 | 0.007584 | -0.2897 | 393 |  |  |
| -0.28702 | 488.3136 | 0.007607 | -0.28854 | 394 |  |  |
| -0.28595 | 489.3817 | 0.00763 | -0.28757 | 395 |  |  |
| -0.28488 | 490.4498 | 0.007653 | -0.28644 | 396 |  |  |
| -0.28381 | 491.5179 | 0.007675 | -0.28543 | 397 |  |  |
| -0.28275 | 492.586 | 0.007696 | -0.2843 | 398 |  |  |
| -0.28168 | 493.6541 | 0.007718 | -0.28336 | 399 |  |  |
| -0.28061 | 494.7222 | 0.007739 | -0.28223 | 400 |  |  |
| -0.27954 | 495.7904 | 0.007759 | -0.28125 | 401 |  |  |
| -0.27847 | 496.8585 | 0.00778 | -0.28009 | 402 |  |  |
| -0.2774 | 497.9266 | 0.0078 | -0.27899 | 403 |  |  |
| -0.27634 | 498.9947 | 0.00782 | -0.27789 | 404 |  |  |
| -0.27527 | 500.0628 | 0.00784 | -0.27682 | 405 |  |  |
| -0.2742 | 501.1309 | 0.007859 | -0.27585 | 406 |  |  |
| -0.27313 | 502.1991 | 0.007878 | -0.27478 | 407 |  |  |
| -0.27206 | 503.2672 | 0.007896 | -0.27377 | 408 |  |  |
| -0.271 | 504.3353 | 0.007914 | -0.27271 | 409 |  |  |
| -0.26993 | 505.4034 | 0.007931 | -0.27161 | 410 |  |  |
| -0.26886 | 506.4715 | 0.007948 | -0.27051 | 411 |  |  |
| -0.26779 | 507.5396 | 0.007965 | -0.26944 | 412 |  |  |
| -0.26672 | 508.6077 | 0.007982 | -0.2684 | 413 |  |  |
| -0.26566 | 509.6759 | 0.007999 | -0.26736 | 414 |  |  |
| -0.26459 | 510.744 | 0.008015 | -0.26627 | 415 |  |  |
| -0.26352 | 511.8121 | 0.00803 | -0.2652 | 416 |  |  |
| -0.26245 | 512.8802 | 0.008046 | -0.26413 | 417 |  |  |
| -0.26138 | 513.9483 | 0.008062 | -0.26318 | 418 |  |  |
| -0.26031 | 515.0164 | 0.008078 | -0.26205 | 419 |  |  |
| -0.25925 | 516.0845 | 0.008092 | -0.26096 | 420 |  |  |
| -0.25818 | 517.1527 | 0.008108 | -0.25983 | 421 |  |  |
| -0.25711 | 518.2208 | 0.008121 | -0.25891 | 422 |  |  |
| -0.25604 | 519.2889 | 0.008134 | -0.25781 | 423 |  |  |
| -0.25497 | 520.357 | 0.008148 | -0.25671 | 424 |  |  |
| -0.25391 | 521.4251 | 0.00816 | -0.25574 | 425 |  |  |
| -0.25284 | 522.4932 | 0.008174 | -0.25458 | 426 |  |  |
| -0.25177 | 523.5614 | 0.008186 | -0.25357 | 427 |  |  |
| -0.2507 | 524.6295 | 0.008199 | -0.25247 | 428 |  |  |
| -0.24963 | 525.6976 | 0.008212 | -0.25134 | 429 |  |  |
| -0.24857 | 526.7657 | 0.008223 | -0.25034 | 430 |  |  |
| -0.2475 | 527.8338 | 0.008233 | -0.2493 | 431 |  |  |
| -0.24643 | 528.9019 | 0.008244 | -0.2482 | 432 |  |  |
| -0.24536 | 529.97 | 0.008257 | -0.24716 | 433 |  |  |
| -0.24429 | 531.0382 | 0.008267 | -0.24603 | 434 |  |  |
| -0.24323 | 532.1063 | 0.00828 | -0.24493 | 435 |  |  |
| -0.24216 | 533.1744 | 0.008291 | -0.24399 | 436 |  |  |
| -0.24109 | 534.2425 | 0.0083 | -0.24283 | 437 |  |  |
| -0.24002 | 535.3106 | 0.008311 | -0.24176 | 438 |  |  |
| -0.23895 | 536.3787 | 0.008319 | -0.24066 | 439 |  |  |
| -0.23788 | 537.4468 | 0.008329 | -0.23965 | 440 |  |  |
| -0.23682 | 538.515 | 0.008338 | -0.23859 | 441 |  |  |
| -0.23575 | 539.5831 | 0.008346 | -0.23758 | 442 |  |  |
| -0.23468 | 540.6512 | 0.008355 | -0.23651 | 443 |  |  |
| -0.23361 | 541.7193 | 0.008366 | -0.23535 | 444 |  |  |
| -0.23254 | 542.7874 | 0.008375 | -0.23431 | 445 |  |  |
| -0.23148 | 543.8555 | 0.008383 | -0.23331 | 446 |  |  |
| -0.23041 | 544.9237 | 0.008391 | -0.23233 | 447 |  |  |
| -0.22934 | 545.9918 | 0.008401 | -0.23108 | 448 |  |  |
| -0.22827 | 547.0599 | 0.008409 | -0.23004 | 449 |  |  |
| -0.2272 | 548.128 | 0.008419 | -0.22903 | 450 |  |  |
| -0.22614 | 549.1961 | 0.008425 | -0.228 | 451 |  |  |
| -0.22507 | 550.2642 | 0.008433 | -0.22693 | 452 |  |  |
| -0.224 | 551.3323 | 0.008441 | -0.2258 | 453 |  |  |
| -0.22293 | 552.4005 | 0.008447 | -0.22476 | 454 |  |  |
| -0.22186 | 553.4686 | 0.008453 | -0.22369 | 455 |  |  |
| -0.22079 | 554.5367 | 0.008461 | -0.2226 | 456 |  |  |
| -0.21973 | 555.6048 | 0.008467 | -0.2215 | 457 |  |  |
| -0.21866 | 556.6729 | 0.008472 | -0.22052 | 458 |  |  |
| -0.21759 | 557.741 | 0.00848 | -0.21939 | 459 |  |  |
| -0.21652 | 558.8091 | 0.008486 | -0.21838 | 460 |  |  |
| -0.21545 | 559.8773 | 0.008495 | -0.21722 | 461 |  |  |
| -0.21439 | 560.9454 | 0.008501 | -0.21622 | 462 |  |  |
| -0.21332 | 562.0135 | 0.008507 | -0.21512 | 463 |  |  |
| -0.21225 | 563.0816 | 0.008513 | -0.21414 | 464 |  |  |
| -0.21118 | 564.1497 | 0.008517 | -0.21298 | 465 |  |  |
| -0.21011 | 565.2178 | 0.008522 | -0.21194 | 466 |  |  |
| -0.20905 | 566.286 | 0.008526 | -0.21088 | 467 |  |  |
| -0.20798 | 567.3541 | 0.008534 | -0.20987 | 468 |  |  |
| -0.20691 | 568.4222 | 0.00854 | -0.20868 | 469 |  |  |

Sample: *Yarrow*, Concentration (ppm): 200, Immersion time: 24h

| Potential applied(V) | Time (s) | WE(1).  Current (A) | WE(1).  Potential (V) | Index |  |  |
| --- | --- | --- | --- | --- | --- | --- |
| -0.66818 | 68.25635 | -9.3E-05 | -0.66626 | 1 |  |  |
| -0.66711 | 69.32447 | -0.00093 | -0.66507 | 2 |  |  |
| -0.66605 | 70.39258 | -0.00602 | -0.66495 | 3 |  |  |
| -0.66498 | 71.4607 | -0.00599 | -0.66379 | 4 |  |  |
| -0.66391 | 72.52881 | -0.00595 | -0.66278 | 5 |  |  |
| -0.66284 | 73.59693 | -0.00592 | -0.66168 | 6 |  |  |
| -0.66177 | 74.66504 | -0.00589 | -0.66064 | 7 |  |  |
| -0.66071 | 75.73316 | -0.00586 | -0.65952 | 8 |  |  |
| -0.65964 | 76.80127 | -0.00583 | -0.65851 | 9 |  |  |
| -0.65857 | 77.86939 | -0.0058 | -0.65741 | 10 |  |  |
| -0.6575 | 78.9375 | -0.00577 | -0.65634 | 11 |  |  |
| -0.65643 | 80.00562 | -0.00574 | -0.65536 | 12 |  |  |
| -0.65536 | 81.07373 | -0.00572 | -0.65433 | 13 |  |  |
| -0.6543 | 82.14185 | -0.00569 | -0.6532 | 14 |  |  |
| -0.65323 | 83.20996 | -0.00566 | -0.65213 | 15 |  |  |
| -0.65216 | 84.27808 | -0.00563 | -0.65112 | 16 |  |  |
| -0.65109 | 85.34619 | -0.0056 | -0.65009 | 17 |  |  |
| -0.65002 | 86.41431 | -0.00558 | -0.64902 | 18 |  |  |
| -0.64896 | 87.48242 | -0.00555 | -0.64795 | 19 |  |  |
| -0.64789 | 88.55054 | -0.00552 | -0.64682 | 20 |  |  |
| -0.64682 | 89.61865 | -0.00549 | -0.64578 | 21 |  |  |
| -0.64575 | 90.68677 | -0.00546 | -0.64471 | 22 |  |  |
| -0.64468 | 91.75488 | -0.00543 | -0.64374 | 23 |  |  |
| -0.64362 | 92.823 | -0.0054 | -0.64264 | 24 |  |  |
| -0.64255 | 93.89111 | -0.00537 | -0.6416 | 25 |  |  |
| -0.64148 | 94.95923 | -0.00534 | -0.64053 | 26 |  |  |
| -0.64041 | 96.02734 | -0.00531 | -0.63947 | 27 |  |  |
| -0.63934 | 97.09546 | -0.00528 | -0.63837 | 28 |  |  |
| -0.63828 | 98.16357 | -0.00525 | -0.63727 | 29 |  |  |
| -0.63721 | 99.23169 | -0.00522 | -0.63623 | 30 |  |  |
| -0.63614 | 100.2998 | -0.00518 | -0.63525 | 31 |  |  |
| -0.63507 | 101.3679 | -0.00515 | -0.63416 | 32 |  |  |
| -0.634 | 102.436 | -0.00511 | -0.63315 | 33 |  |  |
| -0.63293 | 103.5041 | -0.00508 | -0.63214 | 34 |  |  |
| -0.63187 | 104.5723 | -0.00504 | -0.63104 | 35 |  |  |
| -0.6308 | 105.6404 | -0.005 | -0.62988 | 36 |  |  |
| -0.62973 | 106.7085 | -0.00496 | -0.62894 | 37 |  |  |
| -0.62866 | 107.7766 | -0.00492 | -0.62799 | 38 |  |  |
| -0.62759 | 108.8447 | -0.00488 | -0.62677 | 39 |  |  |
| -0.62653 | 109.9128 | -0.00484 | -0.62564 | 40 |  |  |
| -0.62546 | 110.981 | -0.00479 | -0.62466 | 41 |  |  |
| -0.62439 | 112.0491 | -0.00474 | -0.62366 | 42 |  |  |
| -0.62332 | 113.1172 | -0.00469 | -0.62259 | 43 |  |  |
| -0.62225 | 114.1853 | -0.00464 | -0.62146 | 44 |  |  |
| -0.62119 | 115.2534 | -0.00459 | -0.62045 | 45 |  |  |
| -0.62012 | 116.3215 | -0.00453 | -0.61938 | 46 |  |  |
| -0.61905 | 117.3896 | -0.00447 | -0.61832 | 47 |  |  |
| -0.61798 | 118.4578 | -0.0044 | -0.61737 | 48 |  |  |
| -0.61691 | 119.5259 | -0.00434 | -0.61621 | 49 |  |  |
| -0.61584 | 120.594 | -0.00427 | -0.61514 | 50 |  |  |
| -0.61478 | 121.6621 | -0.00419 | -0.61411 | 51 |  |  |
| -0.61371 | 122.7302 | -0.00412 | -0.61304 | 52 |  |  |
| -0.61264 | 123.7983 | -0.00404 | -0.61197 | 53 |  |  |
| -0.61157 | 124.8664 | -0.00396 | -0.61096 | 54 |  |  |
| -0.6105 | 125.9346 | -0.00387 | -0.60992 | 55 |  |  |
| -0.60944 | 127.0027 | -0.00379 | -0.60889 | 56 |  |  |
| -0.60837 | 128.0708 | -0.00371 | -0.60779 | 57 |  |  |
| -0.6073 | 129.1389 | -0.00362 | -0.60678 | 58 |  |  |
| -0.60623 | 130.207 | -0.00354 | -0.60568 | 59 |  |  |
| -0.60516 | 131.2751 | -0.00346 | -0.60474 | 60 |  |  |
| -0.6041 | 132.3433 | -0.00338 | -0.60361 | 61 |  |  |
| -0.60303 | 133.4114 | -0.0033 | -0.60266 | 62 |  |  |
| -0.60196 | 134.4795 | -0.00323 | -0.60153 | 63 |  |  |
| -0.60089 | 135.5476 | -0.00315 | -0.60052 | 64 |  |  |
| -0.59982 | 136.6157 | -0.00308 | -0.5994 | 65 |  |  |
| -0.59875 | 137.6838 | -0.00301 | -0.59836 | 66 |  |  |
| -0.59769 | 138.7519 | -0.00294 | -0.59729 | 67 |  |  |
| -0.59662 | 139.8201 | -0.00287 | -0.59634 | 68 |  |  |
| -0.59555 | 140.8882 | -0.00281 | -0.59531 | 69 |  |  |
| -0.59448 | 141.9563 | -0.00274 | -0.59418 | 70 |  |  |
| -0.59341 | 143.0244 | -0.00268 | -0.59314 | 71 |  |  |
| -0.59235 | 144.0925 | -0.00262 | -0.59204 | 72 |  |  |
| -0.59128 | 145.1606 | -0.00256 | -0.59106 | 73 |  |  |
| -0.59021 | 146.2287 | -0.0025 | -0.5899 | 74 |  |  |
| -0.58914 | 147.2969 | -0.00245 | -0.58896 | 75 |  |  |
| -0.58807 | 148.365 | -0.00239 | -0.58789 | 76 |  |  |
| -0.58701 | 149.4331 | -0.00234 | -0.58679 | 77 |  |  |
| -0.58594 | 150.5012 | -0.00229 | -0.58569 | 78 |  |  |
| -0.58487 | 151.5693 | -0.00223 | -0.58463 | 79 |  |  |
| -0.5838 | 152.6374 | -0.00218 | -0.58362 | 80 |  |  |
| -0.58273 | 153.7056 | -0.00214 | -0.58252 | 81 |  |  |
| -0.58167 | 154.7737 | -0.00209 | -0.58151 | 82 |  |  |
| -0.5806 | 155.8418 | -0.00204 | -0.58035 | 83 |  |  |
| -0.57953 | 156.9099 | -0.002 | -0.57935 | 84 |  |  |
| -0.57846 | 157.978 | -0.00195 | -0.57837 | 85 |  |  |
| -0.57739 | 159.0461 | -0.00191 | -0.57727 | 86 |  |  |
| -0.57632 | 160.1142 | -0.00187 | -0.5762 | 87 |  |  |
| -0.57526 | 161.1824 | -0.00183 | -0.57513 | 88 |  |  |
| -0.57419 | 162.2505 | -0.00179 | -0.57407 | 89 |  |  |
| -0.57312 | 163.3186 | -0.00175 | -0.57306 | 90 |  |  |
| -0.57205 | 164.3867 | -0.00172 | -0.57205 | 91 |  |  |
| -0.57098 | 165.4548 | -0.00168 | -0.57092 | 92 |  |  |
| -0.56992 | 166.5229 | -0.00164 | -0.56989 | 93 |  |  |
| -0.56885 | 167.591 | -0.00161 | -0.56876 | 94 |  |  |
| -0.56778 | 168.6592 | -0.00158 | -0.56775 | 95 |  |  |
| -0.56671 | 169.7273 | -0.00154 | -0.56668 | 96 |  |  |
| -0.56564 | 170.7954 | -0.00151 | -0.56558 | 97 |  |  |
| -0.56458 | 171.8635 | -0.00148 | -0.56464 | 98 |  |  |
| -0.56351 | 172.9316 | -0.00145 | -0.56354 | 99 |  |  |
| -0.56244 | 173.9997 | -0.00142 | -0.56241 | 100 |  |  |
| -0.56137 | 175.0679 | -0.00139 | -0.56131 | 101 |  |  |
| -0.5603 | 176.136 | -0.00136 | -0.5603 | 102 |  |  |
| -0.55923 | 177.2041 | -0.00133 | -0.55917 | 103 |  |  |
| -0.55817 | 178.2722 | -0.0013 | -0.55811 | 104 |  |  |
| -0.5571 | 179.3403 | -0.00128 | -0.55707 | 105 |  |  |
| -0.55603 | 180.4084 | -0.00125 | -0.556 | 106 |  |  |
| -0.55496 | 181.4765 | -0.00123 | -0.55502 | 107 |  |  |
| -0.55389 | 182.5447 | -0.0012 | -0.55392 | 108 |  |  |
| -0.55283 | 183.6128 | -0.00118 | -0.55286 | 109 |  |  |
| -0.55176 | 184.6809 | -0.00115 | -0.55176 | 110 |  |  |
| -0.55069 | 185.749 | -0.00113 | -0.55078 | 111 |  |  |
| -0.54962 | 186.8171 | -0.00111 | -0.54968 | 112 |  |  |
| -0.54855 | 187.8852 | -0.00109 | -0.54855 | 113 |  |  |
| -0.54749 | 188.9533 | -0.00107 | -0.54745 | 114 |  |  |
| -0.54642 | 190.0215 | -0.00104 | -0.54645 | 115 |  |  |
| -0.54535 | 191.0896 | -0.00102 | -0.54538 | 116 |  |  |
| -0.54428 | 192.1577 | -0.001 | -0.54437 | 117 |  |  |
| -0.54321 | 193.2258 | -0.00098 | -0.54327 | 118 |  |  |
| -0.54214 | 194.2939 | -0.00096 | -0.54221 | 119 |  |  |
| -0.54108 | 195.362 | -0.00095 | -0.54117 | 120 |  |  |
| -0.54001 | 196.4302 | -0.00093 | -0.54007 | 121 |  |  |
| -0.53894 | 197.4983 | -0.00091 | -0.539 | 122 |  |  |
| -0.53787 | 198.5664 | -0.00089 | -0.53799 | 123 |  |  |
| -0.5368 | 199.6345 | -0.00088 | -0.53687 | 124 |  |  |
| -0.53574 | 200.7026 | -0.00086 | -0.53577 | 125 |  |  |
| -0.53467 | 201.7707 | -0.00084 | -0.53473 | 126 |  |  |
| -0.5336 | 202.8388 | -0.00083 | -0.53363 | 127 |  |  |
| -0.53253 | 203.907 | -0.00081 | -0.53259 | 128 |  |  |
| -0.53146 | 204.9751 | -0.00079 | -0.53159 | 129 |  |  |
| -0.5304 | 206.0432 | -0.00078 | -0.53036 | 130 |  |  |
| -0.52933 | 207.1113 | -0.00076 | -0.52936 | 131 |  |  |
| -0.52826 | 208.1794 | -0.00075 | -0.52841 | 132 |  |  |
| -0.52719 | 209.2475 | -0.00074 | -0.52722 | 133 |  |  |
| -0.52612 | 210.3156 | -0.00072 | -0.52625 | 134 |  |  |
| -0.52505 | 211.3838 | -0.00071 | -0.52512 | 135 |  |  |
| -0.52399 | 212.4519 | -0.0007 | -0.52414 | 136 |  |  |
| -0.52292 | 213.52 | -0.00068 | -0.52298 | 137 |  |  |
| -0.52185 | 214.5881 | -0.00067 | -0.52194 | 138 |  |  |
| -0.52078 | 215.6562 | -0.00066 | -0.52078 | 139 |  |  |
| -0.51971 | 216.7243 | -0.00064 | -0.51984 | 140 |  |  |
| -0.51865 | 217.7925 | -0.00063 | -0.51883 | 141 |  |  |
| -0.51758 | 218.8606 | -0.00062 | -0.51767 | 142 |  |  |
| -0.51651 | 219.9287 | -0.00061 | -0.51654 | 143 |  |  |
| -0.51544 | 220.9968 | -0.0006 | -0.51556 | 144 |  |  |
| -0.51437 | 222.0649 | -0.00059 | -0.5145 | 145 |  |  |
| -0.51331 | 223.133 | -0.00058 | -0.5134 | 146 |  |  |
| -0.51224 | 224.2011 | -0.00056 | -0.51227 | 147 |  |  |
| -0.51117 | 225.2693 | -0.00055 | -0.51123 | 148 |  |  |
| -0.5101 | 226.3374 | -0.00054 | -0.51022 | 149 |  |  |
| -0.50903 | 227.4055 | -0.00053 | -0.50916 | 150 |  |  |
| -0.50797 | 228.4736 | -0.00052 | -0.50803 | 151 |  |  |
| -0.5069 | 229.5417 | -0.00051 | -0.50705 | 152 |  |  |
| -0.50583 | 230.6098 | -0.0005 | -0.50598 | 153 |  |  |
| -0.50476 | 231.6779 | -0.00049 | -0.50488 | 154 |  |  |
| -0.50369 | 232.7461 | -0.00048 | -0.50385 | 155 |  |  |
| -0.50262 | 233.8142 | -0.00048 | -0.50272 | 156 |  |  |
| -0.50156 | 234.8823 | -0.00047 | -0.50153 | 157 |  |  |
| -0.50049 | 235.9504 | -0.00046 | -0.50052 | 158 |  |  |
| -0.49942 | 237.0185 | -0.00045 | -0.49954 | 159 |  |  |
| -0.49835 | 238.0866 | -0.00044 | -0.49844 | 160 |  |  |
| -0.49728 | 239.1548 | -0.00043 | -0.49738 | 161 |  |  |
| -0.49622 | 240.2229 | -0.00042 | -0.49637 | 162 |  |  |
| -0.49515 | 241.291 | -0.00042 | -0.49527 | 163 |  |  |
| -0.49408 | 242.3591 | -0.00041 | -0.49414 | 164 |  |  |
| -0.49301 | 243.4272 | -0.0004 | -0.4931 | 165 |  |  |
| -0.49194 | 244.4953 | -0.00039 | -0.49203 | 166 |  |  |
| -0.49088 | 245.5634 | -0.00038 | -0.49094 | 167 |  |  |
| -0.48981 | 246.6316 | -0.00038 | -0.48996 | 168 |  |  |
| -0.48874 | 247.6997 | -0.00037 | -0.48883 | 169 |  |  |
| -0.48767 | 248.7678 | -0.00036 | -0.48785 | 170 |  |  |
| -0.4866 | 249.8359 | -0.00035 | -0.48663 | 171 |  |  |
| -0.48553 | 250.904 | -0.00035 | -0.4856 | 172 |  |  |
| -0.48447 | 251.9721 | -0.00034 | -0.48459 | 173 |  |  |
| -0.4834 | 253.0402 | -0.00033 | -0.48358 | 174 |  |  |
| -0.48233 | 254.1084 | -0.00033 | -0.48251 | 175 |  |  |
| -0.48126 | 255.1765 | -0.00032 | -0.48145 | 176 |  |  |
| -0.48019 | 256.2446 | -0.00031 | -0.48029 | 177 |  |  |
| -0.47913 | 257.3127 | -0.00031 | -0.47928 | 178 |  |  |
| -0.47806 | 258.3808 | -0.0003 | -0.47818 | 179 |  |  |
| -0.47699 | 259.4489 | -0.00029 | -0.47708 | 180 |  |  |
| -0.47592 | 260.5171 | -0.00029 | -0.47607 | 181 |  |  |
| -0.47485 | 261.5852 | -0.00028 | -0.47501 | 182 |  |  |
| -0.47379 | 262.6533 | -0.00027 | -0.47391 | 183 |  |  |
| -0.47272 | 263.7214 | -0.00027 | -0.47284 | 184 |  |  |
| -0.47165 | 264.7895 | -0.00026 | -0.47183 | 185 |  |  |
| -0.47058 | 265.8576 | -0.00026 | -0.47073 | 186 |  |  |
| -0.46951 | 266.9257 | -0.00025 | -0.46957 | 187 |  |  |
| -0.46844 | 267.9939 | -0.00024 | -0.46857 | 188 |  |  |
| -0.46738 | 269.062 | -0.00024 | -0.46753 | 189 |  |  |
| -0.46631 | 270.1301 | -0.00023 | -0.4664 | 190 |  |  |
| -0.46524 | 271.1982 | -0.00023 | -0.4653 | 191 |  |  |
| -0.46417 | 272.2663 | -0.00022 | -0.46429 | 192 |  |  |
| -0.4631 | 273.3344 | -0.00021 | -0.4632 | 193 |  |  |
| -0.46204 | 274.4025 | -0.00021 | -0.46219 | 194 |  |  |
| -0.46097 | 275.4707 | -0.0002 | -0.46112 | 195 |  |  |
| -0.4599 | 276.5388 | -0.0002 | -0.45999 | 196 |  |  |
| -0.45883 | 277.6069 | -0.00019 | -0.45898 | 197 |  |  |
| -0.45776 | 278.675 | -0.00019 | -0.45779 | 198 |  |  |
| -0.4567 | 279.7431 | -0.00018 | -0.45676 | 199 |  |  |
| -0.45563 | 280.8112 | -0.00018 | -0.45566 | 200 |  |  |
| -0.45456 | 281.8794 | -0.00017 | -0.45471 | 201 |  |  |
| -0.45349 | 282.9475 | -0.00017 | -0.45361 | 202 |  |  |
| -0.45242 | 284.0156 | -0.00016 | -0.45258 | 203 |  |  |
| -0.45135 | 285.0837 | -0.00016 | -0.45151 | 204 |  |  |
| -0.45029 | 286.1518 | -0.00015 | -0.45044 | 205 |  |  |
| -0.44922 | 287.2199 | -0.00015 | -0.44934 | 206 |  |  |
| -0.44815 | 288.288 | -0.00014 | -0.4483 | 207 |  |  |
| -0.44708 | 289.3562 | -0.00014 | -0.44717 | 208 |  |  |
| -0.44601 | 290.4243 | -0.00013 | -0.44604 | 209 |  |  |
| -0.44495 | 291.4924 | -0.00013 | -0.44495 | 210 |  |  |
| -0.44388 | 292.5605 | -0.00012 | -0.44397 | 211 |  |  |
| -0.44281 | 293.6286 | -0.00012 | -0.44287 | 212 |  |  |
| -0.44174 | 294.6967 | -0.00011 | -0.44196 | 213 |  |  |
| -0.44067 | 295.7648 | -0.00011 | -0.44083 | 214 |  |  |
| -0.43961 | 296.833 | -0.0001 | -0.4397 | 215 |  |  |
| -0.43854 | 297.9011 | -9.6E-05 | -0.43869 | 216 |  |  |
| -0.43747 | 298.9692 | -9.1E-05 | -0.43759 | 217 |  |  |
| -0.4364 | 300.0373 | -8.6E-05 | -0.43646 | 218 |  |  |
| -0.43533 | 301.1054 | -8.1E-05 | -0.43546 | 219 |  |  |
| -0.43427 | 302.1735 | -7.6E-05 | -0.43442 | 220 |  |  |
| -0.4332 | 303.2417 | -7.2E-05 | -0.43332 | 221 |  |  |
| -0.43213 | 304.3098 | -6.7E-05 | -0.43222 | 222 |  |  |
| -0.43106 | 305.3779 | -6.2E-05 | -0.43121 | 223 |  |  |
| -0.42999 | 306.446 | -5.7E-05 | -0.43011 | 224 |  |  |
| -0.42892 | 307.5141 | -5.2E-05 | -0.42889 | 225 |  |  |
| -0.42786 | 308.5822 | -4.7E-05 | -0.42786 | 226 |  |  |
| -0.42679 | 309.6503 | -4.2E-05 | -0.42682 | 227 |  |  |
| -0.42572 | 310.7185 | -3.7E-05 | -0.42578 | 228 |  |  |
| -0.42465 | 311.7866 | -3.2E-05 | -0.42477 | 229 |  |  |
| -0.42358 | 312.8547 | -2.7E-05 | -0.4238 | 230 |  |  |
| -0.42252 | 313.9228 | -2.3E-05 | -0.42273 | 231 |  |  |
| -0.42145 | 314.9909 | -1.9E-05 | -0.42166 | 232 |  |  |
| -0.42038 | 316.059 | -1.4E-05 | -0.42047 | 233 |  |  |
| -0.41931 | 317.1271 | -9.6E-06 | -0.41943 | 234 |  |  |
| -0.41824 | 318.1953 | -4.7E-06 | -0.4183 | 235 |  |  |
| -0.41718 | 319.2634 | -1.8E-07 | -0.41733 | 236 |  |  |
| -0.41611 | 320.3315 | 4.18E-06 | -0.41626 | 237 |  |  |
| -0.41504 | 321.3996 | 8.39E-06 | -0.41513 | 238 |  |  |
| -0.41397 | 322.4677 | 1.36E-05 | -0.4147 | 239 |  |  |
| -0.4129 | 323.5358 | 1.84E-05 | -0.4136 | 240 |  |  |
| -0.41183 | 324.604 | 2.3E-05 | -0.41269 | 241 |  |  |
| -0.41077 | 325.6721 | 2.77E-05 | -0.4111 | 242 |  |  |
| -0.4097 | 326.7402 | 3.24E-05 | -0.41031 | 243 |  |  |
| -0.40863 | 327.8083 | 3.67E-05 | -0.40894 | 244 |  |  |
| -0.40756 | 328.8764 | 4.15E-05 | -0.40787 | 245 |  |  |
| -0.40649 | 329.9445 | 4.68E-05 | -0.40665 | 246 |  |  |
| -0.40543 | 331.0126 | 5.15E-05 | -0.40561 | 247 |  |  |
| -0.40436 | 332.0808 | 5.66E-05 | -0.40463 | 248 |  |  |
| -0.40329 | 333.1489 | 6.12E-05 | -0.40353 | 249 |  |  |
| -0.40222 | 334.217 | 6.62E-05 | -0.40253 | 250 |  |  |
| -0.40115 | 335.2851 | 7.1E-05 | -0.40146 | 251 |  |  |
| -0.40009 | 336.3532 | 7.58E-05 | -0.40039 | 252 |  |  |
| -0.39902 | 337.4213 | 8.06E-05 | -0.39935 | 253 |  |  |
| -0.39795 | 338.4894 | 8.56E-05 | -0.39822 | 254 |  |  |
| -0.39688 | 339.5576 | 9.05E-05 | -0.39725 | 255 |  |  |
| -0.39581 | 340.6257 | 9.55E-05 | -0.39624 | 256 |  |  |
| -0.39474 | 341.6938 | 0.0001 | -0.39514 | 257 |  |  |
| -0.39368 | 342.7619 | 0.000105 | -0.39407 | 258 |  |  |
| -0.39261 | 343.83 | 0.00011 | -0.39291 | 259 |  |  |
| -0.39154 | 344.8981 | 0.000115 | -0.39188 | 260 |  |  |
| -0.39047 | 345.9663 | 0.000121 | -0.39078 | 261 |  |  |
| -0.3894 | 347.0344 | 0.000126 | -0.38962 | 262 |  |  |
| -0.38834 | 348.1025 | 0.000131 | -0.38873 | 263 |  |  |
| -0.38727 | 349.1706 | 0.000136 | -0.3877 | 264 |  |  |
| -0.3862 | 350.2387 | 0.000142 | -0.3866 | 265 |  |  |
| -0.38513 | 351.3068 | 0.000147 | -0.38544 | 266 |  |  |
| -0.38406 | 352.3749 | 0.000152 | -0.38437 | 267 |  |  |
| -0.383 | 353.4431 | 0.000158 | -0.38327 | 268 |  |  |
| -0.38193 | 354.5112 | 0.000163 | -0.38235 | 269 |  |  |
| -0.38086 | 355.5793 | 0.000169 | -0.38135 | 270 |  |  |
| -0.37979 | 356.6474 | 0.000175 | -0.38013 | 271 |  |  |
| -0.37872 | 357.7155 | 0.00018 | -0.37924 | 272 |  |  |
| -0.37766 | 358.7836 | 0.000186 | -0.37802 | 273 |  |  |
| -0.37659 | 359.8517 | 0.000191 | -0.37698 | 274 |  |  |
| -0.37552 | 360.9199 | 0.000198 | -0.37598 | 275 |  |  |
| -0.37445 | 361.988 | 0.000204 | -0.37488 | 276 |  |  |
| -0.37338 | 363.0561 | 0.000209 | -0.37375 | 277 |  |  |
| -0.37231 | 364.1242 | 0.000215 | -0.37271 | 278 |  |  |
| -0.37125 | 365.1923 | 0.000222 | -0.37164 | 279 |  |  |
| -0.37018 | 366.2604 | 0.000228 | -0.37054 | 280 |  |  |
| -0.36911 | 367.3286 | 0.000234 | -0.36948 | 281 |  |  |
| -0.36804 | 368.3967 | 0.000241 | -0.36847 | 282 |  |  |
| -0.36697 | 369.4648 | 0.000248 | -0.36749 | 283 |  |  |
| -0.36591 | 370.5329 | 0.000254 | -0.36642 | 284 |  |  |
| -0.36484 | 371.601 | 0.000261 | -0.3653 | 285 |  |  |
| -0.36377 | 372.6691 | 0.000269 | -0.36417 | 286 |  |  |
| -0.3627 | 373.7372 | 0.000276 | -0.36304 | 287 |  |  |
| -0.36163 | 374.8054 | 0.000283 | -0.36249 | 288 |  |  |
| -0.36057 | 375.8735 | 0.000291 | -0.36105 | 289 |  |  |
| -0.3595 | 376.9416 | 0.000299 | -0.36008 | 290 |  |  |
| -0.35843 | 378.0097 | 0.000307 | -0.35904 | 291 |  |  |
| -0.35736 | 379.0778 | 0.000315 | -0.35751 | 292 |  |  |
| -0.35629 | 380.1459 | 0.000324 | -0.35641 | 293 |  |  |
| -0.35522 | 381.214 | 0.000334 | -0.35541 | 294 |  |  |
| -0.35416 | 382.2822 | 0.000343 | -0.35431 | 295 |  |  |
| -0.35309 | 383.3503 | 0.000353 | -0.3533 | 296 |  |  |
| -0.35202 | 384.4184 | 0.000363 | -0.35217 | 297 |  |  |
| -0.35095 | 385.4865 | 0.000373 | -0.35104 | 298 |  |  |
| -0.34988 | 386.5546 | 0.000384 | -0.35001 | 299 |  |  |
| -0.34882 | 387.6227 | 0.000395 | -0.34894 | 300 |  |  |
| -0.34775 | 388.6909 | 0.000406 | -0.34793 | 301 |  |  |
| -0.34668 | 389.759 | 0.000418 | -0.3468 | 302 |  |  |
| -0.34561 | 390.8271 | 0.000431 | -0.34573 | 303 |  |  |
| -0.34454 | 391.8952 | 0.000444 | -0.3447 | 304 |  |  |
| -0.34348 | 392.9633 | 0.000457 | -0.34354 | 305 |  |  |
| -0.34241 | 394.0314 | 0.00047 | -0.34247 | 306 |  |  |
| -0.34134 | 395.0995 | 0.000485 | -0.34149 | 307 |  |  |
| -0.34027 | 396.1677 | 0.000499 | -0.34048 | 308 |  |  |
| -0.3392 | 397.2358 | 0.000514 | -0.33945 | 309 |  |  |
| -0.33813 | 398.3039 | 0.00053 | -0.33841 | 310 |  |  |
| -0.33707 | 399.372 | 0.000547 | -0.33731 | 311 |  |  |
| -0.336 | 400.4401 | 0.000564 | -0.33627 | 312 |  |  |
| -0.33493 | 401.5082 | 0.000583 | -0.33511 | 313 |  |  |
| -0.33386 | 402.5763 | 0.000602 | -0.33405 | 314 |  |  |
| -0.33279 | 403.6445 | 0.000622 | -0.33295 | 315 |  |  |
| -0.33173 | 404.7126 | 0.000642 | -0.33191 | 316 |  |  |
| -0.33066 | 405.7807 | 0.000664 | -0.33099 | 317 |  |  |
| -0.32959 | 406.8488 | 0.000688 | -0.32974 | 318 |  |  |
| -0.32852 | 407.9169 | 0.000711 | -0.3288 | 319 |  |  |
| -0.32745 | 408.985 | 0.000736 | -0.32776 | 320 |  |  |
| -0.32639 | 410.0532 | 0.000763 | -0.3266 | 321 |  |  |
| -0.32532 | 411.1213 | 0.00079 | -0.32562 | 322 |  |  |
| -0.32425 | 412.1894 | 0.000819 | -0.32449 | 323 |  |  |
| -0.32318 | 413.2575 | 0.000849 | -0.32343 | 324 |  |  |
| -0.32211 | 414.3256 | 0.00088 | -0.3223 | 325 |  |  |
| -0.32104 | 415.3937 | 0.000913 | -0.32129 | 326 |  |  |
| -0.31998 | 416.4618 | 0.000948 | -0.32025 | 327 |  |  |
| -0.31891 | 417.53 | 0.000985 | -0.31915 | 328 |  |  |
| -0.31784 | 418.5981 | 0.001023 | -0.31818 | 329 |  |  |
| -0.31677 | 419.6662 | 0.001063 | -0.31699 | 330 |  |  |
| -0.3157 | 420.7343 | 0.001104 | -0.31592 | 331 |  |  |
| -0.31464 | 421.8024 | 0.001149 | -0.31497 | 332 |  |  |
| -0.31357 | 422.8705 | 0.001196 | -0.31384 | 333 |  |  |
| -0.3125 | 423.9386 | 0.001244 | -0.31287 | 334 |  |  |
| -0.31143 | 425.0068 | 0.001296 | -0.31171 | 335 |  |  |
| -0.31036 | 426.0749 | 0.00135 | -0.31061 | 336 |  |  |
| -0.3093 | 427.143 | 0.001407 | -0.30954 | 337 |  |  |
| -0.30823 | 428.2111 | 0.001468 | -0.30853 | 338 |  |  |
| -0.30716 | 429.2792 | 0.001532 | -0.3075 | 339 |  |  |
| -0.30609 | 430.3473 | 0.0016 | -0.30643 | 340 |  |  |
| -0.30502 | 431.4155 | 0.001673 | -0.30521 | 341 |  |  |
| -0.30396 | 432.4836 | 0.00175 | -0.30417 | 342 |  |  |
| -0.30289 | 433.5517 | 0.00183 | -0.30322 | 343 |  |  |
| -0.30182 | 434.6198 | 0.001916 | -0.30209 | 344 |  |  |
| -0.30075 | 435.6879 | 0.002008 | -0.30103 | 345 |  |  |
| -0.29968 | 436.756 | 0.002107 | -0.29999 | 346 |  |  |
| -0.29861 | 437.8241 | 0.002215 | -0.29895 | 347 |  |  |
| -0.29755 | 438.8923 | 0.00233 | -0.29788 | 348 |  |  |
| -0.29648 | 439.9604 | 0.002452 | -0.29684 | 349 |  |  |
| -0.29541 | 441.0285 | 0.002585 | -0.29581 | 350 |  |  |
| -0.29434 | 442.0966 | 0.002727 | -0.29471 | 351 |  |  |
| -0.29327 | 443.1647 | 0.002882 | -0.29373 | 352 |  |  |
| -0.29221 | 444.2328 | 0.003054 | -0.29263 | 353 |  |  |
| -0.29114 | 445.3009 | 0.003238 | -0.29169 | 354 |  |  |
| -0.29007 | 446.3691 | 0.003437 | -0.29068 | 355 |  |  |
| -0.289 | 447.4372 | 0.003651 | -0.28961 | 356 |  |  |
| -0.28793 | 448.5053 | 0.00388 | -0.28867 | 357 |  |  |
| -0.28687 | 449.5734 | 0.004107 | -0.28757 | 358 |  |  |
| -0.2858 | 450.6415 | 0.004321 | -0.28653 | 359 |  |  |
| -0.28473 | 451.7096 | 0.004513 | -0.28555 | 360 |  |  |
| -0.28366 | 452.7778 | 0.004688 | -0.28445 | 361 |  |  |
| -0.28259 | 453.8459 | 0.004843 | -0.28351 | 362 |  |  |
| -0.28152 | 454.914 | 0.004982 | -0.28247 | 363 |  |  |
| -0.28046 | 455.9821 | 0.005111 | -0.28134 | 364 |  |  |
| -0.27939 | 457.0502 | 0.005234 | -0.28043 | 365 |  |  |
| -0.27832 | 458.1183 | 0.005352 | -0.27936 | 366 |  |  |
| -0.27725 | 459.1864 | 0.005468 | -0.27829 | 367 |  |  |
| -0.27618 | 460.2546 | 0.005581 | -0.27734 | 368 |  |  |
| -0.27512 | 461.3227 | 0.005693 | -0.27621 | 369 |  |  |
| -0.27405 | 462.3908 | 0.005802 | -0.27521 | 370 |  |  |
| -0.27298 | 463.4589 | 0.00591 | -0.27411 | 371 |  |  |
| -0.27191 | 464.527 | 0.006015 | -0.27307 | 372 |  |  |
| -0.27084 | 465.5951 | 0.006117 | -0.27206 | 373 |  |  |
| -0.26978 | 466.6632 | 0.006215 | -0.27094 | 374 |  |  |
| -0.26871 | 467.7314 | 0.006308 | -0.26993 | 375 |  |  |
| -0.26764 | 468.7995 | 0.006397 | -0.26889 | 376 |  |  |
| -0.26657 | 469.8676 | 0.006481 | -0.26794 | 377 |  |  |
| -0.2655 | 470.9357 | 0.006559 | -0.26678 | 378 |  |  |
| -0.26443 | 472.0038 | 0.006632 | -0.26581 | 379 |  |  |
| -0.26337 | 473.0719 | 0.0067 | -0.26468 | 380 |  |  |
| -0.2623 | 474.1401 | 0.006763 | -0.26376 | 381 |  |  |
| -0.26123 | 475.2082 | 0.006821 | -0.26266 | 382 |  |  |
| -0.26016 | 476.2763 | 0.006875 | -0.26157 | 383 |  |  |
| -0.25909 | 477.3444 | 0.006926 | -0.26053 | 384 |  |  |
| -0.25803 | 478.4125 | 0.006973 | -0.25952 | 385 |  |  |
| -0.25696 | 479.4806 | 0.007017 | -0.25836 | 386 |  |  |
| -0.25589 | 480.5487 | 0.007058 | -0.25739 | 387 |  |  |
| -0.25482 | 481.6169 | 0.007097 | -0.25629 | 388 |  |  |
| -0.25375 | 482.685 | 0.007134 | -0.25522 | 389 |  |  |
| -0.25269 | 483.7531 | 0.00717 | -0.25418 | 390 |  |  |
| -0.25162 | 484.8212 | 0.007204 | -0.25311 | 391 |  |  |
| -0.25055 | 485.8893 | 0.007237 | -0.25204 | 392 |  |  |
| -0.24948 | 486.9574 | 0.007269 | -0.25098 | 393 |  |  |
| -0.24841 | 488.0255 | 0.0073 | -0.24994 | 394 |  |  |
| -0.24734 | 489.0937 | 0.007331 | -0.24893 | 395 |  |  |
| -0.24628 | 490.1618 | 0.00736 | -0.24783 | 396 |  |  |
| -0.24521 | 491.2299 | 0.007389 | -0.24677 | 397 |  |  |
| -0.24414 | 492.298 | 0.007418 | -0.24567 | 398 |  |  |
| -0.24307 | 493.3661 | 0.007446 | -0.24454 | 399 |  |  |
| -0.242 | 494.4342 | 0.007473 | -0.24353 | 400 |  |  |
| -0.24094 | 495.5024 | 0.007501 | -0.24255 | 401 |  |  |
| -0.23987 | 496.5705 | 0.007527 | -0.24152 | 402 |  |  |
| -0.2388 | 497.6386 | 0.007554 | -0.24042 | 403 |  |  |
| -0.23773 | 498.7067 | 0.00758 | -0.23938 | 404 |  |  |
| -0.23666 | 499.7748 | 0.007605 | -0.23837 | 405 |  |  |
| -0.2356 | 500.8429 | 0.00763 | -0.23727 | 406 |  |  |
| -0.23453 | 501.911 | 0.007654 | -0.23621 | 407 |  |  |
| -0.23346 | 502.9792 | 0.007678 | -0.23511 | 408 |  |  |
| -0.23239 | 504.0473 | 0.007701 | -0.23401 | 409 |  |  |
| -0.23132 | 505.1154 | 0.007723 | -0.23303 | 410 |  |  |
| -0.23026 | 506.1835 | 0.007746 | -0.23193 | 411 |  |  |
| -0.22919 | 507.2516 | 0.007767 | -0.2308 | 412 |  |  |
| -0.22812 | 508.3197 | 0.007789 | -0.22983 | 413 |  |  |
| -0.22705 | 509.3878 | 0.007809 | -0.22876 | 414 |  |  |
| -0.22598 | 510.456 | 0.007829 | -0.22766 | 415 |  |  |
| -0.22491 | 511.5241 | 0.007849 | -0.22665 | 416 |  |  |
| -0.22385 | 512.5922 | 0.007868 | -0.22549 | 417 |  |  |
| -0.22278 | 513.6603 | 0.007886 | -0.22455 | 418 |  |  |
| -0.22171 | 514.7284 | 0.007903 | -0.22348 | 419 |  |  |
| -0.22064 | 515.7965 | 0.007921 | -0.22238 | 420 |  |  |
| -0.21957 | 516.8647 | 0.007937 | -0.22122 | 421 |  |  |
| -0.21851 | 517.9328 | 0.007954 | -0.22025 | 422 |  |  |
| -0.21744 | 519.0009 | 0.00797 | -0.21921 | 423 |  |  |
| -0.21637 | 520.069 | 0.007985 | -0.21811 | 424 |  |  |
| -0.2153 | 521.1371 | 0.008 | -0.21713 | 425 |  |  |
| -0.21423 | 522.2052 | 0.008015 | -0.21597 | 426 |  |  |
| -0.21317 | 523.2733 | 0.008029 | -0.2149 | 427 |  |  |
| -0.2121 | 524.3415 | 0.008043 | -0.21384 | 428 |  |  |
| -0.21103 | 525.4096 | 0.008058 | -0.2128 | 429 |  |  |
| -0.20996 | 526.4777 | 0.008071 | -0.21176 | 430 |  |  |
| -0.20889 | 527.5458 | 0.008084 | -0.21066 | 431 |  |  |
| -0.20782 | 528.6139 | 0.008096 | -0.20953 | 432 |  |  |
| -0.20676 | 529.682 | 0.008108 | -0.20856 | 433 |  |  |
| -0.20569 | 530.7501 | 0.008121 | -0.20746 | 434 |  |  |
| -0.20462 | 531.8183 | 0.008133 | -0.20639 | 435 |  |  |
| -0.20355 | 532.8864 | 0.008145 | -0.20529 | 436 |  |  |
| -0.20248 | 533.9545 | 0.008156 | -0.20422 | 437 |  |  |
| -0.20142 | 535.0226 | 0.008167 | -0.20325 | 438 |  |  |
| -0.20035 | 536.0907 | 0.008179 | -0.20221 | 439 |  |  |
| -0.19928 | 537.1588 | 0.00819 | -0.20111 | 440 |  |  |
| -0.19821 | 538.227 | 0.008202 | -0.19995 | 441 |  |  |
| -0.19714 | 539.2951 | 0.008212 | -0.19894 | 442 |  |  |
| -0.19608 | 540.3632 | 0.008222 | -0.19791 | 443 |  |  |
| -0.19501 | 541.4313 | 0.008231 | -0.19681 | 444 |  |  |
| -0.19394 | 542.4994 | 0.00824 | -0.19586 | 445 |  |  |
| -0.19287 | 543.5675 | 0.008249 | -0.1947 | 446 |  |  |
| -0.1918 | 544.6356 | 0.00826 | -0.1936 | 447 |  |  |
| -0.19073 | 545.7038 | 0.008267 | -0.19254 | 448 |  |  |
| -0.18967 | 546.7719 | 0.008276 | -0.19144 | 449 |  |  |
| -0.1886 | 547.84 | 0.008287 | -0.19046 | 450 |  |  |
| -0.18753 | 548.9081 | 0.008295 | -0.18939 | 451 |  |  |
| -0.18646 | 549.9762 | 0.008303 | -0.18829 | 452 |  |  |
| -0.18539 | 551.0443 | 0.008311 | -0.18723 | 453 |  |  |
| -0.18433 | 552.1124 | 0.00832 | -0.18616 | 454 |  |  |
| -0.18326 | 553.1806 | 0.008331 | -0.18515 | 455 |  |  |
| -0.18219 | 554.2487 | 0.008339 | -0.18402 | 456 |  |  |
| -0.18112 | 555.3168 | 0.008345 | -0.18289 | 457 |  |  |
| -0.18005 | 556.3849 | 0.008354 | -0.18182 | 458 |  |  |
| -0.17899 | 557.453 | 0.00836 | -0.18079 | 459 |  |  |
| -0.17792 | 558.5211 | 0.008368 | -0.17978 | 460 |  |  |
| -0.17685 | 559.5893 | 0.008377 | -0.17871 | 461 |  |  |
| -0.17578 | 560.6574 | 0.008384 | -0.17761 | 462 |  |  |
| -0.17471 | 561.7255 | 0.008393 | -0.17657 | 463 |  |  |
| -0.17365 | 562.7936 | 0.0084 | -0.17545 | 464 |  |  |
| -0.17258 | 563.8617 | 0.008408 | -0.17441 | 465 |  |  |
| -0.17151 | 564.9298 | 0.008412 | -0.17331 | 466 |  |  |
| -0.17044 | 565.9979 | 0.008425 | -0.1723 | 467 |  |  |
| -0.16937 | 567.0661 | 0.008433 | -0.17114 | 468 |  |  |

Sample: *Yarrow*, Concentration (ppm): 600, Immersion time: 24h

| Potential applied(V) | Time (s) | WE(1).  Current (A) | WE(1).  Potential (V) | Index |  |  |
| --- | --- | --- | --- | --- | --- | --- |
| -0.69885 | 68.27635 | -0.00093 | -0.69684 | 1 |  |  |
| -0.69778 | 69.34447 | -0.00719 | -0.69635 | 2 |  |  |
| -0.69672 | 70.41258 | -0.00716 | -0.69528 | 3 |  |  |
| -0.69565 | 71.4807 | -0.00713 | -0.69431 | 4 |  |  |
| -0.69458 | 72.54881 | -0.00711 | -0.69315 | 5 |  |  |
| -0.69351 | 73.61693 | -0.00708 | -0.69211 | 6 |  |  |
| -0.69244 | 74.68504 | -0.00706 | -0.69095 | 7 |  |  |
| -0.69138 | 75.75316 | -0.00704 | -0.68991 | 8 |  |  |
| -0.69031 | 76.82127 | -0.00702 | -0.68906 | 9 |  |  |
| -0.68924 | 77.88939 | -0.007 | -0.68781 | 10 |  |  |
| -0.68817 | 78.9575 | -0.00698 | -0.68674 | 11 |  |  |
| -0.6871 | 80.02562 | -0.00696 | -0.68567 | 12 |  |  |
| -0.68604 | 81.09373 | -0.00694 | -0.68463 | 13 |  |  |
| -0.68497 | 82.16185 | -0.00692 | -0.68372 | 14 |  |  |
| -0.6839 | 83.22996 | -0.0069 | -0.68253 | 15 |  |  |
| -0.68283 | 84.29808 | -0.00688 | -0.68143 | 16 |  |  |
| -0.68176 | 85.36619 | -0.00686 | -0.68042 | 17 |  |  |
| -0.68069 | 86.43431 | -0.00684 | -0.67938 | 18 |  |  |
| -0.67963 | 87.50242 | -0.00683 | -0.67822 | 19 |  |  |
| -0.67856 | 88.57054 | -0.00681 | -0.67719 | 20 |  |  |
| -0.67749 | 89.63865 | -0.00679 | -0.67624 | 21 |  |  |
| -0.67642 | 90.70677 | -0.00677 | -0.67499 | 22 |  |  |
| -0.67535 | 91.77488 | -0.00675 | -0.67401 | 23 |  |  |
| -0.67429 | 92.843 | -0.00674 | -0.67288 | 24 |  |  |
| -0.67322 | 93.91111 | -0.00672 | -0.67194 | 25 |  |  |
| -0.67215 | 94.97923 | -0.0067 | -0.67087 | 26 |  |  |
| -0.67108 | 96.04734 | -0.00668 | -0.66974 | 27 |  |  |
| -0.67001 | 97.11546 | -0.00666 | -0.66879 | 28 |  |  |
| -0.66895 | 98.18357 | -0.00665 | -0.66772 | 29 |  |  |
| -0.66788 | 99.25169 | -0.00663 | -0.66669 | 30 |  |  |
| -0.66681 | 100.3198 | -0.00661 | -0.66553 | 31 |  |  |
| -0.66574 | 101.3879 | -0.00659 | -0.66452 | 32 |  |  |
| -0.66467 | 102.456 | -0.00657 | -0.66342 | 33 |  |  |
| -0.6636 | 103.5241 | -0.00656 | -0.66251 | 34 |  |  |
| -0.66254 | 104.5923 | -0.00654 | -0.66125 | 35 |  |  |
| -0.66147 | 105.6604 | -0.00652 | -0.66022 | 36 |  |  |
| -0.6604 | 106.7285 | -0.0065 | -0.65903 | 37 |  |  |
| -0.65933 | 107.7966 | -0.00648 | -0.65811 | 38 |  |  |
| -0.65826 | 108.8647 | -0.00647 | -0.65695 | 39 |  |  |
| -0.6572 | 109.9328 | -0.00645 | -0.65585 | 40 |  |  |
| -0.65613 | 111.001 | -0.00643 | -0.65494 | 41 |  |  |
| -0.65506 | 112.0691 | -0.00641 | -0.65381 | 42 |  |  |
| -0.65399 | 113.1372 | -0.00639 | -0.65277 | 43 |  |  |
| -0.65292 | 114.2053 | -0.00637 | -0.65179 | 44 |  |  |
| -0.65186 | 115.2734 | -0.00636 | -0.65079 | 45 |  |  |
| -0.65079 | 116.3415 | -0.00634 | -0.64963 | 46 |  |  |
| -0.64972 | 117.4096 | -0.00632 | -0.64862 | 47 |  |  |
| -0.64865 | 118.4778 | -0.0063 | -0.64752 | 48 |  |  |
| -0.64758 | 119.5459 | -0.00628 | -0.64655 | 49 |  |  |
| -0.64651 | 120.614 | -0.00626 | -0.64532 | 50 |  |  |
| -0.64545 | 121.6821 | -0.00624 | -0.64435 | 51 |  |  |
| -0.64438 | 122.7502 | -0.00622 | -0.64325 | 52 |  |  |
| -0.64331 | 123.8183 | -0.0062 | -0.64212 | 53 |  |  |
| -0.64224 | 124.8864 | -0.00618 | -0.64108 | 54 |  |  |
| -0.64117 | 125.9546 | -0.00617 | -0.64005 | 55 |  |  |
| -0.64011 | 127.0227 | -0.00615 | -0.63898 | 56 |  |  |
| -0.63904 | 128.0908 | -0.00613 | -0.63791 | 57 |  |  |
| -0.63797 | 129.1589 | -0.00611 | -0.6369 | 58 |  |  |
| -0.6369 | 130.227 | -0.00609 | -0.63583 | 59 |  |  |
| -0.63583 | 131.2951 | -0.00607 | -0.6347 | 60 |  |  |
| -0.63477 | 132.3633 | -0.00605 | -0.63367 | 61 |  |  |
| -0.6337 | 133.4314 | -0.00603 | -0.63257 | 62 |  |  |
| -0.63263 | 134.4995 | -0.00601 | -0.63162 | 63 |  |  |
| -0.63156 | 135.5676 | -0.00599 | -0.63052 | 64 |  |  |
| -0.63049 | 136.6357 | -0.00597 | -0.62946 | 65 |  |  |
| -0.62943 | 137.7038 | -0.00595 | -0.62845 | 66 |  |  |
| -0.62836 | 138.7719 | -0.00593 | -0.62735 | 67 |  |  |
| -0.62729 | 139.8401 | -0.00591 | -0.62637 | 68 |  |  |
| -0.62622 | 140.9082 | -0.00588 | -0.62512 | 69 |  |  |
| -0.62515 | 141.9763 | -0.00586 | -0.62427 | 70 |  |  |
| -0.62408 | 143.0444 | -0.00584 | -0.62302 | 71 |  |  |
| -0.62302 | 144.1125 | -0.00582 | -0.62201 | 72 |  |  |
| -0.62195 | 145.1806 | -0.0058 | -0.62094 | 73 |  |  |
| -0.62088 | 146.2487 | -0.00578 | -0.6199 | 74 |  |  |
| -0.61981 | 147.3169 | -0.00575 | -0.61877 | 75 |  |  |
| -0.61874 | 148.385 | -0.00573 | -0.61774 | 76 |  |  |
| -0.61768 | 149.4531 | -0.00571 | -0.61673 | 77 |  |  |
| -0.61661 | 150.5212 | -0.00569 | -0.61572 | 78 |  |  |
| -0.61554 | 151.5893 | -0.00566 | -0.61456 | 79 |  |  |
| -0.61447 | 152.6574 | -0.00564 | -0.61353 | 80 |  |  |
| -0.6134 | 153.7256 | -0.00561 | -0.6124 | 81 |  |  |
| -0.61234 | 154.7937 | -0.00559 | -0.61142 | 82 |  |  |
| -0.61127 | 155.8618 | -0.00557 | -0.61035 | 83 |  |  |
| -0.6102 | 156.9299 | -0.00554 | -0.60931 | 84 |  |  |
| -0.60913 | 157.998 | -0.00552 | -0.60818 | 85 |  |  |
| -0.60806 | 159.0661 | -0.00549 | -0.60709 | 86 |  |  |
| -0.60699 | 160.1342 | -0.00546 | -0.60593 | 87 |  |  |
| -0.60593 | 161.2024 | -0.00544 | -0.60498 | 88 |  |  |
| -0.60486 | 162.2705 | -0.00541 | -0.60394 | 89 |  |  |
| -0.60379 | 163.3386 | -0.00538 | -0.60284 | 90 |  |  |
| -0.60272 | 164.4067 | -0.00535 | -0.60181 | 91 |  |  |
| -0.60165 | 165.4748 | -0.00532 | -0.60083 | 92 |  |  |
| -0.60059 | 166.5429 | -0.00529 | -0.59976 | 93 |  |  |
| -0.59952 | 167.611 | -0.00526 | -0.5986 | 94 |  |  |
| -0.59845 | 168.6792 | -0.00523 | -0.5975 | 95 |  |  |
| -0.59738 | 169.7473 | -0.0052 | -0.59641 | 96 |  |  |
| -0.59631 | 170.8154 | -0.00516 | -0.5954 | 97 |  |  |
| -0.59525 | 171.8835 | -0.00513 | -0.59439 | 98 |  |  |
| -0.59418 | 172.9516 | -0.00509 | -0.59329 | 99 |  |  |
| -0.59311 | 174.0197 | -0.00506 | -0.59229 | 100 |  |  |
| -0.59204 | 175.0879 | -0.00502 | -0.59128 | 101 |  |  |
| -0.59097 | 176.156 | -0.00498 | -0.59018 | 102 |  |  |
| -0.5899 | 177.2241 | -0.00494 | -0.58905 | 103 |  |  |
| -0.58884 | 178.2922 | -0.00489 | -0.58795 | 104 |  |  |
| -0.58777 | 179.3603 | -0.00485 | -0.58685 | 105 |  |  |
| -0.5867 | 180.4284 | -0.0048 | -0.58594 | 106 |  |  |
| -0.58563 | 181.4965 | -0.00475 | -0.58481 | 107 |  |  |
| -0.58456 | 182.5647 | -0.0047 | -0.58389 | 108 |  |  |
| -0.5835 | 183.6328 | -0.00464 | -0.58273 | 109 |  |  |
| -0.58243 | 184.7009 | -0.00459 | -0.58173 | 110 |  |  |
| -0.58136 | 185.769 | -0.00452 | -0.58063 | 111 |  |  |
| -0.58029 | 186.8371 | -0.00446 | -0.57956 | 112 |  |  |
| -0.57922 | 187.9052 | -0.00439 | -0.57849 | 113 |  |  |
| -0.57816 | 188.9733 | -0.00431 | -0.57739 | 114 |  |  |
| -0.57709 | 190.0415 | -0.00424 | -0.57651 | 115 |  |  |
| -0.57602 | 191.1096 | -0.00416 | -0.57553 | 116 |  |  |
| -0.57495 | 192.1777 | -0.00407 | -0.57431 | 117 |  |  |
| -0.57388 | 193.2458 | -0.00399 | -0.57336 | 118 |  |  |
| -0.57281 | 194.3139 | -0.0039 | -0.57227 | 119 |  |  |
| -0.57175 | 195.382 | -0.00381 | -0.5712 | 120 |  |  |
| -0.57068 | 196.4502 | -0.00372 | -0.57019 | 121 |  |  |
| -0.56961 | 197.5183 | -0.00363 | -0.56906 | 122 |  |  |
| -0.56854 | 198.5864 | -0.00354 | -0.56796 | 123 |  |  |
| -0.56747 | 199.6545 | -0.00346 | -0.56708 | 124 |  |  |
| -0.56641 | 200.7226 | -0.00337 | -0.56601 | 125 |  |  |
| -0.56534 | 201.7907 | -0.00329 | -0.56497 | 126 |  |  |
| -0.56427 | 202.8588 | -0.0032 | -0.56384 | 127 |  |  |
| -0.5632 | 203.927 | -0.00312 | -0.56281 | 128 |  |  |
| -0.56213 | 204.9951 | -0.00304 | -0.56171 | 129 |  |  |
| -0.56107 | 206.0632 | -0.00296 | -0.56076 | 130 |  |  |
| -0.56 | 207.1313 | -0.00289 | -0.55969 | 131 |  |  |
| -0.55893 | 208.1994 | -0.00281 | -0.55862 | 132 |  |  |
| -0.55786 | 209.2675 | -0.00274 | -0.55768 | 133 |  |  |
| -0.55679 | 210.3356 | -0.00267 | -0.55655 | 134 |  |  |
| -0.55573 | 211.4038 | -0.0026 | -0.55545 | 135 |  |  |
| -0.55466 | 212.4719 | -0.00254 | -0.55438 | 136 |  |  |
| -0.55359 | 213.54 | -0.00247 | -0.55341 | 137 |  |  |
| -0.55252 | 214.6081 | -0.00241 | -0.55246 | 138 |  |  |
| -0.55145 | 215.6762 | -0.00235 | -0.55133 | 139 |  |  |
| -0.55038 | 216.7443 | -0.00228 | -0.5502 | 140 |  |  |
| -0.54932 | 217.8125 | -0.00223 | -0.54913 | 141 |  |  |
| -0.54825 | 218.8806 | -0.00217 | -0.5481 | 142 |  |  |
| -0.54718 | 219.9487 | -0.00211 | -0.54706 | 143 |  |  |
| -0.54611 | 221.0168 | -0.00206 | -0.54608 | 144 |  |  |
| -0.54504 | 222.0849 | -0.002 | -0.54498 | 145 |  |  |
| -0.54398 | 223.153 | -0.00195 | -0.54395 | 146 |  |  |
| -0.54291 | 224.2211 | -0.0019 | -0.54279 | 147 |  |  |
| -0.54184 | 225.2893 | -0.00185 | -0.54184 | 148 |  |  |
| -0.54077 | 226.3574 | -0.0018 | -0.54062 | 149 |  |  |
| -0.5397 | 227.4255 | -0.00175 | -0.53967 | 150 |  |  |
| -0.53864 | 228.4936 | -0.00171 | -0.53854 | 151 |  |  |
| -0.53757 | 229.5617 | -0.00166 | -0.53751 | 152 |  |  |
| -0.5365 | 230.6298 | -0.00162 | -0.53641 | 153 |  |  |
| -0.53543 | 231.6979 | -0.00158 | -0.53537 | 154 |  |  |
| -0.53436 | 232.7661 | -0.00154 | -0.53442 | 155 |  |  |
| -0.53329 | 233.8342 | -0.0015 | -0.53336 | 156 |  |  |
| -0.53223 | 234.9023 | -0.00146 | -0.53223 | 157 |  |  |
| -0.53116 | 235.9704 | -0.00142 | -0.53113 | 158 |  |  |
| -0.53009 | 237.0385 | -0.00138 | -0.53006 | 159 |  |  |
| -0.52902 | 238.1066 | -0.00134 | -0.52905 | 160 |  |  |
| -0.52795 | 239.1748 | -0.00131 | -0.52792 | 161 |  |  |
| -0.52689 | 240.2429 | -0.00127 | -0.52682 | 162 |  |  |
| -0.52582 | 241.311 | -0.00124 | -0.52594 | 163 |  |  |
| -0.52475 | 242.3791 | -0.00121 | -0.52481 | 164 |  |  |
| -0.52368 | 243.4472 | -0.00117 | -0.52374 | 165 |  |  |
| -0.52261 | 244.5153 | -0.00114 | -0.52267 | 166 |  |  |
| -0.52155 | 245.5834 | -0.00111 | -0.52161 | 167 |  |  |
| -0.52048 | 246.6516 | -0.00108 | -0.52054 | 168 |  |  |
| -0.51941 | 247.7197 | -0.00105 | -0.51938 | 169 |  |  |
| -0.51834 | 248.7878 | -0.00103 | -0.51846 | 170 |  |  |
| -0.51727 | 249.8559 | -0.001 | -0.51752 | 171 |  |  |
| -0.5162 | 250.924 | -0.00097 | -0.51627 | 172 |  |  |
| -0.51514 | 251.9921 | -0.00094 | -0.51526 | 173 |  |  |
| -0.51407 | 253.0602 | -0.00092 | -0.51419 | 174 |  |  |
| -0.513 | 254.1284 | -0.00089 | -0.51306 | 175 |  |  |
| -0.51193 | 255.1965 | -0.00087 | -0.51193 | 176 |  |  |
| -0.51086 | 256.2646 | -0.00085 | -0.51096 | 177 |  |  |
| -0.5098 | 257.3327 | -0.00082 | -0.50986 | 178 |  |  |
| -0.50873 | 258.4008 | -0.0008 | -0.50885 | 179 |  |  |
| -0.50766 | 259.4689 | -0.00078 | -0.50766 | 180 |  |  |
| -0.50659 | 260.5371 | -0.00076 | -0.50668 | 181 |  |  |
| -0.50552 | 261.6052 | -0.00074 | -0.50552 | 182 |  |  |
| -0.50446 | 262.6733 | -0.00072 | -0.50455 | 183 |  |  |
| -0.50339 | 263.7414 | -0.0007 | -0.50351 | 184 |  |  |
| -0.50232 | 264.8095 | -0.00068 | -0.50235 | 185 |  |  |
| -0.50125 | 265.8776 | -0.00066 | -0.50143 | 186 |  |  |
| -0.50018 | 266.9457 | -0.00064 | -0.50034 | 187 |  |  |
| -0.49911 | 268.0139 | -0.00062 | -0.49924 | 188 |  |  |
| -0.49805 | 269.082 | -0.0006 | -0.49817 | 189 |  |  |
| -0.49698 | 270.1501 | -0.00059 | -0.49719 | 190 |  |  |
| -0.49591 | 271.2182 | -0.00057 | -0.49612 | 191 |  |  |
| -0.49484 | 272.2863 | -0.00055 | -0.49509 | 192 |  |  |
| -0.49377 | 273.3544 | -0.00054 | -0.49393 | 193 |  |  |
| -0.49271 | 274.4225 | -0.00052 | -0.49286 | 194 |  |  |
| -0.49164 | 275.4907 | -0.00051 | -0.49185 | 195 |  |  |
| -0.49057 | 276.5588 | -0.00049 | -0.49072 | 196 |  |  |
| -0.4895 | 277.6269 | -0.00048 | -0.48969 | 197 |  |  |
| -0.48843 | 278.695 | -0.00046 | -0.48856 | 198 |  |  |
| -0.48737 | 279.7631 | -0.00045 | -0.48761 | 199 |  |  |
| -0.4863 | 280.8312 | -0.00043 | -0.48642 | 200 |  |  |
| -0.48523 | 281.8994 | -0.00042 | -0.48535 | 201 |  |  |
| -0.48416 | 282.9675 | -0.00041 | -0.48422 | 202 |  |  |
| -0.48309 | 284.0356 | -0.00039 | -0.48325 | 203 |  |  |
| -0.48203 | 285.1037 | -0.00038 | -0.48209 | 204 |  |  |
| -0.48096 | 286.1718 | -0.00037 | -0.48108 | 205 |  |  |
| -0.47989 | 287.2399 | -0.00036 | -0.47995 | 206 |  |  |
| -0.47882 | 288.308 | -0.00035 | -0.47903 | 207 |  |  |
| -0.47775 | 289.3762 | -0.00033 | -0.47787 | 208 |  |  |
| -0.47668 | 290.4443 | -0.00032 | -0.47696 | 209 |  |  |
| -0.47562 | 291.5124 | -0.00031 | -0.47577 | 210 |  |  |
| -0.47455 | 292.5805 | -0.0003 | -0.47479 | 211 |  |  |
| -0.47348 | 293.6486 | -0.00029 | -0.47357 | 212 |  |  |
| -0.47241 | 294.7167 | -0.00028 | -0.47263 | 213 |  |  |
| -0.47134 | 295.7848 | -0.00027 | -0.4715 | 214 |  |  |
| -0.47028 | 296.853 | -0.00026 | -0.47052 | 215 |  |  |
| -0.46921 | 297.9211 | -0.00025 | -0.46942 | 216 |  |  |
| -0.46814 | 298.9892 | -0.00024 | -0.46823 | 217 |  |  |
| -0.46707 | 300.0573 | -0.00023 | -0.46719 | 218 |  |  |
| -0.466 | 301.1254 | -0.00022 | -0.46619 | 219 |  |  |
| -0.46494 | 302.1935 | -0.00021 | -0.46503 | 220 |  |  |
| -0.46387 | 303.2617 | -0.0002 | -0.46408 | 221 |  |  |
| -0.4628 | 304.3298 | -0.00019 | -0.46292 | 222 |  |  |
| -0.46173 | 305.3979 | -0.00019 | -0.46182 | 223 |  |  |
| -0.46066 | 306.466 | -0.00018 | -0.46072 | 224 |  |  |
| -0.45959 | 307.5341 | -0.00017 | -0.45981 | 225 |  |  |
| -0.45853 | 308.6022 | -0.00016 | -0.45871 | 226 |  |  |
| -0.45746 | 309.6703 | -0.00015 | -0.45767 | 227 |  |  |
| -0.45639 | 310.7385 | -0.00014 | -0.45651 | 228 |  |  |
| -0.45532 | 311.8066 | -0.00013 | -0.45551 | 229 |  |  |
| -0.45425 | 312.8747 | -0.00013 | -0.45441 | 230 |  |  |
| -0.45319 | 313.9428 | -0.00012 | -0.45337 | 231 |  |  |
| -0.45212 | 315.0109 | -0.00011 | -0.45224 | 232 |  |  |
| -0.45105 | 316.079 | -0.0001 | -0.45123 | 233 |  |  |
| -0.44998 | 317.1471 | -9.3E-05 | -0.4501 | 234 |  |  |
| -0.44891 | 318.2153 | -8.5E-05 | -0.44916 | 235 |  |  |
| -0.44785 | 319.2834 | -7.7E-05 | -0.44797 | 236 |  |  |
| -0.44678 | 320.3515 | -6.9E-05 | -0.44699 | 237 |  |  |
| -0.44571 | 321.4196 | -6.1E-05 | -0.44592 | 238 |  |  |
| -0.44464 | 322.4877 | -5.4E-05 | -0.44482 | 239 |  |  |
| -0.44357 | 323.5558 | -4.6E-05 | -0.4437 | 240 |  |  |
| -0.4425 | 324.624 | -3.7E-05 | -0.4425 | 241 |  |  |
| -0.44144 | 325.6921 | -2.9E-05 | -0.44156 | 242 |  |  |
| -0.44037 | 326.7602 | -2.1E-05 | -0.44049 | 243 |  |  |
| -0.4393 | 327.8283 | -1.4E-05 | -0.43942 | 244 |  |  |
| -0.43823 | 328.8964 | -5.6E-06 | -0.43845 | 245 |  |  |
| -0.43716 | 329.9645 | 2.5E-06 | -0.43738 | 246 |  |  |
| -0.4361 | 331.0326 | 1.06E-05 | -0.43628 | 247 |  |  |
| -0.43503 | 332.1008 | 1.88E-05 | -0.43518 | 248 |  |  |
| -0.43396 | 333.1689 | 2.69E-05 | -0.43408 | 249 |  |  |
| -0.43289 | 334.237 | 3.49E-05 | -0.43307 | 250 |  |  |
| -0.43182 | 335.3051 | 4.39E-05 | -0.43204 | 251 |  |  |
| -0.43076 | 336.3732 | 5.27E-05 | -0.43106 | 252 |  |  |
| -0.42969 | 337.4413 | 6.14E-05 | -0.42999 | 253 |  |  |
| -0.42862 | 338.5094 | 7.03E-05 | -0.42905 | 254 |  |  |
| -0.42755 | 339.5776 | 7.94E-05 | -0.42789 | 255 |  |  |
| -0.42648 | 340.6457 | 8.84E-05 | -0.42676 | 256 |  |  |
| -0.42542 | 341.7138 | 9.77E-05 | -0.4256 | 257 |  |  |
| -0.42435 | 342.7819 | 0.000107 | -0.4248 | 258 |  |  |
| -0.42328 | 343.85 | 0.000117 | -0.42368 | 259 |  |  |
| -0.42221 | 344.9181 | 0.000127 | -0.42264 | 260 |  |  |
| -0.42114 | 345.9863 | 0.000137 | -0.42145 | 261 |  |  |
| -0.42007 | 347.0544 | 0.000147 | -0.42059 | 262 |  |  |
| -0.41901 | 348.1225 | 0.000158 | -0.4194 | 263 |  |  |
| -0.41794 | 349.1906 | 0.000168 | -0.4184 | 264 |  |  |
| -0.41687 | 350.2587 | 0.00018 | -0.41727 | 265 |  |  |
| -0.4158 | 351.3268 | 0.000191 | -0.41644 | 266 |  |  |
| -0.41473 | 352.3949 | 0.000202 | -0.41522 | 267 |  |  |
| -0.41367 | 353.4631 | 0.000214 | -0.41422 | 268 |  |  |
| -0.4126 | 354.5312 | 0.000226 | -0.41309 | 269 |  |  |
| -0.41153 | 355.5993 | 0.000239 | -0.41205 | 270 |  |  |
| -0.41046 | 356.6674 | 0.000252 | -0.41104 | 271 |  |  |
| -0.40939 | 357.7355 | 0.000265 | -0.40997 | 272 |  |  |
| -0.40833 | 358.8036 | 0.000279 | -0.40891 | 273 |  |  |
| -0.40726 | 359.8717 | 0.000293 | -0.40768 | 274 |  |  |
| -0.40619 | 360.9399 | 0.000308 | -0.40671 | 275 |  |  |
| -0.40512 | 362.008 | 0.000323 | -0.40536 | 276 |  |  |
| -0.40405 | 363.0761 | 0.000339 | -0.40424 | 277 |  |  |
| -0.40298 | 364.1442 | 0.000355 | -0.40311 | 278 |  |  |
| -0.40192 | 365.2123 | 0.000372 | -0.40213 | 279 |  |  |
| -0.40085 | 366.2804 | 0.000389 | -0.40106 | 280 |  |  |
| -0.39978 | 367.3486 | 0.000407 | -0.39993 | 281 |  |  |
| -0.39871 | 368.4167 | 0.000426 | -0.39877 | 282 |  |  |
| -0.39764 | 369.4848 | 0.000445 | -0.39786 | 283 |  |  |
| -0.39658 | 370.5529 | 0.000465 | -0.39679 | 284 |  |  |
| -0.39551 | 371.621 | 0.000486 | -0.39581 | 285 |  |  |
| -0.39444 | 372.6891 | 0.000508 | -0.39465 | 286 |  |  |
| -0.39337 | 373.7572 | 0.000531 | -0.39368 | 287 |  |  |
| -0.3923 | 374.8254 | 0.000554 | -0.39258 | 288 |  |  |
| -0.39124 | 375.8935 | 0.000579 | -0.39157 | 289 |  |  |
| -0.39017 | 376.9616 | 0.000605 | -0.39056 | 290 |  |  |
| -0.3891 | 378.0297 | 0.000631 | -0.38937 | 291 |  |  |
| -0.38803 | 379.0978 | 0.000659 | -0.38837 | 292 |  |  |
| -0.38696 | 380.1659 | 0.000688 | -0.3873 | 293 |  |  |
| -0.38589 | 381.234 | 0.000717 | -0.3862 | 294 |  |  |
| -0.38483 | 382.3022 | 0.000749 | -0.38519 | 295 |  |  |
| -0.38376 | 383.3703 | 0.000781 | -0.38403 | 296 |  |  |
| -0.38269 | 384.4384 | 0.000815 | -0.383 | 297 |  |  |
| -0.38162 | 385.5065 | 0.00085 | -0.38202 | 298 |  |  |
| -0.38055 | 386.5746 | 0.000887 | -0.38089 | 299 |  |  |
| -0.37949 | 387.6427 | 0.000926 | -0.37976 | 300 |  |  |
| -0.37842 | 388.7109 | 0.000966 | -0.37869 | 301 |  |  |
| -0.37735 | 389.779 | 0.001009 | -0.37772 | 302 |  |  |
| -0.37628 | 390.8471 | 0.001053 | -0.37665 | 303 |  |  |
| -0.37521 | 391.9152 | 0.0011 | -0.3754 | 304 |  |  |
| -0.37415 | 392.9833 | 0.001148 | -0.37451 | 305 |  |  |
| -0.37308 | 394.0514 | 0.001199 | -0.37347 | 306 |  |  |
| -0.37201 | 395.1195 | 0.001252 | -0.37231 | 307 |  |  |
| -0.37094 | 396.1877 | 0.001307 | -0.37125 | 308 |  |  |
| -0.36987 | 397.2558 | 0.001364 | -0.37036 | 309 |  |  |
| -0.3688 | 398.3239 | 0.001425 | -0.36911 | 310 |  |  |
| -0.36774 | 399.392 | 0.001489 | -0.36813 | 311 |  |  |
| -0.36667 | 400.4601 | 0.001556 | -0.36703 | 312 |  |  |
| -0.3656 | 401.5282 | 0.001626 | -0.366 | 313 |  |  |
| -0.36453 | 402.5963 | 0.0017 | -0.36493 | 314 |  |  |
| -0.36346 | 403.6645 | 0.001778 | -0.36377 | 315 |  |  |
| -0.3624 | 404.7326 | 0.001859 | -0.36273 | 316 |  |  |
| -0.36133 | 405.8007 | 0.001944 | -0.36157 | 317 |  |  |
| -0.36026 | 406.8688 | 0.002034 | -0.36069 | 318 |  |  |
| -0.35919 | 407.9369 | 0.002127 | -0.35968 | 319 |  |  |
| -0.35812 | 409.005 | 0.002225 | -0.35858 | 320 |  |  |
| -0.35706 | 410.0732 | 0.002328 | -0.35751 | 321 |  |  |
| -0.35599 | 411.1413 | 0.002437 | -0.35651 | 322 |  |  |
| -0.35492 | 412.2094 | 0.002551 | -0.35544 | 323 |  |  |
| -0.35385 | 413.2775 | 0.002672 | -0.35437 | 324 |  |  |
| -0.35278 | 414.3456 | 0.002798 | -0.35336 | 325 |  |  |
| -0.35172 | 415.4137 | 0.002932 | -0.35239 | 326 |  |  |
| -0.35065 | 416.4818 | 0.003072 | -0.35117 | 327 |  |  |
| -0.34958 | 417.55 | 0.00322 | -0.35022 | 328 |  |  |
| -0.34851 | 418.6181 | 0.003374 | -0.34921 | 329 |  |  |
| -0.34744 | 419.6862 | 0.003535 | -0.34814 | 330 |  |  |
| -0.34637 | 420.7543 | 0.003701 | -0.34708 | 331 |  |  |
| -0.34531 | 421.8224 | 0.00387 | -0.34601 | 332 |  |  |
| -0.34424 | 422.8905 | 0.004037 | -0.34506 | 333 |  |  |
| -0.34317 | 423.9586 | 0.004196 | -0.34399 | 334 |  |  |
| -0.3421 | 425.0268 | 0.004345 | -0.34302 | 335 |  |  |
| -0.34103 | 426.0949 | 0.004481 | -0.34204 | 336 |  |  |
| -0.33997 | 427.163 | 0.004604 | -0.34088 | 337 |  |  |
| -0.3389 | 428.2311 | 0.004715 | -0.33987 | 338 |  |  |
| -0.33783 | 429.2992 | 0.004816 | -0.3389 | 339 |  |  |
| -0.33676 | 430.3673 | 0.004908 | -0.33768 | 340 |  |  |
| -0.33569 | 431.4355 | 0.004993 | -0.3367 | 341 |  |  |
| -0.33463 | 432.5036 | 0.005072 | -0.33569 | 342 |  |  |
| -0.33356 | 433.5717 | 0.005146 | -0.3345 | 343 |  |  |
| -0.33249 | 434.6398 | 0.005216 | -0.33356 | 344 |  |  |
| -0.33142 | 435.7079 | 0.005283 | -0.33246 | 345 |  |  |
| -0.33035 | 436.776 | 0.005346 | -0.33145 | 346 |  |  |
| -0.32928 | 437.8441 | 0.005408 | -0.33029 | 347 |  |  |
| -0.32822 | 438.9123 | 0.005468 | -0.32922 | 348 |  |  |
| -0.32715 | 439.9804 | 0.005525 | -0.32828 | 349 |  |  |
| -0.32608 | 441.0485 | 0.005581 | -0.32727 | 350 |  |  |
| -0.32501 | 442.1166 | 0.005636 | -0.32617 | 351 |  |  |
| -0.32394 | 443.1847 | 0.00569 | -0.32526 | 352 |  |  |
| -0.32288 | 444.2528 | 0.005742 | -0.32413 | 353 |  |  |
| -0.32181 | 445.3209 | 0.005794 | -0.32303 | 354 |  |  |
| -0.32074 | 446.3891 | 0.005844 | -0.32187 | 355 |  |  |
| -0.31967 | 447.4572 | 0.005894 | -0.32086 | 356 |  |  |
| -0.3186 | 448.5253 | 0.005944 | -0.31985 | 357 |  |  |
| -0.31754 | 449.5934 | 0.005993 | -0.31882 | 358 |  |  |
| -0.31647 | 450.6615 | 0.006041 | -0.31766 | 359 |  |  |
| -0.3154 | 451.7296 | 0.006089 | -0.31668 | 360 |  |  |
| -0.31433 | 452.7978 | 0.006136 | -0.3157 | 361 |  |  |
| -0.31326 | 453.8659 | 0.006183 | -0.31454 | 362 |  |  |
| -0.31219 | 454.934 | 0.006229 | -0.31348 | 363 |  |  |
| -0.31113 | 456.0021 | 0.006275 | -0.31247 | 364 |  |  |
| -0.31006 | 457.0702 | 0.00632 | -0.31146 | 365 |  |  |
| -0.30899 | 458.1383 | 0.006364 | -0.31033 | 366 |  |  |
| -0.30792 | 459.2064 | 0.006407 | -0.3092 | 367 |  |  |
| -0.30685 | 460.2746 | 0.006451 | -0.30826 | 368 |  |  |
| -0.30579 | 461.3427 | 0.006493 | -0.30719 | 369 |  |  |
| -0.30472 | 462.4108 | 0.006534 | -0.306 | 370 |  |  |
| -0.30365 | 463.4789 | 0.006575 | -0.30496 | 371 |  |  |
| -0.30258 | 464.547 | 0.006614 | -0.30396 | 372 |  |  |
| -0.30151 | 465.6151 | 0.006653 | -0.30298 | 373 |  |  |
| -0.30045 | 466.6832 | 0.00669 | -0.30188 | 374 |  |  |
| -0.29938 | 467.7514 | 0.006727 | -0.30084 | 375 |  |  |
| -0.29831 | 468.8195 | 0.006762 | -0.29971 | 376 |  |  |
| -0.29724 | 469.8876 | 0.006797 | -0.29868 | 377 |  |  |
| -0.29617 | 470.9557 | 0.00683 | -0.29761 | 378 |  |  |
| -0.2951 | 472.0238 | 0.006863 | -0.2966 | 379 |  |  |
| -0.29404 | 473.0919 | 0.006895 | -0.29556 | 380 |  |  |
| -0.29297 | 474.1601 | 0.006926 | -0.29443 | 381 |  |  |
| -0.2919 | 475.2282 | 0.006955 | -0.29343 | 382 |  |  |
| -0.29083 | 476.2963 | 0.006985 | -0.29224 | 383 |  |  |
| -0.28976 | 477.3644 | 0.007013 | -0.29126 | 384 |  |  |
| -0.2887 | 478.4325 | 0.00704 | -0.29025 | 385 |  |  |
| -0.28763 | 479.5006 | 0.007066 | -0.28915 | 386 |  |  |
| -0.28656 | 480.5687 | 0.007092 | -0.28809 | 387 |  |  |
| -0.28549 | 481.6369 | 0.007118 | -0.28702 | 388 |  |  |
| -0.28442 | 482.705 | 0.007142 | -0.28598 | 389 |  |  |
| -0.28336 | 483.7731 | 0.007166 | -0.28488 | 390 |  |  |
| -0.28229 | 484.8412 | 0.007189 | -0.28384 | 391 |  |  |
| -0.28122 | 485.9093 | 0.007212 | -0.28278 | 392 |  |  |
| -0.28015 | 486.9774 | 0.007234 | -0.28171 | 393 |  |  |
| -0.27908 | 488.0455 | 0.007255 | -0.28061 | 394 |  |  |
| -0.27802 | 489.1137 | 0.007275 | -0.27954 | 395 |  |  |
| -0.27695 | 490.1818 | 0.007296 | -0.27847 | 396 |  |  |
| -0.27588 | 491.2499 | 0.007316 | -0.27744 | 397 |  |  |
| -0.27481 | 492.318 | 0.007336 | -0.27637 | 398 |  |  |
| -0.27374 | 493.3861 | 0.007354 | -0.27527 | 399 |  |  |
| -0.27267 | 494.4542 | 0.007372 | -0.27423 | 400 |  |  |
| -0.27161 | 495.5224 | 0.00739 | -0.27319 | 401 |  |  |
| -0.27054 | 496.5905 | 0.007408 | -0.27213 | 402 |  |  |
| -0.26947 | 497.6586 | 0.007425 | -0.27106 | 403 |  |  |
| -0.2684 | 498.7267 | 0.007442 | -0.26996 | 404 |  |  |
| -0.26733 | 499.7948 | 0.007458 | -0.26895 | 405 |  |  |
| -0.26627 | 500.8629 | 0.007474 | -0.26776 | 406 |  |  |
| -0.2652 | 501.931 | 0.007489 | -0.26682 | 407 |  |  |
| -0.26413 | 502.9992 | 0.007504 | -0.26575 | 408 |  |  |
| -0.26306 | 504.0673 | 0.007519 | -0.2648 | 409 |  |  |
| -0.26199 | 505.1354 | 0.007533 | -0.26364 | 410 |  |  |
| -0.26093 | 506.2035 | 0.007548 | -0.26257 | 411 |  |  |
| -0.25986 | 507.2716 | 0.007562 | -0.26144 | 412 |  |  |
| -0.25879 | 508.3397 | 0.007576 | -0.26041 | 413 |  |  |
| -0.25772 | 509.4078 | 0.007589 | -0.25937 | 414 |  |  |
| -0.25665 | 510.476 | 0.007603 | -0.25824 | 415 |  |  |
| -0.25558 | 511.5441 | 0.007616 | -0.25723 | 416 |  |  |
| -0.25452 | 512.6122 | 0.007628 | -0.25613 | 417 |  |  |
| -0.25345 | 513.6803 | 0.007641 | -0.25516 | 418 |  |  |
| -0.25238 | 514.7484 | 0.007653 | -0.25409 | 419 |  |  |
| -0.25131 | 515.8165 | 0.007666 | -0.25293 | 420 |  |  |
| -0.25024 | 516.8847 | 0.007678 | -0.25183 | 421 |  |  |
| -0.24918 | 517.9528 | 0.00769 | -0.25092 | 422 |  |  |
| -0.24811 | 519.0209 | 0.007702 | -0.24979 | 423 |  |  |
| -0.24704 | 520.089 | 0.007714 | -0.24869 | 424 |  |  |
| -0.24597 | 521.1571 | 0.007725 | -0.24774 | 425 |  |  |
| -0.2449 | 522.2252 | 0.007736 | -0.24649 | 426 |  |  |
| -0.24384 | 523.2933 | 0.007747 | -0.24554 | 427 |  |  |
| -0.24277 | 524.3615 | 0.007759 | -0.24448 | 428 |  |  |
| -0.2417 | 525.4296 | 0.00777 | -0.24341 | 429 |  |  |
| -0.24063 | 526.4977 | 0.007781 | -0.24246 | 430 |  |  |
| -0.23956 | 527.5658 | 0.007792 | -0.2413 | 431 |  |  |
| -0.23849 | 528.6339 | 0.007802 | -0.24023 | 432 |  |  |
| -0.23743 | 529.702 | 0.007813 | -0.23911 | 433 |  |  |
| -0.23636 | 530.7701 | 0.007823 | -0.23807 | 434 |  |  |
| -0.23529 | 531.8383 | 0.007833 | -0.23703 | 435 |  |  |
| -0.23422 | 532.9064 | 0.007843 | -0.23587 | 436 |  |  |
| -0.23315 | 533.9745 | 0.007854 | -0.23492 | 437 |  |  |
| -0.23209 | 535.0426 | 0.007864 | -0.23383 | 438 |  |  |
| -0.23102 | 536.1107 | 0.007874 | -0.23282 | 439 |  |  |
| -0.22995 | 537.1788 | 0.007884 | -0.23169 | 440 |  |  |
| -0.22888 | 538.247 | 0.007893 | -0.23065 | 441 |  |  |
| -0.22781 | 539.3151 | 0.007902 | -0.22955 | 442 |  |  |
| -0.22675 | 540.3832 | 0.007911 | -0.22852 | 443 |  |  |
| -0.22568 | 541.4513 | 0.007921 | -0.22742 | 444 |  |  |
| -0.22461 | 542.5194 | 0.007931 | -0.22638 | 445 |  |  |
| -0.22354 | 543.5875 | 0.007939 | -0.22528 | 446 |  |  |
| -0.22247 | 544.6556 | 0.007949 | -0.22406 | 447 |  |  |
| -0.22141 | 545.7238 | 0.007958 | -0.22321 | 448 |  |  |
| -0.22034 | 546.7919 | 0.007966 | -0.22223 | 449 |  |  |
| -0.21927 | 547.86 | 0.007975 | -0.22104 | 450 |  |  |
| -0.2182 | 548.9281 | 0.007983 | -0.21985 | 451 |  |  |
| -0.21713 | 549.9962 | 0.007991 | -0.21881 | 452 |  |  |
| -0.21606 | 551.0643 | 0.007998 | -0.21786 | 453 |  |  |
| -0.215 | 552.1324 | 0.008008 | -0.21671 | 454 |  |  |
| -0.21393 | 553.2006 | 0.008016 | -0.2157 | 455 |  |  |
| -0.21286 | 554.2687 | 0.008024 | -0.2146 | 456 |  |  |
| -0.21179 | 555.3368 | 0.008031 | -0.21347 | 457 |  |  |
| -0.21072 | 556.4049 | 0.00804 | -0.21246 | 458 |  |  |
| -0.20966 | 557.473 | 0.008047 | -0.21149 | 459 |  |  |
| -0.20859 | 558.5411 | 0.008055 | -0.21042 | 460 |  |  |
| -0.20752 | 559.6093 | 0.008063 | -0.20935 | 461 |  |  |
| -0.20645 | 560.6774 | 0.008071 | -0.20825 | 462 |  |  |
| -0.20538 | 561.7455 | 0.008078 | -0.20724 | 463 |  |  |
| -0.20432 | 562.8136 | 0.008085 | -0.20618 | 464 |  |  |
| -0.20325 | 563.8817 | 0.008093 | -0.20496 | 465 |  |  |
| -0.20218 | 564.9498 | 0.0081 | -0.20392 | 466 |  |  |
| -0.20111 | 566.0179 | 0.008108 | -0.20291 | 467 |  |  |
| -0.20004 | 567.0861 | 0.008115 | -0.20197 | 468 |  |  |

Sample: *Yarrow*, Concentration (ppm): 800, Immersion time: 24h

| Potential applied(V) | Time (s) | WE(1).  Current (A) | WE(1).  Potential (V) | Index |  |  |
| --- | --- | --- | --- | --- | --- | --- |
| -0.62775 | 68.30135 | -9.3E-05 | -0.62585 | 1 |  |  |
| -0.62668 | 69.36946 | -0.00094 | -0.62476 | 2 |  |  |
| -0.62561 | 70.43758 | -0.00501 | -0.62469 | 3 |  |  |
| -0.62454 | 71.50569 | -0.00493 | -0.62369 | 4 |  |  |
| -0.62347 | 72.57381 | -0.00486 | -0.62265 | 5 |  |  |
| -0.62241 | 73.64192 | -0.00478 | -0.62155 | 6 |  |  |
| -0.62134 | 74.71004 | -0.0047 | -0.62061 | 7 |  |  |
| -0.62027 | 75.77815 | -0.00463 | -0.61954 | 8 |  |  |
| -0.6192 | 76.84627 | -0.00455 | -0.61841 | 9 |  |  |
| -0.61813 | 77.91438 | -0.00447 | -0.61743 | 10 |  |  |
| -0.61707 | 78.9825 | -0.00439 | -0.61636 | 11 |  |  |
| -0.616 | 80.05061 | -0.00429 | -0.61539 | 12 |  |  |
| -0.61493 | 81.11873 | -0.00419 | -0.61423 | 13 |  |  |
| -0.61386 | 82.18684 | -0.00409 | -0.61322 | 14 |  |  |
| -0.61279 | 83.25496 | -0.00397 | -0.61221 | 15 |  |  |
| -0.61172 | 84.32307 | -0.00386 | -0.61118 | 16 |  |  |
| -0.61066 | 85.39119 | -0.00374 | -0.61005 | 17 |  |  |
| -0.60959 | 86.4593 | -0.00363 | -0.6091 | 18 |  |  |
| -0.60852 | 87.52742 | -0.00352 | -0.60806 | 19 |  |  |
| -0.60745 | 88.59553 | -0.00341 | -0.60706 | 20 |  |  |
| -0.60638 | 89.66365 | -0.0033 | -0.60593 | 21 |  |  |
| -0.60532 | 90.73176 | -0.0032 | -0.60501 | 22 |  |  |
| -0.60425 | 91.79988 | -0.0031 | -0.60391 | 23 |  |  |
| -0.60318 | 92.86799 | -0.003 | -0.60287 | 24 |  |  |
| -0.60211 | 93.93611 | -0.00291 | -0.60178 | 25 |  |  |
| -0.60104 | 95.00422 | -0.00282 | -0.60074 | 26 |  |  |
| -0.59998 | 96.07234 | -0.00274 | -0.59973 | 27 |  |  |
| -0.59891 | 97.14045 | -0.00265 | -0.59869 | 28 |  |  |
| -0.59784 | 98.20857 | -0.00257 | -0.5976 | 29 |  |  |
| -0.59677 | 99.27668 | -0.0025 | -0.59662 | 30 |  |  |
| -0.5957 | 100.3448 | -0.00242 | -0.59552 | 31 |  |  |
| -0.59464 | 101.4129 | -0.00235 | -0.59442 | 32 |  |  |
| -0.59357 | 102.481 | -0.00228 | -0.59344 | 33 |  |  |
| -0.5925 | 103.5491 | -0.00221 | -0.59235 | 34 |  |  |
| -0.59143 | 104.6173 | -0.00215 | -0.59122 | 35 |  |  |
| -0.59036 | 105.6854 | -0.00208 | -0.59027 | 36 |  |  |
| -0.58929 | 106.7535 | -0.00202 | -0.58914 | 37 |  |  |
| -0.58823 | 107.8216 | -0.00196 | -0.5881 | 38 |  |  |
| -0.58716 | 108.8897 | -0.00191 | -0.58707 | 39 |  |  |
| -0.58609 | 109.9578 | -0.00185 | -0.58603 | 40 |  |  |
| -0.58502 | 111.0259 | -0.0018 | -0.58493 | 41 |  |  |
| -0.58395 | 112.0941 | -0.00175 | -0.58389 | 42 |  |  |
| -0.58289 | 113.1622 | -0.0017 | -0.58282 | 43 |  |  |
| -0.58182 | 114.2303 | -0.00165 | -0.58185 | 44 |  |  |
| -0.58075 | 115.2984 | -0.0016 | -0.58072 | 45 |  |  |
| -0.57968 | 116.3665 | -0.00155 | -0.57965 | 46 |  |  |
| -0.57861 | 117.4346 | -0.00151 | -0.5787 | 47 |  |  |
| -0.57755 | 118.5028 | -0.00147 | -0.57748 | 48 |  |  |
| -0.57648 | 119.5709 | -0.00143 | -0.57654 | 49 |  |  |
| -0.57541 | 120.639 | -0.00139 | -0.5755 | 50 |  |  |
| -0.57434 | 121.7071 | -0.00135 | -0.57434 | 51 |  |  |
| -0.57327 | 122.7752 | -0.00131 | -0.5733 | 52 |  |  |
| -0.5722 | 123.8433 | -0.00128 | -0.57224 | 53 |  |  |
| -0.57114 | 124.9114 | -0.00124 | -0.57114 | 54 |  |  |
| -0.57007 | 125.9796 | -0.00121 | -0.57007 | 55 |  |  |
| -0.569 | 127.0477 | -0.00117 | -0.56903 | 56 |  |  |
| -0.56793 | 128.1158 | -0.00114 | -0.56805 | 57 |  |  |
| -0.56686 | 129.1839 | -0.00111 | -0.56696 | 58 |  |  |
| -0.5658 | 130.252 | -0.00108 | -0.56583 | 59 |  |  |
| -0.56473 | 131.3201 | -0.00105 | -0.56485 | 60 |  |  |
| -0.56366 | 132.3882 | -0.00102 | -0.56378 | 61 |  |  |
| -0.56259 | 133.4564 | -0.001 | -0.56262 | 62 |  |  |
| -0.56152 | 134.5245 | -0.00097 | -0.56158 | 63 |  |  |
| -0.56046 | 135.5926 | -0.00094 | -0.56058 | 64 |  |  |
| -0.55939 | 136.6607 | -0.00092 | -0.55939 | 65 |  |  |
| -0.55832 | 137.7288 | -0.0009 | -0.55838 | 66 |  |  |
| -0.55725 | 138.7969 | -0.00087 | -0.55734 | 67 |  |  |
| -0.55618 | 139.8651 | -0.00085 | -0.5563 | 68 |  |  |
| -0.55511 | 140.9332 | -0.00083 | -0.5553 | 69 |  |  |
| -0.55405 | 142.0013 | -0.00081 | -0.55414 | 70 |  |  |
| -0.55298 | 143.0694 | -0.00078 | -0.55304 | 71 |  |  |
| -0.55191 | 144.1375 | -0.00076 | -0.55203 | 72 |  |  |
| -0.55084 | 145.2056 | -0.00074 | -0.55093 | 73 |  |  |
| -0.54977 | 146.2737 | -0.00073 | -0.54996 | 74 |  |  |
| -0.54871 | 147.3419 | -0.00071 | -0.54877 | 75 |  |  |
| -0.54764 | 148.41 | -0.00069 | -0.5477 | 76 |  |  |
| -0.54657 | 149.4781 | -0.00067 | -0.54666 | 77 |  |  |
| -0.5455 | 150.5462 | -0.00065 | -0.5455 | 78 |  |  |
| -0.54443 | 151.6143 | -0.00064 | -0.54456 | 79 |  |  |
| -0.54337 | 152.6824 | -0.00062 | -0.54346 | 80 |  |  |
| -0.5423 | 153.7505 | -0.00061 | -0.54242 | 81 |  |  |
| -0.54123 | 154.8187 | -0.00059 | -0.54132 | 82 |  |  |
| -0.54016 | 155.8868 | -0.00058 | -0.54031 | 83 |  |  |
| -0.53909 | 156.9549 | -0.00056 | -0.53925 | 84 |  |  |
| -0.53802 | 158.023 | -0.00055 | -0.53815 | 85 |  |  |
| -0.53696 | 159.0911 | -0.00054 | -0.53708 | 86 |  |  |
| -0.53589 | 160.1592 | -0.00052 | -0.53604 | 87 |  |  |
| -0.53482 | 161.2274 | -0.00051 | -0.53488 | 88 |  |  |
| -0.53375 | 162.2955 | -0.0005 | -0.53387 | 89 |  |  |
| -0.53268 | 163.3636 | -0.00048 | -0.53281 | 90 |  |  |
| -0.53162 | 164.4317 | -0.00047 | -0.53174 | 91 |  |  |
| -0.53055 | 165.4998 | -0.00046 | -0.53064 | 92 |  |  |
| -0.52948 | 166.5679 | -0.00045 | -0.52966 | 93 |  |  |
| -0.52841 | 167.636 | -0.00044 | -0.52853 | 94 |  |  |
| -0.52734 | 168.7042 | -0.00043 | -0.52747 | 95 |  |  |
| -0.52628 | 169.7723 | -0.00042 | -0.52637 | 96 |  |  |
| -0.52521 | 170.8404 | -0.00041 | -0.52527 | 97 |  |  |
| -0.52414 | 171.9085 | -0.0004 | -0.52426 | 98 |  |  |
| -0.52307 | 172.9766 | -0.00039 | -0.52319 | 99 |  |  |
| -0.522 | 174.0447 | -0.00038 | -0.52209 | 100 |  |  |
| -0.52094 | 175.1128 | -0.00037 | -0.52112 | 101 |  |  |
| -0.51987 | 176.181 | -0.00036 | -0.5199 | 102 |  |  |
| -0.5188 | 177.2491 | -0.00036 | -0.51889 | 103 |  |  |
| -0.51773 | 178.3172 | -0.00035 | -0.51776 | 104 |  |  |
| -0.51666 | 179.3853 | -0.00034 | -0.51682 | 105 |  |  |
| -0.51559 | 180.4534 | -0.00033 | -0.51572 | 106 |  |  |
| -0.51453 | 181.5215 | -0.00033 | -0.51465 | 107 |  |  |
| -0.51346 | 182.5897 | -0.00032 | -0.5137 | 108 |  |  |
| -0.51239 | 183.6578 | -0.00031 | -0.51257 | 109 |  |  |
| -0.51132 | 184.7259 | -0.0003 | -0.51147 | 110 |  |  |
| -0.51025 | 185.794 | -0.0003 | -0.51041 | 111 |  |  |
| -0.50919 | 186.8621 | -0.00029 | -0.50928 | 112 |  |  |
| -0.50812 | 187.9302 | -0.00028 | -0.50827 | 113 |  |  |
| -0.50705 | 188.9983 | -0.00028 | -0.50726 | 114 |  |  |
| -0.50598 | 190.0665 | -0.00027 | -0.5061 | 115 |  |  |
| -0.50491 | 191.1346 | -0.00027 | -0.505 | 116 |  |  |
| -0.50385 | 192.2027 | -0.00026 | -0.50397 | 117 |  |  |
| -0.50278 | 193.2708 | -0.00026 | -0.50287 | 118 |  |  |
| -0.50171 | 194.3389 | -0.00025 | -0.5018 | 119 |  |  |
| -0.50064 | 195.407 | -0.00024 | -0.50076 | 120 |  |  |
| -0.49957 | 196.4751 | -0.00024 | -0.49969 | 121 |  |  |
| -0.4985 | 197.5433 | -0.00023 | -0.49866 | 122 |  |  |
| -0.49744 | 198.6114 | -0.00023 | -0.4975 | 123 |  |  |
| -0.49637 | 199.6795 | -0.00023 | -0.49649 | 124 |  |  |
| -0.4953 | 200.7476 | -0.00022 | -0.49539 | 125 |  |  |
| -0.49423 | 201.8157 | -0.00022 | -0.49432 | 126 |  |  |
| -0.49316 | 202.8838 | -0.00021 | -0.49326 | 127 |  |  |
| -0.4921 | 203.952 | -0.00021 | -0.49231 | 128 |  |  |
| -0.49103 | 205.0201 | -0.0002 | -0.49121 | 129 |  |  |
| -0.48996 | 206.0882 | -0.0002 | -0.49008 | 130 |  |  |
| -0.48889 | 207.1563 | -0.0002 | -0.48907 | 131 |  |  |
| -0.48782 | 208.2244 | -0.00019 | -0.48801 | 132 |  |  |
| -0.48676 | 209.2925 | -0.00019 | -0.48685 | 133 |  |  |
| -0.48569 | 210.3606 | -0.00018 | -0.48581 | 134 |  |  |
| -0.48462 | 211.4288 | -0.00018 | -0.48474 | 135 |  |  |
| -0.48355 | 212.4969 | -0.00018 | -0.4838 | 136 |  |  |
| -0.48248 | 213.565 | -0.00017 | -0.48267 | 137 |  |  |
| -0.48141 | 214.6331 | -0.00017 | -0.4816 | 138 |  |  |
| -0.48035 | 215.7012 | -0.00017 | -0.48059 | 139 |  |  |
| -0.47928 | 216.7693 | -0.00016 | -0.47943 | 140 |  |  |
| -0.47821 | 217.8374 | -0.00016 | -0.4783 | 141 |  |  |
| -0.47714 | 218.9056 | -0.00016 | -0.47726 | 142 |  |  |
| -0.47607 | 219.9737 | -0.00016 | -0.47626 | 143 |  |  |
| -0.47501 | 221.0418 | -0.00015 | -0.47522 | 144 |  |  |
| -0.47394 | 222.1099 | -0.00015 | -0.47415 | 145 |  |  |
| -0.47287 | 223.178 | -0.00015 | -0.47293 | 146 |  |  |
| -0.4718 | 224.2461 | -0.00015 | -0.47192 | 147 |  |  |
| -0.47073 | 225.3143 | -0.00014 | -0.47079 | 148 |  |  |
| -0.46967 | 226.3824 | -0.00014 | -0.46985 | 149 |  |  |
| -0.4686 | 227.4505 | -0.00014 | -0.46875 | 150 |  |  |
| -0.46753 | 228.5186 | -0.00014 | -0.46774 | 151 |  |  |
| -0.46646 | 229.5867 | -0.00013 | -0.46655 | 152 |  |  |
| -0.46539 | 230.6548 | -0.00013 | -0.46555 | 153 |  |  |
| -0.46432 | 231.7229 | -0.00013 | -0.46448 | 154 |  |  |
| -0.46326 | 232.7911 | -0.00013 | -0.46335 | 155 |  |  |
| -0.46219 | 233.8592 | -0.00012 | -0.46231 | 156 |  |  |
| -0.46112 | 234.9273 | -0.00012 | -0.46133 | 157 |  |  |
| -0.46005 | 235.9954 | -0.00012 | -0.46021 | 158 |  |  |
| -0.45898 | 237.0635 | -0.00012 | -0.45917 | 159 |  |  |
| -0.45792 | 238.1316 | -0.00012 | -0.45813 | 160 |  |  |
| -0.45685 | 239.1997 | -0.00011 | -0.45691 | 161 |  |  |
| -0.45578 | 240.2679 | -0.00011 | -0.45587 | 162 |  |  |
| -0.45471 | 241.336 | -0.00011 | -0.45486 | 163 |  |  |
| -0.45364 | 242.4041 | -0.00011 | -0.4537 | 164 |  |  |
| -0.45258 | 243.4722 | -0.00011 | -0.4527 | 165 |  |  |
| -0.45151 | 244.5403 | -0.00011 | -0.4516 | 166 |  |  |
| -0.45044 | 245.6084 | -0.0001 | -0.45059 | 167 |  |  |
| -0.44937 | 246.6766 | -0.0001 | -0.44958 | 168 |  |  |
| -0.4483 | 247.7447 | -0.0001 | -0.4483 | 169 |  |  |
| -0.44724 | 248.8128 | -1E-04 | -0.44736 | 170 |  |  |
| -0.44617 | 249.8809 | -9.8E-05 | -0.44629 | 171 |  |  |
| -0.4451 | 250.949 | -9.7E-05 | -0.44522 | 172 |  |  |
| -0.44403 | 252.0171 | -9.5E-05 | -0.44418 | 173 |  |  |
| -0.44296 | 253.0852 | -9.4E-05 | -0.44308 | 174 |  |  |
| -0.44189 | 254.1534 | -9.2E-05 | -0.44189 | 175 |  |  |
| -0.44083 | 255.2215 | -9.1E-05 | -0.44089 | 176 |  |  |
| -0.43976 | 256.2896 | -9E-05 | -0.43988 | 177 |  |  |
| -0.43869 | 257.3577 | -8.8E-05 | -0.43872 | 178 |  |  |
| -0.43762 | 258.4258 | -8.7E-05 | -0.43765 | 179 |  |  |
| -0.43655 | 259.4939 | -8.6E-05 | -0.43655 | 180 |  |  |
| -0.43549 | 260.562 | -8.4E-05 | -0.43546 | 181 |  |  |
| -0.43442 | 261.6302 | -8.3E-05 | -0.43448 | 182 |  |  |
| -0.43335 | 262.6983 | -8.2E-05 | -0.43338 | 183 |  |  |
| -0.43228 | 263.7664 | -8.1E-05 | -0.4324 | 184 |  |  |
| -0.43121 | 264.8345 | -8E-05 | -0.43118 | 185 |  |  |
| -0.43015 | 265.9026 | -7.8E-05 | -0.43018 | 186 |  |  |
| -0.42908 | 266.9707 | -7.7E-05 | -0.42914 | 187 |  |  |
| -0.42801 | 268.0389 | -7.6E-05 | -0.4281 | 188 |  |  |
| -0.42694 | 269.107 | -7.5E-05 | -0.42709 | 189 |  |  |
| -0.42587 | 270.1751 | -7.4E-05 | -0.42596 | 190 |  |  |
| -0.4248 | 271.2432 | -7.2E-05 | -0.4248 | 191 |  |  |
| -0.42374 | 272.3113 | -7.1E-05 | -0.42383 | 192 |  |  |
| -0.42267 | 273.3794 | -7E-05 | -0.42264 | 193 |  |  |
| -0.4216 | 274.4475 | -6.9E-05 | -0.42154 | 194 |  |  |
| -0.42053 | 275.5157 | -6.8E-05 | -0.42059 | 195 |  |  |
| -0.41946 | 276.5838 | -6.7E-05 | -0.41949 | 196 |  |  |
| -0.4184 | 277.6519 | -6.6E-05 | -0.41849 | 197 |  |  |
| -0.41733 | 278.72 | -6.5E-05 | -0.41745 | 198 |  |  |
| -0.41626 | 279.7881 | -6.4E-05 | -0.41638 | 199 |  |  |
| -0.41519 | 280.8562 | -6.2E-05 | -0.41531 | 200 |  |  |
| -0.41412 | 281.9243 | -6.1E-05 | -0.41415 | 201 |  |  |
| -0.41306 | 282.9925 | -6E-05 | -0.41306 | 202 |  |  |
| -0.41199 | 284.0606 | -5.9E-05 | -0.41211 | 203 |  |  |
| -0.41092 | 285.1287 | -5.8E-05 | -0.41095 | 204 |  |  |
| -0.40985 | 286.1968 | -5.7E-05 | -0.40997 | 205 |  |  |
| -0.40878 | 287.2649 | -5.6E-05 | -0.40872 | 206 |  |  |
| -0.40771 | 288.333 | -5.5E-05 | -0.40781 | 207 |  |  |
| -0.40665 | 289.4012 | -5.4E-05 | -0.40674 | 208 |  |  |
| -0.40558 | 290.4693 | -5.3E-05 | -0.40561 | 209 |  |  |
| -0.40451 | 291.5374 | -5.1E-05 | -0.40466 | 210 |  |  |
| -0.40344 | 292.6055 | -5E-05 | -0.40353 | 211 |  |  |
| -0.40237 | 293.6736 | -4.9E-05 | -0.40237 | 212 |  |  |
| -0.40131 | 294.7417 | -4.8E-05 | -0.40121 | 213 |  |  |
| -0.40024 | 295.8098 | -4.7E-05 | -0.40036 | 214 |  |  |
| -0.39917 | 296.878 | -4.6E-05 | -0.39923 | 215 |  |  |
| -0.3981 | 297.9461 | -4.5E-05 | -0.39819 | 216 |  |  |
| -0.39703 | 299.0142 | -4.3E-05 | -0.39703 | 217 |  |  |
| -0.39597 | 300.0823 | -4.2E-05 | -0.39609 | 218 |  |  |
| -0.3949 | 301.1504 | -4.1E-05 | -0.39502 | 219 |  |  |
| -0.39383 | 302.2185 | -4E-05 | -0.39401 | 220 |  |  |
| -0.39276 | 303.2866 | -3.8E-05 | -0.39282 | 221 |  |  |
| -0.39169 | 304.3548 | -3.7E-05 | -0.39175 | 222 |  |  |
| -0.39063 | 305.4229 | -3.5E-05 | -0.39072 | 223 |  |  |
| -0.38956 | 306.491 | -3.4E-05 | -0.38956 | 224 |  |  |
| -0.38849 | 307.5591 | -3.3E-05 | -0.38858 | 225 |  |  |
| -0.38742 | 308.6272 | -3.1E-05 | -0.38727 | 226 |  |  |
| -0.38635 | 309.6953 | -3E-05 | -0.38632 | 227 |  |  |
| -0.38528 | 310.7635 | -2.9E-05 | -0.38538 | 228 |  |  |
| -0.38422 | 311.8316 | -2.7E-05 | -0.38434 | 229 |  |  |
| -0.38315 | 312.8997 | -2.6E-05 | -0.38333 | 230 |  |  |
| -0.38208 | 313.9678 | -2.4E-05 | -0.38217 | 231 |  |  |
| -0.38101 | 315.0359 | -2.3E-05 | -0.3812 | 232 |  |  |
| -0.37994 | 316.104 | -2.1E-05 | -0.38007 | 233 |  |  |
| -0.37888 | 317.1721 | -1.9E-05 | -0.37888 | 234 |  |  |
| -0.37781 | 318.2403 | -1.8E-05 | -0.37787 | 235 |  |  |
| -0.37674 | 319.3084 | -1.6E-05 | -0.3768 | 236 |  |  |
| -0.37567 | 320.3765 | -1.4E-05 | -0.37567 | 237 |  |  |
| -0.3746 | 321.4446 | -1.2E-05 | -0.37466 | 238 |  |  |
| -0.37354 | 322.5127 | -1E-05 | -0.3736 | 239 |  |  |
| -0.37247 | 323.5808 | -8.2E-06 | -0.3725 | 240 |  |  |
| -0.3714 | 324.6489 | -6.2E-06 | -0.37149 | 241 |  |  |
| -0.37033 | 325.7171 | -4.2E-06 | -0.3703 | 242 |  |  |
| -0.36926 | 326.7852 | -1.9E-06 | -0.36929 | 243 |  |  |
| -0.36819 | 327.8533 | 4.36E-07 | -0.36835 | 244 |  |  |
| -0.36713 | 328.9214 | 2.69E-06 | -0.36752 | 245 |  |  |
| -0.36606 | 329.9895 | 5.18E-06 | -0.36694 | 246 |  |  |
| -0.36499 | 331.0576 | 7.67E-06 | -0.3653 | 247 |  |  |
| -0.36392 | 332.1258 | 1.01E-05 | -0.36423 | 248 |  |  |
| -0.36285 | 333.1939 | 1.33E-05 | -0.36304 | 249 |  |  |
| -0.36179 | 334.262 | 1.62E-05 | -0.36203 | 250 |  |  |
| -0.36072 | 335.3301 | 1.9E-05 | -0.36108 | 251 |  |  |
| -0.35965 | 336.3982 | 2.19E-05 | -0.3602 | 252 |  |  |
| -0.35858 | 337.4663 | 2.55E-05 | -0.35907 | 253 |  |  |
| -0.35751 | 338.5344 | 2.87E-05 | -0.35834 | 254 |  |  |
| -0.35645 | 339.6026 | 3.21E-05 | -0.35693 | 255 |  |  |
| -0.35538 | 340.6707 | 3.54E-05 | -0.35556 | 256 |  |  |
| -0.35431 | 341.7388 | 3.99E-05 | -0.35471 | 257 |  |  |
| -0.35324 | 342.8069 | 4.39E-05 | -0.35342 | 258 |  |  |
| -0.35217 | 343.875 | 4.83E-05 | -0.35248 | 259 |  |  |
| -0.3511 | 344.9431 | 5.29E-05 | -0.35141 | 260 |  |  |
| -0.35004 | 346.0112 | 5.77E-05 | -0.35028 | 261 |  |  |
| -0.34897 | 347.0794 | 6.25E-05 | -0.3493 | 262 |  |  |
| -0.3479 | 348.1475 | 6.75E-05 | -0.34821 | 263 |  |  |
| -0.34683 | 349.2156 | 7.29E-05 | -0.34705 | 264 |  |  |
| -0.34576 | 350.2837 | 7.89E-05 | -0.34598 | 265 |  |  |
| -0.3447 | 351.3518 | 8.49E-05 | -0.34485 | 266 |  |  |
| -0.34363 | 352.4199 | 9.11E-05 | -0.34399 | 267 |  |  |
| -0.34256 | 353.4881 | 9.79E-05 | -0.3429 | 268 |  |  |
| -0.34149 | 354.5562 | 0.000105 | -0.3418 | 269 |  |  |
| -0.34042 | 355.6243 | 0.000112 | -0.34082 | 270 |  |  |
| -0.33936 | 356.6924 | 0.00012 | -0.33972 | 271 |  |  |
| -0.33829 | 357.7605 | 0.000129 | -0.33865 | 272 |  |  |
| -0.33722 | 358.8286 | 0.000137 | -0.33755 | 273 |  |  |
| -0.33615 | 359.8967 | 0.000146 | -0.33652 | 274 |  |  |
| -0.33508 | 360.9649 | 0.000157 | -0.33545 | 275 |  |  |
| -0.33401 | 362.033 | 0.000167 | -0.33435 | 276 |  |  |
| -0.33295 | 363.1011 | 0.000178 | -0.33322 | 277 |  |  |
| -0.33188 | 364.1692 | 0.00019 | -0.33231 | 278 |  |  |
| -0.33081 | 365.2373 | 0.000202 | -0.33118 | 279 |  |  |
| -0.32974 | 366.3054 | 0.000215 | -0.33011 | 280 |  |  |
| -0.32867 | 367.3735 | 0.00023 | -0.32895 | 281 |  |  |
| -0.32761 | 368.4417 | 0.000245 | -0.32806 | 282 |  |  |
| -0.32654 | 369.5098 | 0.000261 | -0.32697 | 283 |  |  |
| -0.32547 | 370.5779 | 0.000278 | -0.32587 | 284 |  |  |
| -0.3244 | 371.646 | 0.000296 | -0.32474 | 285 |  |  |
| -0.32333 | 372.7141 | 0.000315 | -0.32397 | 286 |  |  |
| -0.32227 | 373.7822 | 0.000336 | -0.32242 | 287 |  |  |
| -0.3212 | 374.8504 | 0.000358 | -0.32132 | 288 |  |  |
| -0.32013 | 375.9185 | 0.000381 | -0.32037 | 289 |  |  |
| -0.31906 | 376.9866 | 0.000405 | -0.31937 | 290 |  |  |
| -0.31799 | 378.0547 | 0.000431 | -0.31812 | 291 |  |  |
| -0.31693 | 379.1228 | 0.000459 | -0.31708 | 292 |  |  |
| -0.31586 | 380.1909 | 0.000488 | -0.31598 | 293 |  |  |
| -0.31479 | 381.259 | 0.000519 | -0.31491 | 294 |  |  |
| -0.31372 | 382.3272 | 0.000552 | -0.314 | 295 |  |  |
| -0.31265 | 383.3953 | 0.000588 | -0.31293 | 296 |  |  |
| -0.31158 | 384.4634 | 0.000627 | -0.3118 | 297 |  |  |
| -0.31052 | 385.5315 | 0.000667 | -0.3107 | 298 |  |  |
| -0.30945 | 386.5996 | 0.00071 | -0.30963 | 299 |  |  |
| -0.30838 | 387.6677 | 0.000754 | -0.30862 | 300 |  |  |
| -0.30731 | 388.7358 | 0.000802 | -0.30743 | 301 |  |  |
| -0.30624 | 389.804 | 0.000854 | -0.30637 | 302 |  |  |
| -0.30518 | 390.8721 | 0.000908 | -0.30539 | 303 |  |  |
| -0.30411 | 391.9402 | 0.000965 | -0.30429 | 304 |  |  |
| -0.30304 | 393.0083 | 0.001026 | -0.30325 | 305 |  |  |
| -0.30197 | 394.0764 | 0.001091 | -0.30215 | 306 |  |  |
| -0.3009 | 395.1445 | 0.001159 | -0.30109 | 307 |  |  |
| -0.29984 | 396.2127 | 0.001232 | -0.30005 | 308 |  |  |
| -0.29877 | 397.2808 | 0.001311 | -0.29895 | 309 |  |  |
| -0.2977 | 398.3489 | 0.001394 | -0.29797 | 310 |  |  |
| -0.29663 | 399.417 | 0.001481 | -0.29688 | 311 |  |  |
| -0.29556 | 400.4851 | 0.001574 | -0.29584 | 312 |  |  |
| -0.29449 | 401.5532 | 0.001675 | -0.29483 | 313 |  |  |
| -0.29343 | 402.6213 | 0.001782 | -0.2937 | 314 |  |  |
| -0.29236 | 403.6895 | 0.001899 | -0.29269 | 315 |  |  |
| -0.29129 | 404.7576 | 0.002023 | -0.29166 | 316 |  |  |
| -0.29022 | 405.8257 | 0.002158 | -0.2905 | 317 |  |  |
| -0.28915 | 406.8938 | 0.002305 | -0.28952 | 318 |  |  |
| -0.28809 | 407.9619 | 0.002464 | -0.28839 | 319 |  |  |
| -0.28702 | 409.03 | 0.002636 | -0.28735 | 320 |  |  |
| -0.28595 | 410.0981 | 0.002828 | -0.28644 | 321 |  |  |
| -0.28488 | 411.1663 | 0.00304 | -0.28528 | 322 |  |  |
| -0.28381 | 412.2344 | 0.003272 | -0.28439 | 323 |  |  |
| -0.28275 | 413.3025 | 0.003525 | -0.28333 | 324 |  |  |
| -0.28168 | 414.3706 | 0.003799 | -0.28238 | 325 |  |  |
| -0.28061 | 415.4387 | 0.00408 | -0.28134 | 326 |  |  |
| -0.27954 | 416.5068 | 0.004342 | -0.28033 | 327 |  |  |
| -0.27847 | 417.575 | 0.004572 | -0.27936 | 328 |  |  |
| -0.2774 | 418.6431 | 0.004771 | -0.27835 | 329 |  |  |
| -0.27634 | 419.7112 | 0.004941 | -0.27719 | 330 |  |  |
| -0.27527 | 420.7793 | 0.005092 | -0.27618 | 331 |  |  |
| -0.2742 | 421.8474 | 0.005229 | -0.27512 | 332 |  |  |
| -0.27313 | 422.9155 | 0.005355 | -0.27417 | 333 |  |  |
| -0.27206 | 423.9836 | 0.005472 | -0.27301 | 334 |  |  |
| -0.271 | 425.0518 | 0.005586 | -0.27206 | 335 |  |  |
| -0.26993 | 426.1199 | 0.005695 | -0.27094 | 336 |  |  |
| -0.26886 | 427.188 | 0.0058 | -0.27008 | 337 |  |  |
| -0.26779 | 428.2561 | 0.005899 | -0.26892 | 338 |  |  |
| -0.26672 | 429.3242 | 0.005993 | -0.26788 | 339 |  |  |
| -0.26566 | 430.3923 | 0.006083 | -0.26688 | 340 |  |  |
| -0.26459 | 431.4604 | 0.006166 | -0.26575 | 341 |  |  |
| -0.26352 | 432.5286 | 0.006245 | -0.26483 | 342 |  |  |
| -0.26245 | 433.5967 | 0.006318 | -0.26361 | 343 |  |  |
| -0.26138 | 434.6648 | 0.006388 | -0.26266 | 344 |  |  |
| -0.26031 | 435.7329 | 0.006454 | -0.26157 | 345 |  |  |
| -0.25925 | 436.801 | 0.006515 | -0.26068 | 346 |  |  |
| -0.25818 | 437.8691 | 0.006574 | -0.25946 | 347 |  |  |
| -0.25711 | 438.9373 | 0.00663 | -0.25842 | 348 |  |  |
| -0.25604 | 440.0054 | 0.006682 | -0.25742 | 349 |  |  |
| -0.25497 | 441.0735 | 0.006732 | -0.25644 | 350 |  |  |
| -0.25391 | 442.1416 | 0.00678 | -0.25531 | 351 |  |  |
| -0.25284 | 443.2097 | 0.006825 | -0.25424 | 352 |  |  |
| -0.25177 | 444.2778 | 0.006868 | -0.25323 | 353 |  |  |
| -0.2507 | 445.3459 | 0.006909 | -0.25208 | 354 |  |  |
| -0.24963 | 446.4141 | 0.006949 | -0.25116 | 355 |  |  |
| -0.24857 | 447.4822 | 0.006986 | -0.25003 | 356 |  |  |
| -0.2475 | 448.5503 | 0.007021 | -0.24896 | 357 |  |  |
| -0.24643 | 449.6184 | 0.007056 | -0.24786 | 358 |  |  |
| -0.24536 | 450.6865 | 0.007089 | -0.2468 | 359 |  |  |
| -0.24429 | 451.7546 | 0.00712 | -0.24579 | 360 |  |  |
| -0.24323 | 452.8227 | 0.007149 | -0.24475 | 361 |  |  |
| -0.24216 | 453.8909 | 0.007178 | -0.24371 | 362 |  |  |
| -0.24109 | 454.959 | 0.007205 | -0.24249 | 363 |  |  |
| -0.24002 | 456.0271 | 0.007231 | -0.24155 | 364 |  |  |
| -0.23895 | 457.0952 | 0.007256 | -0.24054 | 365 |  |  |
| -0.23788 | 458.1633 | 0.007281 | -0.23953 | 366 |  |  |
| -0.23682 | 459.2314 | 0.007304 | -0.23834 | 367 |  |  |
| -0.23575 | 460.2996 | 0.007327 | -0.23727 | 368 |  |  |
| -0.23468 | 461.3677 | 0.007348 | -0.23633 | 369 |  |  |
| -0.23361 | 462.4358 | 0.007369 | -0.23517 | 370 |  |  |
| -0.23254 | 463.5039 | 0.00739 | -0.2341 | 371 |  |  |
| -0.23148 | 464.572 | 0.00741 | -0.23306 | 372 |  |  |
| -0.23041 | 465.6401 | 0.007429 | -0.23203 | 373 |  |  |
| -0.22934 | 466.7082 | 0.007448 | -0.23096 | 374 |  |  |
| -0.22827 | 467.7764 | 0.007467 | -0.22983 | 375 |  |  |
| -0.2272 | 468.8445 | 0.007485 | -0.22876 | 376 |  |  |
| -0.22614 | 469.9126 | 0.007502 | -0.22772 | 377 |  |  |
| -0.22507 | 470.9807 | 0.00752 | -0.22668 | 378 |  |  |
| -0.224 | 472.0488 | 0.007537 | -0.22559 | 379 |  |  |
| -0.22293 | 473.1169 | 0.007554 | -0.22458 | 380 |  |  |
| -0.22186 | 474.185 | 0.00757 | -0.22351 | 381 |  |  |
| -0.22079 | 475.2532 | 0.007587 | -0.2225 | 382 |  |  |
| -0.21973 | 476.3213 | 0.007603 | -0.22134 | 383 |  |  |
| -0.21866 | 477.3894 | 0.007619 | -0.22025 | 384 |  |  |
| -0.21759 | 478.4575 | 0.007635 | -0.21924 | 385 |  |  |
| -0.21652 | 479.5256 | 0.00765 | -0.21811 | 386 |  |  |
| -0.21545 | 480.5937 | 0.007666 | -0.21704 | 387 |  |  |
| -0.21439 | 481.6619 | 0.007681 | -0.21603 | 388 |  |  |
| -0.21332 | 482.73 | 0.007696 | -0.21487 | 389 |  |  |
| -0.21225 | 483.7981 | 0.007711 | -0.21393 | 390 |  |  |
| -0.21118 | 484.8662 | 0.007725 | -0.2128 | 391 |  |  |
| -0.21011 | 485.9343 | 0.007739 | -0.21173 | 392 |  |  |
| -0.20905 | 487.0024 | 0.007753 | -0.21066 | 393 |  |  |
| -0.20798 | 488.0705 | 0.007767 | -0.20969 | 394 |  |  |
| -0.20691 | 489.1387 | 0.00778 | -0.20862 | 395 |  |  |
| -0.20584 | 490.2068 | 0.007795 | -0.20761 | 396 |  |  |
| -0.20477 | 491.2749 | 0.007809 | -0.20651 | 397 |  |  |
| -0.2037 | 492.343 | 0.007821 | -0.20538 | 398 |  |  |
| -0.20264 | 493.4111 | 0.007834 | -0.20425 | 399 |  |  |
| -0.20157 | 494.4792 | 0.007847 | -0.20322 | 400 |  |  |
| -0.2005 | 495.5473 | 0.007859 | -0.20221 | 401 |  |  |
| -0.19943 | 496.6155 | 0.007872 | -0.20108 | 402 |  |  |
| -0.19836 | 497.6836 | 0.007884 | -0.20004 | 403 |  |  |
| -0.1973 | 498.7517 | 0.007896 | -0.19901 | 404 |  |  |
| -0.19623 | 499.8198 | 0.007908 | -0.19797 | 405 |  |  |
| -0.19516 | 500.8879 | 0.00792 | -0.19693 | 406 |  |  |
| -0.19409 | 501.956 | 0.007932 | -0.19583 | 407 |  |  |
| -0.19302 | 503.0242 | 0.007943 | -0.19476 | 408 |  |  |
| -0.19196 | 504.0923 | 0.007954 | -0.19363 | 409 |  |  |
| -0.19089 | 505.1604 | 0.007965 | -0.19263 | 410 |  |  |
| -0.18982 | 506.2285 | 0.007976 | -0.19147 | 411 |  |  |
| -0.18875 | 507.2966 | 0.007987 | -0.19055 | 412 |  |  |
| -0.18768 | 508.3647 | 0.007997 | -0.1893 | 413 |  |  |
| -0.18661 | 509.4328 | 0.008008 | -0.18839 | 414 |  |  |
| -0.18555 | 510.501 | 0.008018 | -0.18744 | 415 |  |  |
| -0.18448 | 511.5691 | 0.008028 | -0.18622 | 416 |  |  |
| -0.18341 | 512.6372 | 0.008037 | -0.18518 | 417 |  |  |
| -0.18234 | 513.7053 | 0.008047 | -0.18408 | 418 |  |  |
| -0.18127 | 514.7734 | 0.008058 | -0.18304 | 419 |  |  |
| -0.18021 | 515.8415 | 0.008067 | -0.18198 | 420 |  |  |
| -0.17914 | 516.9096 | 0.008076 | -0.18091 | 421 |  |  |
| -0.17807 | 517.9778 | 0.008086 | -0.17978 | 422 |  |  |
| -0.177 | 519.0459 | 0.008095 | -0.1788 | 423 |  |  |
| -0.17593 | 520.114 | 0.008104 | -0.17773 | 424 |  |  |
| -0.17487 | 521.1821 | 0.008113 | -0.17661 | 425 |  |  |
| -0.1738 | 522.2502 | 0.008122 | -0.17557 | 426 |  |  |
| -0.17273 | 523.3183 | 0.008131 | -0.1745 | 427 |  |  |
| -0.17166 | 524.3865 | 0.00814 | -0.17361 | 428 |  |  |
| -0.17059 | 525.4546 | 0.008148 | -0.17242 | 429 |  |  |
| -0.16953 | 526.5227 | 0.008157 | -0.1712 | 430 |  |  |
| -0.16846 | 527.5908 | 0.008165 | -0.17026 | 431 |  |  |
| -0.16739 | 528.6589 | 0.008173 | -0.16922 | 432 |  |  |
| -0.16632 | 529.727 | 0.008181 | -0.16815 | 433 |  |  |
| -0.16525 | 530.7951 | 0.008189 | -0.16718 | 434 |  |  |
| -0.16418 | 531.8633 | 0.008198 | -0.16602 | 435 |  |  |
| -0.16312 | 532.9314 | 0.008205 | -0.16492 | 436 |  |  |
| -0.16205 | 533.9995 | 0.008212 | -0.16388 | 437 |  |  |
| -0.16098 | 535.0676 | 0.00822 | -0.16278 | 438 |  |  |
| -0.15991 | 536.1357 | 0.008228 | -0.16171 | 439 |  |  |
| -0.15884 | 537.2038 | 0.008236 | -0.16068 | 440 |  |  |
| -0.15778 | 538.2719 | 0.008244 | -0.15955 | 441 |  |  |
| -0.15671 | 539.3401 | 0.008251 | -0.15848 | 442 |  |  |
| -0.15564 | 540.4082 | 0.008257 | -0.15756 | 443 |  |  |
| -0.15457 | 541.4763 | 0.008264 | -0.15643 | 444 |  |  |
| -0.1535 | 542.5444 | 0.00827 | -0.15536 | 445 |  |  |
| -0.15244 | 543.6125 | 0.008276 | -0.1543 | 446 |  |  |
| -0.15137 | 544.6806 | 0.008284 | -0.1532 | 447 |  |  |
| -0.1503 | 545.7488 | 0.008293 | -0.15213 | 448 |  |  |
| -0.14923 | 546.8169 | 0.0083 | -0.15097 | 449 |  |  |
| -0.14816 | 547.885 | 0.008306 | -0.15009 | 450 |  |  |
| -0.14709 | 548.9531 | 0.008313 | -0.1488 | 451 |  |  |
| -0.14603 | 550.0212 | 0.008319 | -0.14786 | 452 |  |  |
| -0.14496 | 551.0893 | 0.008326 | -0.14673 | 453 |  |  |
| -0.14389 | 552.1574 | 0.008333 | -0.14563 | 454 |  |  |
| -0.14282 | 553.2256 | 0.008341 | -0.14471 | 455 |  |  |
| -0.14175 | 554.2937 | 0.008347 | -0.14359 | 456 |  |  |
| -0.14069 | 555.3618 | 0.008353 | -0.14246 | 457 |  |  |
| -0.13962 | 556.4299 | 0.008358 | -0.14154 | 458 |  |  |
| -0.13855 | 557.498 | 0.008364 | -0.14044 | 459 |  |  |
| -0.13748 | 558.5661 | 0.008369 | -0.13934 | 460 |  |  |
| -0.13641 | 559.6342 | 0.008374 | -0.13828 | 461 |  |  |
| -0.13535 | 560.7024 | 0.008379 | -0.13724 | 462 |  |  |
| -0.13428 | 561.7705 | 0.008385 | -0.13617 | 463 |  |  |
| -0.13321 | 562.8386 | 0.008392 | -0.13504 | 464 |  |  |
| -0.13214 | 563.9067 | 0.008399 | -0.13394 | 465 |  |  |
| -0.13107 | 564.9748 | 0.008406 | -0.13287 | 466 |  |  |
| -0.13 | 566.0429 | 0.008412 | -0.13187 | 467 |  |  |
| -0.12894 | 567.1111 | 0.008417 | -0.13077 | 468 |  |  |
| -0.12787 | 568.1792 | 0.008422 | -0.12979 | 469 |  |  |

Sample: *Ribes Rubrum*, Concentration (ppm): 200, Immersion time: 24h

| Potential applied(V) | Time (s) | WE(1).  Current (A) | WE(1).  Potential (V) | Index |  |  |
| --- | --- | --- | --- | --- | --- | --- |
| -0.70313 | 68.62036 | -9.3E-05 | -0.70105 | 1 |  |  |
| -0.70206 | 69.68848 | -0.00093 | -0.70001 | 2 |  |  |
| -0.70099 | 70.75659 | -0.0073 | -0.6994 | 3 |  |  |
| -0.69992 | 71.82471 | -0.00727 | -0.69843 | 4 |  |  |
| -0.69885 | 72.89282 | -0.00724 | -0.69733 | 5 |  |  |
| -0.69778 | 73.96094 | -0.00722 | -0.69626 | 6 |  |  |
| -0.69672 | 75.02905 | -0.00719 | -0.69525 | 7 |  |  |
| -0.69565 | 76.09717 | -0.00717 | -0.69415 | 8 |  |  |
| -0.69458 | 77.16528 | -0.00715 | -0.69308 | 9 |  |  |
| -0.69351 | 78.2334 | -0.00712 | -0.69205 | 10 |  |  |
| -0.69244 | 79.30151 | -0.0071 | -0.69104 | 11 |  |  |
| -0.69138 | 80.36963 | -0.00708 | -0.68988 | 12 |  |  |
| -0.69031 | 81.43774 | -0.00706 | -0.68884 | 13 |  |  |
| -0.68924 | 82.50586 | -0.00704 | -0.68784 | 14 |  |  |
| -0.68817 | 83.57397 | -0.00702 | -0.6868 | 15 |  |  |
| -0.6871 | 84.64209 | -0.007 | -0.68561 | 16 |  |  |
| -0.68604 | 85.7102 | -0.00698 | -0.68457 | 17 |  |  |
| -0.68497 | 86.77832 | -0.00696 | -0.68356 | 18 |  |  |
| -0.6839 | 87.84643 | -0.00694 | -0.6824 | 19 |  |  |
| -0.68283 | 88.91455 | -0.00692 | -0.68149 | 20 |  |  |
| -0.68176 | 89.98266 | -0.0069 | -0.68027 | 21 |  |  |
| -0.68069 | 91.05078 | -0.00689 | -0.67929 | 22 |  |  |
| -0.67963 | 92.11889 | -0.00687 | -0.67813 | 23 |  |  |
| -0.67856 | 93.18701 | -0.00685 | -0.67715 | 24 |  |  |
| -0.67749 | 94.25512 | -0.00683 | -0.67615 | 25 |  |  |
| -0.67642 | 95.32324 | -0.00681 | -0.67502 | 26 |  |  |
| -0.67535 | 96.39135 | -0.00679 | -0.67404 | 27 |  |  |
| -0.67429 | 97.45947 | -0.00677 | -0.67291 | 28 |  |  |
| -0.67322 | 98.52758 | -0.00675 | -0.67188 | 29 |  |  |
| -0.67215 | 99.5957 | -0.00673 | -0.67078 | 30 |  |  |
| -0.67108 | 100.6638 | -0.00672 | -0.66965 | 31 |  |  |
| -0.67001 | 101.7319 | -0.0067 | -0.66867 | 32 |  |  |
| -0.66895 | 102.8 | -0.00668 | -0.66757 | 33 |  |  |
| -0.66788 | 103.8682 | -0.00666 | -0.66653 | 34 |  |  |
| -0.66681 | 104.9363 | -0.00664 | -0.66553 | 35 |  |  |
| -0.66574 | 106.0044 | -0.00662 | -0.66446 | 36 |  |  |
| -0.66467 | 107.0725 | -0.0066 | -0.66333 | 37 |  |  |
| -0.6636 | 108.1406 | -0.00659 | -0.66226 | 38 |  |  |
| -0.66254 | 109.2087 | -0.00657 | -0.66129 | 39 |  |  |
| -0.66147 | 110.2768 | -0.00655 | -0.66025 | 40 |  |  |
| -0.6604 | 111.345 | -0.00653 | -0.65915 | 41 |  |  |
| -0.65933 | 112.4131 | -0.00651 | -0.65799 | 42 |  |  |
| -0.65826 | 113.4812 | -0.00649 | -0.65695 | 43 |  |  |
| -0.6572 | 114.5493 | -0.00647 | -0.65594 | 44 |  |  |
| -0.65613 | 115.6174 | -0.00645 | -0.65482 | 45 |  |  |
| -0.65506 | 116.6855 | -0.00643 | -0.65378 | 46 |  |  |
| -0.65399 | 117.7537 | -0.00641 | -0.65283 | 47 |  |  |
| -0.65292 | 118.8218 | -0.00639 | -0.65167 | 48 |  |  |
| -0.65186 | 119.8899 | -0.00638 | -0.65063 | 49 |  |  |
| -0.65079 | 120.958 | -0.00636 | -0.64954 | 50 |  |  |
| -0.64972 | 122.0261 | -0.00634 | -0.6485 | 51 |  |  |
| -0.64865 | 123.0942 | -0.00632 | -0.6474 | 52 |  |  |
| -0.64758 | 124.1623 | -0.0063 | -0.64636 | 53 |  |  |
| -0.64651 | 125.2305 | -0.00628 | -0.64532 | 54 |  |  |
| -0.64545 | 126.2986 | -0.00626 | -0.64429 | 55 |  |  |
| -0.64438 | 127.3667 | -0.00624 | -0.64316 | 56 |  |  |
| -0.64331 | 128.4348 | -0.00622 | -0.64212 | 57 |  |  |
| -0.64224 | 129.5029 | -0.0062 | -0.64105 | 58 |  |  |
| -0.64117 | 130.571 | -0.00618 | -0.64001 | 59 |  |  |
| -0.64011 | 131.6391 | -0.00616 | -0.63895 | 60 |  |  |
| -0.63904 | 132.7073 | -0.00614 | -0.63791 | 61 |  |  |
| -0.63797 | 133.7754 | -0.00612 | -0.63684 | 62 |  |  |
| -0.6369 | 134.8435 | -0.0061 | -0.63571 | 63 |  |  |
| -0.63583 | 135.9116 | -0.00608 | -0.6347 | 64 |  |  |
| -0.63477 | 136.9797 | -0.00606 | -0.63364 | 65 |  |  |
| -0.6337 | 138.0478 | -0.00604 | -0.63254 | 66 |  |  |
| -0.63263 | 139.116 | -0.00602 | -0.63147 | 67 |  |  |
| -0.63156 | 140.1841 | -0.00599 | -0.6304 | 68 |  |  |
| -0.63049 | 141.2522 | -0.00597 | -0.62936 | 69 |  |  |
| -0.62943 | 142.3203 | -0.00595 | -0.62833 | 70 |  |  |
| -0.62836 | 143.3884 | -0.00593 | -0.62726 | 71 |  |  |
| -0.62729 | 144.4565 | -0.00591 | -0.62619 | 72 |  |  |
| -0.62622 | 145.5246 | -0.00589 | -0.62515 | 73 |  |  |
| -0.62515 | 146.5928 | -0.00587 | -0.62399 | 74 |  |  |
| -0.62408 | 147.6609 | -0.00584 | -0.62302 | 75 |  |  |
| -0.62302 | 148.729 | -0.00582 | -0.62201 | 76 |  |  |
| -0.62195 | 149.7971 | -0.0058 | -0.62088 | 77 |  |  |
| -0.62088 | 150.8652 | -0.00578 | -0.61978 | 78 |  |  |
| -0.61981 | 151.9333 | -0.00575 | -0.61874 | 79 |  |  |
| -0.61874 | 153.0014 | -0.00573 | -0.61771 | 80 |  |  |
| -0.61768 | 154.0696 | -0.0057 | -0.61658 | 81 |  |  |
| -0.61661 | 155.1377 | -0.00568 | -0.61557 | 82 |  |  |
| -0.61554 | 156.2058 | -0.00566 | -0.61453 | 83 |  |  |
| -0.61447 | 157.2739 | -0.00563 | -0.61337 | 84 |  |  |
| -0.6134 | 158.342 | -0.00561 | -0.6123 | 85 |  |  |
| -0.61234 | 159.4101 | -0.00558 | -0.61127 | 86 |  |  |
| -0.61127 | 160.4783 | -0.00556 | -0.61029 | 87 |  |  |
| -0.6102 | 161.5464 | -0.00553 | -0.60919 | 88 |  |  |
| -0.60913 | 162.6145 | -0.0055 | -0.60806 | 89 |  |  |
| -0.60806 | 163.6826 | -0.00548 | -0.60715 | 90 |  |  |
| -0.60699 | 164.7507 | -0.00545 | -0.60602 | 91 |  |  |
| -0.60593 | 165.8188 | -0.00542 | -0.60489 | 92 |  |  |
| -0.60486 | 166.8869 | -0.0054 | -0.60391 | 93 |  |  |
| -0.60379 | 167.9551 | -0.00537 | -0.60284 | 94 |  |  |
| -0.60272 | 169.0232 | -0.00534 | -0.60178 | 95 |  |  |
| -0.60165 | 170.0913 | -0.00531 | -0.60068 | 96 |  |  |
| -0.60059 | 171.1594 | -0.00528 | -0.59964 | 97 |  |  |
| -0.59952 | 172.2275 | -0.00524 | -0.59854 | 98 |  |  |
| -0.59845 | 173.2956 | -0.00521 | -0.59747 | 99 |  |  |
| -0.59738 | 174.3637 | -0.00518 | -0.59641 | 100 |  |  |
| -0.59631 | 175.4319 | -0.00514 | -0.5954 | 101 |  |  |
| -0.59525 | 176.5 | -0.00511 | -0.59436 | 102 |  |  |
| -0.59418 | 177.5681 | -0.00507 | -0.59335 | 103 |  |  |
| -0.59311 | 178.6362 | -0.00504 | -0.59219 | 104 |  |  |
| -0.59204 | 179.7043 | -0.005 | -0.59113 | 105 |  |  |
| -0.59097 | 180.7724 | -0.00496 | -0.59012 | 106 |  |  |
| -0.5899 | 181.8406 | -0.00491 | -0.58908 | 107 |  |  |
| -0.58884 | 182.9087 | -0.00487 | -0.58798 | 108 |  |  |
| -0.58777 | 183.9768 | -0.00482 | -0.58701 | 109 |  |  |
| -0.5867 | 185.0449 | -0.00478 | -0.58585 | 110 |  |  |
| -0.58563 | 186.113 | -0.00472 | -0.58484 | 111 |  |  |
| -0.58456 | 187.1811 | -0.00467 | -0.5838 | 112 |  |  |
| -0.5835 | 188.2492 | -0.00461 | -0.58273 | 113 |  |  |
| -0.58243 | 189.3174 | -0.00455 | -0.58163 | 114 |  |  |
| -0.58136 | 190.3855 | -0.00449 | -0.58063 | 115 |  |  |
| -0.58029 | 191.4536 | -0.00442 | -0.57953 | 116 |  |  |
| -0.57922 | 192.5217 | -0.00435 | -0.57849 | 117 |  |  |
| -0.57816 | 193.5898 | -0.00427 | -0.57745 | 118 |  |  |
| -0.57709 | 194.6579 | -0.00419 | -0.57639 | 119 |  |  |
| -0.57602 | 195.726 | -0.00411 | -0.57529 | 120 |  |  |
| -0.57495 | 196.7942 | -0.00402 | -0.57431 | 121 |  |  |
| -0.57388 | 197.8623 | -0.00393 | -0.5733 | 122 |  |  |
| -0.57281 | 198.9304 | -0.00384 | -0.5722 | 123 |  |  |
| -0.57175 | 199.9985 | -0.00375 | -0.5712 | 124 |  |  |
| -0.57068 | 201.0666 | -0.00366 | -0.57013 | 125 |  |  |
| -0.56961 | 202.1347 | -0.00357 | -0.56906 | 126 |  |  |
| -0.56854 | 203.2029 | -0.00348 | -0.56799 | 127 |  |  |
| -0.56747 | 204.271 | -0.00339 | -0.56702 | 128 |  |  |
| -0.56641 | 205.3391 | -0.00331 | -0.56592 | 129 |  |  |
| -0.56534 | 206.4072 | -0.00322 | -0.56494 | 130 |  |  |
| -0.56427 | 207.4753 | -0.00314 | -0.56387 | 131 |  |  |
| -0.5632 | 208.5434 | -0.00305 | -0.56277 | 132 |  |  |
| -0.56213 | 209.6115 | -0.00297 | -0.56174 | 133 |  |  |
| -0.56107 | 210.6797 | -0.0029 | -0.56061 | 134 |  |  |
| -0.56 | 211.7478 | -0.00282 | -0.5596 | 135 |  |  |
| -0.55893 | 212.8159 | -0.00275 | -0.55862 | 136 |  |  |
| -0.55786 | 213.884 | -0.00267 | -0.55756 | 137 |  |  |
| -0.55679 | 214.9521 | -0.0026 | -0.55652 | 138 |  |  |
| -0.55573 | 216.0202 | -0.00253 | -0.55533 | 139 |  |  |
| -0.55466 | 217.0883 | -0.00247 | -0.55441 | 140 |  |  |
| -0.55359 | 218.1565 | -0.0024 | -0.55344 | 141 |  |  |
| -0.55252 | 219.2246 | -0.00234 | -0.55234 | 142 |  |  |
| -0.55145 | 220.2927 | -0.00228 | -0.55118 | 143 |  |  |
| -0.55038 | 221.3608 | -0.00222 | -0.55011 | 144 |  |  |
| -0.54932 | 222.4289 | -0.00216 | -0.54916 | 145 |  |  |
| -0.54825 | 223.497 | -0.0021 | -0.548 | 146 |  |  |
| -0.54718 | 224.5652 | -0.00204 | -0.54706 | 147 |  |  |
| -0.54611 | 225.6333 | -0.00199 | -0.54596 | 148 |  |  |
| -0.54504 | 226.7014 | -0.00194 | -0.54486 | 149 |  |  |
| -0.54398 | 227.7695 | -0.00188 | -0.54382 | 150 |  |  |
| -0.54291 | 228.8376 | -0.00183 | -0.54282 | 151 |  |  |
| -0.54184 | 229.9057 | -0.00178 | -0.54172 | 152 |  |  |
| -0.54077 | 230.9738 | -0.00174 | -0.54074 | 153 |  |  |
| -0.5397 | 232.042 | -0.00169 | -0.53958 | 154 |  |  |
| -0.53864 | 233.1101 | -0.00164 | -0.53854 | 155 |  |  |
| -0.53757 | 234.1782 | -0.0016 | -0.53751 | 156 |  |  |
| -0.5365 | 235.2463 | -0.00156 | -0.53644 | 157 |  |  |
| -0.53543 | 236.3144 | -0.00152 | -0.5354 | 158 |  |  |
| -0.53436 | 237.3825 | -0.00147 | -0.5343 | 159 |  |  |
| -0.53329 | 238.4506 | -0.00143 | -0.53326 | 160 |  |  |
| -0.53223 | 239.5188 | -0.0014 | -0.53223 | 161 |  |  |
| -0.53116 | 240.5869 | -0.00136 | -0.53116 | 162 |  |  |
| -0.53009 | 241.655 | -0.00132 | -0.53006 | 163 |  |  |
| -0.52902 | 242.7231 | -0.00129 | -0.52899 | 164 |  |  |
| -0.52795 | 243.7912 | -0.00125 | -0.52795 | 165 |  |  |
| -0.52689 | 244.8593 | -0.00122 | -0.52689 | 166 |  |  |
| -0.52582 | 245.9275 | -0.00118 | -0.52579 | 167 |  |  |
| -0.52475 | 246.9956 | -0.00115 | -0.52484 | 168 |  |  |
| -0.52368 | 248.0637 | -0.00112 | -0.52356 | 169 |  |  |
| -0.52261 | 249.1318 | -0.00109 | -0.52261 | 170 |  |  |
| -0.52155 | 250.1999 | -0.00106 | -0.52161 | 171 |  |  |
| -0.52048 | 251.268 | -0.00103 | -0.52048 | 172 |  |  |
| -0.51941 | 252.3361 | -0.001 | -0.51941 | 173 |  |  |
| -0.51834 | 253.4043 | -0.00098 | -0.5184 | 174 |  |  |
| -0.51727 | 254.4724 | -0.00095 | -0.51727 | 175 |  |  |
| -0.5162 | 255.5405 | -0.00092 | -0.51624 | 176 |  |  |
| -0.51514 | 256.6086 | -0.0009 | -0.5152 | 177 |  |  |
| -0.51407 | 257.6767 | -0.00087 | -0.51413 | 178 |  |  |
| -0.513 | 258.7448 | -0.00085 | -0.51303 | 179 |  |  |
| -0.51193 | 259.8129 | -0.00083 | -0.51202 | 180 |  |  |
| -0.51086 | 260.8811 | -0.0008 | -0.51093 | 181 |  |  |
| -0.5098 | 261.9492 | -0.00078 | -0.50986 | 182 |  |  |
| -0.50873 | 263.0173 | -0.00076 | -0.50882 | 183 |  |  |
| -0.50766 | 264.0854 | -0.00074 | -0.50772 | 184 |  |  |
| -0.50659 | 265.1535 | -0.00072 | -0.50674 | 185 |  |  |
| -0.50552 | 266.2216 | -0.0007 | -0.50558 | 186 |  |  |
| -0.50446 | 267.2898 | -0.00068 | -0.50455 | 187 |  |  |
| -0.50339 | 268.3579 | -0.00066 | -0.50351 | 188 |  |  |
| -0.50232 | 269.426 | -0.00064 | -0.50238 | 189 |  |  |
| -0.50125 | 270.4941 | -0.00062 | -0.50134 | 190 |  |  |
| -0.50018 | 271.5622 | -0.0006 | -0.50024 | 191 |  |  |
| -0.49911 | 272.6303 | -0.00059 | -0.49918 | 192 |  |  |
| -0.49805 | 273.6984 | -0.00057 | -0.49817 | 193 |  |  |
| -0.49698 | 274.7666 | -0.00055 | -0.49701 | 194 |  |  |
| -0.49591 | 275.8347 | -0.00054 | -0.496 | 195 |  |  |
| -0.49484 | 276.9028 | -0.00052 | -0.49493 | 196 |  |  |
| -0.49377 | 277.9709 | -0.0005 | -0.49387 | 197 |  |  |
| -0.49271 | 279.039 | -0.00049 | -0.49283 | 198 |  |  |
| -0.49164 | 280.1071 | -0.00048 | -0.49173 | 199 |  |  |
| -0.49057 | 281.1752 | -0.00046 | -0.49063 | 200 |  |  |
| -0.4895 | 282.2434 | -0.00045 | -0.48962 | 201 |  |  |
| -0.48843 | 283.3115 | -0.00043 | -0.48856 | 202 |  |  |
| -0.48737 | 284.3796 | -0.00042 | -0.48746 | 203 |  |  |
| -0.4863 | 285.4477 | -0.00041 | -0.48639 | 204 |  |  |
| -0.48523 | 286.5158 | -0.00039 | -0.48529 | 205 |  |  |
| -0.48416 | 287.5839 | -0.00038 | -0.48428 | 206 |  |  |
| -0.48309 | 288.6521 | -0.00037 | -0.48318 | 207 |  |  |
| -0.48203 | 289.7202 | -0.00036 | -0.48212 | 208 |  |  |
| -0.48096 | 290.7883 | -0.00034 | -0.48099 | 209 |  |  |
| -0.47989 | 291.8564 | -0.00033 | -0.47998 | 210 |  |  |
| -0.47882 | 292.9245 | -0.00032 | -0.47891 | 211 |  |  |
| -0.47775 | 293.9926 | -0.00031 | -0.47784 | 212 |  |  |
| -0.47668 | 295.0607 | -0.0003 | -0.47681 | 213 |  |  |
| -0.47562 | 296.1289 | -0.00029 | -0.47574 | 214 |  |  |
| -0.47455 | 297.197 | -0.00028 | -0.4747 | 215 |  |  |
| -0.47348 | 298.2651 | -0.00027 | -0.4736 | 216 |  |  |
| -0.47241 | 299.3332 | -0.00026 | -0.4726 | 217 |  |  |
| -0.47134 | 300.4013 | -0.00025 | -0.47144 | 218 |  |  |
| -0.47028 | 301.4694 | -0.00024 | -0.47043 | 219 |  |  |
| -0.46921 | 302.5375 | -0.00023 | -0.46933 | 220 |  |  |
| -0.46814 | 303.6057 | -0.00022 | -0.46829 | 221 |  |  |
| -0.46707 | 304.6738 | -0.00021 | -0.46719 | 222 |  |  |
| -0.466 | 305.7419 | -0.0002 | -0.46613 | 223 |  |  |
| -0.46494 | 306.81 | -0.00019 | -0.46509 | 224 |  |  |
| -0.46387 | 307.8781 | -0.00018 | -0.46399 | 225 |  |  |
| -0.4628 | 308.9462 | -0.00017 | -0.46301 | 226 |  |  |
| -0.46173 | 310.0144 | -0.00016 | -0.46188 | 227 |  |  |
| -0.46066 | 311.0825 | -0.00016 | -0.46082 | 228 |  |  |
| -0.45959 | 312.1506 | -0.00015 | -0.45975 | 229 |  |  |
| -0.45853 | 313.2187 | -0.00014 | -0.45871 | 230 |  |  |
| -0.45746 | 314.2868 | -0.00013 | -0.45761 | 231 |  |  |
| -0.45639 | 315.3549 | -0.00012 | -0.45648 | 232 |  |  |
| -0.45532 | 316.423 | -0.00011 | -0.45538 | 233 |  |  |
| -0.45425 | 317.4912 | -0.00011 | -0.45438 | 234 |  |  |
| -0.45319 | 318.5593 | -9.7E-05 | -0.45331 | 235 |  |  |
| -0.45212 | 319.6274 | -8.9E-05 | -0.45221 | 236 |  |  |
| -0.45105 | 320.6955 | -8.1E-05 | -0.45117 | 237 |  |  |
| -0.44998 | 321.7636 | -7.3E-05 | -0.4501 | 238 |  |  |
| -0.44891 | 322.8317 | -6.5E-05 | -0.44907 | 239 |  |  |
| -0.44785 | 323.8998 | -5.7E-05 | -0.44791 | 240 |  |  |
| -0.44678 | 324.968 | -4.9E-05 | -0.44693 | 241 |  |  |
| -0.44571 | 326.0361 | -4.1E-05 | -0.4458 | 242 |  |  |
| -0.44464 | 327.1042 | -3.2E-05 | -0.44482 | 243 |  |  |
| -0.44357 | 328.1723 | -2.4E-05 | -0.44379 | 244 |  |  |
| -0.4425 | 329.2404 | -1.6E-05 | -0.44257 | 245 |  |  |
| -0.44144 | 330.3085 | -8.2E-06 | -0.44162 | 246 |  |  |
| -0.44037 | 331.3767 | -3.7E-07 | -0.44049 | 247 |  |  |
| -0.4393 | 332.4448 | 7.63E-06 | -0.43951 | 248 |  |  |
| -0.43823 | 333.5129 | 1.59E-05 | -0.43839 | 249 |  |  |
| -0.43716 | 334.581 | 2.37E-05 | -0.43735 | 250 |  |  |
| -0.4361 | 335.6491 | 3.17E-05 | -0.43631 | 251 |  |  |
| -0.43503 | 336.7172 | 4.12E-05 | -0.43521 | 252 |  |  |
| -0.43396 | 337.7853 | 5E-05 | -0.43411 | 253 |  |  |
| -0.43289 | 338.8535 | 5.85E-05 | -0.43314 | 254 |  |  |
| -0.43182 | 339.9216 | 6.75E-05 | -0.43216 | 255 |  |  |
| -0.43076 | 340.9897 | 7.66E-05 | -0.43106 | 256 |  |  |
| -0.42969 | 342.0578 | 8.55E-05 | -0.43005 | 257 |  |  |
| -0.42862 | 343.1259 | 9.49E-05 | -0.42883 | 258 |  |  |
| -0.42755 | 344.194 | 0.000104 | -0.42786 | 259 |  |  |
| -0.42648 | 345.2621 | 0.000114 | -0.42676 | 260 |  |  |
| -0.42542 | 346.3303 | 0.000124 | -0.42569 | 261 |  |  |
| -0.42435 | 347.3984 | 0.000134 | -0.42474 | 262 |  |  |
| -0.42328 | 348.4665 | 0.000144 | -0.42374 | 263 |  |  |
| -0.42221 | 349.5346 | 0.000154 | -0.42249 | 264 |  |  |
| -0.42114 | 350.6027 | 0.000165 | -0.42145 | 265 |  |  |
| -0.42007 | 351.6708 | 0.000176 | -0.42044 | 266 |  |  |
| -0.41901 | 352.739 | 0.000187 | -0.41946 | 267 |  |  |
| -0.41794 | 353.8071 | 0.000199 | -0.41846 | 268 |  |  |
| -0.41687 | 354.8752 | 0.000211 | -0.41727 | 269 |  |  |
| -0.4158 | 355.9433 | 0.000223 | -0.41632 | 270 |  |  |
| -0.41473 | 357.0114 | 0.000235 | -0.41519 | 271 |  |  |
| -0.41367 | 358.0795 | 0.000248 | -0.41415 | 272 |  |  |
| -0.4126 | 359.1476 | 0.000261 | -0.41312 | 273 |  |  |
| -0.41153 | 360.2158 | 0.000275 | -0.41208 | 274 |  |  |
| -0.41046 | 361.2839 | 0.000289 | -0.41101 | 275 |  |  |
| -0.40939 | 362.352 | 0.000304 | -0.41 | 276 |  |  |
| -0.40833 | 363.4201 | 0.000319 | -0.40863 | 277 |  |  |
| -0.40726 | 364.4882 | 0.000335 | -0.40738 | 278 |  |  |
| -0.40619 | 365.5563 | 0.000351 | -0.40634 | 279 |  |  |
| -0.40512 | 366.6244 | 0.000368 | -0.4053 | 280 |  |  |
| -0.40405 | 367.6926 | 0.000385 | -0.40424 | 281 |  |  |
| -0.40298 | 368.7607 | 0.000403 | -0.40314 | 282 |  |  |
| -0.40192 | 369.8288 | 0.000422 | -0.40204 | 283 |  |  |
| -0.40085 | 370.8969 | 0.000441 | -0.40106 | 284 |  |  |
| -0.39978 | 371.965 | 0.000461 | -0.39999 | 285 |  |  |
| -0.39871 | 373.0331 | 0.000482 | -0.39893 | 286 |  |  |
| -0.39764 | 374.1013 | 0.000504 | -0.39777 | 287 |  |  |
| -0.39658 | 375.1694 | 0.000527 | -0.39676 | 288 |  |  |
| -0.39551 | 376.2375 | 0.000551 | -0.39575 | 289 |  |  |
| -0.39444 | 377.3056 | 0.000575 | -0.39468 | 290 |  |  |
| -0.39337 | 378.3737 | 0.0006 | -0.39365 | 291 |  |  |
| -0.3923 | 379.4418 | 0.000627 | -0.39264 | 292 |  |  |
| -0.39124 | 380.5099 | 0.000654 | -0.39145 | 293 |  |  |
| -0.39017 | 381.5781 | 0.000683 | -0.39047 | 294 |  |  |
| -0.3891 | 382.6462 | 0.000713 | -0.38937 | 295 |  |  |
| -0.38803 | 383.7143 | 0.000745 | -0.38828 | 296 |  |  |
| -0.38696 | 384.7824 | 0.000777 | -0.38724 | 297 |  |  |
| -0.38589 | 385.8505 | 0.000812 | -0.38623 | 298 |  |  |
| -0.38483 | 386.9186 | 0.000848 | -0.3851 | 299 |  |  |
| -0.38376 | 387.9867 | 0.000885 | -0.384 | 300 |  |  |
| -0.38269 | 389.0549 | 0.000924 | -0.383 | 301 |  |  |
| -0.38162 | 390.123 | 0.000965 | -0.3819 | 302 |  |  |
| -0.38055 | 391.1911 | 0.001009 | -0.38083 | 303 |  |  |
| -0.37949 | 392.2592 | 0.001053 | -0.37991 | 304 |  |  |
| -0.37842 | 393.3273 | 0.0011 | -0.37872 | 305 |  |  |
| -0.37735 | 394.3954 | 0.001149 | -0.37769 | 306 |  |  |
| -0.37628 | 395.4636 | 0.0012 | -0.37659 | 307 |  |  |
| -0.37521 | 396.5317 | 0.001254 | -0.37555 | 308 |  |  |
| -0.37415 | 397.5998 | 0.00131 | -0.37454 | 309 |  |  |
| -0.37308 | 398.6679 | 0.00137 | -0.37341 | 310 |  |  |
| -0.37201 | 399.736 | 0.001431 | -0.37234 | 311 |  |  |
| -0.37094 | 400.8041 | 0.001495 | -0.37125 | 312 |  |  |
| -0.36987 | 401.8722 | 0.001563 | -0.37027 | 313 |  |  |
| -0.3688 | 402.9404 | 0.001634 | -0.36917 | 314 |  |  |
| -0.36774 | 404.0085 | 0.001709 | -0.36804 | 315 |  |  |
| -0.36667 | 405.0766 | 0.001787 | -0.3671 | 316 |  |  |
| -0.3656 | 406.1447 | 0.00187 | -0.36594 | 317 |  |  |
| -0.36453 | 407.2128 | 0.001956 | -0.36496 | 318 |  |  |
| -0.36346 | 408.2809 | 0.002048 | -0.36383 | 319 |  |  |
| -0.3624 | 409.349 | 0.002144 | -0.36279 | 320 |  |  |
| -0.36133 | 410.4172 | 0.002245 | -0.36179 | 321 |  |  |
| -0.36026 | 411.4853 | 0.002351 | -0.36066 | 322 |  |  |
| -0.35919 | 412.5534 | 0.00246 | -0.35962 | 323 |  |  |
| -0.35812 | 413.6215 | 0.002574 | -0.35855 | 324 |  |  |
| -0.35706 | 414.6896 | 0.002693 | -0.3576 | 325 |  |  |
| -0.35599 | 415.7577 | 0.002819 | -0.35645 | 326 |  |  |
| -0.35492 | 416.8259 | 0.002951 | -0.35553 | 327 |  |  |
| -0.35385 | 417.894 | 0.003089 | -0.35443 | 328 |  |  |
| -0.35278 | 418.9621 | 0.003235 | -0.35342 | 329 |  |  |
| -0.35172 | 420.0302 | 0.003384 | -0.35239 | 330 |  |  |
| -0.35065 | 421.0983 | 0.003542 | -0.35132 | 331 |  |  |
| -0.34958 | 422.1664 | 0.003703 | -0.35031 | 332 |  |  |
| -0.34851 | 423.2345 | 0.003865 | -0.34924 | 333 |  |  |
| -0.34744 | 424.3027 | 0.004026 | -0.34824 | 334 |  |  |
| -0.34637 | 425.3708 | 0.00418 | -0.34717 | 335 |  |  |
| -0.34531 | 426.4389 | 0.004322 | -0.3461 | 336 |  |  |
| -0.34424 | 427.507 | 0.004453 | -0.34515 | 337 |  |  |
| -0.34317 | 428.5751 | 0.004572 | -0.34409 | 338 |  |  |
| -0.3421 | 429.6432 | 0.004679 | -0.34299 | 339 |  |  |
| -0.34103 | 430.7113 | 0.004777 | -0.34204 | 340 |  |  |
| -0.33997 | 431.7795 | 0.004867 | -0.34094 | 341 |  |  |
| -0.3389 | 432.8476 | 0.00495 | -0.33987 | 342 |  |  |
| -0.33783 | 433.9157 | 0.005027 | -0.33884 | 343 |  |  |
| -0.33676 | 434.9838 | 0.005099 | -0.33777 | 344 |  |  |
| -0.33569 | 436.0519 | 0.005168 | -0.33673 | 345 |  |  |
| -0.33463 | 437.12 | 0.005233 | -0.3356 | 346 |  |  |
| -0.33356 | 438.1882 | 0.005297 | -0.33456 | 347 |  |  |
| -0.33249 | 439.2563 | 0.005357 | -0.33353 | 348 |  |  |
| -0.33142 | 440.3244 | 0.005415 | -0.33252 | 349 |  |  |
| -0.33035 | 441.3925 | 0.005471 | -0.33148 | 350 |  |  |
| -0.32928 | 442.4606 | 0.005527 | -0.33035 | 351 |  |  |
| -0.32822 | 443.5287 | 0.005581 | -0.32938 | 352 |  |  |
| -0.32715 | 444.5968 | 0.005633 | -0.32825 | 353 |  |  |
| -0.32608 | 445.665 | 0.005685 | -0.32721 | 354 |  |  |
| -0.32501 | 446.7331 | 0.005735 | -0.32617 | 355 |  |  |
| -0.32394 | 447.8012 | 0.005786 | -0.32507 | 356 |  |  |
| -0.32288 | 448.8693 | 0.005835 | -0.32407 | 357 |  |  |
| -0.32181 | 449.9374 | 0.005883 | -0.323 | 358 |  |  |
| -0.32074 | 451.0055 | 0.005932 | -0.32196 | 359 |  |  |
| -0.31967 | 452.0736 | 0.00598 | -0.32089 | 360 |  |  |
| -0.3186 | 453.1418 | 0.006028 | -0.31979 | 361 |  |  |
| -0.31754 | 454.2099 | 0.006075 | -0.31879 | 362 |  |  |
| -0.31647 | 455.278 | 0.006121 | -0.31769 | 363 |  |  |
| -0.3154 | 456.3461 | 0.006168 | -0.31668 | 364 |  |  |
| -0.31433 | 457.4142 | 0.006215 | -0.31561 | 365 |  |  |
| -0.31326 | 458.4823 | 0.006261 | -0.31458 | 366 |  |  |
| -0.31219 | 459.5505 | 0.006307 | -0.31345 | 367 |  |  |
| -0.31113 | 460.6186 | 0.006353 | -0.31241 | 368 |  |  |
| -0.31006 | 461.6867 | 0.006399 | -0.3114 | 369 |  |  |
| -0.30899 | 462.7548 | 0.006444 | -0.3103 | 370 |  |  |
| -0.30792 | 463.8229 | 0.006489 | -0.3093 | 371 |  |  |
| -0.30685 | 464.891 | 0.006534 | -0.3082 | 372 |  |  |
| -0.30579 | 465.9591 | 0.006578 | -0.30707 | 373 |  |  |
| -0.30472 | 467.0273 | 0.006623 | -0.30612 | 374 |  |  |
| -0.30365 | 468.0954 | 0.006666 | -0.30505 | 375 |  |  |
| -0.30258 | 469.1635 | 0.00671 | -0.30396 | 376 |  |  |
| -0.30151 | 470.2316 | 0.006753 | -0.30292 | 377 |  |  |
| -0.30045 | 471.2997 | 0.006795 | -0.30185 | 378 |  |  |
| -0.29938 | 472.3678 | 0.006836 | -0.30069 | 379 |  |  |
| -0.29831 | 473.4359 | 0.006877 | -0.29971 | 380 |  |  |
| -0.29724 | 474.5041 | 0.006916 | -0.29871 | 381 |  |  |
| -0.29617 | 475.5722 | 0.006954 | -0.29767 | 382 |  |  |
| -0.2951 | 476.6403 | 0.006992 | -0.2966 | 383 |  |  |
| -0.29404 | 477.7084 | 0.007029 | -0.29553 | 384 |  |  |
| -0.29297 | 478.7765 | 0.007064 | -0.29446 | 385 |  |  |
| -0.2919 | 479.8446 | 0.007099 | -0.29343 | 386 |  |  |
| -0.29083 | 480.9128 | 0.007132 | -0.29233 | 387 |  |  |
| -0.28976 | 481.9809 | 0.007165 | -0.29132 | 388 |  |  |
| -0.2887 | 483.049 | 0.007196 | -0.29022 | 389 |  |  |
| -0.28763 | 484.1171 | 0.007227 | -0.28918 | 390 |  |  |
| -0.28656 | 485.1852 | 0.007257 | -0.28802 | 391 |  |  |
| -0.28549 | 486.2533 | 0.007286 | -0.28699 | 392 |  |  |
| -0.28442 | 487.3214 | 0.007314 | -0.28601 | 393 |  |  |
| -0.28336 | 488.3896 | 0.007341 | -0.28497 | 394 |  |  |
| -0.28229 | 489.4577 | 0.007368 | -0.28378 | 395 |  |  |
| -0.28122 | 490.5258 | 0.007394 | -0.28278 | 396 |  |  |
| -0.28015 | 491.5939 | 0.007419 | -0.28177 | 397 |  |  |
| -0.27908 | 492.662 | 0.007444 | -0.28067 | 398 |  |  |
| -0.27802 | 493.7301 | 0.007468 | -0.27966 | 399 |  |  |
| -0.27695 | 494.7982 | 0.007491 | -0.2785 | 400 |  |  |
| -0.27588 | 495.8664 | 0.007514 | -0.2775 | 401 |  |  |
| -0.27481 | 496.9345 | 0.007536 | -0.27637 | 402 |  |  |
| -0.27374 | 498.0026 | 0.007557 | -0.27536 | 403 |  |  |
| -0.27267 | 499.0707 | 0.007578 | -0.27432 | 404 |  |  |
| -0.27161 | 500.1388 | 0.007597 | -0.27332 | 405 |  |  |
| -0.27054 | 501.2069 | 0.007617 | -0.27219 | 406 |  |  |
| -0.26947 | 502.2751 | 0.007636 | -0.27106 | 407 |  |  |
| -0.2684 | 503.3432 | 0.007655 | -0.26999 | 408 |  |  |
| -0.26733 | 504.4113 | 0.007673 | -0.26892 | 409 |  |  |
| -0.26627 | 505.4794 | 0.007691 | -0.26797 | 410 |  |  |
| -0.2652 | 506.5475 | 0.007709 | -0.26675 | 411 |  |  |
| -0.26413 | 507.6156 | 0.007726 | -0.26572 | 412 |  |  |
| -0.26306 | 508.6837 | 0.007743 | -0.26471 | 413 |  |  |
| -0.26199 | 509.7519 | 0.007758 | -0.26355 | 414 |  |  |
| -0.26093 | 510.82 | 0.007774 | -0.26254 | 415 |  |  |
| -0.25986 | 511.8881 | 0.00779 | -0.26157 | 416 |  |  |
| -0.25879 | 512.9562 | 0.007805 | -0.26041 | 417 |  |  |
| -0.25772 | 514.0243 | 0.00782 | -0.25934 | 418 |  |  |
| -0.25665 | 515.0924 | 0.007835 | -0.25833 | 419 |  |  |
| -0.25558 | 516.1605 | 0.007849 | -0.25732 | 420 |  |  |
| -0.25452 | 517.2287 | 0.007863 | -0.25629 | 421 |  |  |
| -0.25345 | 518.2968 | 0.007876 | -0.25513 | 422 |  |  |
| -0.25238 | 519.3649 | 0.00789 | -0.25412 | 423 |  |  |
| -0.25131 | 520.433 | 0.007905 | -0.25299 | 424 |  |  |
| -0.25024 | 521.5011 | 0.007917 | -0.25189 | 425 |  |  |
| -0.24918 | 522.5692 | 0.007929 | -0.25089 | 426 |  |  |
| -0.24811 | 523.6374 | 0.007944 | -0.24985 | 427 |  |  |
| -0.24704 | 524.7055 | 0.007957 | -0.24875 | 428 |  |  |
| -0.24597 | 525.7736 | 0.007969 | -0.24774 | 429 |  |  |
| -0.2449 | 526.8417 | 0.007981 | -0.24658 | 430 |  |  |
| -0.24384 | 527.9098 | 0.007993 | -0.24554 | 431 |  |  |
| -0.24277 | 528.9779 | 0.008005 | -0.24454 | 432 |  |  |
| -0.2417 | 530.046 | 0.008017 | -0.24347 | 433 |  |  |
| -0.24063 | 531.1142 | 0.008028 | -0.24237 | 434 |  |  |
| -0.23956 | 532.1823 | 0.008039 | -0.24133 | 435 |  |  |
| -0.23849 | 533.2504 | 0.008049 | -0.24023 | 436 |  |  |
| -0.23743 | 534.3185 | 0.008061 | -0.23923 | 437 |  |  |
| -0.23636 | 535.3866 | 0.00807 | -0.23813 | 438 |  |  |
| -0.23529 | 536.4547 | 0.008081 | -0.23703 | 439 |  |  |
| -0.23422 | 537.5228 | 0.008091 | -0.23599 | 440 |  |  |
| -0.23315 | 538.591 | 0.008102 | -0.23495 | 441 |  |  |
| -0.23209 | 539.6591 | 0.008112 | -0.23386 | 442 |  |  |
| -0.23102 | 540.7272 | 0.008122 | -0.23282 | 443 |  |  |
| -0.22995 | 541.7953 | 0.008132 | -0.23181 | 444 |  |  |
| -0.22888 | 542.8634 | 0.008144 | -0.23065 | 445 |  |  |
| -0.22781 | 543.9315 | 0.008152 | -0.22961 | 446 |  |  |
| -0.22675 | 544.9997 | 0.008163 | -0.22852 | 447 |  |  |
| -0.22568 | 546.0678 | 0.008172 | -0.22751 | 448 |  |  |
| -0.22461 | 547.1359 | 0.008181 | -0.22638 | 449 |  |  |
| -0.22354 | 548.204 | 0.008189 | -0.22537 | 450 |  |  |
| -0.22247 | 549.2721 | 0.0082 | -0.22424 | 451 |  |  |
| -0.22141 | 550.3402 | 0.008209 | -0.22318 | 452 |  |  |
| -0.22034 | 551.4083 | 0.008217 | -0.22208 | 453 |  |  |
| -0.21927 | 552.4765 | 0.008226 | -0.2211 | 454 |  |  |
| -0.2182 | 553.5446 | 0.008235 | -0.22006 | 455 |  |  |
| -0.21713 | 554.6127 | 0.008242 | -0.21899 | 456 |  |  |
| -0.21606 | 555.6808 | 0.008251 | -0.2178 | 457 |  |  |
| -0.215 | 556.7489 | 0.008261 | -0.21683 | 458 |  |  |
| -0.21393 | 557.817 | 0.00827 | -0.21573 | 459 |  |  |
| -0.21286 | 558.8851 | 0.008279 | -0.21466 | 460 |  |  |
| -0.21179 | 559.9533 | 0.008287 | -0.21362 | 461 |  |  |
| -0.21072 | 561.0214 | 0.008296 | -0.21255 | 462 |  |  |
| -0.20966 | 562.0895 | 0.008306 | -0.21146 | 463 |  |  |
| -0.20859 | 563.1576 | 0.008313 | -0.21045 | 464 |  |  |
| -0.20752 | 564.2257 | 0.008321 | -0.20935 | 465 |  |  |
| -0.20645 | 565.2938 | 0.00833 | -0.20831 | 466 |  |  |
| -0.20538 | 566.362 | 0.008336 | -0.20721 | 467 |  |  |
| -0.20432 | 567.4301 | 0.008344 | -0.20609 | 468 |  |  |
| -0.20325 | 568.4982 | 0.00835 | -0.20505 | 469 |  |  |

Sample: *Ribes Rubrum*, Concentration (ppm): 600, Immersion time: 24h

| Potential applied(V) | Time (s) | WE(1).  Current (A) | WE(1).  Potential (V) | Index |
| --- | --- | --- | --- | --- |
| -0.66116 | 68.63736 | -9.3E-05 | -0.65936 | 1 |
| -0.6601 | 69.70547 | -0.00093 | -0.65814 | 2 |
| -0.65903 | 70.77359 | -0.00622 | -0.65787 | 3 |
| -0.65796 | 71.8417 | -0.00619 | -0.6568 | 4 |
| -0.65689 | 72.90982 | -0.00615 | -0.65573 | 5 |
| -0.65582 | 73.97793 | -0.00612 | -0.65457 | 6 |
| -0.65475 | 75.04605 | -0.00609 | -0.65356 | 7 |
| -0.65369 | 76.11416 | -0.00606 | -0.65256 | 8 |
| -0.65262 | 77.18228 | -0.00603 | -0.65143 | 9 |
| -0.65155 | 78.25039 | -0.006 | -0.6503 | 10 |
| -0.65048 | 79.31851 | -0.00597 | -0.64941 | 11 |
| -0.64941 | 80.38662 | -0.00594 | -0.64835 | 12 |
| -0.64835 | 81.45474 | -0.00591 | -0.64722 | 13 |
| -0.64728 | 82.52285 | -0.00589 | -0.64618 | 14 |
| -0.64621 | 83.59097 | -0.00586 | -0.6452 | 15 |
| -0.64514 | 84.65908 | -0.00583 | -0.6441 | 16 |
| -0.64407 | 85.7272 | -0.0058 | -0.64301 | 17 |
| -0.64301 | 86.79531 | -0.00577 | -0.64191 | 18 |
| -0.64194 | 87.86343 | -0.00574 | -0.64096 | 19 |
| -0.64087 | 88.93154 | -0.00572 | -0.63983 | 20 |
| -0.6398 | 89.99966 | -0.00569 | -0.63882 | 21 |
| -0.63873 | 91.06777 | -0.00566 | -0.63776 | 22 |
| -0.63766 | 92.13589 | -0.00563 | -0.63672 | 23 |
| -0.6366 | 93.204 | -0.0056 | -0.63559 | 24 |
| -0.63553 | 94.27212 | -0.00557 | -0.63446 | 25 |
| -0.63446 | 95.34023 | -0.00554 | -0.63339 | 26 |
| -0.63339 | 96.40835 | -0.00551 | -0.63245 | 27 |
| -0.63232 | 97.47646 | -0.00547 | -0.63135 | 28 |
| -0.63126 | 98.54458 | -0.00544 | -0.63031 | 29 |
| -0.63019 | 99.61269 | -0.00541 | -0.62921 | 30 |
| -0.62912 | 100.6808 | -0.00537 | -0.62817 | 31 |
| -0.62805 | 101.7489 | -0.00534 | -0.62723 | 32 |
| -0.62698 | 102.817 | -0.0053 | -0.6261 | 33 |
| -0.62592 | 103.8852 | -0.00527 | -0.62509 | 34 |
| -0.62485 | 104.9533 | -0.00523 | -0.62399 | 35 |
| -0.62378 | 106.0214 | -0.00519 | -0.62289 | 36 |
| -0.62271 | 107.0895 | -0.00515 | -0.62183 | 37 |
| -0.62164 | 108.1576 | -0.00511 | -0.62073 | 38 |
| -0.62057 | 109.2257 | -0.00507 | -0.61978 | 39 |
| -0.61951 | 110.2938 | -0.00502 | -0.61853 | 40 |
| -0.61844 | 111.362 | -0.00498 | -0.61771 | 41 |
| -0.61737 | 112.4301 | -0.00493 | -0.61658 | 42 |
| -0.6163 | 113.4982 | -0.00488 | -0.61554 | 43 |
| -0.61523 | 114.5663 | -0.00483 | -0.6145 | 44 |
| -0.61417 | 115.6344 | -0.00477 | -0.61331 | 45 |
| -0.6131 | 116.7025 | -0.00471 | -0.6123 | 46 |
| -0.61203 | 117.7706 | -0.00465 | -0.61136 | 47 |
| -0.61096 | 118.8388 | -0.00458 | -0.6102 | 48 |
| -0.60989 | 119.9069 | -0.00451 | -0.60907 | 49 |
| -0.60883 | 120.975 | -0.00443 | -0.60818 | 50 |
| -0.60776 | 122.0431 | -0.00435 | -0.60718 | 51 |
| -0.60669 | 123.1112 | -0.00426 | -0.60614 | 52 |
| -0.60562 | 124.1793 | -0.00417 | -0.60495 | 53 |
| -0.60455 | 125.2475 | -0.00408 | -0.60397 | 54 |
| -0.60349 | 126.3156 | -0.00398 | -0.60287 | 55 |
| -0.60242 | 127.3837 | -0.00389 | -0.60181 | 56 |
| -0.60135 | 128.4518 | -0.00379 | -0.60077 | 57 |
| -0.60028 | 129.5199 | -0.00369 | -0.59976 | 58 |
| -0.59921 | 130.588 | -0.00359 | -0.59885 | 59 |
| -0.59814 | 131.6561 | -0.00349 | -0.59766 | 60 |
| -0.59708 | 132.7243 | -0.0034 | -0.59653 | 61 |
| -0.59601 | 133.7924 | -0.00331 | -0.59549 | 62 |
| -0.59494 | 134.8605 | -0.00322 | -0.59457 | 63 |
| -0.59387 | 135.9286 | -0.00313 | -0.59351 | 64 |
| -0.5928 | 136.9967 | -0.00304 | -0.59238 | 65 |
| -0.59174 | 138.0648 | -0.00296 | -0.59146 | 66 |
| -0.59067 | 139.1329 | -0.00288 | -0.5903 | 67 |
| -0.5896 | 140.2011 | -0.0028 | -0.58926 | 68 |
| -0.58853 | 141.2692 | -0.00272 | -0.58826 | 69 |
| -0.58746 | 142.3373 | -0.00265 | -0.58716 | 70 |
| -0.5864 | 143.4054 | -0.00258 | -0.5863 | 71 |
| -0.58533 | 144.4735 | -0.00251 | -0.58521 | 72 |
| -0.58426 | 145.5416 | -0.00244 | -0.58405 | 73 |
| -0.58319 | 146.6098 | -0.00237 | -0.58298 | 74 |
| -0.58212 | 147.6779 | -0.00231 | -0.58194 | 75 |
| -0.58105 | 148.746 | -0.00225 | -0.58093 | 76 |
| -0.57999 | 149.8141 | -0.00219 | -0.57983 | 77 |
| -0.57892 | 150.8822 | -0.00213 | -0.57886 | 78 |
| -0.57785 | 151.9503 | -0.00207 | -0.57773 | 79 |
| -0.57678 | 153.0184 | -0.00201 | -0.57669 | 80 |
| -0.57571 | 154.0866 | -0.00196 | -0.57562 | 81 |
| -0.57465 | 155.1547 | -0.00191 | -0.57465 | 82 |
| -0.57358 | 156.2228 | -0.00185 | -0.57358 | 83 |
| -0.57251 | 157.2909 | -0.0018 | -0.57242 | 84 |
| -0.57144 | 158.359 | -0.00176 | -0.57138 | 85 |
| -0.57037 | 159.4271 | -0.00171 | -0.57034 | 86 |
| -0.56931 | 160.4952 | -0.00166 | -0.56921 | 87 |
| -0.56824 | 161.5634 | -0.00162 | -0.56818 | 88 |
| -0.56717 | 162.6315 | -0.00158 | -0.5672 | 89 |
| -0.5661 | 163.6996 | -0.00153 | -0.56607 | 90 |
| -0.56503 | 164.7677 | -0.00149 | -0.565 | 91 |
| -0.56396 | 165.8358 | -0.00145 | -0.56393 | 92 |
| -0.5629 | 166.9039 | -0.00141 | -0.56293 | 93 |
| -0.56183 | 167.9721 | -0.00138 | -0.56192 | 94 |
| -0.56076 | 169.0402 | -0.00134 | -0.56076 | 95 |
| -0.55969 | 170.1083 | -0.00131 | -0.55966 | 96 |
| -0.55862 | 171.1764 | -0.00127 | -0.55875 | 97 |
| -0.55756 | 172.2445 | -0.00124 | -0.55759 | 98 |
| -0.55649 | 173.3126 | -0.0012 | -0.55652 | 99 |
| -0.55542 | 174.3807 | -0.00117 | -0.55548 | 100 |
| -0.55435 | 175.4489 | -0.00114 | -0.5545 | 101 |
| -0.55328 | 176.517 | -0.00111 | -0.55325 | 102 |
| -0.55222 | 177.5851 | -0.00108 | -0.55231 | 103 |
| -0.55115 | 178.6532 | -0.00105 | -0.55112 | 104 |
| -0.55008 | 179.7213 | -0.00103 | -0.55014 | 105 |
| -0.54901 | 180.7894 | -0.001 | -0.54913 | 106 |
| -0.54794 | 181.8575 | -0.00097 | -0.548 | 107 |
| -0.54688 | 182.9257 | -0.00095 | -0.54703 | 108 |
| -0.54581 | 183.9938 | -0.00092 | -0.54593 | 109 |
| -0.54474 | 185.0619 | -0.0009 | -0.5448 | 110 |
| -0.54367 | 186.13 | -0.00088 | -0.54385 | 111 |
| -0.5426 | 187.1981 | -0.00085 | -0.54272 | 112 |
| -0.54153 | 188.2662 | -0.00083 | -0.5416 | 113 |
| -0.54047 | 189.3344 | -0.00081 | -0.54053 | 114 |
| -0.5394 | 190.4025 | -0.00079 | -0.53946 | 115 |
| -0.53833 | 191.4706 | -0.00077 | -0.53851 | 116 |
| -0.53726 | 192.5387 | -0.00075 | -0.53738 | 117 |
| -0.53619 | 193.6068 | -0.00073 | -0.53619 | 118 |
| -0.53513 | 194.6749 | -0.00071 | -0.53531 | 119 |
| -0.53406 | 195.743 | -0.00069 | -0.53415 | 120 |
| -0.53299 | 196.8112 | -0.00068 | -0.53305 | 121 |
| -0.53192 | 197.8793 | -0.00066 | -0.53207 | 122 |
| -0.53085 | 198.9474 | -0.00064 | -0.53094 | 123 |
| -0.52979 | 200.0155 | -0.00063 | -0.53009 | 124 |
| -0.52872 | 201.0836 | -0.00061 | -0.5289 | 125 |
| -0.52765 | 202.1517 | -0.0006 | -0.52786 | 126 |
| -0.52658 | 203.2198 | -0.00058 | -0.52673 | 127 |
| -0.52551 | 204.288 | -0.00057 | -0.52563 | 128 |
| -0.52444 | 205.3561 | -0.00055 | -0.52454 | 129 |
| -0.52338 | 206.4242 | -0.00054 | -0.52359 | 130 |
| -0.52231 | 207.4923 | -0.00052 | -0.52249 | 131 |
| -0.52124 | 208.5604 | -0.00051 | -0.52136 | 132 |
| -0.52017 | 209.6285 | -0.0005 | -0.52036 | 133 |
| -0.5191 | 210.6967 | -0.00049 | -0.51929 | 134 |
| -0.51804 | 211.7648 | -0.00047 | -0.51828 | 135 |
| -0.51697 | 212.8329 | -0.00046 | -0.51706 | 136 |
| -0.5159 | 213.901 | -0.00045 | -0.51602 | 137 |
| -0.51483 | 214.9691 | -0.00044 | -0.51495 | 138 |
| -0.51376 | 216.0372 | -0.00043 | -0.51392 | 139 |
| -0.5127 | 217.1053 | -0.00042 | -0.51294 | 140 |
| -0.51163 | 218.1735 | -0.00041 | -0.51175 | 141 |
| -0.51056 | 219.2416 | -0.0004 | -0.51065 | 142 |
| -0.50949 | 220.3097 | -0.00039 | -0.50955 | 143 |
| -0.50842 | 221.3778 | -0.00038 | -0.50858 | 144 |
| -0.50735 | 222.4459 | -0.00037 | -0.50748 | 145 |
| -0.50629 | 223.514 | -0.00036 | -0.50641 | 146 |
| -0.50522 | 224.5821 | -0.00035 | -0.50534 | 147 |
| -0.50415 | 225.6503 | -0.00034 | -0.5043 | 148 |
| -0.50308 | 226.7184 | -0.00034 | -0.5032 | 149 |
| -0.50201 | 227.7865 | -0.00033 | -0.50217 | 150 |
| -0.50095 | 228.8546 | -0.00032 | -0.50107 | 151 |
| -0.49988 | 229.9227 | -0.00031 | -0.5 | 152 |
| -0.49881 | 230.9908 | -0.00031 | -0.49902 | 153 |
| -0.49774 | 232.059 | -0.0003 | -0.49792 | 154 |
| -0.49667 | 233.1271 | -0.00029 | -0.49686 | 155 |
| -0.49561 | 234.1952 | -0.00028 | -0.49576 | 156 |
| -0.49454 | 235.2633 | -0.00028 | -0.49475 | 157 |
| -0.49347 | 236.3314 | -0.00027 | -0.49359 | 158 |
| -0.4924 | 237.3995 | -0.00027 | -0.49246 | 159 |
| -0.49133 | 238.4676 | -0.00026 | -0.49158 | 160 |
| -0.49026 | 239.5358 | -0.00025 | -0.49039 | 161 |
| -0.4892 | 240.6039 | -0.00025 | -0.48923 | 162 |
| -0.48813 | 241.672 | -0.00024 | -0.48834 | 163 |
| -0.48706 | 242.7401 | -0.00024 | -0.48712 | 164 |
| -0.48599 | 243.8082 | -0.00023 | -0.48611 | 165 |
| -0.48492 | 244.8763 | -0.00023 | -0.48511 | 166 |
| -0.48386 | 245.9444 | -0.00022 | -0.48404 | 167 |
| -0.48279 | 247.0126 | -0.00022 | -0.48285 | 168 |
| -0.48172 | 248.0807 | -0.00021 | -0.48181 | 169 |
| -0.48065 | 249.1488 | -0.00021 | -0.4809 | 170 |
| -0.47958 | 250.2169 | -0.0002 | -0.47971 | 171 |
| -0.47852 | 251.285 | -0.0002 | -0.47873 | 172 |
| -0.47745 | 252.3531 | -0.00019 | -0.47757 | 173 |
| -0.47638 | 253.4213 | -0.00019 | -0.47659 | 174 |
| -0.47531 | 254.4894 | -0.00018 | -0.4754 | 175 |
| -0.47424 | 255.5575 | -0.00018 | -0.47449 | 176 |
| -0.47318 | 256.6256 | -0.00018 | -0.47327 | 177 |
| -0.47211 | 257.6937 | -0.00017 | -0.47235 | 178 |
| -0.47104 | 258.7618 | -0.00017 | -0.47125 | 179 |
| -0.46997 | 259.8299 | -0.00016 | -0.47012 | 180 |
| -0.4689 | 260.8981 | -0.00016 | -0.46906 | 181 |
| -0.46783 | 261.9662 | -0.00016 | -0.46808 | 182 |
| -0.46677 | 263.0343 | -0.00015 | -0.46692 | 183 |
| -0.4657 | 264.1024 | -0.00015 | -0.46582 | 184 |
| -0.46463 | 265.1705 | -0.00015 | -0.46472 | 185 |
| -0.46356 | 266.2386 | -0.00014 | -0.46378 | 186 |
| -0.46249 | 267.3067 | -0.00014 | -0.46265 | 187 |
| -0.46143 | 268.3749 | -0.00014 | -0.46155 | 188 |
| -0.46036 | 269.443 | -0.00013 | -0.46057 | 189 |
| -0.45929 | 270.5111 | -0.00013 | -0.45941 | 190 |
| -0.45822 | 271.5792 | -0.00013 | -0.45837 | 191 |
| -0.45715 | 272.6473 | -0.00012 | -0.45731 | 192 |
| -0.45609 | 273.7154 | -0.00012 | -0.45615 | 193 |
| -0.45502 | 274.7836 | -0.00012 | -0.45502 | 194 |
| -0.45395 | 275.8517 | -0.00012 | -0.45407 | 195 |
| -0.45288 | 276.9198 | -0.00011 | -0.45303 | 196 |
| -0.45181 | 277.9879 | -0.00011 | -0.452 | 197 |
| -0.45074 | 279.056 | -0.00011 | -0.45087 | 198 |
| -0.44968 | 280.1241 | -0.00011 | -0.44983 | 199 |
| -0.44861 | 281.1922 | -0.0001 | -0.44891 | 200 |
| -0.44754 | 282.2604 | -0.0001 | -0.44769 | 201 |
| -0.44647 | 283.3285 | -9.9E-05 | -0.44669 | 202 |
| -0.4454 | 284.3966 | -9.7E-05 | -0.44559 | 203 |
| -0.44434 | 285.4647 | -9.4E-05 | -0.44443 | 204 |
| -0.44327 | 286.5328 | -9.2E-05 | -0.44348 | 205 |
| -0.4422 | 287.6009 | -9E-05 | -0.44235 | 206 |
| -0.44113 | 288.669 | -8.8E-05 | -0.44107 | 207 |
| -0.44006 | 289.7372 | -8.5E-05 | -0.44016 | 208 |
| -0.439 | 290.8053 | -8.3E-05 | -0.43893 | 209 |
| -0.43793 | 291.8734 | -8.1E-05 | -0.43799 | 210 |
| -0.43686 | 292.9415 | -7.9E-05 | -0.43695 | 211 |
| -0.43579 | 294.0096 | -7.7E-05 | -0.43588 | 212 |
| -0.43472 | 295.0777 | -7.5E-05 | -0.43481 | 213 |
| -0.43365 | 296.1459 | -7.3E-05 | -0.43384 | 214 |
| -0.43259 | 297.214 | -7.1E-05 | -0.43268 | 215 |
| -0.43152 | 298.2821 | -6.9E-05 | -0.4317 | 216 |
| -0.43045 | 299.3502 | -6.6E-05 | -0.43057 | 217 |
| -0.42938 | 300.4183 | -6.5E-05 | -0.42944 | 218 |
| -0.42831 | 301.4864 | -6.2E-05 | -0.42834 | 219 |
| -0.42725 | 302.5545 | -6E-05 | -0.42731 | 220 |
| -0.42618 | 303.6227 | -5.8E-05 | -0.4263 | 221 |
| -0.42511 | 304.6908 | -5.6E-05 | -0.4252 | 222 |
| -0.42404 | 305.7589 | -5.4E-05 | -0.42413 | 223 |
| -0.42297 | 306.827 | -5.2E-05 | -0.42307 | 224 |
| -0.42191 | 307.8951 | -5E-05 | -0.42206 | 225 |
| -0.42084 | 308.9632 | -4.8E-05 | -0.42099 | 226 |
| -0.41977 | 310.0313 | -4.6E-05 | -0.41983 | 227 |
| -0.4187 | 311.0995 | -4.4E-05 | -0.41876 | 228 |
| -0.41763 | 312.1676 | -4.1E-05 | -0.41772 | 229 |
| -0.41656 | 313.2357 | -3.9E-05 | -0.41663 | 230 |
| -0.4155 | 314.3038 | -3.7E-05 | -0.41562 | 231 |
| -0.41443 | 315.3719 | -3.5E-05 | -0.41452 | 232 |
| -0.41336 | 316.44 | -3.3E-05 | -0.41342 | 233 |
| -0.41229 | 317.5082 | -3E-05 | -0.41235 | 234 |
| -0.41122 | 318.5763 | -2.9E-05 | -0.41147 | 235 |
| -0.41016 | 319.6444 | -2.6E-05 | -0.41037 | 236 |
| -0.40909 | 320.7125 | -2.4E-05 | -0.40918 | 237 |
| -0.40802 | 321.7806 | -2.1E-05 | -0.4082 | 238 |
| -0.40695 | 322.8487 | -1.9E-05 | -0.4072 | 239 |
| -0.40588 | 323.9168 | -1.8E-05 | -0.40604 | 240 |
| -0.40482 | 324.985 | -1.5E-05 | -0.40506 | 241 |
| -0.40375 | 326.0531 | -1.3E-05 | -0.40384 | 242 |
| -0.40268 | 327.1212 | -9.9E-06 | -0.40286 | 243 |
| -0.40161 | 328.1893 | -7.4E-06 | -0.40179 | 244 |
| -0.40054 | 329.2574 | -4.9E-06 | -0.40051 | 245 |
| -0.39948 | 330.3255 | -1.8E-06 | -0.39957 | 246 |
| -0.39841 | 331.3936 | 8.42E-07 | -0.39883 | 247 |
| -0.39734 | 332.4618 | 4E-06 | -0.39774 | 248 |
| -0.39627 | 333.5299 | 6.98E-06 | -0.39658 | 249 |
| -0.3952 | 334.598 | 9.68E-06 | -0.39517 | 250 |
| -0.39413 | 335.6661 | 1.3E-05 | -0.3941 | 251 |
| -0.39307 | 336.7342 | 1.6E-05 | -0.39359 | 252 |
| -0.392 | 337.8023 | 1.99E-05 | -0.39246 | 253 |
| -0.39093 | 338.8705 | 2.3E-05 | -0.39102 | 254 |
| -0.38986 | 339.9386 | 2.69E-05 | -0.39154 | 255 |
| -0.38879 | 341.0067 | 3.07E-05 | -0.3894 | 256 |
| -0.38773 | 342.0748 | 3.43E-05 | -0.38797 | 257 |
| -0.38666 | 343.1429 | 3.82E-05 | -0.38696 | 258 |
| -0.38559 | 344.211 | 4.33E-05 | -0.38586 | 259 |
| -0.38452 | 345.2791 | 4.77E-05 | -0.38492 | 260 |
| -0.38345 | 346.3473 | 5.22E-05 | -0.38364 | 261 |
| -0.38239 | 347.4154 | 5.64E-05 | -0.38266 | 262 |
| -0.38132 | 348.4835 | 6.18E-05 | -0.38184 | 263 |
| -0.38025 | 349.5516 | 6.69E-05 | -0.38043 | 264 |
| -0.37918 | 350.6197 | 7.23E-05 | -0.37936 | 265 |
| -0.37811 | 351.6878 | 7.82E-05 | -0.37842 | 266 |
| -0.37704 | 352.7559 | 8.44E-05 | -0.37726 | 267 |
| -0.37598 | 353.8241 | 9.07E-05 | -0.37637 | 268 |
| -0.37491 | 354.8922 | 9.73E-05 | -0.37518 | 269 |
| -0.37384 | 355.9603 | 0.000104 | -0.37411 | 270 |
| -0.37277 | 357.0284 | 0.000111 | -0.37311 | 271 |
| -0.3717 | 358.0965 | 0.00012 | -0.37207 | 272 |
| -0.37064 | 359.1646 | 0.000128 | -0.37094 | 273 |
| -0.36957 | 360.2328 | 0.000136 | -0.36996 | 274 |
| -0.3685 | 361.3009 | 0.000145 | -0.36896 | 275 |
| -0.36743 | 362.369 | 0.000154 | -0.36783 | 276 |
| -0.36636 | 363.4371 | 0.000164 | -0.36682 | 277 |
| -0.3653 | 364.5052 | 0.000174 | -0.36566 | 278 |
| -0.36423 | 365.5733 | 0.000185 | -0.3645 | 279 |
| -0.36316 | 366.6414 | 0.000197 | -0.36353 | 280 |
| -0.36209 | 367.7096 | 0.000209 | -0.36249 | 281 |
| -0.36102 | 368.7777 | 0.000222 | -0.36148 | 282 |
| -0.35995 | 369.8458 | 0.000235 | -0.36041 | 283 |
| -0.35889 | 370.9139 | 0.00025 | -0.35922 | 284 |
| -0.35782 | 371.982 | 0.000265 | -0.35806 | 285 |
| -0.35675 | 373.0501 | 0.000281 | -0.3573 | 286 |
| -0.35568 | 374.1182 | 0.000299 | -0.35632 | 287 |
| -0.35461 | 375.1864 | 0.000317 | -0.35513 | 288 |
| -0.35355 | 376.2545 | 0.000337 | -0.35376 | 289 |
| -0.35248 | 377.3226 | 0.000357 | -0.35272 | 290 |
| -0.35141 | 378.3907 | 0.000379 | -0.35159 | 291 |
| -0.35034 | 379.4588 | 0.000402 | -0.35049 | 292 |
| -0.34927 | 380.5269 | 0.000426 | -0.34943 | 293 |
| -0.34821 | 381.5951 | 0.000452 | -0.34839 | 294 |
| -0.34714 | 382.6632 | 0.000479 | -0.34732 | 295 |
| -0.34607 | 383.7313 | 0.000508 | -0.34619 | 296 |
| -0.345 | 384.7994 | 0.000539 | -0.34521 | 297 |
| -0.34393 | 385.8675 | 0.000571 | -0.34418 | 298 |
| -0.34286 | 386.9356 | 0.000605 | -0.34302 | 299 |
| -0.3418 | 388.0037 | 0.000641 | -0.34201 | 300 |
| -0.34073 | 389.0719 | 0.00068 | -0.34097 | 301 |
| -0.33966 | 390.14 | 0.000722 | -0.33994 | 302 |
| -0.33859 | 391.2081 | 0.000766 | -0.33884 | 303 |
| -0.33752 | 392.2762 | 0.000812 | -0.33774 | 304 |
| -0.33646 | 393.3443 | 0.000861 | -0.33673 | 305 |
| -0.33539 | 394.4124 | 0.000913 | -0.33563 | 306 |
| -0.33432 | 395.4805 | 0.000969 | -0.33459 | 307 |
| -0.33325 | 396.5487 | 0.001027 | -0.33353 | 308 |
| -0.33218 | 397.6168 | 0.00109 | -0.33246 | 309 |
| -0.33112 | 398.6849 | 0.001156 | -0.33136 | 310 |
| -0.33005 | 399.753 | 0.001227 | -0.33035 | 311 |
| -0.32898 | 400.8211 | 0.001302 | -0.32916 | 312 |
| -0.32791 | 401.8892 | 0.001384 | -0.32831 | 313 |
| -0.32684 | 402.9574 | 0.001472 | -0.32709 | 314 |
| -0.32578 | 404.0255 | 0.001566 | -0.32608 | 315 |
| -0.32471 | 405.0936 | 0.001667 | -0.3251 | 316 |
| -0.32364 | 406.1617 | 0.001775 | -0.32401 | 317 |
| -0.32257 | 407.2298 | 0.001889 | -0.32306 | 318 |
| -0.3215 | 408.2979 | 0.00201 | -0.32193 | 319 |
| -0.32043 | 409.366 | 0.002143 | -0.32083 | 320 |
| -0.31937 | 410.4342 | 0.002289 | -0.31973 | 321 |
| -0.3183 | 411.5023 | 0.002447 | -0.31882 | 322 |
| -0.31723 | 412.5704 | 0.002626 | -0.31775 | 323 |
| -0.31616 | 413.6385 | 0.002823 | -0.31677 | 324 |
| -0.31509 | 414.7066 | 0.003031 | -0.3157 | 325 |
| -0.31403 | 415.7747 | 0.003256 | -0.31467 | 326 |
| -0.31296 | 416.8428 | 0.003499 | -0.31357 | 327 |
| -0.31189 | 417.911 | 0.003765 | -0.31256 | 328 |
| -0.31082 | 418.9791 | 0.00405 | -0.31158 | 329 |
| -0.30975 | 420.0472 | 0.004303 | -0.31058 | 330 |
| -0.30869 | 421.1153 | 0.004515 | -0.30951 | 331 |
| -0.30762 | 422.1834 | 0.004699 | -0.30841 | 332 |
| -0.30655 | 423.2515 | 0.004856 | -0.3075 | 333 |
| -0.30548 | 424.3197 | 0.004993 | -0.30643 | 334 |
| -0.30441 | 425.3878 | 0.005117 | -0.30539 | 335 |
| -0.30334 | 426.4559 | 0.00523 | -0.30441 | 336 |
| -0.30228 | 427.524 | 0.005335 | -0.30331 | 337 |
| -0.30121 | 428.5921 | 0.005434 | -0.30222 | 338 |
| -0.30014 | 429.6602 | 0.005526 | -0.30121 | 339 |
| -0.29907 | 430.7283 | 0.005615 | -0.30014 | 340 |
| -0.298 | 431.7965 | 0.005699 | -0.29916 | 341 |
| -0.29694 | 432.8646 | 0.005781 | -0.2981 | 342 |
| -0.29587 | 433.9327 | 0.005859 | -0.29712 | 343 |
| -0.2948 | 435.0008 | 0.005933 | -0.29593 | 344 |
| -0.29373 | 436.0689 | 0.006005 | -0.29495 | 345 |
| -0.29266 | 437.137 | 0.006073 | -0.29388 | 346 |
| -0.2916 | 438.2051 | 0.006139 | -0.29291 | 347 |
| -0.29053 | 439.2733 | 0.006204 | -0.29172 | 348 |
| -0.28946 | 440.3414 | 0.006266 | -0.2908 | 349 |
| -0.28839 | 441.4095 | 0.006325 | -0.28973 | 350 |
| -0.28732 | 442.4776 | 0.006381 | -0.28864 | 351 |
| -0.28625 | 443.5457 | 0.006436 | -0.2876 | 352 |
| -0.28519 | 444.6138 | 0.006488 | -0.2865 | 353 |
| -0.28412 | 445.682 | 0.006539 | -0.28549 | 354 |
| -0.28305 | 446.7501 | 0.006588 | -0.28442 | 355 |
| -0.28198 | 447.8182 | 0.006635 | -0.28345 | 356 |
| -0.28091 | 448.8863 | 0.00668 | -0.28226 | 357 |
| -0.27985 | 449.9544 | 0.006723 | -0.28131 | 358 |
| -0.27878 | 451.0225 | 0.006765 | -0.2803 | 359 |
| -0.27771 | 452.0906 | 0.006805 | -0.27917 | 360 |
| -0.27664 | 453.1588 | 0.006843 | -0.27808 | 361 |
| -0.27557 | 454.2269 | 0.00688 | -0.27707 | 362 |
| -0.27451 | 455.295 | 0.006916 | -0.27597 | 363 |
| -0.27344 | 456.3631 | 0.00695 | -0.2749 | 364 |
| -0.27237 | 457.4312 | 0.006983 | -0.2739 | 365 |
| -0.2713 | 458.4993 | 0.007015 | -0.27277 | 366 |
| -0.27023 | 459.5674 | 0.007046 | -0.27161 | 367 |
| -0.26917 | 460.6356 | 0.007075 | -0.27066 | 368 |
| -0.2681 | 461.7037 | 0.007104 | -0.2695 | 369 |
| -0.26703 | 462.7718 | 0.007132 | -0.26852 | 370 |
| -0.26596 | 463.8399 | 0.007159 | -0.26743 | 371 |
| -0.26489 | 464.908 | 0.007186 | -0.26642 | 372 |
| -0.26382 | 465.9761 | 0.007212 | -0.26544 | 373 |
| -0.26276 | 467.0443 | 0.007238 | -0.26419 | 374 |
| -0.26169 | 468.1124 | 0.007262 | -0.26324 | 375 |
| -0.26062 | 469.1805 | 0.007287 | -0.26221 | 376 |
| -0.25955 | 470.2486 | 0.007311 | -0.26114 | 377 |
| -0.25848 | 471.3167 | 0.007335 | -0.26001 | 378 |
| -0.25742 | 472.3848 | 0.007358 | -0.259 | 379 |
| -0.25635 | 473.4529 | 0.007381 | -0.2579 | 380 |
| -0.25528 | 474.5211 | 0.007404 | -0.25687 | 381 |
| -0.25421 | 475.5892 | 0.007426 | -0.25583 | 382 |
| -0.25314 | 476.6573 | 0.007448 | -0.2547 | 383 |
| -0.25208 | 477.7254 | 0.00747 | -0.25375 | 384 |
| -0.25101 | 478.7935 | 0.007492 | -0.25256 | 385 |
| -0.24994 | 479.8616 | 0.007514 | -0.25153 | 386 |
| -0.24887 | 480.9297 | 0.007535 | -0.25046 | 387 |
| -0.2478 | 481.9979 | 0.007557 | -0.24951 | 388 |
| -0.24673 | 483.066 | 0.007578 | -0.24841 | 389 |
| -0.24567 | 484.1341 | 0.007599 | -0.24725 | 390 |
| -0.2446 | 485.2022 | 0.007619 | -0.24622 | 391 |
| -0.24353 | 486.2703 | 0.007639 | -0.24524 | 392 |
| -0.24246 | 487.3384 | 0.007659 | -0.24414 | 393 |
| -0.24139 | 488.4066 | 0.007679 | -0.24307 | 394 |
| -0.24033 | 489.4747 | 0.007699 | -0.24197 | 395 |
| -0.23926 | 490.5428 | 0.007719 | -0.24094 | 396 |
| -0.23819 | 491.6109 | 0.007738 | -0.23987 | 397 |
| -0.23712 | 492.679 | 0.007758 | -0.23889 | 398 |
| -0.23605 | 493.7471 | 0.007776 | -0.23782 | 399 |
| -0.23499 | 494.8152 | 0.007796 | -0.23679 | 400 |
| -0.23392 | 495.8834 | 0.007815 | -0.23572 | 401 |
| -0.23285 | 496.9515 | 0.007834 | -0.23459 | 402 |
| -0.23178 | 498.0196 | 0.007852 | -0.23349 | 403 |
| -0.23071 | 499.0877 | 0.007871 | -0.23239 | 404 |
| -0.22964 | 500.1558 | 0.007889 | -0.23132 | 405 |
| -0.22858 | 501.2239 | 0.007907 | -0.23029 | 406 |
| -0.22751 | 502.292 | 0.007926 | -0.22931 | 407 |
| -0.22644 | 503.3602 | 0.007944 | -0.22812 | 408 |
| -0.22537 | 504.4283 | 0.007962 | -0.22711 | 409 |
| -0.2243 | 505.4964 | 0.007979 | -0.22607 | 410 |
| -0.22324 | 506.5645 | 0.007997 | -0.22501 | 411 |
| -0.22217 | 507.6326 | 0.008014 | -0.22388 | 412 |
| -0.2211 | 508.7007 | 0.008031 | -0.22299 | 413 |
| -0.22003 | 509.7689 | 0.008047 | -0.22174 | 414 |
| -0.21896 | 510.837 | 0.008064 | -0.22073 | 415 |
| -0.2179 | 511.9051 | 0.00808 | -0.21976 | 416 |
| -0.21683 | 512.9732 | 0.008096 | -0.21863 | 417 |
| -0.21576 | 514.0413 | 0.008111 | -0.21753 | 418 |
| -0.21469 | 515.1094 | 0.008127 | -0.21652 | 419 |
| -0.21362 | 516.1775 | 0.008142 | -0.21548 | 420 |
| -0.21255 | 517.2457 | 0.008157 | -0.21451 | 421 |
| -0.21149 | 518.3138 | 0.008173 | -0.21338 | 422 |
| -0.21042 | 519.3819 | 0.008188 | -0.21225 | 423 |
| -0.20935 | 520.45 | 0.008203 | -0.21124 | 424 |
| -0.20828 | 521.5181 | 0.008217 | -0.21005 | 425 |
| -0.20721 | 522.5862 | 0.00823 | -0.20901 | 426 |
| -0.20615 | 523.6543 | 0.008243 | -0.20798 | 427 |
| -0.20508 | 524.7225 | 0.008256 | -0.20688 | 428 |
| -0.20401 | 525.7906 | 0.00827 | -0.20578 | 429 |
| -0.20294 | 526.8587 | 0.008284 | -0.20483 | 430 |
| -0.20187 | 527.9268 | 0.008297 | -0.20361 | 431 |
| -0.20081 | 528.9949 | 0.00831 | -0.2027 | 432 |
| -0.19974 | 530.063 | 0.008322 | -0.20154 | 433 |
| -0.19867 | 531.1312 | 0.008334 | -0.2005 | 434 |
| -0.1976 | 532.1993 | 0.008347 | -0.19943 | 435 |
| -0.19653 | 533.2674 | 0.008359 | -0.1983 | 436 |
| -0.19547 | 534.3355 | 0.00837 | -0.19736 | 437 |
| -0.1944 | 535.4036 | 0.008383 | -0.19626 | 438 |
| -0.19333 | 536.4717 | 0.008394 | -0.19519 | 439 |
| -0.19226 | 537.5398 | 0.008404 | -0.19406 | 440 |
| -0.19119 | 538.608 | 0.008414 | -0.19305 | 441 |
| -0.19012 | 539.6761 | 0.008424 | -0.19189 | 442 |
| -0.18906 | 540.7442 | 0.008434 | -0.19095 | 443 |
| -0.18799 | 541.8123 | 0.008446 | -0.18991 | 444 |
| -0.18692 | 542.8804 | 0.008457 | -0.18893 | 445 |
| -0.18585 | 543.9485 | 0.008467 | -0.18771 | 446 |
| -0.18478 | 545.0166 | 0.008477 | -0.18671 | 447 |
| -0.18372 | 546.0848 | 0.008488 | -0.18558 | 448 |
| -0.18265 | 547.1529 | 0.008499 | -0.18457 | 449 |
| -0.18158 | 548.221 | 0.008509 | -0.18347 | 450 |
| -0.18051 | 549.2891 | 0.008518 | -0.18246 | 451 |
| -0.17944 | 550.3572 | 0.008527 | -0.1814 | 452 |
| -0.17838 | 551.4253 | 0.008536 | -0.1803 | 453 |
| -0.17731 | 552.4935 | 0.008546 | -0.17914 | 454 |
| -0.17624 | 553.5616 | 0.008556 | -0.17816 | 455 |
| -0.17517 | 554.6297 | 0.008566 | -0.17703 | 456 |
| -0.1741 | 555.6978 | 0.008576 | -0.17609 | 457 |
| -0.17303 | 556.7659 | 0.008584 | -0.17493 | 458 |
| -0.17197 | 557.834 | 0.008593 | -0.17395 | 459 |
| -0.1709 | 558.9021 | 0.008602 | -0.17288 | 460 |
| -0.16983 | 559.9703 | 0.008611 | -0.17172 | 461 |
| -0.16876 | 561.0384 | 0.00862 | -0.17068 | 462 |
| -0.16769 | 562.1065 | 0.008629 | -0.16968 | 463 |
| -0.16663 | 563.1746 | 0.008638 | -0.16861 | 464 |
| -0.16556 | 564.2427 | 0.008647 | -0.16757 | 465 |
| -0.16449 | 565.3108 | 0.008657 | -0.16644 | 466 |
| -0.16342 | 566.3789 | 0.008664 | -0.16541 | 467 |
| -0.16235 | 567.4471 | 0.008673 | -0.16431 | 468 |
|  |  |  |  |  |

Sample: *Ribes Rubrum*, Concentration (ppm): 800, Immersion time: 24h

| Potential applied(V) | Time (s) | WE(1).  Current (A) | WE(1).  Potential (V) | Index |
| --- | --- | --- | --- | --- |
| -0.66406 | 68.73037 | -9.3E-05 | -0.66208 | 1 |
| -0.66299 | 69.79849 | -0.00093 | -0.66098 | 2 |
| -0.66193 | 70.8666 | -0.00626 | -0.66074 | 3 |
| -0.66086 | 71.93472 | -0.00623 | -0.65952 | 4 |
| -0.65979 | 73.00283 | -0.00619 | -0.65851 | 5 |
| -0.65872 | 74.07095 | -0.00616 | -0.65744 | 6 |
| -0.65765 | 75.13906 | -0.00614 | -0.65649 | 7 |
| -0.65659 | 76.20718 | -0.00611 | -0.65543 | 8 |
| -0.65552 | 77.27529 | -0.00608 | -0.6543 | 9 |
| -0.65445 | 78.34341 | -0.00605 | -0.65311 | 10 |
| -0.65338 | 79.41152 | -0.00603 | -0.65219 | 11 |
| -0.65231 | 80.47964 | -0.006 | -0.65109 | 12 |
| -0.65125 | 81.54775 | -0.00597 | -0.64993 | 13 |
| -0.65018 | 82.61587 | -0.00595 | -0.64905 | 14 |
| -0.64911 | 83.68398 | -0.00592 | -0.64792 | 15 |
| -0.64804 | 84.7521 | -0.00589 | -0.64688 | 16 |
| -0.64697 | 85.82021 | -0.00587 | -0.64578 | 17 |
| -0.6459 | 86.88833 | -0.00584 | -0.64465 | 18 |
| -0.64484 | 87.95644 | -0.00582 | -0.64374 | 19 |
| -0.64377 | 89.02456 | -0.00579 | -0.64264 | 20 |
| -0.6427 | 90.09267 | -0.00576 | -0.6416 | 21 |
| -0.64163 | 91.16079 | -0.00574 | -0.64047 | 22 |
| -0.64056 | 92.2289 | -0.00571 | -0.63953 | 23 |
| -0.6395 | 93.29702 | -0.00568 | -0.63855 | 24 |
| -0.63843 | 94.36513 | -0.00566 | -0.6373 | 25 |
| -0.63736 | 95.43325 | -0.00563 | -0.63629 | 26 |
| -0.63629 | 96.50136 | -0.0056 | -0.63525 | 27 |
| -0.63522 | 97.56948 | -0.00557 | -0.63416 | 28 |
| -0.63416 | 98.63759 | -0.00555 | -0.63293 | 29 |
| -0.63309 | 99.70571 | -0.00552 | -0.63199 | 30 |
| -0.63202 | 100.7738 | -0.00549 | -0.63101 | 31 |
| -0.63095 | 101.8419 | -0.00546 | -0.62985 | 32 |
| -0.62988 | 102.9101 | -0.00543 | -0.62885 | 33 |
| -0.62881 | 103.9782 | -0.0054 | -0.62781 | 34 |
| -0.62775 | 105.0463 | -0.00537 | -0.62668 | 35 |
| -0.62668 | 106.1144 | -0.00534 | -0.62564 | 36 |
| -0.62561 | 107.1825 | -0.00531 | -0.62457 | 37 |
| -0.62454 | 108.2506 | -0.00527 | -0.62354 | 38 |
| -0.62347 | 109.3187 | -0.00524 | -0.62247 | 39 |
| -0.62241 | 110.3869 | -0.00521 | -0.62143 | 40 |
| -0.62134 | 111.455 | -0.00517 | -0.62036 | 41 |
| -0.62027 | 112.5231 | -0.00514 | -0.61932 | 42 |
| -0.6192 | 113.5912 | -0.0051 | -0.61832 | 43 |
| -0.61813 | 114.6593 | -0.00506 | -0.61713 | 44 |
| -0.61707 | 115.7274 | -0.00502 | -0.61606 | 45 |
| -0.616 | 116.7955 | -0.00498 | -0.61502 | 46 |
| -0.61493 | 117.8637 | -0.00494 | -0.61401 | 47 |
| -0.61386 | 118.9318 | -0.00489 | -0.61298 | 48 |
| -0.61279 | 119.9999 | -0.00485 | -0.61191 | 49 |
| -0.61172 | 121.068 | -0.0048 | -0.61084 | 50 |
| -0.61066 | 122.1361 | -0.00475 | -0.60986 | 51 |
| -0.60959 | 123.2042 | -0.00469 | -0.6087 | 52 |
| -0.60852 | 124.2724 | -0.00463 | -0.60767 | 53 |
| -0.60745 | 125.3405 | -0.00457 | -0.60651 | 54 |
| -0.60638 | 126.4086 | -0.00451 | -0.60559 | 55 |
| -0.60532 | 127.4767 | -0.00444 | -0.60468 | 56 |
| -0.60425 | 128.5448 | -0.00437 | -0.60349 | 57 |
| -0.60318 | 129.6129 | -0.00429 | -0.60239 | 58 |
| -0.60211 | 130.681 | -0.00421 | -0.60129 | 59 |
| -0.60104 | 131.7492 | -0.00413 | -0.60028 | 60 |
| -0.59998 | 132.8173 | -0.00404 | -0.59924 | 61 |
| -0.59891 | 133.8854 | -0.00395 | -0.59818 | 62 |
| -0.59784 | 134.9535 | -0.00385 | -0.59723 | 63 |
| -0.59677 | 136.0216 | -0.00376 | -0.59613 | 64 |
| -0.5957 | 137.0897 | -0.00366 | -0.59515 | 65 |
| -0.59464 | 138.1578 | -0.00357 | -0.59399 | 66 |
| -0.59357 | 139.226 | -0.00348 | -0.59311 | 67 |
| -0.5925 | 140.2941 | -0.00338 | -0.59201 | 68 |
| -0.59143 | 141.3622 | -0.00329 | -0.591 | 69 |
| -0.59036 | 142.4303 | -0.00321 | -0.58981 | 70 |
| -0.58929 | 143.4984 | -0.00312 | -0.58893 | 71 |
| -0.58823 | 144.5665 | -0.00304 | -0.58774 | 72 |
| -0.58716 | 145.6347 | -0.00295 | -0.58667 | 73 |
| -0.58609 | 146.7028 | -0.00287 | -0.58566 | 74 |
| -0.58502 | 147.7709 | -0.0028 | -0.58469 | 75 |
| -0.58395 | 148.839 | -0.00272 | -0.58365 | 76 |
| -0.58289 | 149.9071 | -0.00265 | -0.58246 | 77 |
| -0.58182 | 150.9752 | -0.00258 | -0.58154 | 78 |
| -0.58075 | 152.0433 | -0.00251 | -0.58054 | 79 |
| -0.57968 | 153.1115 | -0.00244 | -0.57941 | 80 |
| -0.57861 | 154.1796 | -0.00237 | -0.57825 | 81 |
| -0.57755 | 155.2477 | -0.00231 | -0.57727 | 82 |
| -0.57648 | 156.3158 | -0.00225 | -0.57623 | 83 |
| -0.57541 | 157.3839 | -0.00219 | -0.5752 | 84 |
| -0.57434 | 158.452 | -0.00213 | -0.57416 | 85 |
| -0.57327 | 159.5201 | -0.00207 | -0.57297 | 86 |
| -0.5722 | 160.5883 | -0.00201 | -0.57211 | 87 |
| -0.57114 | 161.6564 | -0.00196 | -0.57086 | 88 |
| -0.57007 | 162.7245 | -0.00191 | -0.56992 | 89 |
| -0.569 | 163.7926 | -0.00186 | -0.56885 | 90 |
| -0.56793 | 164.8607 | -0.00181 | -0.56778 | 91 |
| -0.56686 | 165.9288 | -0.00176 | -0.56677 | 92 |
| -0.5658 | 166.997 | -0.00171 | -0.56561 | 93 |
| -0.56473 | 168.0651 | -0.00167 | -0.56464 | 94 |
| -0.56366 | 169.1332 | -0.00162 | -0.56357 | 95 |
| -0.56259 | 170.2013 | -0.00158 | -0.56259 | 96 |
| -0.56152 | 171.2694 | -0.00154 | -0.56128 | 97 |
| -0.56046 | 172.3375 | -0.00149 | -0.56033 | 98 |
| -0.55939 | 173.4056 | -0.00145 | -0.55933 | 99 |
| -0.55832 | 174.4738 | -0.00142 | -0.55826 | 100 |
| -0.55725 | 175.5419 | -0.00138 | -0.55722 | 101 |
| -0.55618 | 176.61 | -0.00134 | -0.55603 | 102 |
| -0.55511 | 177.6781 | -0.00131 | -0.55508 | 103 |
| -0.55405 | 178.7462 | -0.00127 | -0.55402 | 104 |
| -0.55298 | 179.8143 | -0.00124 | -0.55292 | 105 |
| -0.55191 | 180.8824 | -0.00121 | -0.55173 | 106 |
| -0.55084 | 181.9506 | -0.00117 | -0.55084 | 107 |
| -0.54977 | 183.0187 | -0.00114 | -0.54965 | 108 |
| -0.54871 | 184.0868 | -0.00111 | -0.54861 | 109 |
| -0.54764 | 185.1549 | -0.00108 | -0.54761 | 110 |
| -0.54657 | 186.223 | -0.00106 | -0.54657 | 111 |
| -0.5455 | 187.2911 | -0.00103 | -0.5455 | 112 |
| -0.54443 | 188.3593 | -0.001 | -0.5444 | 113 |
| -0.54337 | 189.4274 | -0.00098 | -0.54337 | 114 |
| -0.5423 | 190.4955 | -0.00095 | -0.5423 | 115 |
| -0.54123 | 191.5636 | -0.00093 | -0.54132 | 116 |
| -0.54016 | 192.6317 | -0.0009 | -0.54022 | 117 |
| -0.53909 | 193.6998 | -0.00088 | -0.53903 | 118 |
| -0.53802 | 194.7679 | -0.00086 | -0.53806 | 119 |
| -0.53696 | 195.8361 | -0.00083 | -0.53696 | 120 |
| -0.53589 | 196.9042 | -0.00081 | -0.53589 | 121 |
| -0.53482 | 197.9723 | -0.00079 | -0.53479 | 122 |
| -0.53375 | 199.0404 | -0.00077 | -0.53378 | 123 |
| -0.53268 | 200.1085 | -0.00075 | -0.53275 | 124 |
| -0.53162 | 201.1766 | -0.00073 | -0.53177 | 125 |
| -0.53055 | 202.2447 | -0.00071 | -0.53052 | 126 |
| -0.52948 | 203.3129 | -0.00069 | -0.52951 | 127 |
| -0.52841 | 204.381 | -0.00068 | -0.52841 | 128 |
| -0.52734 | 205.4491 | -0.00066 | -0.5274 | 129 |
| -0.52628 | 206.5172 | -0.00064 | -0.52634 | 130 |
| -0.52521 | 207.5853 | -0.00063 | -0.52524 | 131 |
| -0.52414 | 208.6534 | -0.00061 | -0.52423 | 132 |
| -0.52307 | 209.7216 | -0.0006 | -0.52322 | 133 |
| -0.522 | 210.7897 | -0.00058 | -0.52194 | 134 |
| -0.52094 | 211.8578 | -0.00057 | -0.52097 | 135 |
| -0.51987 | 212.9259 | -0.00055 | -0.51996 | 136 |
| -0.5188 | 213.994 | -0.00054 | -0.51871 | 137 |
| -0.51773 | 215.0621 | -0.00052 | -0.51773 | 138 |
| -0.51666 | 216.1302 | -0.00051 | -0.51675 | 139 |
| -0.51559 | 217.1984 | -0.0005 | -0.51553 | 140 |
| -0.51453 | 218.2665 | -0.00049 | -0.51468 | 141 |
| -0.51346 | 219.3346 | -0.00047 | -0.51343 | 142 |
| -0.51239 | 220.4027 | -0.00046 | -0.51242 | 143 |
| -0.51132 | 221.4708 | -0.00045 | -0.51154 | 144 |
| -0.51025 | 222.5389 | -0.00044 | -0.51038 | 145 |
| -0.50919 | 223.607 | -0.00043 | -0.50919 | 146 |
| -0.50812 | 224.6752 | -0.00042 | -0.50815 | 147 |
| -0.50705 | 225.7433 | -0.00041 | -0.5072 | 148 |
| -0.50598 | 226.8114 | -0.0004 | -0.50595 | 149 |
| -0.50491 | 227.8795 | -0.00039 | -0.505 | 150 |
| -0.50385 | 228.9476 | -0.00038 | -0.50388 | 151 |
| -0.50278 | 230.0157 | -0.00037 | -0.50281 | 152 |
| -0.50171 | 231.0839 | -0.00036 | -0.50171 | 153 |
| -0.50064 | 232.152 | -0.00035 | -0.50064 | 154 |
| -0.49957 | 233.2201 | -0.00034 | -0.4996 | 155 |
| -0.4985 | 234.2882 | -0.00033 | -0.4986 | 156 |
| -0.49744 | 235.3563 | -0.00033 | -0.49756 | 157 |
| -0.49637 | 236.4244 | -0.00032 | -0.49652 | 158 |
| -0.4953 | 237.4925 | -0.00031 | -0.49539 | 159 |
| -0.49423 | 238.5607 | -0.0003 | -0.49426 | 160 |
| -0.49316 | 239.6288 | -0.0003 | -0.4931 | 161 |
| -0.4921 | 240.6969 | -0.00029 | -0.49222 | 162 |
| -0.49103 | 241.765 | -0.00028 | -0.49106 | 163 |
| -0.48996 | 242.8331 | -0.00028 | -0.49017 | 164 |
| -0.48889 | 243.9012 | -0.00027 | -0.48898 | 165 |
| -0.48782 | 244.9693 | -0.00026 | -0.48785 | 166 |
| -0.48676 | 246.0375 | -0.00026 | -0.48685 | 167 |
| -0.48569 | 247.1056 | -0.00025 | -0.48566 | 168 |
| -0.48462 | 248.1737 | -0.00024 | -0.48468 | 169 |
| -0.48355 | 249.2418 | -0.00024 | -0.48364 | 170 |
| -0.48248 | 250.3099 | -0.00023 | -0.48251 | 171 |
| -0.48141 | 251.378 | -0.00023 | -0.48157 | 172 |
| -0.48035 | 252.4462 | -0.00022 | -0.48041 | 173 |
| -0.47928 | 253.5143 | -0.00022 | -0.4794 | 174 |
| -0.47821 | 254.5824 | -0.00021 | -0.47836 | 175 |
| -0.47714 | 255.6505 | -0.00021 | -0.47726 | 176 |
| -0.47607 | 256.7186 | -0.0002 | -0.4762 | 177 |
| -0.47501 | 257.7867 | -0.0002 | -0.47519 | 178 |
| -0.47394 | 258.8548 | -0.00019 | -0.47403 | 179 |
| -0.47287 | 259.923 | -0.00019 | -0.47293 | 180 |
| -0.4718 | 260.9911 | -0.00018 | -0.47195 | 181 |
| -0.47073 | 262.0592 | -0.00018 | -0.47083 | 182 |
| -0.46967 | 263.1273 | -0.00017 | -0.4697 | 183 |
| -0.4686 | 264.1954 | -0.00017 | -0.4686 | 184 |
| -0.46753 | 265.2635 | -0.00017 | -0.46768 | 185 |
| -0.46646 | 266.3316 | -0.00016 | -0.46658 | 186 |
| -0.46539 | 267.3998 | -0.00016 | -0.46548 | 187 |
| -0.46432 | 268.4679 | -0.00015 | -0.46439 | 188 |
| -0.46326 | 269.536 | -0.00015 | -0.46341 | 189 |
| -0.46219 | 270.6041 | -0.00015 | -0.46228 | 190 |
| -0.46112 | 271.6722 | -0.00014 | -0.46118 | 191 |
| -0.46005 | 272.7403 | -0.00014 | -0.46017 | 192 |
| -0.45898 | 273.8085 | -0.00014 | -0.45911 | 193 |
| -0.45792 | 274.8766 | -0.00013 | -0.4581 | 194 |
| -0.45685 | 275.9447 | -0.00013 | -0.45688 | 195 |
| -0.45578 | 277.0128 | -0.00013 | -0.45578 | 196 |
| -0.45471 | 278.0809 | -0.00012 | -0.45493 | 197 |
| -0.45364 | 279.149 | -0.00012 | -0.45374 | 198 |
| -0.45258 | 280.2171 | -0.00012 | -0.45264 | 199 |
| -0.45151 | 281.2853 | -0.00011 | -0.45163 | 200 |
| -0.45044 | 282.3534 | -0.00011 | -0.4505 | 201 |
| -0.44937 | 283.4215 | -0.00011 | -0.44949 | 202 |
| -0.4483 | 284.4896 | -0.00011 | -0.44855 | 203 |
| -0.44724 | 285.5577 | -0.0001 | -0.44742 | 204 |
| -0.44617 | 286.6258 | -0.0001 | -0.44626 | 205 |
| -0.4451 | 287.6939 | -9.7E-05 | -0.44513 | 206 |
| -0.44403 | 288.7621 | -9.5E-05 | -0.44403 | 207 |
| -0.44296 | 289.8302 | -9.2E-05 | -0.44302 | 208 |
| -0.44189 | 290.8983 | -9E-05 | -0.44193 | 209 |
| -0.44083 | 291.9664 | -8.7E-05 | -0.44092 | 210 |
| -0.43976 | 293.0345 | -8.5E-05 | -0.43973 | 211 |
| -0.43869 | 294.1026 | -8.2E-05 | -0.43851 | 212 |
| -0.43762 | 295.1708 | -7.9E-05 | -0.43762 | 213 |
| -0.43655 | 296.2389 | -7.8E-05 | -0.43652 | 214 |
| -0.43549 | 297.307 | -7.5E-05 | -0.43552 | 215 |
| -0.43442 | 298.3751 | -7.3E-05 | -0.43433 | 216 |
| -0.43335 | 299.4432 | -7E-05 | -0.43332 | 217 |
| -0.43228 | 300.5113 | -6.8E-05 | -0.43234 | 218 |
| -0.43121 | 301.5794 | -6.6E-05 | -0.43121 | 219 |
| -0.43015 | 302.6476 | -6.3E-05 | -0.43011 | 220 |
| -0.42908 | 303.7157 | -6.1E-05 | -0.42926 | 221 |
| -0.42801 | 304.7838 | -5.9E-05 | -0.42804 | 222 |
| -0.42694 | 305.8519 | -5.7E-05 | -0.42694 | 223 |
| -0.42587 | 306.92 | -5.4E-05 | -0.42593 | 224 |
| -0.4248 | 307.9881 | -5.2E-05 | -0.42487 | 225 |
| -0.42374 | 309.0562 | -5E-05 | -0.42386 | 226 |
| -0.42267 | 310.1244 | -4.8E-05 | -0.42264 | 227 |
| -0.4216 | 311.1925 | -4.6E-05 | -0.42157 | 228 |
| -0.42053 | 312.2606 | -4.3E-05 | -0.42068 | 229 |
| -0.41946 | 313.3287 | -4E-05 | -0.4194 | 230 |
| -0.4184 | 314.3968 | -3.8E-05 | -0.41843 | 231 |
| -0.41733 | 315.4649 | -3.6E-05 | -0.41721 | 232 |
| -0.41626 | 316.5331 | -3.3E-05 | -0.41644 | 233 |
| -0.41519 | 317.6012 | -3.1E-05 | -0.41516 | 234 |
| -0.41412 | 318.6693 | -2.8E-05 | -0.41428 | 235 |
| -0.41306 | 319.7374 | -2.6E-05 | -0.4129 | 236 |
| -0.41199 | 320.8055 | -2.3E-05 | -0.41211 | 237 |
| -0.41092 | 321.8736 | -2.1E-05 | -0.41107 | 238 |
| -0.40985 | 322.9417 | -1.9E-05 | -0.40997 | 239 |
| -0.40878 | 324.0099 | -1.6E-05 | -0.40897 | 240 |
| -0.40771 | 325.078 | -1.4E-05 | -0.40778 | 241 |
| -0.40665 | 326.1461 | -1.1E-05 | -0.40668 | 242 |
| -0.40558 | 327.2142 | -8E-06 | -0.40558 | 243 |
| -0.40451 | 328.2823 | -5.8E-06 | -0.40454 | 244 |
| -0.40344 | 329.3504 | -2.9E-06 | -0.40347 | 245 |
| -0.40237 | 330.4185 | 4.58E-07 | -0.40247 | 246 |
| -0.40131 | 331.4867 | 2.84E-06 | -0.40137 | 247 |
| -0.40024 | 332.5548 | 6.24E-06 | -0.3999 | 248 |
| -0.39917 | 333.6229 | 9.29E-06 | -0.3992 | 249 |
| -0.3981 | 334.691 | 1.26E-05 | -0.39822 | 250 |
| -0.39703 | 335.7591 | 1.61E-05 | -0.39673 | 251 |
| -0.39597 | 336.8272 | 1.91E-05 | -0.39645 | 252 |
| -0.3949 | 337.8954 | 2.28E-05 | -0.39511 | 253 |
| -0.39383 | 338.9635 | 2.66E-05 | -0.3942 | 254 |
| -0.39276 | 340.0316 | 3.02E-05 | -0.39291 | 255 |
| -0.39169 | 341.0997 | 3.46E-05 | -0.39197 | 256 |
| -0.39063 | 342.1678 | 3.88E-05 | -0.39078 | 257 |
| -0.38956 | 343.2359 | 4.32E-05 | -0.38965 | 258 |
| -0.38849 | 344.304 | 4.82E-05 | -0.38855 | 259 |
| -0.38742 | 345.3722 | 5.27E-05 | -0.38773 | 260 |
| -0.38635 | 346.4403 | 5.73E-05 | -0.38638 | 261 |
| -0.38528 | 347.5084 | 6.25E-05 | -0.38553 | 262 |
| -0.38422 | 348.5765 | 6.78E-05 | -0.38449 | 263 |
| -0.38315 | 349.6446 | 7.33E-05 | -0.38354 | 264 |
| -0.38208 | 350.7127 | 7.9E-05 | -0.38242 | 265 |
| -0.38101 | 351.7808 | 8.51E-05 | -0.38126 | 266 |
| -0.37994 | 352.849 | 9.11E-05 | -0.38028 | 267 |
| -0.37888 | 353.9171 | 9.75E-05 | -0.37906 | 268 |
| -0.37781 | 354.9852 | 0.000104 | -0.37811 | 269 |
| -0.37674 | 356.0533 | 0.000112 | -0.37711 | 270 |
| -0.37567 | 357.1214 | 0.000119 | -0.37598 | 271 |
| -0.3746 | 358.1895 | 0.000127 | -0.37488 | 272 |
| -0.37354 | 359.2577 | 0.000136 | -0.37378 | 273 |
| -0.37247 | 360.3258 | 0.000145 | -0.37283 | 274 |
| -0.3714 | 361.3939 | 0.000154 | -0.37177 | 275 |
| -0.37033 | 362.462 | 0.000164 | -0.37067 | 276 |
| -0.36926 | 363.5301 | 0.000174 | -0.36966 | 277 |
| -0.36819 | 364.5982 | 0.000185 | -0.36862 | 278 |
| -0.36713 | 365.6663 | 0.000196 | -0.36746 | 279 |
| -0.36606 | 366.7345 | 0.000208 | -0.36642 | 280 |
| -0.36499 | 367.8026 | 0.00022 | -0.36545 | 281 |
| -0.36392 | 368.8707 | 0.000233 | -0.36432 | 282 |
| -0.36285 | 369.9388 | 0.000248 | -0.36331 | 283 |
| -0.36179 | 371.0069 | 0.000262 | -0.36221 | 284 |
| -0.36072 | 372.075 | 0.000278 | -0.36115 | 285 |
| -0.35965 | 373.1431 | 0.000294 | -0.36017 | 286 |
| -0.35858 | 374.2113 | 0.000311 | -0.35922 | 287 |
| -0.35751 | 375.2794 | 0.000329 | -0.35764 | 288 |
| -0.35645 | 376.3475 | 0.000348 | -0.35657 | 289 |
| -0.35538 | 377.4156 | 0.000368 | -0.35553 | 290 |
| -0.35431 | 378.4837 | 0.000389 | -0.35443 | 291 |
| -0.35324 | 379.5518 | 0.000412 | -0.35327 | 292 |
| -0.35217 | 380.62 | 0.000435 | -0.35242 | 293 |
| -0.3511 | 381.6881 | 0.00046 | -0.35126 | 294 |
| -0.35004 | 382.7562 | 0.000486 | -0.35016 | 295 |
| -0.34897 | 383.8243 | 0.000515 | -0.349 | 296 |
| -0.3479 | 384.8924 | 0.000545 | -0.34818 | 297 |
| -0.34683 | 385.9605 | 0.000576 | -0.34711 | 298 |
| -0.34576 | 387.0286 | 0.00061 | -0.34592 | 299 |
| -0.3447 | 388.0968 | 0.000645 | -0.34494 | 300 |
| -0.34363 | 389.1649 | 0.000681 | -0.34381 | 301 |
| -0.34256 | 390.233 | 0.000721 | -0.34274 | 302 |
| -0.34149 | 391.3011 | 0.000762 | -0.34174 | 303 |
| -0.34042 | 392.3692 | 0.000806 | -0.34064 | 304 |
| -0.33936 | 393.4373 | 0.000853 | -0.33948 | 305 |
| -0.33829 | 394.5054 | 0.000905 | -0.3385 | 306 |
| -0.33722 | 395.5736 | 0.000957 | -0.33746 | 307 |
| -0.33615 | 396.6417 | 0.001013 | -0.3364 | 308 |
| -0.33508 | 397.7098 | 0.001072 | -0.3353 | 309 |
| -0.33401 | 398.7779 | 0.001135 | -0.33426 | 310 |
| -0.33295 | 399.846 | 0.001203 | -0.33322 | 311 |
| -0.33188 | 400.9141 | 0.001275 | -0.33212 | 312 |
| -0.33081 | 401.9823 | 0.001353 | -0.33109 | 313 |
| -0.32974 | 403.0504 | 0.001435 | -0.32996 | 314 |
| -0.32867 | 404.1185 | 0.001525 | -0.32904 | 315 |
| -0.32761 | 405.1866 | 0.001621 | -0.32794 | 316 |
| -0.32654 | 406.2547 | 0.001724 | -0.32687 | 317 |
| -0.32547 | 407.3228 | 0.001832 | -0.32578 | 318 |
| -0.3244 | 408.3909 | 0.001951 | -0.32477 | 319 |
| -0.32333 | 409.4591 | 0.002079 | -0.32367 | 320 |
| -0.32227 | 410.5272 | 0.002219 | -0.32272 | 321 |
| -0.3212 | 411.5953 | 0.00237 | -0.32153 | 322 |
| -0.32013 | 412.6634 | 0.002534 | -0.32062 | 323 |
| -0.31906 | 413.7315 | 0.00271 | -0.31952 | 324 |
| -0.31799 | 414.7996 | 0.002905 | -0.31845 | 325 |
| -0.31693 | 415.8677 | 0.003117 | -0.31747 | 326 |
| -0.31586 | 416.9359 | 0.003346 | -0.31647 | 327 |
| -0.31479 | 418.004 | 0.003591 | -0.31549 | 328 |
| -0.31372 | 419.0721 | 0.003846 | -0.31439 | 329 |
| -0.31265 | 420.1402 | 0.0041 | -0.31339 | 330 |
| -0.31158 | 421.2083 | 0.004336 | -0.31244 | 331 |
| -0.31052 | 422.2764 | 0.004537 | -0.31143 | 332 |
| -0.30945 | 423.3446 | 0.004712 | -0.31033 | 333 |
| -0.30838 | 424.4127 | 0.004861 | -0.3092 | 334 |
| -0.30731 | 425.4808 | 0.004994 | -0.30826 | 335 |
| -0.30624 | 426.5489 | 0.005114 | -0.30722 | 336 |
| -0.30518 | 427.617 | 0.005224 | -0.30615 | 337 |
| -0.30411 | 428.6851 | 0.005326 | -0.30499 | 338 |
| -0.30304 | 429.7532 | 0.005421 | -0.30402 | 339 |
| -0.30197 | 430.8214 | 0.005511 | -0.30304 | 340 |
| -0.3009 | 431.8895 | 0.005596 | -0.302 | 341 |
| -0.29984 | 432.9576 | 0.005677 | -0.30084 | 342 |
| -0.29877 | 434.0257 | 0.005755 | -0.29984 | 343 |
| -0.2977 | 435.0938 | 0.005829 | -0.29886 | 344 |
| -0.29663 | 436.1619 | 0.005901 | -0.29776 | 345 |
| -0.29556 | 437.23 | 0.005969 | -0.29666 | 346 |
| -0.29449 | 438.2982 | 0.006034 | -0.29568 | 347 |
| -0.29343 | 439.3663 | 0.006096 | -0.29459 | 348 |
| -0.29236 | 440.4344 | 0.006156 | -0.29343 | 349 |
| -0.29129 | 441.5025 | 0.006213 | -0.29254 | 350 |
| -0.29022 | 442.5706 | 0.006268 | -0.29141 | 351 |
| -0.28915 | 443.6387 | 0.00632 | -0.29037 | 352 |
| -0.28809 | 444.7069 | 0.006371 | -0.2894 | 353 |
| -0.28702 | 445.775 | 0.006419 | -0.28827 | 354 |
| -0.28595 | 446.8431 | 0.006465 | -0.28726 | 355 |
| -0.28488 | 447.9112 | 0.00651 | -0.28616 | 356 |
| -0.28381 | 448.9793 | 0.006553 | -0.28516 | 357 |
| -0.28275 | 450.0474 | 0.006594 | -0.28415 | 358 |
| -0.28168 | 451.1155 | 0.006634 | -0.28302 | 359 |
| -0.28061 | 452.1837 | 0.006672 | -0.28195 | 360 |
| -0.27954 | 453.2518 | 0.006709 | -0.28085 | 361 |
| -0.27847 | 454.3199 | 0.006744 | -0.27985 | 362 |
| -0.2774 | 455.388 | 0.006779 | -0.27878 | 363 |
| -0.27634 | 456.4561 | 0.006812 | -0.27774 | 364 |
| -0.27527 | 457.5242 | 0.006844 | -0.27655 | 365 |
| -0.2742 | 458.5923 | 0.006875 | -0.27557 | 366 |
| -0.27313 | 459.6605 | 0.006906 | -0.27448 | 367 |
| -0.27206 | 460.7286 | 0.006935 | -0.27353 | 368 |
| -0.271 | 461.7967 | 0.006964 | -0.27237 | 369 |
| -0.26993 | 462.8648 | 0.006992 | -0.27124 | 370 |
| -0.26886 | 463.9329 | 0.007019 | -0.27029 | 371 |
| -0.26779 | 465.001 | 0.007046 | -0.26913 | 372 |
| -0.26672 | 466.0692 | 0.007072 | -0.26822 | 373 |
| -0.26566 | 467.1373 | 0.007097 | -0.26712 | 374 |
| -0.26459 | 468.2054 | 0.007122 | -0.26593 | 375 |
| -0.26352 | 469.2735 | 0.007147 | -0.26495 | 376 |
| -0.26245 | 470.3416 | 0.007171 | -0.26401 | 377 |
| -0.26138 | 471.4097 | 0.007194 | -0.26282 | 378 |
| -0.26031 | 472.4778 | 0.007217 | -0.26181 | 379 |
| -0.25925 | 473.546 | 0.00724 | -0.26077 | 380 |
| -0.25818 | 474.6141 | 0.007263 | -0.25967 | 381 |
| -0.25711 | 475.6822 | 0.007285 | -0.25867 | 382 |
| -0.25604 | 476.7503 | 0.007307 | -0.25751 | 383 |
| -0.25497 | 477.8184 | 0.007328 | -0.25656 | 384 |
| -0.25391 | 478.8865 | 0.007349 | -0.25546 | 385 |
| -0.25284 | 479.9546 | 0.007369 | -0.25433 | 386 |
| -0.25177 | 481.0228 | 0.007389 | -0.25327 | 387 |
| -0.2507 | 482.0909 | 0.007409 | -0.2522 | 388 |
| -0.24963 | 483.159 | 0.007429 | -0.25122 | 389 |
| -0.24857 | 484.2271 | 0.007448 | -0.25015 | 390 |
| -0.2475 | 485.2952 | 0.007468 | -0.24915 | 391 |
| -0.24643 | 486.3633 | 0.007487 | -0.24814 | 392 |
| -0.24536 | 487.4315 | 0.007505 | -0.24695 | 393 |
| -0.24429 | 488.4996 | 0.007524 | -0.24582 | 394 |
| -0.24323 | 489.5677 | 0.007543 | -0.24487 | 395 |
| -0.24216 | 490.6358 | 0.007561 | -0.24384 | 396 |
| -0.24109 | 491.7039 | 0.007579 | -0.24265 | 397 |
| -0.24002 | 492.772 | 0.007597 | -0.24167 | 398 |
| -0.23895 | 493.8401 | 0.007615 | -0.24042 | 399 |
| -0.23788 | 494.9083 | 0.007633 | -0.23941 | 400 |
| -0.23682 | 495.9764 | 0.007651 | -0.2384 | 401 |
| -0.23575 | 497.0445 | 0.007669 | -0.2374 | 402 |
| -0.23468 | 498.1126 | 0.007686 | -0.23642 | 403 |
| -0.23361 | 499.1807 | 0.007704 | -0.23514 | 404 |
| -0.23254 | 500.2488 | 0.007721 | -0.23419 | 405 |
| -0.23148 | 501.3169 | 0.007739 | -0.23306 | 406 |
| -0.23041 | 502.3851 | 0.007756 | -0.23206 | 407 |
| -0.22934 | 503.4532 | 0.007773 | -0.23099 | 408 |
| -0.22827 | 504.5213 | 0.00779 | -0.22995 | 409 |
| -0.2272 | 505.5894 | 0.007807 | -0.22873 | 410 |
| -0.22614 | 506.6575 | 0.007823 | -0.22781 | 411 |
| -0.22507 | 507.7256 | 0.007838 | -0.22672 | 412 |
| -0.224 | 508.7938 | 0.007854 | -0.22574 | 413 |
| -0.22293 | 509.8619 | 0.00787 | -0.22458 | 414 |
| -0.22186 | 510.93 | 0.007885 | -0.2236 | 415 |
| -0.22079 | 511.9981 | 0.0079 | -0.22247 | 416 |
| -0.21973 | 513.0662 | 0.007915 | -0.22141 | 417 |
| -0.21866 | 514.1343 | 0.00793 | -0.2204 | 418 |
| -0.21759 | 515.2024 | 0.007945 | -0.21918 | 419 |
| -0.21652 | 516.2706 | 0.007961 | -0.2182 | 420 |
| -0.21545 | 517.3387 | 0.007975 | -0.21701 | 421 |
| -0.21439 | 518.4068 | 0.00799 | -0.21606 | 422 |
| -0.21332 | 519.4749 | 0.008004 | -0.215 | 423 |
| -0.21225 | 520.543 | 0.008017 | -0.21387 | 424 |
| -0.21118 | 521.6111 | 0.008031 | -0.21289 | 425 |
| -0.21011 | 522.6792 | 0.008044 | -0.21185 | 426 |
| -0.20905 | 523.7474 | 0.008058 | -0.21078 | 427 |
| -0.20798 | 524.8155 | 0.008071 | -0.20972 | 428 |
| -0.20691 | 525.8836 | 0.008084 | -0.20862 | 429 |
| -0.20584 | 526.9517 | 0.008097 | -0.20764 | 430 |
| -0.20477 | 528.0198 | 0.00811 | -0.20651 | 431 |
| -0.2037 | 529.0879 | 0.008125 | -0.20541 | 432 |
| -0.20264 | 530.1561 | 0.008137 | -0.20428 | 433 |
| -0.20157 | 531.2242 | 0.008149 | -0.20325 | 434 |
| -0.2005 | 532.2923 | 0.008162 | -0.20236 | 435 |
| -0.19943 | 533.3604 | 0.008176 | -0.2012 | 436 |
| -0.19836 | 534.4285 | 0.008189 | -0.20007 | 437 |
| -0.1973 | 535.4966 | 0.0082 | -0.19901 | 438 |
| -0.19623 | 536.5647 | 0.008212 | -0.19806 | 439 |
| -0.19516 | 537.6329 | 0.008224 | -0.19693 | 440 |
| -0.19409 | 538.701 | 0.008236 | -0.19583 | 441 |
| -0.19302 | 539.7691 | 0.008247 | -0.19473 | 442 |
| -0.19196 | 540.8372 | 0.008258 | -0.19379 | 443 |
| -0.19089 | 541.9053 | 0.00827 | -0.19263 | 444 |
| -0.18982 | 542.9734 | 0.008281 | -0.19159 | 445 |
| -0.18875 | 544.0415 | 0.008292 | -0.19058 | 446 |
| -0.18768 | 545.1097 | 0.008303 | -0.18942 | 447 |
| -0.18661 | 546.1778 | 0.008312 | -0.18839 | 448 |
| -0.18555 | 547.2459 | 0.008323 | -0.18732 | 449 |
| -0.18448 | 548.314 | 0.008333 | -0.1864 | 450 |
| -0.18341 | 549.3821 | 0.008344 | -0.18527 | 451 |
| -0.18234 | 550.4502 | 0.008355 | -0.18414 | 452 |
| -0.18127 | 551.5184 | 0.008366 | -0.18311 | 453 |
| -0.18021 | 552.5865 | 0.008378 | -0.1821 | 454 |
| -0.17914 | 553.6546 | 0.008389 | -0.18091 | 455 |
| -0.17807 | 554.7227 | 0.008399 | -0.1799 | 456 |
| -0.177 | 555.7908 | 0.008409 | -0.17889 | 457 |
| -0.17593 | 556.8589 | 0.008416 | -0.17783 | 458 |
| -0.17487 | 557.927 | 0.008426 | -0.17679 | 459 |
| -0.1738 | 558.9952 | 0.008434 | -0.17557 | 460 |
| -0.17273 | 560.0633 | 0.008441 | -0.17447 | 461 |
| -0.17166 | 561.1314 | 0.008452 | -0.17346 | 462 |
| -0.17059 | 562.1995 | 0.008462 | -0.17252 | 463 |
| -0.16953 | 563.2676 | 0.008472 | -0.17151 | 464 |
| -0.16846 | 564.3357 | 0.00848 | -0.17026 | 465 |
| -0.16739 | 565.4038 | 0.008487 | -0.16928 | 466 |
| -0.16632 | 566.472 | 0.008494 | -0.16818 | 467 |
| -0.16525 | 567.5401 | 0.008501 | -0.16711 | 468 |
| -0.16418 | 568.6082 | 0.00851 | -0.16605 | 469 |
|  |  |  |  |  |

Sample: *Marjoram*, Concentration (ppm): 200, Immersion time: 24h

| Potential applied(V) | Time (s) | WE(1).  Current (A) | WE(1).  Potential (V) | Index |  |  |
| --- | --- | --- | --- | --- | --- | --- |
| -0.69366 | 68.74837 | -9.3E-05 | -0.69168 | 1 |  |  |
| -0.6926 | 69.81649 | -0.00093 | -0.69061 | 2 |  |  |
| -0.69153 | 70.8846 | -0.00728 | -0.69 | 3 |  |  |
| -0.69046 | 71.95272 | -0.00725 | -0.6889 | 4 |  |  |
| -0.68939 | 73.02083 | -0.00722 | -0.68787 | 5 |  |  |
| -0.68832 | 74.08895 | -0.0072 | -0.68686 | 6 |  |  |
| -0.68726 | 75.15706 | -0.00717 | -0.68579 | 7 |  |  |
| -0.68619 | 76.22518 | -0.00715 | -0.68475 | 8 |  |  |
| -0.68512 | 77.29329 | -0.00713 | -0.68356 | 9 |  |  |
| -0.68405 | 78.36141 | -0.0071 | -0.68262 | 10 |  |  |
| -0.68298 | 79.42952 | -0.00708 | -0.68152 | 11 |  |  |
| -0.68192 | 80.49764 | -0.00706 | -0.68039 | 12 |  |  |
| -0.68085 | 81.56575 | -0.00704 | -0.67938 | 13 |  |  |
| -0.67978 | 82.63387 | -0.00702 | -0.67838 | 14 |  |  |
| -0.67871 | 83.70198 | -0.007 | -0.67725 | 15 |  |  |
| -0.67764 | 84.7701 | -0.00698 | -0.67621 | 16 |  |  |
| -0.67657 | 85.83821 | -0.00696 | -0.67514 | 17 |  |  |
| -0.67551 | 86.90633 | -0.00694 | -0.6741 | 18 |  |  |
| -0.67444 | 87.97444 | -0.00692 | -0.673 | 19 |  |  |
| -0.67337 | 89.04256 | -0.0069 | -0.672 | 20 |  |  |
| -0.6723 | 90.11067 | -0.00688 | -0.67093 | 21 |  |  |
| -0.67123 | 91.17879 | -0.00686 | -0.66986 | 22 |  |  |
| -0.67017 | 92.2469 | -0.00684 | -0.66879 | 23 |  |  |
| -0.6691 | 93.31502 | -0.00682 | -0.66769 | 24 |  |  |
| -0.66803 | 94.38313 | -0.0068 | -0.66663 | 25 |  |  |
| -0.66696 | 95.45125 | -0.00678 | -0.66556 | 26 |  |  |
| -0.66589 | 96.51936 | -0.00676 | -0.66446 | 27 |  |  |
| -0.66483 | 97.58748 | -0.00674 | -0.66348 | 28 |  |  |
| -0.66376 | 98.65559 | -0.00672 | -0.66245 | 29 |  |  |
| -0.66269 | 99.72371 | -0.0067 | -0.66141 | 30 |  |  |
| -0.66162 | 100.7918 | -0.00668 | -0.66025 | 31 |  |  |
| -0.66055 | 101.8599 | -0.00666 | -0.65924 | 32 |  |  |
| -0.65948 | 102.9281 | -0.00664 | -0.65814 | 33 |  |  |
| -0.65842 | 103.9962 | -0.00662 | -0.65704 | 34 |  |  |
| -0.65735 | 105.0643 | -0.0066 | -0.65607 | 35 |  |  |
| -0.65628 | 106.1324 | -0.00658 | -0.65497 | 36 |  |  |
| -0.65521 | 107.2005 | -0.00656 | -0.6539 | 37 |  |  |
| -0.65414 | 108.2686 | -0.00655 | -0.65283 | 38 |  |  |
| -0.65308 | 109.3367 | -0.00653 | -0.65179 | 39 |  |  |
| -0.65201 | 110.4049 | -0.00651 | -0.6507 | 40 |  |  |
| -0.65094 | 111.473 | -0.00649 | -0.64966 | 41 |  |  |
| -0.64987 | 112.5411 | -0.00647 | -0.64862 | 42 |  |  |
| -0.6488 | 113.6092 | -0.00645 | -0.6474 | 43 |  |  |
| -0.64774 | 114.6773 | -0.00643 | -0.64642 | 44 |  |  |
| -0.64667 | 115.7454 | -0.00641 | -0.64539 | 45 |  |  |
| -0.6456 | 116.8135 | -0.00639 | -0.64435 | 46 |  |  |
| -0.64453 | 117.8817 | -0.00637 | -0.64331 | 47 |  |  |
| -0.64346 | 118.9498 | -0.00635 | -0.64224 | 48 |  |  |
| -0.6424 | 120.0179 | -0.00633 | -0.6412 | 49 |  |  |
| -0.64133 | 121.086 | -0.00631 | -0.64011 | 50 |  |  |
| -0.64026 | 122.1541 | -0.00629 | -0.63907 | 51 |  |  |
| -0.63919 | 123.2222 | -0.00627 | -0.63794 | 52 |  |  |
| -0.63812 | 124.2904 | -0.00625 | -0.63699 | 53 |  |  |
| -0.63705 | 125.3585 | -0.00623 | -0.63583 | 54 |  |  |
| -0.63599 | 126.4266 | -0.00621 | -0.63477 | 55 |  |  |
| -0.63492 | 127.4947 | -0.00618 | -0.63376 | 56 |  |  |
| -0.63385 | 128.5628 | -0.00616 | -0.63266 | 57 |  |  |
| -0.63278 | 129.6309 | -0.00614 | -0.63156 | 58 |  |  |
| -0.63171 | 130.699 | -0.00612 | -0.63058 | 59 |  |  |
| -0.63065 | 131.7672 | -0.0061 | -0.62952 | 60 |  |  |
| -0.62958 | 132.8353 | -0.00608 | -0.62842 | 61 |  |  |
| -0.62851 | 133.9034 | -0.00606 | -0.62738 | 62 |  |  |
| -0.62744 | 134.9715 | -0.00604 | -0.62631 | 63 |  |  |
| -0.62637 | 136.0396 | -0.00602 | -0.62524 | 64 |  |  |
| -0.62531 | 137.1077 | -0.006 | -0.62421 | 65 |  |  |
| -0.62424 | 138.1758 | -0.00597 | -0.62317 | 66 |  |  |
| -0.62317 | 139.244 | -0.00595 | -0.62207 | 67 |  |  |
| -0.6221 | 140.3121 | -0.00593 | -0.62097 | 68 |  |  |
| -0.62103 | 141.3802 | -0.00591 | -0.61993 | 69 |  |  |
| -0.61996 | 142.4483 | -0.00589 | -0.6189 | 70 |  |  |
| -0.6189 | 143.5164 | -0.00586 | -0.61786 | 71 |  |  |
| -0.61783 | 144.5845 | -0.00584 | -0.61679 | 72 |  |  |
| -0.61676 | 145.6527 | -0.00582 | -0.61566 | 73 |  |  |
| -0.61569 | 146.7208 | -0.00579 | -0.61459 | 74 |  |  |
| -0.61462 | 147.7889 | -0.00577 | -0.61356 | 75 |  |  |
| -0.61356 | 148.857 | -0.00575 | -0.6124 | 76 |  |  |
| -0.61249 | 149.9251 | -0.00572 | -0.61142 | 77 |  |  |
| -0.61142 | 150.9932 | -0.0057 | -0.61035 | 78 |  |  |
| -0.61035 | 152.0613 | -0.00568 | -0.60934 | 79 |  |  |
| -0.60928 | 153.1295 | -0.00565 | -0.60825 | 80 |  |  |
| -0.60822 | 154.1976 | -0.00563 | -0.60718 | 81 |  |  |
| -0.60715 | 155.2657 | -0.0056 | -0.60608 | 82 |  |  |
| -0.60608 | 156.3338 | -0.00558 | -0.60507 | 83 |  |  |
| -0.60501 | 157.4019 | -0.00555 | -0.60403 | 84 |  |  |
| -0.60394 | 158.47 | -0.00552 | -0.60287 | 85 |  |  |
| -0.60287 | 159.5381 | -0.0055 | -0.60196 | 86 |  |  |
| -0.60181 | 160.6063 | -0.00547 | -0.6008 | 87 |  |  |
| -0.60074 | 161.6744 | -0.00544 | -0.5997 | 88 |  |  |
| -0.59967 | 162.7425 | -0.00541 | -0.59869 | 89 |  |  |
| -0.5986 | 163.8106 | -0.00539 | -0.59763 | 90 |  |  |
| -0.59753 | 164.8787 | -0.00536 | -0.59647 | 91 |  |  |
| -0.59647 | 165.9468 | -0.00533 | -0.59549 | 92 |  |  |
| -0.5954 | 167.015 | -0.0053 | -0.59442 | 93 |  |  |
| -0.59433 | 168.0831 | -0.00527 | -0.59332 | 94 |  |  |
| -0.59326 | 169.1512 | -0.00523 | -0.59225 | 95 |  |  |
| -0.59219 | 170.2193 | -0.0052 | -0.59125 | 96 |  |  |
| -0.59113 | 171.2874 | -0.00517 | -0.59024 | 97 |  |  |
| -0.59006 | 172.3555 | -0.00513 | -0.58914 | 98 |  |  |
| -0.58899 | 173.4236 | -0.0051 | -0.58798 | 99 |  |  |
| -0.58792 | 174.4918 | -0.00506 | -0.58704 | 100 |  |  |
| -0.58685 | 175.5599 | -0.00502 | -0.58597 | 101 |  |  |
| -0.58578 | 176.628 | -0.00498 | -0.58484 | 102 |  |  |
| -0.58472 | 177.6961 | -0.00494 | -0.58383 | 103 |  |  |
| -0.58365 | 178.7642 | -0.0049 | -0.58279 | 104 |  |  |
| -0.58258 | 179.8323 | -0.00485 | -0.5817 | 105 |  |  |
| -0.58151 | 180.9004 | -0.0048 | -0.58063 | 106 |  |  |
| -0.58044 | 181.9686 | -0.00475 | -0.57959 | 107 |  |  |
| -0.57938 | 183.0367 | -0.0047 | -0.57855 | 108 |  |  |
| -0.57831 | 184.1048 | -0.00465 | -0.57755 | 109 |  |  |
| -0.57724 | 185.1729 | -0.00459 | -0.57645 | 110 |  |  |
| -0.57617 | 186.241 | -0.00453 | -0.57541 | 111 |  |  |
| -0.5751 | 187.3091 | -0.00446 | -0.57431 | 112 |  |  |
| -0.57404 | 188.3773 | -0.00439 | -0.57336 | 113 |  |  |
| -0.57297 | 189.4454 | -0.00432 | -0.57224 | 114 |  |  |
| -0.5719 | 190.5135 | -0.00424 | -0.5712 | 115 |  |  |
| -0.57083 | 191.5816 | -0.00416 | -0.57016 | 116 |  |  |
| -0.56976 | 192.6497 | -0.00407 | -0.56906 | 117 |  |  |
| -0.5687 | 193.7178 | -0.00399 | -0.56802 | 118 |  |  |
| -0.56763 | 194.7859 | -0.0039 | -0.56705 | 119 |  |  |
| -0.56656 | 195.8541 | -0.0038 | -0.56595 | 120 |  |  |
| -0.56549 | 196.9222 | -0.00371 | -0.56497 | 121 |  |  |
| -0.56442 | 197.9903 | -0.00362 | -0.56387 | 122 |  |  |
| -0.56335 | 199.0584 | -0.00353 | -0.56277 | 123 |  |  |
| -0.56229 | 200.1265 | -0.00344 | -0.56174 | 124 |  |  |
| -0.56122 | 201.1946 | -0.00335 | -0.56076 | 125 |  |  |
| -0.56015 | 202.2627 | -0.00326 | -0.55963 | 126 |  |  |
| -0.55908 | 203.3309 | -0.00318 | -0.55859 | 127 |  |  |
| -0.55801 | 204.399 | -0.0031 | -0.55756 | 128 |  |  |
| -0.55695 | 205.4671 | -0.00302 | -0.55655 | 129 |  |  |
| -0.55588 | 206.5352 | -0.00294 | -0.55548 | 130 |  |  |
| -0.55481 | 207.6033 | -0.00286 | -0.55444 | 131 |  |  |
| -0.55374 | 208.6714 | -0.00278 | -0.55341 | 132 |  |  |
| -0.55267 | 209.7396 | -0.00271 | -0.55237 | 133 |  |  |
| -0.55161 | 210.8077 | -0.00264 | -0.55133 | 134 |  |  |
| -0.55054 | 211.8758 | -0.00257 | -0.5502 | 135 |  |  |
| -0.54947 | 212.9439 | -0.0025 | -0.54913 | 136 |  |  |
| -0.5484 | 214.012 | -0.00244 | -0.54822 | 137 |  |  |
| -0.54733 | 215.0801 | -0.00237 | -0.54709 | 138 |  |  |
| -0.54626 | 216.1482 | -0.00231 | -0.54599 | 139 |  |  |
| -0.5452 | 217.2164 | -0.00225 | -0.54501 | 140 |  |  |
| -0.54413 | 218.2845 | -0.00219 | -0.54382 | 141 |  |  |
| -0.54306 | 219.3526 | -0.00213 | -0.54285 | 142 |  |  |
| -0.54199 | 220.4207 | -0.00207 | -0.54178 | 143 |  |  |
| -0.54092 | 221.4888 | -0.00202 | -0.54071 | 144 |  |  |
| -0.53986 | 222.5569 | -0.00197 | -0.53973 | 145 |  |  |
| -0.53879 | 223.625 | -0.00191 | -0.53864 | 146 |  |  |
| -0.53772 | 224.6932 | -0.00186 | -0.5376 | 147 |  |  |
| -0.53665 | 225.7613 | -0.00181 | -0.5365 | 148 |  |  |
| -0.53558 | 226.8294 | -0.00176 | -0.53546 | 149 |  |  |
| -0.53452 | 227.8975 | -0.00172 | -0.53442 | 150 |  |  |
| -0.53345 | 228.9656 | -0.00167 | -0.53333 | 151 |  |  |
| -0.53238 | 230.0337 | -0.00163 | -0.53238 | 152 |  |  |
| -0.53131 | 231.1019 | -0.00159 | -0.53116 | 153 |  |  |
| -0.53024 | 232.17 | -0.00154 | -0.53018 | 154 |  |  |
| -0.52917 | 233.2381 | -0.0015 | -0.52914 | 155 |  |  |
| -0.52811 | 234.3062 | -0.00146 | -0.52805 | 156 |  |  |
| -0.52704 | 235.3743 | -0.00142 | -0.52701 | 157 |  |  |
| -0.52597 | 236.4424 | -0.00139 | -0.52591 | 158 |  |  |
| -0.5249 | 237.5105 | -0.00135 | -0.52484 | 159 |  |  |
| -0.52383 | 238.5787 | -0.00131 | -0.52377 | 160 |  |  |
| -0.52277 | 239.6468 | -0.00128 | -0.52274 | 161 |  |  |
| -0.5217 | 240.7149 | -0.00124 | -0.5217 | 162 |  |  |
| -0.52063 | 241.783 | -0.00121 | -0.52066 | 163 |  |  |
| -0.51956 | 242.8511 | -0.00118 | -0.51953 | 164 |  |  |
| -0.51849 | 243.9192 | -0.00115 | -0.51849 | 165 |  |  |
| -0.51743 | 244.9873 | -0.00112 | -0.51749 | 166 |  |  |
| -0.51636 | 246.0555 | -0.00109 | -0.51636 | 167 |  |  |
| -0.51529 | 247.1236 | -0.00106 | -0.51526 | 168 |  |  |
| -0.51422 | 248.1917 | -0.00103 | -0.51422 | 169 |  |  |
| -0.51315 | 249.2598 | -0.001 | -0.51309 | 170 |  |  |
| -0.51208 | 250.3279 | -0.00098 | -0.51208 | 171 |  |  |
| -0.51102 | 251.396 | -0.00095 | -0.51105 | 172 |  |  |
| -0.50995 | 252.4642 | -0.00093 | -0.50992 | 173 |  |  |
| -0.50888 | 253.5323 | -0.0009 | -0.50888 | 174 |  |  |
| -0.50781 | 254.6004 | -0.00088 | -0.50784 | 175 |  |  |
| -0.50674 | 255.6685 | -0.00085 | -0.50684 | 176 |  |  |
| -0.50568 | 256.7366 | -0.00083 | -0.50574 | 177 |  |  |
| -0.50461 | 257.8047 | -0.00081 | -0.50461 | 178 |  |  |
| -0.50354 | 258.8728 | -0.00079 | -0.50357 | 179 |  |  |
| -0.50247 | 259.941 | -0.00076 | -0.50247 | 180 |  |  |
| -0.5014 | 261.0091 | -0.00074 | -0.50143 | 181 |  |  |
| -0.50034 | 262.0772 | -0.00072 | -0.5004 | 182 |  |  |
| -0.49927 | 263.1453 | -0.0007 | -0.49939 | 183 |  |  |
| -0.4982 | 264.2134 | -0.00069 | -0.49829 | 184 |  |  |
| -0.49713 | 265.2815 | -0.00067 | -0.49719 | 185 |  |  |
| -0.49606 | 266.3496 | -0.00065 | -0.49609 | 186 |  |  |
| -0.495 | 267.4178 | -0.00063 | -0.49509 | 187 |  |  |
| -0.49393 | 268.4859 | -0.00061 | -0.49396 | 188 |  |  |
| -0.49286 | 269.554 | -0.0006 | -0.49292 | 189 |  |  |
| -0.49179 | 270.6221 | -0.00058 | -0.49191 | 190 |  |  |
| -0.49072 | 271.6902 | -0.00056 | -0.49075 | 191 |  |  |
| -0.48965 | 272.7583 | -0.00055 | -0.48969 | 192 |  |  |
| -0.48859 | 273.8265 | -0.00053 | -0.48871 | 193 |  |  |
| -0.48752 | 274.8946 | -0.00052 | -0.48761 | 194 |  |  |
| -0.48645 | 275.9627 | -0.0005 | -0.48651 | 195 |  |  |
| -0.48538 | 277.0308 | -0.00049 | -0.48544 | 196 |  |  |
| -0.48431 | 278.0989 | -0.00047 | -0.48444 | 197 |  |  |
| -0.48325 | 279.167 | -0.00046 | -0.48325 | 198 |  |  |
| -0.48218 | 280.2351 | -0.00045 | -0.48227 | 199 |  |  |
| -0.48111 | 281.3033 | -0.00043 | -0.48126 | 200 |  |  |
| -0.48004 | 282.3714 | -0.00042 | -0.4801 | 201 |  |  |
| -0.47897 | 283.4395 | -0.00041 | -0.47903 | 202 |  |  |
| -0.47791 | 284.5076 | -0.0004 | -0.47806 | 203 |  |  |
| -0.47684 | 285.5757 | -0.00038 | -0.47681 | 204 |  |  |
| -0.47577 | 286.6438 | -0.00037 | -0.47586 | 205 |  |  |
| -0.4747 | 287.7119 | -0.00036 | -0.47479 | 206 |  |  |
| -0.47363 | 288.7801 | -0.00035 | -0.47375 | 207 |  |  |
| -0.47256 | 289.8482 | -0.00034 | -0.47266 | 208 |  |  |
| -0.4715 | 290.9163 | -0.00033 | -0.47153 | 209 |  |  |
| -0.47043 | 291.9844 | -0.00032 | -0.47049 | 210 |  |  |
| -0.46936 | 293.0525 | -0.00031 | -0.46942 | 211 |  |  |
| -0.46829 | 294.1206 | -0.0003 | -0.46841 | 212 |  |  |
| -0.46722 | 295.1888 | -0.00029 | -0.46725 | 213 |  |  |
| -0.46616 | 296.2569 | -0.00028 | -0.46631 | 214 |  |  |
| -0.46509 | 297.325 | -0.00027 | -0.46527 | 215 |  |  |
| -0.46402 | 298.3931 | -0.00026 | -0.46411 | 216 |  |  |
| -0.46295 | 299.4612 | -0.00025 | -0.46304 | 217 |  |  |
| -0.46188 | 300.5293 | -0.00024 | -0.46201 | 218 |  |  |
| -0.46082 | 301.5974 | -0.00023 | -0.46091 | 219 |  |  |
| -0.45975 | 302.6656 | -0.00022 | -0.45984 | 220 |  |  |
| -0.45868 | 303.7337 | -0.00021 | -0.45877 | 221 |  |  |
| -0.45761 | 304.8018 | -0.0002 | -0.4577 | 222 |  |  |
| -0.45654 | 305.8699 | -0.00019 | -0.45663 | 223 |  |  |
| -0.45547 | 306.938 | -0.00018 | -0.45551 | 224 |  |  |
| -0.45441 | 308.0061 | -0.00018 | -0.45453 | 225 |  |  |
| -0.45334 | 309.0742 | -0.00017 | -0.45352 | 226 |  |  |
| -0.45227 | 310.1424 | -0.00016 | -0.45239 | 227 |  |  |
| -0.4512 | 311.2105 | -0.00015 | -0.45129 | 228 |  |  |
| -0.45013 | 312.2786 | -0.00014 | -0.45029 | 229 |  |  |
| -0.44907 | 313.3467 | -0.00013 | -0.44919 | 230 |  |  |
| -0.448 | 314.4148 | -0.00013 | -0.44809 | 231 |  |  |
| -0.44693 | 315.4829 | -0.00012 | -0.44705 | 232 |  |  |
| -0.44586 | 316.5511 | -0.00011 | -0.44595 | 233 |  |  |
| -0.44479 | 317.6192 | -0.0001 | -0.44492 | 234 |  |  |
| -0.44373 | 318.6873 | -9.3E-05 | -0.44382 | 235 |  |  |
| -0.44266 | 319.7554 | -8.5E-05 | -0.44284 | 236 |  |  |
| -0.44159 | 320.8235 | -7.7E-05 | -0.44174 | 237 |  |  |
| -0.44052 | 321.8916 | -6.9E-05 | -0.44055 | 238 |  |  |
| -0.43945 | 322.9597 | -6.1E-05 | -0.43958 | 239 |  |  |
| -0.43839 | 324.0279 | -5.3E-05 | -0.43848 | 240 |  |  |
| -0.43732 | 325.096 | -4.4E-05 | -0.4375 | 241 |  |  |
| -0.43625 | 326.1641 | -3.5E-05 | -0.43631 | 242 |  |  |
| -0.43518 | 327.2322 | -2.7E-05 | -0.43524 | 243 |  |  |
| -0.43411 | 328.3003 | -1.9E-05 | -0.4342 | 244 |  |  |
| -0.43304 | 329.3684 | -1.2E-05 | -0.43314 | 245 |  |  |
| -0.43198 | 330.4365 | -3.6E-06 | -0.4321 | 246 |  |  |
| -0.43091 | 331.5047 | 4.24E-06 | -0.43103 | 247 |  |  |
| -0.42984 | 332.5728 | 1.27E-05 | -0.43002 | 248 |  |  |
| -0.42877 | 333.6409 | 1.99E-05 | -0.42892 | 249 |  |  |
| -0.4277 | 334.709 | 2.87E-05 | -0.42798 | 250 |  |  |
| -0.42664 | 335.7771 | 3.71E-05 | -0.42688 | 251 |  |  |
| -0.42557 | 336.8452 | 4.76E-05 | -0.42575 | 252 |  |  |
| -0.4245 | 337.9134 | 5.68E-05 | -0.4248 | 253 |  |  |
| -0.42343 | 338.9815 | 6.6E-05 | -0.42368 | 254 |  |  |
| -0.42236 | 340.0496 | 7.55E-05 | -0.42255 | 255 |  |  |
| -0.4213 | 341.1177 | 8.52E-05 | -0.42166 | 256 |  |  |
| -0.42023 | 342.1858 | 9.47E-05 | -0.42053 | 257 |  |  |
| -0.41916 | 343.2539 | 0.000105 | -0.41946 | 258 |  |  |
| -0.41809 | 344.322 | 0.000115 | -0.41837 | 259 |  |  |
| -0.41702 | 345.3902 | 0.000125 | -0.41739 | 260 |  |  |
| -0.41595 | 346.4583 | 0.000136 | -0.41629 | 261 |  |  |
| -0.41489 | 347.5264 | 0.000147 | -0.41531 | 262 |  |  |
| -0.41382 | 348.5945 | 0.000159 | -0.41418 | 263 |  |  |
| -0.41275 | 349.6626 | 0.00017 | -0.41312 | 264 |  |  |
| -0.41168 | 350.7307 | 0.000182 | -0.41214 | 265 |  |  |
| -0.41061 | 351.7988 | 0.000194 | -0.4111 | 266 |  |  |
| -0.40955 | 352.867 | 0.000207 | -0.40985 | 267 |  |  |
| -0.40848 | 353.9351 | 0.00022 | -0.40891 | 268 |  |  |
| -0.40741 | 355.0032 | 0.000234 | -0.40784 | 269 |  |  |
| -0.40634 | 356.0713 | 0.000248 | -0.40671 | 270 |  |  |
| -0.40527 | 357.1394 | 0.000262 | -0.40567 | 271 |  |  |
| -0.40421 | 358.2075 | 0.000277 | -0.40472 | 272 |  |  |
| -0.40314 | 359.2757 | 0.000292 | -0.40356 | 273 |  |  |
| -0.40207 | 360.3438 | 0.000308 | -0.40262 | 274 |  |  |
| -0.401 | 361.4119 | 0.000325 | -0.40125 | 275 |  |  |
| -0.39993 | 362.48 | 0.000342 | -0.40012 | 276 |  |  |
| -0.39886 | 363.5481 | 0.00036 | -0.39902 | 277 |  |  |
| -0.3978 | 364.6162 | 0.000379 | -0.39798 | 278 |  |  |
| -0.39673 | 365.6843 | 0.000398 | -0.39685 | 279 |  |  |
| -0.39566 | 366.7525 | 0.000418 | -0.39581 | 280 |  |  |
| -0.39459 | 367.8206 | 0.000439 | -0.39478 | 281 |  |  |
| -0.39352 | 368.8887 | 0.000461 | -0.39374 | 282 |  |  |
| -0.39246 | 369.9568 | 0.000484 | -0.39261 | 283 |  |  |
| -0.39139 | 371.0249 | 0.000508 | -0.39151 | 284 |  |  |
| -0.39032 | 372.093 | 0.000533 | -0.39053 | 285 |  |  |
| -0.38925 | 373.1611 | 0.000558 | -0.3895 | 286 |  |  |
| -0.38818 | 374.2293 | 0.000586 | -0.3884 | 287 |  |  |
| -0.38712 | 375.2974 | 0.000614 | -0.38733 | 288 |  |  |
| -0.38605 | 376.3655 | 0.000644 | -0.38635 | 289 |  |  |
| -0.38498 | 377.4336 | 0.000675 | -0.38522 | 290 |  |  |
| -0.38391 | 378.5017 | 0.000707 | -0.38412 | 291 |  |  |
| -0.38284 | 379.5698 | 0.000741 | -0.38312 | 292 |  |  |
| -0.38177 | 380.638 | 0.000777 | -0.38202 | 293 |  |  |
| -0.38071 | 381.7061 | 0.000814 | -0.38098 | 294 |  |  |
| -0.37964 | 382.7742 | 0.000853 | -0.37991 | 295 |  |  |
| -0.37857 | 383.8423 | 0.000894 | -0.37878 | 296 |  |  |
| -0.3775 | 384.9104 | 0.000936 | -0.37781 | 297 |  |  |
| -0.37643 | 385.9785 | 0.00098 | -0.37671 | 298 |  |  |
| -0.37537 | 387.0466 | 0.001027 | -0.37573 | 299 |  |  |
| -0.3743 | 388.1148 | 0.001076 | -0.37463 | 300 |  |  |
| -0.37323 | 389.1829 | 0.001127 | -0.3735 | 301 |  |  |
| -0.37216 | 390.251 | 0.001181 | -0.37247 | 302 |  |  |
| -0.37109 | 391.3191 | 0.001237 | -0.37137 | 303 |  |  |
| -0.37003 | 392.3872 | 0.001297 | -0.37033 | 304 |  |  |
| -0.36896 | 393.4553 | 0.001359 | -0.3692 | 305 |  |  |
| -0.36789 | 394.5234 | 0.001424 | -0.36819 | 306 |  |  |
| -0.36682 | 395.5916 | 0.001492 | -0.36713 | 307 |  |  |
| -0.36575 | 396.6597 | 0.001564 | -0.36609 | 308 |  |  |
| -0.36469 | 397.7278 | 0.00164 | -0.36505 | 309 |  |  |
| -0.36362 | 398.7959 | 0.001719 | -0.36401 | 310 |  |  |
| -0.36255 | 399.864 | 0.001803 | -0.36292 | 311 |  |  |
| -0.36148 | 400.9321 | 0.001891 | -0.36185 | 312 |  |  |
| -0.36041 | 402.0003 | 0.001983 | -0.36084 | 313 |  |  |
| -0.35934 | 403.0684 | 0.00208 | -0.35965 | 314 |  |  |
| -0.35828 | 404.1365 | 0.002182 | -0.35864 | 315 |  |  |
| -0.35721 | 405.2046 | 0.00229 | -0.3576 | 316 |  |  |
| -0.35614 | 406.2727 | 0.002404 | -0.35654 | 317 |  |  |
| -0.35507 | 407.3408 | 0.002523 | -0.3555 | 318 |  |  |
| -0.354 | 408.4089 | 0.00265 | -0.35446 | 319 |  |  |
| -0.35294 | 409.4771 | 0.002784 | -0.35345 | 320 |  |  |
| -0.35187 | 410.5452 | 0.002925 | -0.35236 | 321 |  |  |
| -0.3508 | 411.6133 | 0.003072 | -0.35135 | 322 |  |  |
| -0.34973 | 412.6814 | 0.003227 | -0.35034 | 323 |  |  |
| -0.34866 | 413.7495 | 0.00339 | -0.3494 | 324 |  |  |
| -0.3476 | 414.8176 | 0.00356 | -0.34824 | 325 |  |  |
| -0.34653 | 415.8857 | 0.003734 | -0.34729 | 326 |  |  |
| -0.34546 | 416.9539 | 0.003907 | -0.34616 | 327 |  |  |
| -0.34439 | 418.022 | 0.004078 | -0.34515 | 328 |  |  |
| -0.34332 | 419.0901 | 0.00424 | -0.34409 | 329 |  |  |
| -0.34225 | 420.1582 | 0.004388 | -0.34305 | 330 |  |  |
| -0.34119 | 421.2263 | 0.004522 | -0.34207 | 331 |  |  |
| -0.34012 | 422.2944 | 0.004642 | -0.341 | 332 |  |  |
| -0.33905 | 423.3626 | 0.00475 | -0.33994 | 333 |  |  |
| -0.33798 | 424.4307 | 0.004848 | -0.3389 | 334 |  |  |
| -0.33691 | 425.4988 | 0.004939 | -0.33789 | 335 |  |  |
| -0.33585 | 426.5669 | 0.005023 | -0.33682 | 336 |  |  |
| -0.33478 | 427.635 | 0.005101 | -0.33582 | 337 |  |  |
| -0.33371 | 428.7031 | 0.005175 | -0.33469 | 338 |  |  |
| -0.33264 | 429.7712 | 0.005246 | -0.33365 | 339 |  |  |
| -0.33157 | 430.8394 | 0.005313 | -0.33264 | 340 |  |  |
| -0.33051 | 431.9075 | 0.005378 | -0.33151 | 341 |  |  |
| -0.32944 | 432.9756 | 0.00544 | -0.33057 | 342 |  |  |
| -0.32837 | 434.0437 | 0.005501 | -0.32947 | 343 |  |  |
| -0.3273 | 435.1118 | 0.00556 | -0.32843 | 344 |  |  |
| -0.32623 | 436.1799 | 0.005618 | -0.32736 | 345 |  |  |
| -0.32516 | 437.248 | 0.005674 | -0.32626 | 346 |  |  |
| -0.3241 | 438.3162 | 0.00573 | -0.32526 | 347 |  |  |
| -0.32303 | 439.3843 | 0.005785 | -0.32413 | 348 |  |  |
| -0.32196 | 440.4524 | 0.005839 | -0.32309 | 349 |  |  |
| -0.32089 | 441.5205 | 0.005892 | -0.32208 | 350 |  |  |
| -0.31982 | 442.5886 | 0.005944 | -0.32104 | 351 |  |  |
| -0.31876 | 443.6567 | 0.005996 | -0.32001 | 352 |  |  |
| -0.31769 | 444.7249 | 0.006048 | -0.31897 | 353 |  |  |
| -0.31662 | 445.793 | 0.006099 | -0.31787 | 354 |  |  |
| -0.31555 | 446.8611 | 0.00615 | -0.3168 | 355 |  |  |
| -0.31448 | 447.9292 | 0.0062 | -0.31583 | 356 |  |  |
| -0.31342 | 448.9973 | 0.00625 | -0.31476 | 357 |  |  |
| -0.31235 | 450.0654 | 0.0063 | -0.3136 | 358 |  |  |
| -0.31128 | 451.1335 | 0.00635 | -0.31259 | 359 |  |  |
| -0.31021 | 452.2017 | 0.006399 | -0.31152 | 360 |  |  |
| -0.30914 | 453.2698 | 0.006447 | -0.31049 | 361 |  |  |
| -0.30807 | 454.3379 | 0.006495 | -0.30939 | 362 |  |  |
| -0.30701 | 455.406 | 0.006543 | -0.30838 | 363 |  |  |
| -0.30594 | 456.4741 | 0.006591 | -0.30728 | 364 |  |  |
| -0.30487 | 457.5422 | 0.006638 | -0.3063 | 365 |  |  |
| -0.3038 | 458.6103 | 0.006684 | -0.30518 | 366 |  |  |
| -0.30273 | 459.6785 | 0.00673 | -0.30417 | 367 |  |  |
| -0.30167 | 460.7466 | 0.006775 | -0.30301 | 368 |  |  |
| -0.3006 | 461.8147 | 0.006819 | -0.30203 | 369 |  |  |
| -0.29953 | 462.8828 | 0.006862 | -0.30096 | 370 |  |  |
| -0.29846 | 463.9509 | 0.006905 | -0.29987 | 371 |  |  |
| -0.29739 | 465.019 | 0.006946 | -0.29877 | 372 |  |  |
| -0.29633 | 466.0872 | 0.006986 | -0.29779 | 373 |  |  |
| -0.29526 | 467.1553 | 0.007025 | -0.29672 | 374 |  |  |
| -0.29419 | 468.2234 | 0.007063 | -0.29572 | 375 |  |  |
| -0.29312 | 469.2915 | 0.0071 | -0.29465 | 376 |  |  |
| -0.29205 | 470.3596 | 0.007136 | -0.29355 | 377 |  |  |
| -0.29099 | 471.4277 | 0.00717 | -0.29248 | 378 |  |  |
| -0.28992 | 472.4958 | 0.007204 | -0.29147 | 379 |  |  |
| -0.28885 | 473.564 | 0.007235 | -0.29037 | 380 |  |  |
| -0.28778 | 474.6321 | 0.007267 | -0.28925 | 381 |  |  |
| -0.28671 | 475.7002 | 0.007296 | -0.2883 | 382 |  |  |
| -0.28564 | 476.7683 | 0.007325 | -0.28717 | 383 |  |  |
| -0.28458 | 477.8364 | 0.007353 | -0.2861 | 384 |  |  |
| -0.28351 | 478.9045 | 0.00738 | -0.2851 | 385 |  |  |
| -0.28244 | 479.9726 | 0.007406 | -0.284 | 386 |  |  |
| -0.28137 | 481.0408 | 0.007431 | -0.28293 | 387 |  |  |
| -0.2803 | 482.1089 | 0.007455 | -0.28189 | 388 |  |  |
| -0.27924 | 483.177 | 0.007479 | -0.28079 | 389 |  |  |
| -0.27817 | 484.2451 | 0.007502 | -0.27969 | 390 |  |  |
| -0.2771 | 485.3132 | 0.007525 | -0.27863 | 391 |  |  |
| -0.27603 | 486.3813 | 0.007547 | -0.27762 | 392 |  |  |
| -0.27496 | 487.4495 | 0.007568 | -0.27655 | 393 |  |  |
| -0.2739 | 488.5176 | 0.00759 | -0.27545 | 394 |  |  |
| -0.27283 | 489.5857 | 0.00761 | -0.27451 | 395 |  |  |
| -0.27176 | 490.6538 | 0.007631 | -0.27335 | 396 |  |  |
| -0.27069 | 491.7219 | 0.00765 | -0.27234 | 397 |  |  |
| -0.26962 | 492.79 | 0.00767 | -0.27127 | 398 |  |  |
| -0.26855 | 493.8581 | 0.007689 | -0.2702 | 399 |  |  |
| -0.26749 | 494.9263 | 0.007708 | -0.26904 | 400 |  |  |
| -0.26642 | 495.9944 | 0.007726 | -0.26807 | 401 |  |  |
| -0.26535 | 497.0625 | 0.007744 | -0.26703 | 402 |  |  |
| -0.26428 | 498.1306 | 0.007762 | -0.26593 | 403 |  |  |
| -0.26321 | 499.1987 | 0.007779 | -0.26489 | 404 |  |  |
| -0.26215 | 500.2668 | 0.007796 | -0.26376 | 405 |  |  |
| -0.26108 | 501.3349 | 0.007813 | -0.26263 | 406 |  |  |
| -0.26001 | 502.4031 | 0.00783 | -0.26166 | 407 |  |  |
| -0.25894 | 503.4712 | 0.007846 | -0.26059 | 408 |  |  |
| -0.25787 | 504.5393 | 0.007863 | -0.25958 | 409 |  |  |
| -0.25681 | 505.6074 | 0.007879 | -0.25845 | 410 |  |  |
| -0.25574 | 506.6755 | 0.007894 | -0.25742 | 411 |  |  |
| -0.25467 | 507.7436 | 0.00791 | -0.25638 | 412 |  |  |
| -0.2536 | 508.8118 | 0.007925 | -0.25531 | 413 |  |  |
| -0.25253 | 509.8799 | 0.007941 | -0.25424 | 414 |  |  |
| -0.25146 | 510.948 | 0.007956 | -0.2532 | 415 |  |  |
| -0.2504 | 512.0161 | 0.00797 | -0.25211 | 416 |  |  |
| -0.24933 | 513.0842 | 0.007984 | -0.25098 | 417 |  |  |
| -0.24826 | 514.1523 | 0.007998 | -0.24997 | 418 |  |  |
| -0.24719 | 515.2204 | 0.008013 | -0.2489 | 419 |  |  |
| -0.24612 | 516.2886 | 0.008027 | -0.24786 | 420 |  |  |
| -0.24506 | 517.3567 | 0.00804 | -0.2468 | 421 |  |  |
| -0.24399 | 518.4248 | 0.008055 | -0.24573 | 422 |  |  |
| -0.24292 | 519.4929 | 0.008068 | -0.24466 | 423 |  |  |
| -0.24185 | 520.561 | 0.008081 | -0.24359 | 424 |  |  |
| -0.24078 | 521.6291 | 0.008094 | -0.24243 | 425 |  |  |
| -0.23972 | 522.6972 | 0.008107 | -0.24149 | 426 |  |  |
| -0.23865 | 523.7654 | 0.008119 | -0.24045 | 427 |  |  |
| -0.23758 | 524.8335 | 0.008132 | -0.23932 | 428 |  |  |
| -0.23651 | 525.9016 | 0.008143 | -0.23825 | 429 |  |  |
| -0.23544 | 526.9697 | 0.008155 | -0.23721 | 430 |  |  |
| -0.23438 | 528.0378 | 0.008167 | -0.23615 | 431 |  |  |
| -0.23331 | 529.1059 | 0.008179 | -0.23511 | 432 |  |  |
| -0.23224 | 530.1741 | 0.00819 | -0.23404 | 433 |  |  |
| -0.23117 | 531.2422 | 0.008201 | -0.233 | 434 |  |  |
| -0.2301 | 532.3103 | 0.008213 | -0.2319 | 435 |  |  |
| -0.22903 | 533.3784 | 0.008224 | -0.2308 | 436 |  |  |
| -0.22797 | 534.4465 | 0.008235 | -0.22986 | 437 |  |  |
| -0.2269 | 535.5146 | 0.008245 | -0.22873 | 438 |  |  |
| -0.22583 | 536.5827 | 0.008255 | -0.22757 | 439 |  |  |
| -0.22476 | 537.6509 | 0.008265 | -0.22653 | 440 |  |  |
| -0.22369 | 538.719 | 0.008275 | -0.22552 | 441 |  |  |
| -0.22263 | 539.7871 | 0.008288 | -0.2244 | 442 |  |  |
| -0.22156 | 540.8552 | 0.008298 | -0.22336 | 443 |  |  |
| -0.22049 | 541.9233 | 0.008308 | -0.22235 | 444 |  |  |
| -0.21942 | 542.9914 | 0.008318 | -0.22122 | 445 |  |  |
| -0.21835 | 544.0595 | 0.008327 | -0.22021 | 446 |  |  |
| -0.21729 | 545.1277 | 0.008337 | -0.21912 | 447 |  |  |
| -0.21622 | 546.1958 | 0.008346 | -0.21805 | 448 |  |  |
| -0.21515 | 547.2639 | 0.008354 | -0.21698 | 449 |  |  |
| -0.21408 | 548.332 | 0.008363 | -0.21597 | 450 |  |  |
| -0.21301 | 549.4001 | 0.008372 | -0.21484 | 451 |  |  |
| -0.21194 | 550.4682 | 0.008382 | -0.21381 | 452 |  |  |
| -0.21088 | 551.5364 | 0.008392 | -0.21274 | 453 |  |  |
| -0.20981 | 552.6045 | 0.008402 | -0.21167 | 454 |  |  |
| -0.20874 | 553.6726 | 0.008412 | -0.21063 | 455 |  |  |
| -0.20767 | 554.7407 | 0.008421 | -0.20956 | 456 |  |  |
| -0.2066 | 555.8088 | 0.008429 | -0.2085 | 457 |  |  |
| -0.20554 | 556.8769 | 0.008437 | -0.20737 | 458 |  |  |
| -0.20447 | 557.945 | 0.008445 | -0.20639 | 459 |  |  |
| -0.2034 | 559.0132 | 0.008451 | -0.20529 | 460 |  |  |
| -0.20233 | 560.0813 | 0.008467 | -0.20419 | 461 |  |  |
| -0.20126 | 561.1494 | 0.008475 | -0.20319 | 462 |  |  |
| -0.2002 | 562.2175 | 0.008483 | -0.20203 | 463 |  |  |
| -0.19913 | 563.2856 | 0.008489 | -0.20099 | 464 |  |  |
| -0.19806 | 564.3537 | 0.008495 | -0.19992 | 465 |  |  |
| -0.19699 | 565.4218 | 0.008501 | -0.19888 | 466 |  |  |
| -0.19592 | 566.49 | 0.008507 | -0.19781 | 467 |  |  |
| -0.19485 | 567.5581 | 0.008514 | -0.19666 | 468 |  |  |
| -0.19379 | 568.6262 | 0.008519 | -0.19565 | 469 |  |  |

Sample: *Marjoram*, Concentration (ppm): 600, Immersion time: 24h

| Potential applied(V) | Time (s) | WE(1).  Current (A) |  | WE(1).  Potential (V) | Index |  |  |
| --- | --- | --- | --- | --- | --- | --- | --- |
| -0.68802 | 68.25335 | -9.3E-06 |  | -0.68594 | 1 |  |  |
| -0.68695 | 69.32147 | -9.3E-05 |  | -0.68491 | 2 |  |  |
| -0.68588 | 70.38958 | -0.00093 |  | -0.68375 | 3 |  |  |
| -0.68481 | 71.4577 | -0.00652 |  | -0.68338 | 4 |  |  |
| -0.68375 | 72.52581 | -0.0065 |  | -0.68246 | 5 |  |  |
| -0.68268 | 73.59393 | -0.00648 |  | -0.68137 | 6 |  |  |
| -0.68161 | 74.66204 | -0.00647 |  | -0.6803 | 7 |  |  |
| -0.68054 | 75.73016 | -0.00645 |  | -0.67926 | 8 |  |  |
| -0.67947 | 76.79827 | -0.00643 |  | -0.6781 | 9 |  |  |
| -0.67841 | 77.86639 | -0.00641 |  | -0.67715 | 10 |  |  |
| -0.67734 | 78.9345 | -0.0064 |  | -0.67599 | 11 |  |  |
| -0.67627 | 80.00262 | -0.00638 |  | -0.67505 | 12 |  |  |
| -0.6752 | 81.07073 | -0.00636 |  | -0.67401 | 13 |  |  |
| -0.67413 | 82.13885 | -0.00635 |  | -0.67282 | 14 |  |  |
| -0.67307 | 83.20696 | -0.00633 |  | -0.67184 | 15 |  |  |
| -0.672 | 84.27508 | -0.00631 |  | -0.67075 | 16 |  |  |
| -0.67093 | 85.34319 | -0.0063 |  | -0.66962 | 17 |  |  |
| -0.66986 | 86.41131 | -0.00628 |  | -0.66852 | 18 |  |  |
| -0.66879 | 87.47942 | -0.00626 |  | -0.66757 | 19 |  |  |
| -0.66772 | 88.54754 | -0.00625 |  | -0.66644 | 20 |  |  |
| -0.66666 | 89.61565 | -0.00623 |  | -0.66537 | 21 |  |  |
| -0.66559 | 90.68377 | -0.00621 |  | -0.66418 | 22 |  |  |
| -0.66452 | 91.75188 | -0.0062 |  | -0.6633 | 23 |  |  |
| -0.66345 | 92.82 | -0.00618 |  | -0.66223 | 24 |  |  |
| -0.66238 | 93.88811 | -0.00616 |  | -0.66119 | 25 |  |  |
| -0.66132 | 94.95623 | -0.00615 |  | -0.66006 | 26 |  |  |
| -0.66025 | 96.02434 | -0.00613 |  | -0.65906 | 27 |  |  |
| -0.65918 | 97.09246 | -0.00611 |  | -0.65805 | 28 |  |  |
| -0.65811 | 98.16057 | -0.00609 |  | -0.65692 | 29 |  |  |
| -0.65704 | 99.22869 | -0.00608 |  | -0.65591 | 30 |  |  |
| -0.65598 | 100.2968 | -0.00606 |  | -0.65479 | 31 |  |  |
| -0.65491 | 101.3649 | -0.00604 |  | -0.65375 | 32 |  |  |
| -0.65384 | 102.433 | -0.00602 |  | -0.65265 | 33 |  |  |
| -0.65277 | 103.5011 | -0.006 |  | -0.65176 | 34 |  |  |
| -0.6517 | 104.5693 | -0.00598 |  | -0.6506 | 35 |  |  |
| -0.65063 | 105.6374 | -0.00597 |  | -0.64948 | 36 |  |  |
| -0.64957 | 106.7055 | -0.00595 |  | -0.64841 | 37 |  |  |
| -0.6485 | 107.7736 | -0.00593 |  | -0.64737 | 38 |  |  |
| -0.64743 | 108.8417 | -0.00591 |  | -0.64636 | 39 |  |  |
| -0.64636 | 109.9098 | -0.00589 |  | -0.64529 | 40 |  |  |
| -0.64529 | 110.978 | -0.00587 |  | -0.64417 | 41 |  |  |
| -0.64423 | 112.0461 | -0.00585 |  | -0.6431 | 42 |  |  |
| -0.64316 | 113.1142 | -0.00583 |  | -0.64209 | 43 |  |  |
| -0.64209 | 114.1823 | -0.00581 |  | -0.64105 | 44 |  |  |
| -0.64102 | 115.2504 | -0.00579 |  | -0.63995 | 45 |  |  |
| -0.63995 | 116.3185 | -0.00577 |  | -0.63889 | 46 |  |  |
| -0.63889 | 117.3866 | -0.00575 |  | -0.63797 | 47 |  |  |
| -0.63782 | 118.4548 | -0.00573 |  | -0.63666 | 48 |  |  |
| -0.63675 | 119.5229 | -0.0057 |  | -0.63574 | 49 |  |  |
| -0.63568 | 120.591 | -0.00568 |  | -0.63461 | 50 |  |  |
| -0.63461 | 121.6591 | -0.00566 |  | -0.63361 | 51 |  |  |
| -0.63354 | 122.7272 | -0.00564 |  | -0.63248 | 52 |  |  |
| -0.63248 | 123.7953 | -0.00562 |  | -0.6315 | 53 |  |  |
| -0.63141 | 124.8634 | -0.00559 |  | -0.63037 | 54 |  |  |
| -0.63034 | 125.9316 | -0.00557 |  | -0.62927 | 55 |  |  |
| -0.62927 | 126.9997 | -0.00555 |  | -0.62827 | 56 |  |  |
| -0.6282 | 128.0678 | -0.00552 |  | -0.62723 | 57 |  |  |
| -0.62714 | 129.1359 | -0.0055 |  | -0.62604 | 58 |  |  |
| -0.62607 | 130.204 | -0.00547 |  | -0.62506 | 59 |  |  |
| -0.625 | 131.2721 | -0.00545 |  | -0.62408 | 60 |  |  |
| -0.62393 | 132.3403 | -0.00542 |  | -0.62299 | 61 |  |  |
| -0.62286 | 133.4084 | -0.00539 |  | -0.62186 | 62 |  |  |
| -0.6218 | 134.4765 | -0.00537 |  | -0.62073 | 63 |  |  |
| -0.62073 | 135.5446 | -0.00534 |  | -0.61984 | 64 |  |  |
| -0.61966 | 136.6127 | -0.00531 |  | -0.61871 | 65 |  |  |
| -0.61859 | 137.6808 | -0.00528 |  | -0.61765 | 66 |  |  |
| -0.61752 | 138.7489 | -0.00525 |  | -0.61649 | 67 |  |  |
| -0.61646 | 139.8171 | -0.00522 |  | -0.61548 | 68 |  |  |
| -0.61539 | 140.8852 | -0.00519 |  | -0.61447 | 69 |  |  |
| -0.61432 | 141.9533 | -0.00516 |  | -0.6134 | 70 |  |  |
| -0.61325 | 143.0214 | -0.00513 |  | -0.6123 | 71 |  |  |
| -0.61218 | 144.0895 | -0.00509 |  | -0.61121 | 72 |  |  |
| -0.61111 | 145.1576 | -0.00506 |  | -0.6102 | 73 |  |  |
| -0.61005 | 146.2257 | -0.00502 |  | -0.60922 | 74 |  |  |
| -0.60898 | 147.2939 | -0.00498 |  | -0.60806 | 75 |  |  |
| -0.60791 | 148.362 | -0.00494 |  | -0.60712 | 76 |  |  |
| -0.60684 | 149.4301 | -0.0049 |  | -0.60605 | 77 |  |  |
| -0.60577 | 150.4982 | -0.00486 |  | -0.60492 | 78 |  |  |
| -0.60471 | 151.5663 | -0.00481 |  | -0.60394 | 79 |  |  |
| -0.60364 | 152.6344 | -0.00476 |  | -0.60284 | 80 |  |  |
| -0.60257 | 153.7026 | -0.00471 |  | -0.60172 | 81 |  |  |
| -0.6015 | 154.7707 | -0.00466 |  | -0.60074 | 82 |  |  |
| -0.60043 | 155.8388 | -0.0046 |  | -0.59964 | 83 |  |  |
| -0.59937 | 156.9069 | -0.00454 |  | -0.5986 | 84 |  |  |
| -0.5983 | 157.975 | -0.00448 |  | -0.5975 | 85 |  |  |
| -0.59723 | 159.0431 | -0.00441 |  | -0.59647 | 86 |  |  |
| -0.59616 | 160.1112 | -0.00434 |  | -0.59543 | 87 |  |  |
| -0.59509 | 161.1794 | -0.00427 |  | -0.59442 | 88 |  |  |
| -0.59402 | 162.2475 | -0.00419 |  | -0.59323 | 89 |  |  |
| -0.59296 | 163.3156 | -0.00411 |  | -0.59225 | 90 |  |  |
| -0.59189 | 164.3837 | -0.00402 |  | -0.59116 | 91 |  |  |
| -0.59082 | 165.4518 | -0.00394 |  | -0.59012 | 92 |  |  |
| -0.58975 | 166.5199 | -0.00385 |  | -0.58914 | 93 |  |  |
| -0.58868 | 167.588 | -0.00376 |  | -0.58807 | 94 |  |  |
| -0.58762 | 168.6562 | -0.00367 |  | -0.58707 | 95 |  |  |
| -0.58655 | 169.7243 | -0.00358 |  | -0.58606 | 96 |  |  |
| -0.58548 | 170.7924 | -0.00349 |  | -0.58505 | 97 |  |  |
| -0.58441 | 171.8605 | -0.0034 |  | -0.58389 | 98 |  |  |
| -0.58334 | 172.9286 | -0.00331 |  | -0.58282 | 99 |  |  |
| -0.58228 | 173.9967 | -0.00323 |  | -0.58185 | 100 |  |  |
| -0.58121 | 175.0649 | -0.00314 |  | -0.58078 | 101 |  |  |
| -0.58014 | 176.133 | -0.00306 |  | -0.57959 | 102 |  |  |
| -0.57907 | 177.2011 | -0.00298 |  | -0.57864 | 103 |  |  |
| -0.578 | 178.2692 | -0.0029 |  | -0.57751 | 104 |  |  |
| -0.57693 | 179.3373 | -0.00283 |  | -0.57663 | 105 |  |  |
| -0.57587 | 180.4054 | -0.00275 |  | -0.57556 | 106 |  |  |
| -0.5748 | 181.4735 | -0.00268 |  | -0.57446 | 107 |  |  |
| -0.57373 | 182.5417 | -0.00261 |  | -0.57346 | 108 |  |  |
| -0.57266 | 183.6098 | -0.00254 |  | -0.57242 | 109 |  |  |
| -0.57159 | 184.6779 | -0.00247 |  | -0.57132 | 110 |  |  |
| -0.57053 | 185.746 | -0.00241 |  | -0.57028 | 111 |  |  |
| -0.56946 | 186.8141 | -0.00234 |  | -0.56921 | 112 |  |  |
| -0.56839 | 187.8822 | -0.00228 |  | -0.56815 | 113 |  |  |
| -0.56732 | 188.9503 | -0.00222 |  | -0.56726 | 114 |  |  |
| -0.56625 | 190.0185 | -0.00216 |  | -0.56598 | 115 |  |  |
| -0.56519 | 191.0866 | -0.0021 |  | -0.56503 | 116 |  |  |
| -0.56412 | 192.1547 | -0.00205 |  | -0.564 | 117 |  |  |
| -0.56305 | 193.2228 | -0.00199 |  | -0.56287 | 118 |  |  |
| -0.56198 | 194.2909 | -0.00194 |  | -0.56195 | 119 |  |  |
| -0.56091 | 195.359 | -0.00189 |  | -0.56079 | 120 |  |  |
| -0.55984 | 196.4272 | -0.00184 |  | -0.55975 | 121 |  |  |
| -0.55878 | 197.4953 | -0.00179 |  | -0.55862 | 122 |  |  |
| -0.55771 | 198.5634 | -0.00174 |  | -0.55762 | 123 |  |  |
| -0.55664 | 199.6315 | -0.00169 |  | -0.55652 | 124 |  |  |
| -0.55557 | 200.6996 | -0.00165 |  | -0.55545 | 125 |  |  |
| -0.5545 | 201.7677 | -0.0016 |  | -0.5545 | 126 |  |  |
| -0.55344 | 202.8358 | -0.00156 |  | -0.55341 | 127 |  |  |
| -0.55237 | 203.904 | -0.00152 |  | -0.55225 | 128 |  |  |
| -0.5513 | 204.9721 | -0.00148 |  | -0.55127 | 129 |  |  |
| -0.55023 | 206.0402 | -0.00144 |  | -0.55029 | 130 |  |  |
| -0.54916 | 207.1083 | -0.0014 |  | -0.54916 | 131 |  |  |
| -0.5481 | 208.1764 | -0.00136 |  | -0.5481 | 132 |  |  |
| -0.54703 | 209.2445 | -0.00132 |  | -0.547 | 133 |  |  |
| -0.54596 | 210.3126 | -0.00129 |  | -0.54602 | 134 |  |  |
| -0.54489 | 211.3808 | -0.00125 |  | -0.54489 | 135 |  |  |
| -0.54382 | 212.4489 | -0.00122 |  | -0.54385 | 136 |  |  |
| -0.54276 | 213.517 | -0.00119 |  | -0.54294 | 137 |  |  |
| -0.54169 | 214.5851 | -0.00115 |  | -0.54166 | 138 |  |  |
| -0.54062 | 215.6532 | -0.00112 |  | -0.54065 | 139 |  |  |
| -0.53955 | 216.7213 | -0.00109 |  | -0.53961 | 140 |  |  |
| -0.53848 | 217.7895 | -0.00106 |  | -0.53845 | 141 |  |  |
| -0.53741 | 218.8576 | -0.00103 |  | -0.53751 | 142 |  |  |
| -0.53635 | 219.9257 | -0.001 |  | -0.53638 | 143 |  |  |
| -0.53528 | 220.9938 | -0.00098 |  | -0.53531 | 144 |  |  |
| -0.53421 | 222.0619 | -0.00095 |  | -0.53433 | 145 |  |  |
| -0.53314 | 223.13 | -0.00093 |  | -0.53323 | 146 |  |  |
| -0.53207 | 224.1981 | -0.0009 |  | -0.53214 | 147 |  |  |
| -0.53101 | 225.2663 | -0.00088 |  | -0.53101 | 148 |  |  |
| -0.52994 | 226.3344 | -0.00085 |  | -0.53006 | 149 |  |  |
| -0.52887 | 227.4025 | -0.00083 |  | -0.5289 | 150 |  |  |
| -0.5278 | 228.4706 | -0.00081 |  | -0.52783 | 151 |  |  |
| -0.52673 | 229.5387 | -0.00078 |  | -0.52673 | 152 |  |  |
| -0.52567 | 230.6068 | -0.00076 |  | -0.52573 | 153 |  |  |
| -0.5246 | 231.6749 | -0.00074 |  | -0.52463 | 154 |  |  |
| -0.52353 | 232.7431 | -0.00072 |  | -0.52365 | 155 |  |  |
| -0.52246 | 233.8112 | -0.0007 |  | -0.52258 | 156 |  |  |
| -0.52139 | 234.8793 | -0.00068 |  | -0.52139 | 157 |  |  |
| -0.52032 | 235.9474 | -0.00066 |  | -0.52039 | 158 |  |  |
| -0.51926 | 237.0155 | -0.00064 |  | -0.51938 | 159 |  |  |
| -0.51819 | 238.0836 | -0.00063 |  | -0.51822 | 160 |  |  |
| -0.51712 | 239.1518 | -0.00061 |  | -0.51727 | 161 |  |  |
| -0.51605 | 240.2199 | -0.00059 |  | -0.51611 | 162 |  |  |
| -0.51498 | 241.288 | -0.00058 |  | -0.51511 | 163 |  |  |
| -0.51392 | 242.3561 | -0.00056 |  | -0.51404 | 164 |  |  |
| -0.51285 | 243.4242 | -0.00054 |  | -0.51294 | 165 |  |  |
| -0.51178 | 244.4923 | -0.00053 |  | -0.51196 | 166 |  |  |
| -0.51071 | 245.5604 | -0.00051 |  | -0.5108 | 167 |  |  |
| -0.50964 | 246.6286 | -0.0005 |  | -0.50983 | 168 |  |  |
| -0.50858 | 247.6967 | -0.00049 |  | -0.50867 | 169 |  |  |
| -0.50751 | 248.7648 | -0.00047 |  | -0.50757 | 170 |  |  |
| -0.50644 | 249.8329 | -0.00046 |  | -0.50644 | 171 |  |  |
| -0.50537 | 250.901 | -0.00045 |  | -0.50543 | 172 |  |  |
| -0.5043 | 251.9691 | -0.00043 |  | -0.50439 | 173 |  |  |
| -0.50323 | 253.0372 | -0.00042 |  | -0.5032 | 174 |  |  |
| -0.50217 | 254.1054 | -0.00041 |  | -0.50226 | 175 |  |  |
| -0.5011 | 255.1735 | -0.0004 |  | -0.50119 | 176 |  |  |
| -0.50003 | 256.2416 | -0.00039 |  | -0.50015 | 177 |  |  |
| -0.49896 | 257.3097 | -0.00037 |  | -0.49896 | 178 |  |  |
| -0.49789 | 258.3778 | -0.00036 |  | -0.49792 | 179 |  |  |
| -0.49683 | 259.4459 | -0.00035 |  | -0.49704 | 180 |  |  |
| -0.49576 | 260.5141 | -0.00034 |  | -0.49588 | 181 |  |  |
| -0.49469 | 261.5822 | -0.00033 |  | -0.49481 | 182 |  |  |
| -0.49362 | 262.6503 | -0.00032 |  | -0.49377 | 183 |  |  |
| -0.49255 | 263.7184 | -0.00031 |  | -0.49268 | 184 |  |  |
| -0.49149 | 264.7865 | -0.0003 |  | -0.4917 | 185 |  |  |
| -0.49042 | 265.8546 | -0.00029 |  | -0.49051 | 186 |  |  |
| -0.48935 | 266.9227 | -0.00029 |  | -0.48944 | 187 |  |  |
| -0.48828 | 267.9909 | -0.00028 |  | -0.4884 | 188 |  |  |
| -0.48721 | 269.059 | -0.00027 |  | -0.48727 | 189 |  |  |
| -0.48615 | 270.1271 | -0.00026 |  | -0.48611 | 190 |  |  |
| -0.48508 | 271.1952 | -0.00025 |  | -0.48514 | 191 |  |  |
| -0.48401 | 272.2633 | -0.00024 |  | -0.48413 | 192 |  |  |
| -0.48294 | 273.3314 | -0.00024 |  | -0.48306 | 193 |  |  |
| -0.48187 | 274.3995 | -0.00023 |  | -0.48193 | 194 |  |  |
| -0.4808 | 275.4677 | -0.00022 |  | -0.48093 | 195 |  |  |
| -0.47974 | 276.5358 | -0.00021 |  | -0.47986 | 196 |  |  |
| -0.47867 | 277.6039 | -0.00021 |  | -0.47876 | 197 |  |  |
| -0.4776 | 278.672 | -0.0002 |  | -0.47769 | 198 |  |  |
| -0.47653 | 279.7401 | -0.00019 |  | -0.47668 | 199 |  |  |
| -0.47546 | 280.8082 | -0.00019 |  | -0.47562 | 200 |  |  |
| -0.4744 | 281.8764 | -0.00018 |  | -0.47458 | 201 |  |  |
| -0.47333 | 282.9445 | -0.00017 |  | -0.47336 | 202 |  |  |
| -0.47226 | 284.0126 | -0.00017 |  | -0.47232 | 203 |  |  |
| -0.47119 | 285.0807 | -0.00016 |  | -0.47137 | 204 |  |  |
| -0.47012 | 286.1488 | -0.00016 |  | -0.47028 | 205 |  |  |
| -0.46906 | 287.2169 | -0.00015 |  | -0.46912 | 206 |  |  |
| -0.46799 | 288.285 | -0.00014 |  | -0.46805 | 207 |  |  |
| -0.46692 | 289.3532 | -0.00014 |  | -0.46707 | 208 |  |  |
| -0.46585 | 290.4213 | -0.00013 |  | -0.46591 | 209 |  |  |
| -0.46478 | 291.4894 | -0.00013 |  | -0.46503 | 210 |  |  |
| -0.46371 | 292.5575 | -0.00012 |  | -0.46381 | 211 |  |  |
| -0.46265 | 293.6256 | -0.00012 |  | -0.46271 | 212 |  |  |
| -0.46158 | 294.6937 | -0.00011 |  | -0.46167 | 213 |  |  |
| -0.46051 | 295.7618 | -0.00011 |  | -0.46048 | 214 |  |  |
| -0.45944 | 296.83 | -0.0001 |  | -0.45963 | 215 |  |  |
| -0.45837 | 297.8981 | -9.6E-05 |  | -0.45859 | 216 |  |  |
| -0.45731 | 298.9662 | -9.1E-05 |  | -0.45746 | 217 |  |  |
| -0.45624 | 300.0343 | -8.7E-05 |  | -0.45633 | 218 |  |  |
| -0.45517 | 301.1024 | -8.2E-05 |  | -0.45532 | 219 |  |  |
| -0.4541 | 302.1705 | -7.7E-05 |  | -0.45422 | 220 |  |  |
| -0.45303 | 303.2387 | -7.3E-05 |  | -0.45306 | 221 |  |  |
| -0.45197 | 304.3068 | -6.8E-05 |  | -0.45187 | 222 |  |  |
| -0.4509 | 305.3749 | -6.4E-05 |  | -0.45093 | 223 |  |  |
| -0.44983 | 306.443 | -5.9E-05 |  | -0.44995 | 224 |  |  |
| -0.44876 | 307.5111 | -5.5E-05 |  | -0.44882 | 225 |  |  |
| -0.44769 | 308.5792 | -5.1E-05 |  | -0.44778 | 226 |  |  |
| -0.44662 | 309.6473 | -4.6E-05 |  | -0.44681 | 227 |  |  |
| -0.44556 | 310.7155 | -4.2E-05 |  | -0.4455 | 228 |  |  |
| -0.44449 | 311.7836 | -3.8E-05 |  | -0.44461 | 229 |  |  |
| -0.44342 | 312.8517 | -3.4E-05 |  | -0.44345 | 230 |  |  |
| -0.44235 | 313.9198 | -3E-05 |  | -0.44247 | 231 |  |  |
| -0.44128 | 314.9879 | -2.6E-05 |  | -0.44141 | 232 |  |  |
| -0.44022 | 316.056 | -2.2E-05 |  | -0.44037 | 233 |  |  |
| -0.43915 | 317.1241 | -1.7E-05 |  | -0.43921 | 234 |  |  |
| -0.43808 | 318.1923 | -1.3E-05 |  | -0.43811 | 235 |  |  |
| -0.43701 | 319.2604 | -9.2E-06 |  | -0.43707 | 236 |  |  |
| -0.43594 | 320.3285 | -5.3E-06 |  | -0.43604 | 237 |  |  |
| -0.43488 | 321.3966 | -1.3E-06 |  | -0.43491 | 238 |  |  |
| -0.43381 | 322.4647 | 2.77E-06 |  | -0.4339 | 239 |  |  |
| -0.43274 | 323.5328 | 6.99E-06 |  | -0.43283 | 240 |  |  |
| -0.43167 | 324.601 | 1.09E-05 |  | -0.43173 | 241 |  |  |
| -0.4306 | 325.6691 | 1.51E-05 |  | -0.43069 | 242 |  |  |
| -0.42953 | 326.7372 | 1.92E-05 |  | -0.43048 | 243 |  |  |
| -0.42847 | 327.8053 | 2.31E-05 |  | -0.42914 | 244 |  |  |
| -0.4274 | 328.8734 | 2.7E-05 |  | -0.42844 | 245 |  |  |
| -0.42633 | 329.9415 | 3.12E-05 |  | -0.427 | 246 |  |  |
| -0.42526 | 331.0096 | 3.52E-05 |  | -0.42542 | 247 |  |  |
| -0.42419 | 332.0778 | 3.96E-05 |  | -0.42441 | 248 |  |  |
| -0.42313 | 333.1459 | 4.39E-05 |  | -0.42331 | 249 |  |  |
| -0.42206 | 334.214 | 4.82E-05 |  | -0.42233 | 250 |  |  |
| -0.42099 | 335.2821 | 5.26E-05 |  | -0.42126 | 251 |  |  |
| -0.41992 | 336.3502 | 5.7E-05 |  | -0.41998 | 252 |  |  |
| -0.41885 | 337.4183 | 6.13E-05 |  | -0.41907 | 253 |  |  |
| -0.41779 | 338.4864 | 6.58E-05 |  | -0.418 | 254 |  |  |
| -0.41672 | 339.5546 | 7.05E-05 |  | -0.41708 | 255 |  |  |
| -0.41565 | 340.6227 | 7.49E-05 |  | -0.41586 | 256 |  |  |
| -0.41458 | 341.6908 | 7.97E-05 |  | -0.41479 | 257 |  |  |
| -0.41351 | 342.7589 | 8.44E-05 |  | -0.41382 | 258 |  |  |
| -0.41245 | 343.827 | 8.93E-05 |  | -0.41266 | 259 |  |  |
| -0.41138 | 344.8951 | 9.44E-05 |  | -0.41165 | 260 |  |  |
| -0.41031 | 345.9633 | 9.95E-05 |  | -0.41071 | 261 |  |  |
| -0.40924 | 347.0314 | 0.000105 |  | -0.40958 | 262 |  |  |
| -0.40817 | 348.0995 | 0.00011 |  | -0.40836 | 263 |  |  |
| -0.4071 | 349.1676 | 0.000115 |  | -0.40738 | 264 |  |  |
| -0.40604 | 350.2357 | 0.000121 |  | -0.40637 | 265 |  |  |
| -0.40497 | 351.3038 | 0.000127 |  | -0.40518 | 266 |  |  |
| -0.4039 | 352.3719 | 0.000133 |  | -0.40427 | 267 |  |  |
| -0.40283 | 353.4401 | 0.000139 |  | -0.40323 | 268 |  |  |
| -0.40176 | 354.5082 | 0.000145 |  | -0.40213 | 269 |  |  |
| -0.4007 | 355.5763 | 0.000151 |  | -0.40112 | 270 |  |  |
| -0.39963 | 356.6444 | 0.000158 |  | -0.3999 | 271 |  |  |
| -0.39856 | 357.7125 | 0.000165 |  | -0.39883 | 272 |  |  |
| -0.39749 | 358.7806 | 0.000172 |  | -0.39777 | 273 |  |  |
| -0.39642 | 359.8487 | 0.000179 |  | -0.39691 | 274 |  |  |
| -0.39536 | 360.9169 | 0.000186 |  | -0.39581 | 275 |  |  |
| -0.39429 | 361.985 | 0.000194 |  | -0.39462 | 276 |  |  |
| -0.39322 | 363.0531 | 0.000202 |  | -0.39349 | 277 |  |  |
| -0.39215 | 364.1212 | 0.00021 |  | -0.39255 | 278 |  |  |
| -0.39108 | 365.1893 | 0.000218 |  | -0.39142 | 279 |  |  |
| -0.39001 | 366.2574 | 0.000227 |  | -0.39053 | 280 |  |  |
| -0.38895 | 367.3256 | 0.000236 |  | -0.38937 | 281 |  |  |
| -0.38788 | 368.3937 | 0.000245 |  | -0.38849 | 282 |  |  |
| -0.38681 | 369.4618 | 0.000255 |  | -0.38727 | 283 |  |  |
| -0.38574 | 370.5299 | 0.000265 |  | -0.38632 | 284 |  |  |
| -0.38467 | 371.598 | 0.000275 |  | -0.38519 | 285 |  |  |
| -0.38361 | 372.6661 | 0.000286 |  | -0.38406 | 286 |  |  |
| -0.38254 | 373.7342 | 0.000298 |  | -0.38303 | 287 |  |  |
| -0.38147 | 374.8024 | 0.00031 |  | -0.3822 | 288 |  |  |
| -0.3804 | 375.8705 | 0.000322 |  | -0.38058 | 289 |  |  |
| -0.37933 | 376.9386 | 0.000335 |  | -0.37943 | 290 |  |  |
| -0.37827 | 378.0067 | 0.000348 |  | -0.37839 | 291 |  |  |
| -0.3772 | 379.0748 | 0.000362 |  | -0.37744 | 292 |  |  |
| -0.37613 | 380.1429 | 0.000377 |  | -0.37622 | 293 |  |  |
| -0.37506 | 381.211 | 0.000392 |  | -0.37518 | 294 |  |  |
| -0.37399 | 382.2792 | 0.000408 |  | -0.37421 | 295 |  |  |
| -0.37292 | 383.3473 | 0.000425 |  | -0.37311 | 296 |  |  |
| -0.37186 | 384.4154 | 0.000442 |  | -0.37195 | 297 |  |  |
| -0.37079 | 385.4835 | 0.00046 |  | -0.371 | 298 |  |  |
| -0.36972 | 386.5516 | 0.00048 |  | -0.36984 | 299 |  |  |
| -0.36865 | 387.6197 | 0.000499 |  | -0.36887 | 300 |  |  |
| -0.36758 | 388.6879 | 0.00052 |  | -0.36789 | 301 |  |  |
| -0.36652 | 389.756 | 0.000542 |  | -0.36679 | 302 |  |  |
| -0.36545 | 390.8241 | 0.000565 |  | -0.36575 | 303 |  |  |
| -0.36438 | 391.8922 | 0.000589 |  | -0.36462 | 304 |  |  |
| -0.36331 | 392.9603 | 0.000614 |  | -0.36356 | 305 |  |  |
| -0.36224 | 394.0284 | 0.00064 |  | -0.36243 | 306 |  |  |
| -0.36118 | 395.0965 | 0.000668 |  | -0.36139 | 307 |  |  |
| -0.36011 | 396.1647 | 0.000697 |  | -0.36032 | 308 |  |  |
| -0.35904 | 397.2328 | 0.000728 |  | -0.35931 | 309 |  |  |
| -0.35797 | 398.3009 | 0.00076 |  | -0.35831 | 310 |  |  |
| -0.3569 | 399.369 | 0.000794 |  | -0.35718 | 311 |  |  |
| -0.35583 | 400.4371 | 0.00083 |  | -0.35605 | 312 |  |  |
| -0.35477 | 401.5052 | 0.000867 |  | -0.35495 | 313 |  |  |
| -0.3537 | 402.5733 | 0.000907 |  | -0.354 | 314 |  |  |
| -0.35263 | 403.6415 | 0.000948 |  | -0.35284 | 315 |  |  |
| -0.35156 | 404.7096 | 0.000992 |  | -0.35172 | 316 |  |  |
| -0.35049 | 405.7777 | 0.001038 |  | -0.35077 | 317 |  |  |
| -0.34943 | 406.8458 | 0.001086 |  | -0.34973 | 318 |  |  |
| -0.34836 | 407.9139 | 0.001138 |  | -0.34866 | 319 |  |  |
| -0.34729 | 408.982 | 0.001192 |  | -0.34766 | 320 |  |  |
| -0.34622 | 410.0502 | 0.001249 |  | -0.34662 | 321 |  |  |
| -0.34515 | 411.1183 | 0.00131 |  | -0.3454 | 322 |  |  |
| -0.34409 | 412.1864 | 0.001373 |  | -0.34442 | 323 |  |  |
| -0.34302 | 413.2545 | 0.00144 |  | -0.34332 | 324 |  |  |
| -0.34195 | 414.3226 | 0.001512 |  | -0.34225 | 325 |  |  |
| -0.34088 | 415.3907 | 0.001587 |  | -0.34125 | 326 |  |  |
| -0.33981 | 416.4588 | 0.001666 |  | -0.34003 | 327 |  |  |
| -0.33875 | 417.527 | 0.001752 |  | -0.33911 | 328 |  |  |
| -0.33768 | 418.5951 | 0.001843 |  | -0.33804 | 329 |  |  |
| -0.33661 | 419.6632 | 0.001938 |  | -0.33694 | 330 |  |  |
| -0.33554 | 420.7313 | 0.00204 |  | -0.33597 | 331 |  |  |
| -0.33447 | 421.7994 | 0.002147 |  | -0.33487 | 332 |  |  |
| -0.3334 | 422.8675 | 0.002259 |  | -0.33377 | 333 |  |  |
| -0.33234 | 423.9356 | 0.002378 |  | -0.33279 | 334 |  |  |
| -0.33127 | 425.0038 | 0.002503 |  | -0.3316 | 335 |  |  |
| -0.3302 | 426.0719 | 0.002635 |  | -0.33078 | 336 |  |  |
| -0.32913 | 427.14 | 0.002773 |  | -0.32962 | 337 |  |  |
| -0.32806 | 428.2081 | 0.002922 |  | -0.32864 | 338 |  |  |
| -0.327 | 429.2762 | 0.003079 |  | -0.32755 | 339 |  |  |
| -0.32593 | 430.3443 | 0.003248 |  | -0.32645 | 340 |  |  |
| -0.32486 | 431.4125 | 0.003423 |  | -0.32538 | 341 |  |  |
| -0.32379 | 432.4806 | 0.003605 |  | -0.32449 | 342 |  |  |
| -0.32272 | 433.5487 | 0.003793 |  | -0.32346 | 343 |  |  |
| -0.32166 | 434.6168 | 0.003981 |  | -0.32233 | 344 |  |  |
| -0.32059 | 435.6849 | 0.00416 |  | -0.32135 | 345 |  |  |
| -0.31952 | 436.753 | 0.004325 |  | -0.32028 | 346 |  |  |
| -0.31845 | 437.8211 | 0.004474 |  | -0.31931 | 347 |  |  |
| -0.31738 | 438.8893 | 0.004606 |  | -0.31833 | 348 |  |  |
| -0.31631 | 439.9574 | 0.004725 |  | -0.31717 | 349 |  |  |
| -0.31525 | 441.0255 | 0.004832 |  | -0.31619 | 350 |  |  |
| -0.31418 | 442.0936 | 0.00493 |  | -0.31512 | 351 |  |  |
| -0.31311 | 443.1617 | 0.005017 |  | -0.31409 | 352 |  |  |
| -0.31204 | 444.2298 | 0.005101 |  | -0.31311 | 353 |  |  |
| -0.31097 | 445.2979 | 0.005178 |  | -0.31195 | 354 |  |  |
| -0.30991 | 446.3661 | 0.00525 |  | -0.31091 | 355 |  |  |
| -0.30884 | 447.4342 | 0.005319 |  | -0.30984 | 356 |  |  |
| -0.30777 | 448.5023 | 0.005385 |  | -0.30869 | 357 |  |  |
| -0.3067 | 449.5704 | 0.005447 |  | -0.30777 | 358 |  |  |
| -0.30563 | 450.6385 | 0.005507 |  | -0.30676 | 359 |  |  |
| -0.30457 | 451.7066 | 0.005565 |  | -0.30554 | 360 |  |  |
| -0.3035 | 452.7748 | 0.00562 |  | -0.30453 | 361 |  |  |
| -0.30243 | 453.8429 | 0.005674 |  | -0.30344 | 362 |  |  |
| -0.30136 | 454.911 | 0.005727 |  | -0.30258 | 363 |  |  |
| -0.30029 | 455.9791 | 0.005777 |  | -0.30142 | 364 |  |  |
| -0.29922 | 457.0472 | 0.005827 |  | -0.30029 | 365 |  |  |
| -0.29816 | 458.1153 | 0.005874 |  | -0.29932 | 366 |  |  |
| -0.29709 | 459.1834 | 0.005921 |  | -0.29816 | 367 |  |  |
| -0.29602 | 460.2516 | 0.005966 |  | -0.29724 | 368 |  |  |
| -0.29495 | 461.3197 | 0.00601 |  | -0.29614 | 369 |  |  |
| -0.29388 | 462.3878 | 0.006053 |  | -0.29514 | 370 |  |  |
| -0.29282 | 463.4559 | 0.006094 |  | -0.29398 | 371 |  |  |
| -0.29175 | 464.524 | 0.006134 |  | -0.293 | 372 |  |  |
| -0.29068 | 465.5921 | 0.006173 |  | -0.29196 | 373 |  |  |
| -0.28961 | 466.6602 | 0.006211 |  | -0.2908 | 374 |  |  |
| -0.28854 | 467.7284 | 0.006248 |  | -0.28986 | 375 |  |  |
| -0.28748 | 468.7965 | 0.006284 |  | -0.28876 | 376 |  |  |
| -0.28641 | 469.8646 | 0.006318 |  | -0.28766 | 377 |  |  |
| -0.28534 | 470.9327 | 0.006352 |  | -0.28659 | 378 |  |  |
| -0.28427 | 472.0008 | 0.006384 |  | -0.28549 | 379 |  |  |
| -0.2832 | 473.0689 | 0.006415 |  | -0.28448 | 380 |  |  |
| -0.28214 | 474.1371 | 0.006445 |  | -0.28339 | 381 |  |  |
| -0.28107 | 475.2052 | 0.006475 |  | -0.28235 | 382 |  |  |
| -0.28 | 476.2733 | 0.006503 |  | -0.28122 | 383 |  |  |
| -0.27893 | 477.3414 | 0.006531 |  | -0.2803 | 384 |  |  |
| -0.27786 | 478.4095 | 0.006557 |  | -0.27921 | 385 |  |  |
| -0.27679 | 479.4776 | 0.006583 |  | -0.27808 | 386 |  |  |
| -0.27573 | 480.5457 | 0.006608 |  | -0.27707 | 387 |  |  |
| -0.27466 | 481.6139 | 0.006631 |  | -0.276 | 388 |  |  |
| -0.27359 | 482.682 | 0.006655 |  | -0.27505 | 389 |  |  |
| -0.27252 | 483.7501 | 0.006677 |  | -0.27393 | 390 |  |  |
| -0.27145 | 484.8182 | 0.006699 |  | -0.2728 | 391 |  |  |
| -0.27039 | 485.8863 | 0.006721 |  | -0.27176 | 392 |  |  |
| -0.26932 | 486.9544 | 0.006741 |  | -0.27069 | 393 |  |  |
| -0.26825 | 488.0225 | 0.006761 |  | -0.26965 | 394 |  |  |
| -0.26718 | 489.0907 | 0.006781 |  | -0.26859 | 395 |  |  |
| -0.26611 | 490.1588 | 0.0068 |  | -0.26749 | 396 |  |  |
| -0.26505 | 491.2269 | 0.006819 |  | -0.26636 | 397 |  |  |
| -0.26398 | 492.295 | 0.006837 |  | -0.26547 | 398 |  |  |
| -0.26291 | 493.3631 | 0.006855 |  | -0.26425 | 399 |  |  |
| -0.26184 | 494.4312 | 0.006872 |  | -0.26321 | 400 |  |  |
| -0.26077 | 495.4994 | 0.006889 |  | -0.26221 | 401 |  |  |
| -0.2597 | 496.5675 | 0.006906 |  | -0.26117 | 402 |  |  |
| -0.25864 | 497.6356 | 0.006922 |  | -0.25995 | 403 |  |  |
| -0.25757 | 498.7037 | 0.006938 |  | -0.25909 | 404 |  |  |
| -0.2565 | 499.7718 | 0.006954 |  | -0.25797 | 405 |  |  |
| -0.25543 | 500.8399 | 0.006969 |  | -0.25681 | 406 |  |  |
| -0.25436 | 501.908 | 0.006984 |  | -0.25577 | 407 |  |  |
| -0.2533 | 502.9762 | 0.006999 |  | -0.2547 | 408 |  |  |
| -0.25223 | 504.0443 | 0.007013 |  | -0.25369 | 409 |  |  |
| -0.25116 | 505.1124 | 0.007028 |  | -0.2525 | 410 |  |  |
| -0.25009 | 506.1805 | 0.007042 |  | -0.25153 | 411 |  |  |
| -0.24902 | 507.2486 | 0.007055 |  | -0.25055 | 412 |  |  |
| -0.24796 | 508.3167 | 0.007069 |  | -0.24942 | 413 |  |  |
| -0.24689 | 509.3848 | 0.007083 |  | -0.24841 | 414 |  |  |
| -0.24582 | 510.453 | 0.007095 |  | -0.24731 | 415 |  |  |
| -0.24475 | 511.5211 | 0.007108 |  | -0.24628 | 416 |  |  |
| -0.24368 | 512.5892 | 0.007121 |  | -0.24521 | 417 |  |  |
| -0.24261 | 513.6573 | 0.007134 |  | -0.24411 | 418 |  |  |
| -0.24155 | 514.7254 | 0.007146 |  | -0.2431 | 419 |  |  |
| -0.24048 | 515.7935 | 0.007159 |  | -0.24194 | 420 |  |  |
| -0.23941 | 516.8617 | 0.00717 |  | -0.24091 | 421 |  |  |
| -0.23834 | 517.9298 | 0.007182 |  | -0.23996 | 422 |  |  |
| -0.23727 | 518.9979 | 0.007194 |  | -0.23886 | 423 |  |  |
| -0.23621 | 520.066 | 0.007206 |  | -0.2377 | 424 |  |  |
| -0.23514 | 521.1341 | 0.007217 |  | -0.23657 | 425 |  |  |
| -0.23407 | 522.2022 | 0.007228 |  | -0.23563 | 426 |  |  |
| -0.233 | 523.2703 | 0.007239 |  | -0.23453 | 427 |  |  |
| -0.23193 | 524.3385 | 0.00725 |  | -0.23349 | 428 |  |  |
| -0.23087 | 525.4066 | 0.007261 |  | -0.23248 | 429 |  |  |
| -0.2298 | 526.4747 | 0.007271 |  | -0.23132 | 430 |  |  |
| -0.22873 | 527.5428 | 0.007282 |  | -0.23026 | 431 |  |  |
| -0.22766 | 528.6109 | 0.007292 |  | -0.22913 | 432 |  |  |
| -0.22659 | 529.679 | 0.007302 |  | -0.22815 | 433 |  |  |
| -0.22552 | 530.7471 | 0.007312 |  | -0.22714 | 434 |  |  |
| -0.22446 | 531.8153 | 0.007322 |  | -0.22598 | 435 |  |  |
| -0.22339 | 532.8834 | 0.007332 |  | -0.22485 | 436 |  |  |
| -0.22232 | 533.9515 | 0.007342 |  | -0.22397 | 437 |  |  |
| -0.22125 | 535.0196 | 0.007351 |  | -0.22272 | 438 |  |  |
| -0.22018 | 536.0877 | 0.007361 |  | -0.22174 | 439 |  |  |
| -0.21912 | 537.1558 | 0.00737 |  | -0.2207 | 440 |  |  |
| -0.21805 | 538.224 | 0.007379 |  | -0.21957 | 441 |  |  |
| -0.21698 | 539.2921 | 0.007389 |  | -0.2186 | 442 |  |  |
| -0.21591 | 540.3602 | 0.007398 |  | -0.2175 | 443 |  |  |
| -0.21484 | 541.4283 | 0.007407 |  | -0.21652 | 444 |  |  |
| -0.21378 | 542.4964 | 0.007415 |  | -0.2153 | 445 |  |  |
| -0.21271 | 543.5645 | 0.007424 |  | -0.21429 | 446 |  |  |
| -0.21164 | 544.6326 | 0.007433 |  | -0.21317 | 447 |  |  |
| -0.21057 | 545.7008 | 0.007442 |  | -0.21216 | 448 |  |  |
| -0.2095 | 546.7689 | 0.00745 |  | -0.21103 | 449 |  |  |
| -0.20844 | 547.837 | 0.007458 |  | -0.20993 | 450 |  |  |
| -0.20737 | 548.9051 | 0.007467 |  | -0.20901 | 451 |  |  |
| -0.2063 | 549.9732 | 0.007475 |  | -0.20786 | 452 |  |  |
| -0.20523 | 551.0413 | 0.007483 |  | -0.20688 | 453 |  |  |
| -0.20416 | 552.1094 | 0.007491 |  | -0.2056 | 454 |  |  |
| -0.20309 | 553.1776 | 0.007499 |  | -0.20474 | 455 |  |  |
| -0.20203 | 554.2457 | 0.007507 |  | -0.20361 | 456 |  |  |
| -0.20096 | 555.3138 | 0.007515 |  | -0.20264 | 457 |  |  |
| -0.19989 | 556.3819 | 0.007523 |  | -0.20148 | 458 |  |  |
| -0.19882 | 557.45 | 0.00753 |  | -0.20047 | 459 |  |  |
| -0.19775 | 558.5181 | 0.007538 |  | -0.19934 | 460 |  |  |
| -0.19669 | 559.5863 | 0.007545 |  | -0.19833 | 461 |  |  |
| -0.19562 | 560.6544 | 0.007553 |  | -0.19708 | 462 |  |  |
| -0.19455 | 561.7225 | 0.00756 |  | -0.19608 | 463 |  |  |
| -0.19348 | 562.7906 | 0.007568 |  | -0.19507 | 464 |  |  |
| -0.19241 | 563.8587 | 0.007575 |  | -0.19406 | 465 |  |  |
| -0.19135 | 564.9268 | 0.007583 |  | -0.19296 | 466 |  |  |
| -0.19028 | 565.9949 | 0.00759 |  | -0.19193 | 467 |  |  |
| -0.18921 | 567.0631 | 0.007597 |  | -0.19083 | 468 |  |  |
| -0.18814 | 568.1312 | 0.007604 |  | -0.18979 | 469 |  |  |

Sample: *Marjoram*, Concentration (ppm): 800, Immersion time: 24h

| Potential applied(V) | Time (s) | WE(1).  Current (A) | WE(1).  Potential (V) | Index |  |  |
| --- | --- | --- | --- | --- | --- | --- |
| -0.69885 | 68.27635 | -0.00093 | -0.69684 | 1 |  |  |
| -0.69778 | 69.34447 | -0.00719 | -0.69635 | 2 |  |  |
| -0.69672 | 70.41258 | -0.00716 | -0.69528 | 3 |  |  |
| -0.69565 | 71.4807 | -0.00713 | -0.69431 | 4 |  |  |
| -0.69458 | 72.54881 | -0.00711 | -0.69315 | 5 |  |  |
| -0.69351 | 73.61693 | -0.00708 | -0.69211 | 6 |  |  |
| -0.69244 | 74.68504 | -0.00706 | -0.69095 | 7 |  |  |
| -0.69138 | 75.75316 | -0.00704 | -0.68991 | 8 |  |  |
| -0.69031 | 76.82127 | -0.00702 | -0.68906 | 9 |  |  |
| -0.68924 | 77.88939 | -0.007 | -0.68781 | 10 |  |  |
| -0.68817 | 78.9575 | -0.00698 | -0.68674 | 11 |  |  |
| -0.6871 | 80.02562 | -0.00696 | -0.68567 | 12 |  |  |
| -0.68604 | 81.09373 | -0.00694 | -0.68463 | 13 |  |  |
| -0.68497 | 82.16185 | -0.00692 | -0.68372 | 14 |  |  |
| -0.6839 | 83.22996 | -0.0069 | -0.68253 | 15 |  |  |
| -0.68283 | 84.29808 | -0.00688 | -0.68143 | 16 |  |  |
| -0.68176 | 85.36619 | -0.00686 | -0.68042 | 17 |  |  |
| -0.68069 | 86.43431 | -0.00684 | -0.67938 | 18 |  |  |
| -0.67963 | 87.50242 | -0.00683 | -0.67822 | 19 |  |  |
| -0.67856 | 88.57054 | -0.00681 | -0.67719 | 20 |  |  |
| -0.67749 | 89.63865 | -0.00679 | -0.67624 | 21 |  |  |
| -0.67642 | 90.70677 | -0.00677 | -0.67499 | 22 |  |  |
| -0.67535 | 91.77488 | -0.00675 | -0.67401 | 23 |  |  |
| -0.67429 | 92.843 | -0.00674 | -0.67288 | 24 |  |  |
| -0.67322 | 93.91111 | -0.00672 | -0.67194 | 25 |  |  |
| -0.67215 | 94.97923 | -0.0067 | -0.67087 | 26 |  |  |
| -0.67108 | 96.04734 | -0.00668 | -0.66974 | 27 |  |  |
| -0.67001 | 97.11546 | -0.00666 | -0.66879 | 28 |  |  |
| -0.66895 | 98.18357 | -0.00665 | -0.66772 | 29 |  |  |
| -0.66788 | 99.25169 | -0.00663 | -0.66669 | 30 |  |  |
| -0.66681 | 100.3198 | -0.00661 | -0.66553 | 31 |  |  |
| -0.66574 | 101.3879 | -0.00659 | -0.66452 | 32 |  |  |
| -0.66467 | 102.456 | -0.00657 | -0.66342 | 33 |  |  |
| -0.6636 | 103.5241 | -0.00656 | -0.66251 | 34 |  |  |
| -0.66254 | 104.5923 | -0.00654 | -0.66125 | 35 |  |  |
| -0.66147 | 105.6604 | -0.00652 | -0.66022 | 36 |  |  |
| -0.6604 | 106.7285 | -0.0065 | -0.65903 | 37 |  |  |
| -0.65933 | 107.7966 | -0.00648 | -0.65811 | 38 |  |  |
| -0.65826 | 108.8647 | -0.00647 | -0.65695 | 39 |  |  |
| -0.6572 | 109.9328 | -0.00645 | -0.65585 | 40 |  |  |
| -0.65613 | 111.001 | -0.00643 | -0.65494 | 41 |  |  |
| -0.65506 | 112.0691 | -0.00641 | -0.65381 | 42 |  |  |
| -0.65399 | 113.1372 | -0.00639 | -0.65277 | 43 |  |  |
| -0.65292 | 114.2053 | -0.00637 | -0.65179 | 44 |  |  |
| -0.65186 | 115.2734 | -0.00636 | -0.65079 | 45 |  |  |
| -0.65079 | 116.3415 | -0.00634 | -0.64963 | 46 |  |  |
| -0.64972 | 117.4096 | -0.00632 | -0.64862 | 47 |  |  |
| -0.64865 | 118.4778 | -0.0063 | -0.64752 | 48 |  |  |
| -0.64758 | 119.5459 | -0.00628 | -0.64655 | 49 |  |  |
| -0.64651 | 120.614 | -0.00626 | -0.64532 | 50 |  |  |
| -0.64545 | 121.6821 | -0.00624 | -0.64435 | 51 |  |  |
| -0.64438 | 122.7502 | -0.00622 | -0.64325 | 52 |  |  |
| -0.64331 | 123.8183 | -0.0062 | -0.64212 | 53 |  |  |
| -0.64224 | 124.8864 | -0.00618 | -0.64108 | 54 |  |  |
| -0.64117 | 125.9546 | -0.00617 | -0.64005 | 55 |  |  |
| -0.64011 | 127.0227 | -0.00615 | -0.63898 | 56 |  |  |
| -0.63904 | 128.0908 | -0.00613 | -0.63791 | 57 |  |  |
| -0.63797 | 129.1589 | -0.00611 | -0.6369 | 58 |  |  |
| -0.6369 | 130.227 | -0.00609 | -0.63583 | 59 |  |  |
| -0.63583 | 131.2951 | -0.00607 | -0.6347 | 60 |  |  |
| -0.63477 | 132.3633 | -0.00605 | -0.63367 | 61 |  |  |
| -0.6337 | 133.4314 | -0.00603 | -0.63257 | 62 |  |  |
| -0.63263 | 134.4995 | -0.00601 | -0.63162 | 63 |  |  |
| -0.63156 | 135.5676 | -0.00599 | -0.63052 | 64 |  |  |
| -0.63049 | 136.6357 | -0.00597 | -0.62946 | 65 |  |  |
| -0.62943 | 137.7038 | -0.00595 | -0.62845 | 66 |  |  |
| -0.62836 | 138.7719 | -0.00593 | -0.62735 | 67 |  |  |
| -0.62729 | 139.8401 | -0.00591 | -0.62637 | 68 |  |  |
| -0.62622 | 140.9082 | -0.00588 | -0.62512 | 69 |  |  |
| -0.62515 | 141.9763 | -0.00586 | -0.62427 | 70 |  |  |
| -0.62408 | 143.0444 | -0.00584 | -0.62302 | 71 |  |  |
| -0.62302 | 144.1125 | -0.00582 | -0.62201 | 72 |  |  |
| -0.62195 | 145.1806 | -0.0058 | -0.62094 | 73 |  |  |
| -0.62088 | 146.2487 | -0.00578 | -0.6199 | 74 |  |  |
| -0.61981 | 147.3169 | -0.00575 | -0.61877 | 75 |  |  |
| -0.61874 | 148.385 | -0.00573 | -0.61774 | 76 |  |  |
| -0.61768 | 149.4531 | -0.00571 | -0.61673 | 77 |  |  |
| -0.61661 | 150.5212 | -0.00569 | -0.61572 | 78 |  |  |
| -0.61554 | 151.5893 | -0.00566 | -0.61456 | 79 |  |  |
| -0.61447 | 152.6574 | -0.00564 | -0.61353 | 80 |  |  |
| -0.6134 | 153.7256 | -0.00561 | -0.6124 | 81 |  |  |
| -0.61234 | 154.7937 | -0.00559 | -0.61142 | 82 |  |  |
| -0.61127 | 155.8618 | -0.00557 | -0.61035 | 83 |  |  |
| -0.6102 | 156.9299 | -0.00554 | -0.60931 | 84 |  |  |
| -0.60913 | 157.998 | -0.00552 | -0.60818 | 85 |  |  |
| -0.60806 | 159.0661 | -0.00549 | -0.60709 | 86 |  |  |
| -0.60699 | 160.1342 | -0.00546 | -0.60593 | 87 |  |  |
| -0.60593 | 161.2024 | -0.00544 | -0.60498 | 88 |  |  |
| -0.60486 | 162.2705 | -0.00541 | -0.60394 | 89 |  |  |
| -0.60379 | 163.3386 | -0.00538 | -0.60284 | 90 |  |  |
| -0.60272 | 164.4067 | -0.00535 | -0.60181 | 91 |  |  |
| -0.60165 | 165.4748 | -0.00532 | -0.60083 | 92 |  |  |
| -0.60059 | 166.5429 | -0.00529 | -0.59976 | 93 |  |  |
| -0.59952 | 167.611 | -0.00526 | -0.5986 | 94 |  |  |
| -0.59845 | 168.6792 | -0.00523 | -0.5975 | 95 |  |  |
| -0.59738 | 169.7473 | -0.0052 | -0.59641 | 96 |  |  |
| -0.59631 | 170.8154 | -0.00516 | -0.5954 | 97 |  |  |
| -0.59525 | 171.8835 | -0.00513 | -0.59439 | 98 |  |  |
| -0.59418 | 172.9516 | -0.00509 | -0.59329 | 99 |  |  |
| -0.59311 | 174.0197 | -0.00506 | -0.59229 | 100 |  |  |
| -0.59204 | 175.0879 | -0.00502 | -0.59128 | 101 |  |  |
| -0.59097 | 176.156 | -0.00498 | -0.59018 | 102 |  |  |
| -0.5899 | 177.2241 | -0.00494 | -0.58905 | 103 |  |  |
| -0.58884 | 178.2922 | -0.00489 | -0.58795 | 104 |  |  |
| -0.58777 | 179.3603 | -0.00485 | -0.58685 | 105 |  |  |
| -0.5867 | 180.4284 | -0.0048 | -0.58594 | 106 |  |  |
| -0.58563 | 181.4965 | -0.00475 | -0.58481 | 107 |  |  |
| -0.58456 | 182.5647 | -0.0047 | -0.58389 | 108 |  |  |
| -0.5835 | 183.6328 | -0.00464 | -0.58273 | 109 |  |  |
| -0.58243 | 184.7009 | -0.00459 | -0.58173 | 110 |  |  |
| -0.58136 | 185.769 | -0.00452 | -0.58063 | 111 |  |  |
| -0.58029 | 186.8371 | -0.00446 | -0.57956 | 112 |  |  |
| -0.57922 | 187.9052 | -0.00439 | -0.57849 | 113 |  |  |
| -0.57816 | 188.9733 | -0.00431 | -0.57739 | 114 |  |  |
| -0.57709 | 190.0415 | -0.00424 | -0.57651 | 115 |  |  |
| -0.57602 | 191.1096 | -0.00416 | -0.57553 | 116 |  |  |
| -0.57495 | 192.1777 | -0.00407 | -0.57431 | 117 |  |  |
| -0.57388 | 193.2458 | -0.00399 | -0.57336 | 118 |  |  |
| -0.57281 | 194.3139 | -0.0039 | -0.57227 | 119 |  |  |
| -0.57175 | 195.382 | -0.00381 | -0.5712 | 120 |  |  |
| -0.57068 | 196.4502 | -0.00372 | -0.57019 | 121 |  |  |
| -0.56961 | 197.5183 | -0.00363 | -0.56906 | 122 |  |  |
| -0.56854 | 198.5864 | -0.00354 | -0.56796 | 123 |  |  |
| -0.56747 | 199.6545 | -0.00346 | -0.56708 | 124 |  |  |
| -0.56641 | 200.7226 | -0.00337 | -0.56601 | 125 |  |  |
| -0.56534 | 201.7907 | -0.00329 | -0.56497 | 126 |  |  |
| -0.56427 | 202.8588 | -0.0032 | -0.56384 | 127 |  |  |
| -0.5632 | 203.927 | -0.00312 | -0.56281 | 128 |  |  |
| -0.56213 | 204.9951 | -0.00304 | -0.56171 | 129 |  |  |
| -0.56107 | 206.0632 | -0.00296 | -0.56076 | 130 |  |  |
| -0.56 | 207.1313 | -0.00289 | -0.55969 | 131 |  |  |
| -0.55893 | 208.1994 | -0.00281 | -0.55862 | 132 |  |  |
| -0.55786 | 209.2675 | -0.00274 | -0.55768 | 133 |  |  |
| -0.55679 | 210.3356 | -0.00267 | -0.55655 | 134 |  |  |
| -0.55573 | 211.4038 | -0.0026 | -0.55545 | 135 |  |  |
| -0.55466 | 212.4719 | -0.00254 | -0.55438 | 136 |  |  |
| -0.55359 | 213.54 | -0.00247 | -0.55341 | 137 |  |  |
| -0.55252 | 214.6081 | -0.00241 | -0.55246 | 138 |  |  |
| -0.55145 | 215.6762 | -0.00235 | -0.55133 | 139 |  |  |
| -0.55038 | 216.7443 | -0.00228 | -0.5502 | 140 |  |  |
| -0.54932 | 217.8125 | -0.00223 | -0.54913 | 141 |  |  |
| -0.54825 | 218.8806 | -0.00217 | -0.5481 | 142 |  |  |
| -0.54718 | 219.9487 | -0.00211 | -0.54706 | 143 |  |  |
| -0.54611 | 221.0168 | -0.00206 | -0.54608 | 144 |  |  |
| -0.54504 | 222.0849 | -0.002 | -0.54498 | 145 |  |  |
| -0.54398 | 223.153 | -0.00195 | -0.54395 | 146 |  |  |
| -0.54291 | 224.2211 | -0.0019 | -0.54279 | 147 |  |  |
| -0.54184 | 225.2893 | -0.00185 | -0.54184 | 148 |  |  |
| -0.54077 | 226.3574 | -0.0018 | -0.54062 | 149 |  |  |
| -0.5397 | 227.4255 | -0.00175 | -0.53967 | 150 |  |  |
| -0.53864 | 228.4936 | -0.00171 | -0.53854 | 151 |  |  |
| -0.53757 | 229.5617 | -0.00166 | -0.53751 | 152 |  |  |
| -0.5365 | 230.6298 | -0.00162 | -0.53641 | 153 |  |  |
| -0.53543 | 231.6979 | -0.00158 | -0.53537 | 154 |  |  |
| -0.53436 | 232.7661 | -0.00154 | -0.53442 | 155 |  |  |
| -0.53329 | 233.8342 | -0.0015 | -0.53336 | 156 |  |  |
| -0.53223 | 234.9023 | -0.00146 | -0.53223 | 157 |  |  |
| -0.53116 | 235.9704 | -0.00142 | -0.53113 | 158 |  |  |
| -0.53009 | 237.0385 | -0.00138 | -0.53006 | 159 |  |  |
| -0.52902 | 238.1066 | -0.00134 | -0.52905 | 160 |  |  |
| -0.52795 | 239.1748 | -0.00131 | -0.52792 | 161 |  |  |
| -0.52689 | 240.2429 | -0.00127 | -0.52682 | 162 |  |  |
| -0.52582 | 241.311 | -0.00124 | -0.52594 | 163 |  |  |
| -0.52475 | 242.3791 | -0.00121 | -0.52481 | 164 |  |  |
| -0.52368 | 243.4472 | -0.00117 | -0.52374 | 165 |  |  |
| -0.52261 | 244.5153 | -0.00114 | -0.52267 | 166 |  |  |
| -0.52155 | 245.5834 | -0.00111 | -0.52161 | 167 |  |  |
| -0.52048 | 246.6516 | -0.00108 | -0.52054 | 168 |  |  |
| -0.51941 | 247.7197 | -0.00105 | -0.51938 | 169 |  |  |
| -0.51834 | 248.7878 | -0.00103 | -0.51846 | 170 |  |  |
| -0.51727 | 249.8559 | -0.001 | -0.51752 | 171 |  |  |
| -0.5162 | 250.924 | -0.00097 | -0.51627 | 172 |  |  |
| -0.51514 | 251.9921 | -0.00094 | -0.51526 | 173 |  |  |
| -0.51407 | 253.0602 | -0.00092 | -0.51419 | 174 |  |  |
| -0.513 | 254.1284 | -0.00089 | -0.51306 | 175 |  |  |
| -0.51193 | 255.1965 | -0.00087 | -0.51193 | 176 |  |  |
| -0.51086 | 256.2646 | -0.00085 | -0.51096 | 177 |  |  |
| -0.5098 | 257.3327 | -0.00082 | -0.50986 | 178 |  |  |
| -0.50873 | 258.4008 | -0.0008 | -0.50885 | 179 |  |  |
| -0.50766 | 259.4689 | -0.00078 | -0.50766 | 180 |  |  |
| -0.50659 | 260.5371 | -0.00076 | -0.50668 | 181 |  |  |
| -0.50552 | 261.6052 | -0.00074 | -0.50552 | 182 |  |  |
| -0.50446 | 262.6733 | -0.00072 | -0.50455 | 183 |  |  |
| -0.50339 | 263.7414 | -0.0007 | -0.50351 | 184 |  |  |
| -0.50232 | 264.8095 | -0.00068 | -0.50235 | 185 |  |  |
| -0.50125 | 265.8776 | -0.00066 | -0.50143 | 186 |  |  |
| -0.50018 | 266.9457 | -0.00064 | -0.50034 | 187 |  |  |
| -0.49911 | 268.0139 | -0.00062 | -0.49924 | 188 |  |  |
| -0.49805 | 269.082 | -0.0006 | -0.49817 | 189 |  |  |
| -0.49698 | 270.1501 | -0.00059 | -0.49719 | 190 |  |  |
| -0.49591 | 271.2182 | -0.00057 | -0.49612 | 191 |  |  |
| -0.49484 | 272.2863 | -0.00055 | -0.49509 | 192 |  |  |
| -0.49377 | 273.3544 | -0.00054 | -0.49393 | 193 |  |  |
| -0.49271 | 274.4225 | -0.00052 | -0.49286 | 194 |  |  |
| -0.49164 | 275.4907 | -0.00051 | -0.49185 | 195 |  |  |
| -0.49057 | 276.5588 | -0.00049 | -0.49072 | 196 |  |  |
| -0.4895 | 277.6269 | -0.00048 | -0.48969 | 197 |  |  |
| -0.48843 | 278.695 | -0.00046 | -0.48856 | 198 |  |  |
| -0.48737 | 279.7631 | -0.00045 | -0.48761 | 199 |  |  |
| -0.4863 | 280.8312 | -0.00043 | -0.48642 | 200 |  |  |
| -0.48523 | 281.8994 | -0.00042 | -0.48535 | 201 |  |  |
| -0.48416 | 282.9675 | -0.00041 | -0.48422 | 202 |  |  |
| -0.48309 | 284.0356 | -0.00039 | -0.48325 | 203 |  |  |
| -0.48203 | 285.1037 | -0.00038 | -0.48209 | 204 |  |  |
| -0.48096 | 286.1718 | -0.00037 | -0.48108 | 205 |  |  |
| -0.47989 | 287.2399 | -0.00036 | -0.47995 | 206 |  |  |
| -0.47882 | 288.308 | -0.00035 | -0.47903 | 207 |  |  |
| -0.47775 | 289.3762 | -0.00033 | -0.47787 | 208 |  |  |
| -0.47668 | 290.4443 | -0.00032 | -0.47696 | 209 |  |  |
| -0.47562 | 291.5124 | -0.00031 | -0.47577 | 210 |  |  |
| -0.47455 | 292.5805 | -0.0003 | -0.47479 | 211 |  |  |
| -0.47348 | 293.6486 | -0.00029 | -0.47357 | 212 |  |  |
| -0.47241 | 294.7167 | -0.00028 | -0.47263 | 213 |  |  |
| -0.47134 | 295.7848 | -0.00027 | -0.4715 | 214 |  |  |
| -0.47028 | 296.853 | -0.00026 | -0.47052 | 215 |  |  |
| -0.46921 | 297.9211 | -0.00025 | -0.46942 | 216 |  |  |
| -0.46814 | 298.9892 | -0.00024 | -0.46823 | 217 |  |  |
| -0.46707 | 300.0573 | -0.00023 | -0.46719 | 218 |  |  |
| -0.466 | 301.1254 | -0.00022 | -0.46619 | 219 |  |  |
| -0.46494 | 302.1935 | -0.00021 | -0.46503 | 220 |  |  |
| -0.46387 | 303.2617 | -0.0002 | -0.46408 | 221 |  |  |
| -0.4628 | 304.3298 | -0.00019 | -0.46292 | 222 |  |  |
| -0.46173 | 305.3979 | -0.00019 | -0.46182 | 223 |  |  |
| -0.46066 | 306.466 | -0.00018 | -0.46072 | 224 |  |  |
| -0.45959 | 307.5341 | -0.00017 | -0.45981 | 225 |  |  |
| -0.45853 | 308.6022 | -0.00016 | -0.45871 | 226 |  |  |
| -0.45746 | 309.6703 | -0.00015 | -0.45767 | 227 |  |  |
| -0.45639 | 310.7385 | -0.00014 | -0.45651 | 228 |  |  |
| -0.45532 | 311.8066 | -0.00013 | -0.45551 | 229 |  |  |
| -0.45425 | 312.8747 | -0.00013 | -0.45441 | 230 |  |  |
| -0.45319 | 313.9428 | -0.00012 | -0.45337 | 231 |  |  |
| -0.45212 | 315.0109 | -0.00011 | -0.45224 | 232 |  |  |
| -0.45105 | 316.079 | -0.0001 | -0.45123 | 233 |  |  |
| -0.44998 | 317.1471 | -9.3E-05 | -0.4501 | 234 |  |  |
| -0.44891 | 318.2153 | -8.5E-05 | -0.44916 | 235 |  |  |
| -0.44785 | 319.2834 | -7.7E-05 | -0.44797 | 236 |  |  |
| -0.44678 | 320.3515 | -6.9E-05 | -0.44699 | 237 |  |  |
| -0.44571 | 321.4196 | -6.1E-05 | -0.44592 | 238 |  |  |
| -0.44464 | 322.4877 | -5.4E-05 | -0.44482 | 239 |  |  |
| -0.44357 | 323.5558 | -4.6E-05 | -0.4437 | 240 |  |  |
| -0.4425 | 324.624 | -3.7E-05 | -0.4425 | 241 |  |  |
| -0.44144 | 325.6921 | -2.9E-05 | -0.44156 | 242 |  |  |
| -0.44037 | 326.7602 | -2.1E-05 | -0.44049 | 243 |  |  |
| -0.4393 | 327.8283 | -1.4E-05 | -0.43942 | 244 |  |  |
| -0.43823 | 328.8964 | -5.6E-06 | -0.43845 | 245 |  |  |
| -0.43716 | 329.9645 | 2.5E-06 | -0.43738 | 246 |  |  |
| -0.4361 | 331.0326 | 1.06E-05 | -0.43628 | 247 |  |  |
| -0.43503 | 332.1008 | 1.88E-05 | -0.43518 | 248 |  |  |
| -0.43396 | 333.1689 | 2.69E-05 | -0.43408 | 249 |  |  |
| -0.43289 | 334.237 | 3.49E-05 | -0.43307 | 250 |  |  |
| -0.43182 | 335.3051 | 4.39E-05 | -0.43204 | 251 |  |  |
| -0.43076 | 336.3732 | 5.27E-05 | -0.43106 | 252 |  |  |
| -0.42969 | 337.4413 | 6.14E-05 | -0.42999 | 253 |  |  |
| -0.42862 | 338.5094 | 7.03E-05 | -0.42905 | 254 |  |  |
| -0.42755 | 339.5776 | 7.94E-05 | -0.42789 | 255 |  |  |
| -0.42648 | 340.6457 | 8.84E-05 | -0.42676 | 256 |  |  |
| -0.42542 | 341.7138 | 9.77E-05 | -0.4256 | 257 |  |  |
| -0.42435 | 342.7819 | 0.000107 | -0.4248 | 258 |  |  |
| -0.42328 | 343.85 | 0.000117 | -0.42368 | 259 |  |  |
| -0.42221 | 344.9181 | 0.000127 | -0.42264 | 260 |  |  |
| -0.42114 | 345.9863 | 0.000137 | -0.42145 | 261 |  |  |
| -0.42007 | 347.0544 | 0.000147 | -0.42059 | 262 |  |  |
| -0.41901 | 348.1225 | 0.000158 | -0.4194 | 263 |  |  |
| -0.41794 | 349.1906 | 0.000168 | -0.4184 | 264 |  |  |
| -0.41687 | 350.2587 | 0.00018 | -0.41727 | 265 |  |  |
| -0.4158 | 351.3268 | 0.000191 | -0.41644 | 266 |  |  |
| -0.41473 | 352.3949 | 0.000202 | -0.41522 | 267 |  |  |
| -0.41367 | 353.4631 | 0.000214 | -0.41422 | 268 |  |  |
| -0.4126 | 354.5312 | 0.000226 | -0.41309 | 269 |  |  |
| -0.41153 | 355.5993 | 0.000239 | -0.41205 | 270 |  |  |
| -0.41046 | 356.6674 | 0.000252 | -0.41104 | 271 |  |  |
| -0.40939 | 357.7355 | 0.000265 | -0.40997 | 272 |  |  |
| -0.40833 | 358.8036 | 0.000279 | -0.40891 | 273 |  |  |
| -0.40726 | 359.8717 | 0.000293 | -0.40768 | 274 |  |  |
| -0.40619 | 360.9399 | 0.000308 | -0.40671 | 275 |  |  |
| -0.40512 | 362.008 | 0.000323 | -0.40536 | 276 |  |  |
| -0.40405 | 363.0761 | 0.000339 | -0.40424 | 277 |  |  |
| -0.40298 | 364.1442 | 0.000355 | -0.40311 | 278 |  |  |
| -0.40192 | 365.2123 | 0.000372 | -0.40213 | 279 |  |  |
| -0.40085 | 366.2804 | 0.000389 | -0.40106 | 280 |  |  |
| -0.39978 | 367.3486 | 0.000407 | -0.39993 | 281 |  |  |
| -0.39871 | 368.4167 | 0.000426 | -0.39877 | 282 |  |  |
| -0.39764 | 369.4848 | 0.000445 | -0.39786 | 283 |  |  |
| -0.39658 | 370.5529 | 0.000465 | -0.39679 | 284 |  |  |
| -0.39551 | 371.621 | 0.000486 | -0.39581 | 285 |  |  |
| -0.39444 | 372.6891 | 0.000508 | -0.39465 | 286 |  |  |
| -0.39337 | 373.7572 | 0.000531 | -0.39368 | 287 |  |  |
| -0.3923 | 374.8254 | 0.000554 | -0.39258 | 288 |  |  |
| -0.39124 | 375.8935 | 0.000579 | -0.39157 | 289 |  |  |
| -0.39017 | 376.9616 | 0.000605 | -0.39056 | 290 |  |  |
| -0.3891 | 378.0297 | 0.000631 | -0.38937 | 291 |  |  |
| -0.38803 | 379.0978 | 0.000659 | -0.38837 | 292 |  |  |
| -0.38696 | 380.1659 | 0.000688 | -0.3873 | 293 |  |  |
| -0.38589 | 381.234 | 0.000717 | -0.3862 | 294 |  |  |
| -0.38483 | 382.3022 | 0.000749 | -0.38519 | 295 |  |  |
| -0.38376 | 383.3703 | 0.000781 | -0.38403 | 296 |  |  |
| -0.38269 | 384.4384 | 0.000815 | -0.383 | 297 |  |  |
| -0.38162 | 385.5065 | 0.00085 | -0.38202 | 298 |  |  |
| -0.38055 | 386.5746 | 0.000887 | -0.38089 | 299 |  |  |
| -0.37949 | 387.6427 | 0.000926 | -0.37976 | 300 |  |  |
| -0.37842 | 388.7109 | 0.000966 | -0.37869 | 301 |  |  |
| -0.37735 | 389.779 | 0.001009 | -0.37772 | 302 |  |  |
| -0.37628 | 390.8471 | 0.001053 | -0.37665 | 303 |  |  |
| -0.37521 | 391.9152 | 0.0011 | -0.3754 | 304 |  |  |
| -0.37415 | 392.9833 | 0.001148 | -0.37451 | 305 |  |  |
| -0.37308 | 394.0514 | 0.001199 | -0.37347 | 306 |  |  |
| -0.37201 | 395.1195 | 0.001252 | -0.37231 | 307 |  |  |
| -0.37094 | 396.1877 | 0.001307 | -0.37125 | 308 |  |  |
| -0.36987 | 397.2558 | 0.001364 | -0.37036 | 309 |  |  |
| -0.3688 | 398.3239 | 0.001425 | -0.36911 | 310 |  |  |
| -0.36774 | 399.392 | 0.001489 | -0.36813 | 311 |  |  |
| -0.36667 | 400.4601 | 0.001556 | -0.36703 | 312 |  |  |
| -0.3656 | 401.5282 | 0.001626 | -0.366 | 313 |  |  |
| -0.36453 | 402.5963 | 0.0017 | -0.36493 | 314 |  |  |
| -0.36346 | 403.6645 | 0.001778 | -0.36377 | 315 |  |  |
| -0.3624 | 404.7326 | 0.001859 | -0.36273 | 316 |  |  |
| -0.36133 | 405.8007 | 0.001944 | -0.36157 | 317 |  |  |
| -0.36026 | 406.8688 | 0.002034 | -0.36069 | 318 |  |  |
| -0.35919 | 407.9369 | 0.002127 | -0.35968 | 319 |  |  |
| -0.35812 | 409.005 | 0.002225 | -0.35858 | 320 |  |  |
| -0.35706 | 410.0732 | 0.002328 | -0.35751 | 321 |  |  |
| -0.35599 | 411.1413 | 0.002437 | -0.35651 | 322 |  |  |
| -0.35492 | 412.2094 | 0.002551 | -0.35544 | 323 |  |  |
| -0.35385 | 413.2775 | 0.002672 | -0.35437 | 324 |  |  |
| -0.35278 | 414.3456 | 0.002798 | -0.35336 | 325 |  |  |
| -0.35172 | 415.4137 | 0.002932 | -0.35239 | 326 |  |  |
| -0.35065 | 416.4818 | 0.003072 | -0.35117 | 327 |  |  |
| -0.34958 | 417.55 | 0.00322 | -0.35022 | 328 |  |  |
| -0.34851 | 418.6181 | 0.003374 | -0.34921 | 329 |  |  |
| -0.34744 | 419.6862 | 0.003535 | -0.34814 | 330 |  |  |
| -0.34637 | 420.7543 | 0.003701 | -0.34708 | 331 |  |  |
| -0.34531 | 421.8224 | 0.00387 | -0.34601 | 332 |  |  |
| -0.34424 | 422.8905 | 0.004037 | -0.34506 | 333 |  |  |
| -0.34317 | 423.9586 | 0.004196 | -0.34399 | 334 |  |  |
| -0.3421 | 425.0268 | 0.004345 | -0.34302 | 335 |  |  |
| -0.34103 | 426.0949 | 0.004481 | -0.34204 | 336 |  |  |
| -0.33997 | 427.163 | 0.004604 | -0.34088 | 337 |  |  |
| -0.3389 | 428.2311 | 0.004715 | -0.33987 | 338 |  |  |
| -0.33783 | 429.2992 | 0.004816 | -0.3389 | 339 |  |  |
| -0.33676 | 430.3673 | 0.004908 | -0.33768 | 340 |  |  |
| -0.33569 | 431.4355 | 0.004993 | -0.3367 | 341 |  |  |
| -0.33463 | 432.5036 | 0.005072 | -0.33569 | 342 |  |  |
| -0.33356 | 433.5717 | 0.005146 | -0.3345 | 343 |  |  |
| -0.33249 | 434.6398 | 0.005216 | -0.33356 | 344 |  |  |
| -0.33142 | 435.7079 | 0.005283 | -0.33246 | 345 |  |  |
| -0.33035 | 436.776 | 0.005346 | -0.33145 | 346 |  |  |
| -0.32928 | 437.8441 | 0.005408 | -0.33029 | 347 |  |  |
| -0.32822 | 438.9123 | 0.005468 | -0.32922 | 348 |  |  |
| -0.32715 | 439.9804 | 0.005525 | -0.32828 | 349 |  |  |
| -0.32608 | 441.0485 | 0.005581 | -0.32727 | 350 |  |  |
| -0.32501 | 442.1166 | 0.005636 | -0.32617 | 351 |  |  |
| -0.32394 | 443.1847 | 0.00569 | -0.32526 | 352 |  |  |
| -0.32288 | 444.2528 | 0.005742 | -0.32413 | 353 |  |  |
| -0.32181 | 445.3209 | 0.005794 | -0.32303 | 354 |  |  |
| -0.32074 | 446.3891 | 0.005844 | -0.32187 | 355 |  |  |
| -0.31967 | 447.4572 | 0.005894 | -0.32086 | 356 |  |  |
| -0.3186 | 448.5253 | 0.005944 | -0.31985 | 357 |  |  |
| -0.31754 | 449.5934 | 0.005993 | -0.31882 | 358 |  |  |
| -0.31647 | 450.6615 | 0.006041 | -0.31766 | 359 |  |  |
| -0.3154 | 451.7296 | 0.006089 | -0.31668 | 360 |  |  |
| -0.31433 | 452.7978 | 0.006136 | -0.3157 | 361 |  |  |
| -0.31326 | 453.8659 | 0.006183 | -0.31454 | 362 |  |  |
| -0.31219 | 454.934 | 0.006229 | -0.31348 | 363 |  |  |
| -0.31113 | 456.0021 | 0.006275 | -0.31247 | 364 |  |  |
| -0.31006 | 457.0702 | 0.00632 | -0.31146 | 365 |  |  |
| -0.30899 | 458.1383 | 0.006364 | -0.31033 | 366 |  |  |
| -0.30792 | 459.2064 | 0.006407 | -0.3092 | 367 |  |  |
| -0.30685 | 460.2746 | 0.006451 | -0.30826 | 368 |  |  |
| -0.30579 | 461.3427 | 0.006493 | -0.30719 | 369 |  |  |
| -0.30472 | 462.4108 | 0.006534 | -0.306 | 370 |  |  |
| -0.30365 | 463.4789 | 0.006575 | -0.30496 | 371 |  |  |
| -0.30258 | 464.547 | 0.006614 | -0.30396 | 372 |  |  |
| -0.30151 | 465.6151 | 0.006653 | -0.30298 | 373 |  |  |
| -0.30045 | 466.6832 | 0.00669 | -0.30188 | 374 |  |  |
| -0.29938 | 467.7514 | 0.006727 | -0.30084 | 375 |  |  |
| -0.29831 | 468.8195 | 0.006762 | -0.29971 | 376 |  |  |
| -0.29724 | 469.8876 | 0.006797 | -0.29868 | 377 |  |  |
| -0.29617 | 470.9557 | 0.00683 | -0.29761 | 378 |  |  |
| -0.2951 | 472.0238 | 0.006863 | -0.2966 | 379 |  |  |
| -0.29404 | 473.0919 | 0.006895 | -0.29556 | 380 |  |  |
| -0.29297 | 474.1601 | 0.006926 | -0.29443 | 381 |  |  |
| -0.2919 | 475.2282 | 0.006955 | -0.29343 | 382 |  |  |
| -0.29083 | 476.2963 | 0.006985 | -0.29224 | 383 |  |  |
| -0.28976 | 477.3644 | 0.007013 | -0.29126 | 384 |  |  |
| -0.2887 | 478.4325 | 0.00704 | -0.29025 | 385 |  |  |
| -0.28763 | 479.5006 | 0.007066 | -0.28915 | 386 |  |  |
| -0.28656 | 480.5687 | 0.007092 | -0.28809 | 387 |  |  |
| -0.28549 | 481.6369 | 0.007118 | -0.28702 | 388 |  |  |
| -0.28442 | 482.705 | 0.007142 | -0.28598 | 389 |  |  |
| -0.28336 | 483.7731 | 0.007166 | -0.28488 | 390 |  |  |
| -0.28229 | 484.8412 | 0.007189 | -0.28384 | 391 |  |  |
| -0.28122 | 485.9093 | 0.007212 | -0.28278 | 392 |  |  |
| -0.28015 | 486.9774 | 0.007234 | -0.28171 | 393 |  |  |
| -0.27908 | 488.0455 | 0.007255 | -0.28061 | 394 |  |  |
| -0.27802 | 489.1137 | 0.007275 | -0.27954 | 395 |  |  |
| -0.27695 | 490.1818 | 0.007296 | -0.27847 | 396 |  |  |
| -0.27588 | 491.2499 | 0.007316 | -0.27744 | 397 |  |  |
| -0.27481 | 492.318 | 0.007336 | -0.27637 | 398 |  |  |
| -0.27374 | 493.3861 | 0.007354 | -0.27527 | 399 |  |  |
| -0.27267 | 494.4542 | 0.007372 | -0.27423 | 400 |  |  |
| -0.27161 | 495.5224 | 0.00739 | -0.27319 | 401 |  |  |
| -0.27054 | 496.5905 | 0.007408 | -0.27213 | 402 |  |  |
| -0.26947 | 497.6586 | 0.007425 | -0.27106 | 403 |  |  |
| -0.2684 | 498.7267 | 0.007442 | -0.26996 | 404 |  |  |
| -0.26733 | 499.7948 | 0.007458 | -0.26895 | 405 |  |  |
| -0.26627 | 500.8629 | 0.007474 | -0.26776 | 406 |  |  |
| -0.2652 | 501.931 | 0.007489 | -0.26682 | 407 |  |  |
| -0.26413 | 502.9992 | 0.007504 | -0.26575 | 408 |  |  |
| -0.26306 | 504.0673 | 0.007519 | -0.2648 | 409 |  |  |
| -0.26199 | 505.1354 | 0.007533 | -0.26364 | 410 |  |  |
| -0.26093 | 506.2035 | 0.007548 | -0.26257 | 411 |  |  |
| -0.25986 | 507.2716 | 0.007562 | -0.26144 | 412 |  |  |
| -0.25879 | 508.3397 | 0.007576 | -0.26041 | 413 |  |  |
| -0.25772 | 509.4078 | 0.007589 | -0.25937 | 414 |  |  |
| -0.25665 | 510.476 | 0.007603 | -0.25824 | 415 |  |  |
| -0.25558 | 511.5441 | 0.007616 | -0.25723 | 416 |  |  |
| -0.25452 | 512.6122 | 0.007628 | -0.25613 | 417 |  |  |
| -0.25345 | 513.6803 | 0.007641 | -0.25516 | 418 |  |  |
| -0.25238 | 514.7484 | 0.007653 | -0.25409 | 419 |  |  |
| -0.25131 | 515.8165 | 0.007666 | -0.25293 | 420 |  |  |
| -0.25024 | 516.8847 | 0.007678 | -0.25183 | 421 |  |  |
| -0.24918 | 517.9528 | 0.00769 | -0.25092 | 422 |  |  |
| -0.24811 | 519.0209 | 0.007702 | -0.24979 | 423 |  |  |
| -0.24704 | 520.089 | 0.007714 | -0.24869 | 424 |  |  |
| -0.24597 | 521.1571 | 0.007725 | -0.24774 | 425 |  |  |
| -0.2449 | 522.2252 | 0.007736 | -0.24649 | 426 |  |  |
| -0.24384 | 523.2933 | 0.007747 | -0.24554 | 427 |  |  |
| -0.24277 | 524.3615 | 0.007759 | -0.24448 | 428 |  |  |
| -0.2417 | 525.4296 | 0.00777 | -0.24341 | 429 |  |  |
| -0.24063 | 526.4977 | 0.007781 | -0.24246 | 430 |  |  |
| -0.23956 | 527.5658 | 0.007792 | -0.2413 | 431 |  |  |
| -0.23849 | 528.6339 | 0.007802 | -0.24023 | 432 |  |  |
| -0.23743 | 529.702 | 0.007813 | -0.23911 | 433 |  |  |
| -0.23636 | 530.7701 | 0.007823 | -0.23807 | 434 |  |  |
| -0.23529 | 531.8383 | 0.007833 | -0.23703 | 435 |  |  |
| -0.23422 | 532.9064 | 0.007843 | -0.23587 | 436 |  |  |
| -0.23315 | 533.9745 | 0.007854 | -0.23492 | 437 |  |  |
| -0.23209 | 535.0426 | 0.007864 | -0.23383 | 438 |  |  |
| -0.23102 | 536.1107 | 0.007874 | -0.23282 | 439 |  |  |
| -0.22995 | 537.1788 | 0.007884 | -0.23169 | 440 |  |  |
| -0.22888 | 538.247 | 0.007893 | -0.23065 | 441 |  |  |
| -0.22781 | 539.3151 | 0.007902 | -0.22955 | 442 |  |  |
| -0.22675 | 540.3832 | 0.007911 | -0.22852 | 443 |  |  |
| -0.22568 | 541.4513 | 0.007921 | -0.22742 | 444 |  |  |
| -0.22461 | 542.5194 | 0.007931 | -0.22638 | 445 |  |  |
| -0.22354 | 543.5875 | 0.007939 | -0.22528 | 446 |  |  |
| -0.22247 | 544.6556 | 0.007949 | -0.22406 | 447 |  |  |
| -0.22141 | 545.7238 | 0.007958 | -0.22321 | 448 |  |  |
| -0.22034 | 546.7919 | 0.007966 | -0.22223 | 449 |  |  |
| -0.21927 | 547.86 | 0.007975 | -0.22104 | 450 |  |  |
| -0.2182 | 548.9281 | 0.007983 | -0.21985 | 451 |  |  |
| -0.21713 | 549.9962 | 0.007991 | -0.21881 | 452 |  |  |
| -0.21606 | 551.0643 | 0.007998 | -0.21786 | 453 |  |  |
| -0.215 | 552.1324 | 0.008008 | -0.21671 | 454 |  |  |
| -0.21393 | 553.2006 | 0.008016 | -0.2157 | 455 |  |  |
| -0.21286 | 554.2687 | 0.008024 | -0.2146 | 456 |  |  |
| -0.21179 | 555.3368 | 0.008031 | -0.21347 | 457 |  |  |
| -0.21072 | 556.4049 | 0.00804 | -0.21246 | 458 |  |  |
| -0.20966 | 557.473 | 0.008047 | -0.21149 | 459 |  |  |
| -0.20859 | 558.5411 | 0.008055 | -0.21042 | 460 |  |  |
| -0.20752 | 559.6093 | 0.008063 | -0.20935 | 461 |  |  |
| -0.20645 | 560.6774 | 0.008071 | -0.20825 | 462 |  |  |
| -0.20538 | 561.7455 | 0.008078 | -0.20724 | 463 |  |  |
| -0.20432 | 562.8136 | 0.008085 | -0.20618 | 464 |  |  |
| -0.20325 | 563.8817 | 0.008093 | -0.20496 | 465 |  |  |
| -0.20218 | 564.9498 | 0.0081 | -0.20392 | 466 |  |  |
| -0.20111 | 566.0179 | 0.008108 | -0.20291 | 467 |  |  |
| -0.20004 | 567.0861 | 0.008115 | -0.20197 | 468 |  |  |

Sample: *Wormwood*, Concentration (ppm): 600, Immersion time: 24h

| Potential applied(V) | Time (s) | WE(1).  Current (A) | WE(1).  Potential (V) | Index |  |  |
| --- | --- | --- | --- | --- | --- | --- |
| -0.69305 | 68.77236 | -9.3E-05 | -0.69107 | 1 |  |  |
| -0.69199 | 69.84048 | -0.00093 | -0.68991 | 2 |  |  |
| -0.69092 | 70.90859 | -0.00715 | -0.68939 | 3 |  |  |
| -0.68985 | 71.97671 | -0.00712 | -0.68835 | 4 |  |  |
| -0.68878 | 73.04482 | -0.00709 | -0.68723 | 5 |  |  |
| -0.68771 | 74.11294 | -0.00706 | -0.68628 | 6 |  |  |
| -0.68665 | 75.18105 | -0.00704 | -0.68518 | 7 |  |  |
| -0.68558 | 76.24917 | -0.00701 | -0.68411 | 8 |  |  |
| -0.68451 | 77.31728 | -0.00699 | -0.68311 | 9 |  |  |
| -0.68344 | 78.3854 | -0.00697 | -0.68204 | 10 |  |  |
| -0.68237 | 79.45351 | -0.00694 | -0.68088 | 11 |  |  |
| -0.6813 | 80.52163 | -0.00692 | -0.67987 | 12 |  |  |
| -0.68024 | 81.58974 | -0.0069 | -0.6788 | 13 |  |  |
| -0.67917 | 82.65786 | -0.00688 | -0.67773 | 14 |  |  |
| -0.6781 | 83.72597 | -0.00686 | -0.67667 | 15 |  |  |
| -0.67703 | 84.79409 | -0.00683 | -0.67563 | 16 |  |  |
| -0.67596 | 85.8622 | -0.00681 | -0.67456 | 17 |  |  |
| -0.6749 | 86.93032 | -0.00679 | -0.67352 | 18 |  |  |
| -0.67383 | 87.99843 | -0.00677 | -0.67252 | 19 |  |  |
| -0.67276 | 89.06655 | -0.00675 | -0.67139 | 20 |  |  |
| -0.67169 | 90.13466 | -0.00673 | -0.67035 | 21 |  |  |
| -0.67062 | 91.20278 | -0.00671 | -0.66931 | 22 |  |  |
| -0.66956 | 92.27089 | -0.00669 | -0.66827 | 23 |  |  |
| -0.66849 | 93.33901 | -0.00667 | -0.66718 | 24 |  |  |
| -0.66742 | 94.40712 | -0.00665 | -0.66611 | 25 |  |  |
| -0.66635 | 95.47524 | -0.00663 | -0.66507 | 26 |  |  |
| -0.66528 | 96.54335 | -0.00661 | -0.66391 | 27 |  |  |
| -0.66422 | 97.61147 | -0.00659 | -0.6629 | 28 |  |  |
| -0.66315 | 98.67958 | -0.00657 | -0.6619 | 29 |  |  |
| -0.66208 | 99.7477 | -0.00655 | -0.66074 | 30 |  |  |
| -0.66101 | 100.8158 | -0.00653 | -0.65973 | 31 |  |  |
| -0.65994 | 101.8839 | -0.00651 | -0.65857 | 32 |  |  |
| -0.65887 | 102.952 | -0.00649 | -0.65759 | 33 |  |  |
| -0.65781 | 104.0202 | -0.00647 | -0.65662 | 34 |  |  |
| -0.65674 | 105.0883 | -0.00645 | -0.6554 | 35 |  |  |
| -0.65567 | 106.1564 | -0.00643 | -0.65445 | 36 |  |  |
| -0.6546 | 107.2245 | -0.00641 | -0.65329 | 37 |  |  |
| -0.65353 | 108.2926 | -0.00639 | -0.65228 | 38 |  |  |
| -0.65247 | 109.3607 | -0.00636 | -0.65121 | 39 |  |  |
| -0.6514 | 110.4288 | -0.00634 | -0.65015 | 40 |  |  |
| -0.65033 | 111.497 | -0.00632 | -0.64914 | 41 |  |  |
| -0.64926 | 112.5651 | -0.0063 | -0.64804 | 42 |  |  |
| -0.64819 | 113.6332 | -0.00628 | -0.64697 | 43 |  |  |
| -0.64713 | 114.7013 | -0.00626 | -0.64594 | 44 |  |  |
| -0.64606 | 115.7694 | -0.00624 | -0.64484 | 45 |  |  |
| -0.64499 | 116.8375 | -0.00622 | -0.64374 | 46 |  |  |
| -0.64392 | 117.9057 | -0.0062 | -0.64273 | 47 |  |  |
| -0.64285 | 118.9738 | -0.00618 | -0.64166 | 48 |  |  |
| -0.64178 | 120.0419 | -0.00616 | -0.64063 | 49 |  |  |
| -0.64072 | 121.11 | -0.00614 | -0.63956 | 50 |  |  |
| -0.63965 | 122.1781 | -0.00612 | -0.63852 | 51 |  |  |
| -0.63858 | 123.2462 | -0.0061 | -0.63748 | 52 |  |  |
| -0.63751 | 124.3143 | -0.00607 | -0.63629 | 53 |  |  |
| -0.63644 | 125.3825 | -0.00605 | -0.63531 | 54 |  |  |
| -0.63538 | 126.4506 | -0.00603 | -0.63422 | 55 |  |  |
| -0.63431 | 127.5187 | -0.00601 | -0.63321 | 56 |  |  |
| -0.63324 | 128.5868 | -0.00599 | -0.63214 | 57 |  |  |
| -0.63217 | 129.6549 | -0.00597 | -0.63104 | 58 |  |  |
| -0.6311 | 130.723 | -0.00594 | -0.63 | 59 |  |  |
| -0.63004 | 131.7911 | -0.00592 | -0.62888 | 60 |  |  |
| -0.62897 | 132.8593 | -0.0059 | -0.6279 | 61 |  |  |
| -0.6279 | 133.9274 | -0.00588 | -0.62683 | 62 |  |  |
| -0.62683 | 134.9955 | -0.00585 | -0.6257 | 63 |  |  |
| -0.62576 | 136.0636 | -0.00583 | -0.62469 | 64 |  |  |
| -0.62469 | 137.1317 | -0.00581 | -0.62369 | 65 |  |  |
| -0.62363 | 138.1998 | -0.00578 | -0.62256 | 66 |  |  |
| -0.62256 | 139.268 | -0.00576 | -0.62152 | 67 |  |  |
| -0.62149 | 140.3361 | -0.00574 | -0.62048 | 68 |  |  |
| -0.62042 | 141.4042 | -0.00571 | -0.61938 | 69 |  |  |
| -0.61935 | 142.4723 | -0.00569 | -0.61832 | 70 |  |  |
| -0.61829 | 143.5404 | -0.00566 | -0.61725 | 71 |  |  |
| -0.61722 | 144.6085 | -0.00564 | -0.61615 | 72 |  |  |
| -0.61615 | 145.6766 | -0.00561 | -0.61502 | 73 |  |  |
| -0.61508 | 146.7448 | -0.00559 | -0.61401 | 74 |  |  |
| -0.61401 | 147.8129 | -0.00556 | -0.61298 | 75 |  |  |
| -0.61295 | 148.881 | -0.00553 | -0.61191 | 76 |  |  |
| -0.61188 | 149.9491 | -0.00551 | -0.61096 | 77 |  |  |
| -0.61081 | 151.0172 | -0.00548 | -0.60971 | 78 |  |  |
| -0.60974 | 152.0853 | -0.00545 | -0.60873 | 79 |  |  |
| -0.60867 | 153.1534 | -0.00542 | -0.6077 | 80 |  |  |
| -0.6076 | 154.2216 | -0.00539 | -0.60666 | 81 |  |  |
| -0.60654 | 155.2897 | -0.00536 | -0.6055 | 82 |  |  |
| -0.60547 | 156.3578 | -0.00533 | -0.60449 | 83 |  |  |
| -0.6044 | 157.4259 | -0.0053 | -0.60342 | 84 |  |  |
| -0.60333 | 158.494 | -0.00527 | -0.60239 | 85 |  |  |
| -0.60226 | 159.5621 | -0.00524 | -0.60129 | 86 |  |  |
| -0.6012 | 160.6303 | -0.0052 | -0.60022 | 87 |  |  |
| -0.60013 | 161.6984 | -0.00517 | -0.59921 | 88 |  |  |
| -0.59906 | 162.7665 | -0.00513 | -0.59811 | 89 |  |  |
| -0.59799 | 163.8346 | -0.0051 | -0.59708 | 90 |  |  |
| -0.59692 | 164.9027 | -0.00506 | -0.59598 | 91 |  |  |
| -0.59586 | 165.9708 | -0.00502 | -0.59497 | 92 |  |  |
| -0.59479 | 167.0389 | -0.00498 | -0.5939 | 93 |  |  |
| -0.59372 | 168.1071 | -0.00494 | -0.5928 | 94 |  |  |
| -0.59265 | 169.1752 | -0.00489 | -0.59174 | 95 |  |  |
| -0.59158 | 170.2433 | -0.00484 | -0.59067 | 96 |  |  |
| -0.59052 | 171.3114 | -0.0048 | -0.58963 | 97 |  |  |
| -0.58945 | 172.3795 | -0.00474 | -0.58865 | 98 |  |  |
| -0.58838 | 173.4476 | -0.00469 | -0.58752 | 99 |  |  |
| -0.58731 | 174.5157 | -0.00463 | -0.58649 | 100 |  |  |
| -0.58624 | 175.5839 | -0.00457 | -0.58548 | 101 |  |  |
| -0.58517 | 176.652 | -0.00451 | -0.58438 | 102 |  |  |
| -0.58411 | 177.7201 | -0.00444 | -0.58334 | 103 |  |  |
| -0.58304 | 178.7882 | -0.00437 | -0.58237 | 104 |  |  |
| -0.58197 | 179.8563 | -0.00429 | -0.58124 | 105 |  |  |
| -0.5809 | 180.9244 | -0.00421 | -0.58014 | 106 |  |  |
| -0.57983 | 181.9926 | -0.00413 | -0.57916 | 107 |  |  |
| -0.57877 | 183.0607 | -0.00404 | -0.57797 | 108 |  |  |
| -0.5777 | 184.1288 | -0.00395 | -0.57712 | 109 |  |  |
| -0.57663 | 185.1969 | -0.00386 | -0.57605 | 110 |  |  |
| -0.57556 | 186.265 | -0.00376 | -0.57492 | 111 |  |  |
| -0.57449 | 187.3331 | -0.00367 | -0.57391 | 112 |  |  |
| -0.57343 | 188.4012 | -0.00358 | -0.57288 | 113 |  |  |
| -0.57236 | 189.4694 | -0.00349 | -0.57187 | 114 |  |  |
| -0.57129 | 190.5375 | -0.00339 | -0.57086 | 115 |  |  |
| -0.57022 | 191.6056 | -0.00331 | -0.5697 | 116 |  |  |
| -0.56915 | 192.6737 | -0.00322 | -0.56866 | 117 |  |  |
| -0.56808 | 193.7418 | -0.00313 | -0.56766 | 118 |  |  |
| -0.56702 | 194.8099 | -0.00305 | -0.56662 | 119 |  |  |
| -0.56595 | 195.878 | -0.00297 | -0.56555 | 120 |  |  |
| -0.56488 | 196.9462 | -0.00289 | -0.56448 | 121 |  |  |
| -0.56381 | 198.0143 | -0.00281 | -0.56351 | 122 |  |  |
| -0.56274 | 199.0824 | -0.00274 | -0.56244 | 123 |  |  |
| -0.56168 | 200.1505 | -0.00266 | -0.56134 | 124 |  |  |
| -0.56061 | 201.2186 | -0.00259 | -0.5603 | 125 |  |  |
| -0.55954 | 202.2867 | -0.00252 | -0.55923 | 126 |  |  |
| -0.55847 | 203.3549 | -0.00245 | -0.5582 | 127 |  |  |
| -0.5574 | 204.423 | -0.00239 | -0.55719 | 128 |  |  |
| -0.55634 | 205.4911 | -0.00232 | -0.55612 | 129 |  |  |
| -0.55527 | 206.5592 | -0.00226 | -0.55502 | 130 |  |  |
| -0.5542 | 207.6273 | -0.0022 | -0.55402 | 131 |  |  |
| -0.55313 | 208.6954 | -0.00214 | -0.55298 | 132 |  |  |
| -0.55206 | 209.7635 | -0.00208 | -0.55188 | 133 |  |  |
| -0.55099 | 210.8317 | -0.00203 | -0.55081 | 134 |  |  |
| -0.54993 | 211.8998 | -0.00197 | -0.54977 | 135 |  |  |
| -0.54886 | 212.9679 | -0.00192 | -0.54874 | 136 |  |  |
| -0.54779 | 214.036 | -0.00187 | -0.5477 | 137 |  |  |
| -0.54672 | 215.1041 | -0.00182 | -0.5466 | 138 |  |  |
| -0.54565 | 216.1722 | -0.00177 | -0.5455 | 139 |  |  |
| -0.54459 | 217.2403 | -0.00172 | -0.54446 | 140 |  |  |
| -0.54352 | 218.3085 | -0.00167 | -0.54343 | 141 |  |  |
| -0.54245 | 219.3766 | -0.00163 | -0.54236 | 142 |  |  |
| -0.54138 | 220.4447 | -0.00158 | -0.54135 | 143 |  |  |
| -0.54031 | 221.5128 | -0.00154 | -0.54022 | 144 |  |  |
| -0.53925 | 222.5809 | -0.0015 | -0.53915 | 145 |  |  |
| -0.53818 | 223.649 | -0.00146 | -0.53812 | 146 |  |  |
| -0.53711 | 224.7172 | -0.00142 | -0.53705 | 147 |  |  |
| -0.53604 | 225.7853 | -0.00138 | -0.53598 | 148 |  |  |
| -0.53497 | 226.8534 | -0.00134 | -0.53497 | 149 |  |  |
| -0.53391 | 227.9215 | -0.0013 | -0.53387 | 150 |  |  |
| -0.53284 | 228.9896 | -0.00127 | -0.53275 | 151 |  |  |
| -0.53177 | 230.0577 | -0.00123 | -0.53174 | 152 |  |  |
| -0.5307 | 231.1258 | -0.0012 | -0.53073 | 153 |  |  |
| -0.52963 | 232.194 | -0.00117 | -0.52969 | 154 |  |  |
| -0.52856 | 233.2621 | -0.00113 | -0.52856 | 155 |  |  |
| -0.5275 | 234.3302 | -0.0011 | -0.52753 | 156 |  |  |
| -0.52643 | 235.3983 | -0.00107 | -0.52649 | 157 |  |  |
| -0.52536 | 236.4664 | -0.00104 | -0.52545 | 158 |  |  |
| -0.52429 | 237.5345 | -0.00102 | -0.52426 | 159 |  |  |
| -0.52322 | 238.6026 | -0.00099 | -0.52328 | 160 |  |  |
| -0.52216 | 239.6708 | -0.00096 | -0.52222 | 161 |  |  |
| -0.52109 | 240.7389 | -0.00093 | -0.52112 | 162 |  |  |
| -0.52002 | 241.807 | -0.00091 | -0.52005 | 163 |  |  |
| -0.51895 | 242.8751 | -0.00088 | -0.51895 | 164 |  |  |
| -0.51788 | 243.9432 | -0.00086 | -0.51794 | 165 |  |  |
| -0.51682 | 245.0113 | -0.00083 | -0.51688 | 166 |  |  |
| -0.51575 | 246.0795 | -0.00081 | -0.51578 | 167 |  |  |
| -0.51468 | 247.1476 | -0.00079 | -0.51465 | 168 |  |  |
| -0.51361 | 248.2157 | -0.00077 | -0.51364 | 169 |  |  |
| -0.51254 | 249.2838 | -0.00075 | -0.51263 | 170 |  |  |
| -0.51147 | 250.3519 | -0.00072 | -0.51151 | 171 |  |  |
| -0.51041 | 251.42 | -0.0007 | -0.5105 | 172 |  |  |
| -0.50934 | 252.4881 | -0.00068 | -0.50946 | 173 |  |  |
| -0.50827 | 253.5563 | -0.00067 | -0.5083 | 174 |  |  |
| -0.5072 | 254.6244 | -0.00065 | -0.50723 | 175 |  |  |
| -0.50613 | 255.6925 | -0.00063 | -0.50629 | 176 |  |  |
| -0.50507 | 256.7606 | -0.00061 | -0.50519 | 177 |  |  |
| -0.504 | 257.8287 | -0.00059 | -0.50403 | 178 |  |  |
| -0.50293 | 258.8968 | -0.00058 | -0.50302 | 179 |  |  |
| -0.50186 | 259.9649 | -0.00056 | -0.50192 | 180 |  |  |
| -0.50079 | 261.0331 | -0.00054 | -0.50089 | 181 |  |  |
| -0.49973 | 262.1012 | -0.00053 | -0.49979 | 182 |  |  |
| -0.49866 | 263.1693 | -0.00051 | -0.49878 | 183 |  |  |
| -0.49759 | 264.2374 | -0.0005 | -0.49777 | 184 |  |  |
| -0.49652 | 265.3055 | -0.00048 | -0.49664 | 185 |  |  |
| -0.49545 | 266.3736 | -0.00047 | -0.49551 | 186 |  |  |
| -0.49438 | 267.4418 | -0.00046 | -0.49451 | 187 |  |  |
| -0.49332 | 268.5099 | -0.00044 | -0.49341 | 188 |  |  |
| -0.49225 | 269.578 | -0.00043 | -0.49237 | 189 |  |  |
| -0.49118 | 270.6461 | -0.00042 | -0.49124 | 190 |  |  |
| -0.49011 | 271.7142 | -0.0004 | -0.49023 | 191 |  |  |
| -0.48904 | 272.7823 | -0.00039 | -0.48917 | 192 |  |  |
| -0.48798 | 273.8504 | -0.00038 | -0.48804 | 193 |  |  |
| -0.48691 | 274.9186 | -0.00037 | -0.48703 | 194 |  |  |
| -0.48584 | 275.9867 | -0.00036 | -0.48596 | 195 |  |  |
| -0.48477 | 277.0548 | -0.00035 | -0.48489 | 196 |  |  |
| -0.4837 | 278.1229 | -0.00033 | -0.48386 | 197 |  |  |
| -0.48264 | 279.191 | -0.00032 | -0.48276 | 198 |  |  |
| -0.48157 | 280.2591 | -0.00031 | -0.48166 | 199 |  |  |
| -0.4805 | 281.3272 | -0.0003 | -0.48056 | 200 |  |  |
| -0.47943 | 282.3954 | -0.00029 | -0.47952 | 201 |  |  |
| -0.47836 | 283.4635 | -0.00028 | -0.47852 | 202 |  |  |
| -0.47729 | 284.5316 | -0.00027 | -0.47742 | 203 |  |  |
| -0.47623 | 285.5997 | -0.00027 | -0.47638 | 204 |  |  |
| -0.47516 | 286.6678 | -0.00026 | -0.47525 | 205 |  |  |
| -0.47409 | 287.7359 | -0.00025 | -0.47415 | 206 |  |  |
| -0.47302 | 288.8041 | -0.00024 | -0.47311 | 207 |  |  |
| -0.47195 | 289.8722 | -0.00023 | -0.47208 | 208 |  |  |
| -0.47089 | 290.9403 | -0.00022 | -0.47098 | 209 |  |  |
| -0.46982 | 292.0084 | -0.00021 | -0.46991 | 210 |  |  |
| -0.46875 | 293.0765 | -0.00021 | -0.46881 | 211 |  |  |
| -0.46768 | 294.1446 | -0.0002 | -0.4678 | 212 |  |  |
| -0.46661 | 295.2127 | -0.00019 | -0.46674 | 213 |  |  |
| -0.46555 | 296.2809 | -0.00018 | -0.46567 | 214 |  |  |
| -0.46448 | 297.349 | -0.00017 | -0.46454 | 215 |  |  |
| -0.46341 | 298.4171 | -0.00017 | -0.4635 | 216 |  |  |
| -0.46234 | 299.4852 | -0.00016 | -0.46249 | 217 |  |  |
| -0.46127 | 300.5533 | -0.00015 | -0.46133 | 218 |  |  |
| -0.46021 | 301.6214 | -0.00015 | -0.46033 | 219 |  |  |
| -0.45914 | 302.6895 | -0.00014 | -0.45923 | 220 |  |  |
| -0.45807 | 303.7577 | -0.00013 | -0.45822 | 221 |  |  |
| -0.457 | 304.8258 | -0.00013 | -0.45712 | 222 |  |  |
| -0.45593 | 305.8939 | -0.00012 | -0.45602 | 223 |  |  |
| -0.45486 | 306.962 | -0.00011 | -0.45499 | 224 |  |  |
| -0.4538 | 308.0301 | -0.00011 | -0.45398 | 225 |  |  |
| -0.45273 | 309.0982 | -9.9E-05 | -0.45285 | 226 |  |  |
| -0.45166 | 310.1664 | -9.3E-05 | -0.45172 | 227 |  |  |
| -0.45059 | 311.2345 | -8.7E-05 | -0.45078 | 228 |  |  |
| -0.44952 | 312.3026 | -8E-05 | -0.44962 | 229 |  |  |
| -0.44846 | 313.3707 | -7.4E-05 | -0.44855 | 230 |  |  |
| -0.44739 | 314.4388 | -6.8E-05 | -0.44742 | 231 |  |  |
| -0.44632 | 315.5069 | -6.2E-05 | -0.44644 | 232 |  |  |
| -0.44525 | 316.575 | -5.6E-05 | -0.44534 | 233 |  |  |
| -0.44418 | 317.6432 | -5E-05 | -0.44418 | 234 |  |  |
| -0.44312 | 318.7113 | -4.4E-05 | -0.44318 | 235 |  |  |
| -0.44205 | 319.7794 | -3.8E-05 | -0.44217 | 236 |  |  |
| -0.44098 | 320.8475 | -3.1E-05 | -0.44104 | 237 |  |  |
| -0.43991 | 321.9156 | -2.7E-05 | -0.44 | 238 |  |  |
| -0.43884 | 322.9837 | -2.1E-05 | -0.43896 | 239 |  |  |
| -0.43777 | 324.0518 | -1.5E-05 | -0.4379 | 240 |  |  |
| -0.43671 | 325.12 | -9.3E-06 | -0.4368 | 241 |  |  |
| -0.43564 | 326.1881 | -3.5E-06 | -0.43585 | 242 |  |  |
| -0.43457 | 327.2562 | 2.27E-06 | -0.43475 | 243 |  |  |
| -0.4335 | 328.3243 | 8.21E-06 | -0.43365 | 244 |  |  |
| -0.43243 | 329.3924 | 1.41E-05 | -0.43253 | 245 |  |  |
| -0.43137 | 330.4605 | 1.99E-05 | -0.43149 | 246 |  |  |
| -0.4303 | 331.5287 | 2.51E-05 | -0.43033 | 247 |  |  |
| -0.42923 | 332.5968 | 3.15E-05 | -0.42941 | 248 |  |  |
| -0.42816 | 333.6649 | 3.74E-05 | -0.42834 | 249 |  |  |
| -0.42709 | 334.733 | 4.42E-05 | -0.42725 | 250 |  |  |
| -0.42603 | 335.8011 | 5.04E-05 | -0.42618 | 251 |  |  |
| -0.42496 | 336.8692 | 5.66E-05 | -0.42517 | 252 |  |  |
| -0.42389 | 337.9373 | 6.28E-05 | -0.42416 | 253 |  |  |
| -0.42282 | 339.0055 | 6.94E-05 | -0.42307 | 254 |  |  |
| -0.42175 | 340.0736 | 7.58E-05 | -0.422 | 255 |  |  |
| -0.42068 | 341.1417 | 8.23E-05 | -0.42099 | 256 |  |  |
| -0.41962 | 342.2098 | 8.87E-05 | -0.41989 | 257 |  |  |
| -0.41855 | 343.2779 | 9.56E-05 | -0.41879 | 258 |  |  |
| -0.41748 | 344.346 | 0.000103 | -0.41785 | 259 |  |  |
| -0.41641 | 345.4141 | 0.00011 | -0.41675 | 260 |  |  |
| -0.41534 | 346.4823 | 0.000117 | -0.41571 | 261 |  |  |
| -0.41428 | 347.5504 | 0.000124 | -0.41464 | 262 |  |  |
| -0.41321 | 348.6185 | 0.000132 | -0.41354 | 263 |  |  |
| -0.41214 | 349.6866 | 0.000139 | -0.41251 | 264 |  |  |
| -0.41107 | 350.7547 | 0.000147 | -0.41147 | 265 |  |  |
| -0.41 | 351.8228 | 0.000155 | -0.4104 | 266 |  |  |
| -0.40894 | 352.891 | 0.000163 | -0.40939 | 267 |  |  |
| -0.40787 | 353.9591 | 0.000172 | -0.4082 | 268 |  |  |
| -0.4068 | 355.0272 | 0.00018 | -0.40717 | 269 |  |  |
| -0.40573 | 356.0953 | 0.000189 | -0.40616 | 270 |  |  |
| -0.40466 | 357.1634 | 0.000198 | -0.40503 | 271 |  |  |
| -0.40359 | 358.2315 | 0.000207 | -0.40405 | 272 |  |  |
| -0.40253 | 359.2996 | 0.000217 | -0.40302 | 273 |  |  |
| -0.40146 | 360.3678 | 0.000227 | -0.40179 | 274 |  |  |
| -0.40039 | 361.4359 | 0.000237 | -0.40082 | 275 |  |  |
| -0.39932 | 362.504 | 0.000248 | -0.39978 | 276 |  |  |
| -0.39825 | 363.5721 | 0.000259 | -0.39874 | 277 |  |  |
| -0.39719 | 364.6402 | 0.00027 | -0.39777 | 278 |  |  |
| -0.39612 | 365.7083 | 0.000282 | -0.39664 | 279 |  |  |
| -0.39505 | 366.7764 | 0.000294 | -0.39554 | 280 |  |  |
| -0.39398 | 367.8446 | 0.000307 | -0.39459 | 281 |  |  |
| -0.39291 | 368.9127 | 0.00032 | -0.3931 | 282 |  |  |
| -0.39185 | 369.9808 | 0.000333 | -0.392 | 283 |  |  |
| -0.39078 | 371.0489 | 0.000347 | -0.39096 | 284 |  |  |
| -0.38971 | 372.117 | 0.000362 | -0.38983 | 285 |  |  |
| -0.38864 | 373.1851 | 0.000377 | -0.38879 | 286 |  |  |
| -0.38757 | 374.2533 | 0.000393 | -0.3877 | 287 |  |  |
| -0.38651 | 375.3214 | 0.000409 | -0.38663 | 288 |  |  |
| -0.38544 | 376.3895 | 0.000427 | -0.38565 | 289 |  |  |
| -0.38437 | 377.4576 | 0.000444 | -0.38458 | 290 |  |  |
| -0.3833 | 378.5257 | 0.000463 | -0.38345 | 291 |  |  |
| -0.38223 | 379.5938 | 0.000482 | -0.38242 | 292 |  |  |
| -0.38116 | 380.6619 | 0.000502 | -0.38129 | 293 |  |  |
| -0.3801 | 381.7301 | 0.000523 | -0.38028 | 294 |  |  |
| -0.37903 | 382.7982 | 0.000545 | -0.37924 | 295 |  |  |
| -0.37796 | 383.8663 | 0.000567 | -0.37823 | 296 |  |  |
| -0.37689 | 384.9344 | 0.000591 | -0.37717 | 297 |  |  |
| -0.37582 | 386.0025 | 0.000616 | -0.37598 | 298 |  |  |
| -0.37476 | 387.0706 | 0.000642 | -0.375 | 299 |  |  |
| -0.37369 | 388.1387 | 0.000669 | -0.37396 | 300 |  |  |
| -0.37262 | 389.2069 | 0.000698 | -0.37283 | 301 |  |  |
| -0.37155 | 390.275 | 0.000727 | -0.37177 | 302 |  |  |
| -0.37048 | 391.3431 | 0.000758 | -0.3707 | 303 |  |  |
| -0.36942 | 392.4112 | 0.000791 | -0.36969 | 304 |  |  |
| -0.36835 | 393.4793 | 0.000826 | -0.36853 | 305 |  |  |
| -0.36728 | 394.5474 | 0.000862 | -0.36755 | 306 |  |  |
| -0.36621 | 395.6156 | 0.0009 | -0.36649 | 307 |  |  |
| -0.36514 | 396.6837 | 0.000941 | -0.36548 | 308 |  |  |
| -0.36407 | 397.7518 | 0.000982 | -0.36432 | 309 |  |  |
| -0.36301 | 398.8199 | 0.001027 | -0.36328 | 310 |  |  |
| -0.36194 | 399.888 | 0.001074 | -0.36224 | 311 |  |  |
| -0.36087 | 400.9561 | 0.001123 | -0.36111 | 312 |  |  |
| -0.3598 | 402.0242 | 0.001174 | -0.36014 | 313 |  |  |
| -0.35873 | 403.0924 | 0.001229 | -0.35904 | 314 |  |  |
| -0.35767 | 404.1605 | 0.001286 | -0.35788 | 315 |  |  |
| -0.3566 | 405.2286 | 0.001346 | -0.35693 | 316 |  |  |
| -0.35553 | 406.2967 | 0.00141 | -0.3559 | 317 |  |  |
| -0.35446 | 407.3648 | 0.001477 | -0.35477 | 318 |  |  |
| -0.35339 | 408.4329 | 0.001547 | -0.35373 | 319 |  |  |
| -0.35233 | 409.501 | 0.001622 | -0.35257 | 320 |  |  |
| -0.35126 | 410.5692 | 0.001701 | -0.3515 | 321 |  |  |
| -0.35019 | 411.6373 | 0.001784 | -0.35062 | 322 |  |  |
| -0.34912 | 412.7054 | 0.001873 | -0.34943 | 323 |  |  |
| -0.34805 | 413.7735 | 0.001966 | -0.34845 | 324 |  |  |
| -0.34698 | 414.8416 | 0.002065 | -0.34735 | 325 |  |  |
| -0.34592 | 415.9097 | 0.00217 | -0.34631 | 326 |  |  |
| -0.34485 | 416.9779 | 0.002281 | -0.34531 | 327 |  |  |
| -0.34378 | 418.046 | 0.002397 | -0.34412 | 328 |  |  |
| -0.34271 | 419.1141 | 0.002521 | -0.34311 | 329 |  |  |
| -0.34164 | 420.1822 | 0.00265 | -0.34219 | 330 |  |  |
| -0.34058 | 421.2503 | 0.002791 | -0.34109 | 331 |  |  |
| -0.33951 | 422.3184 | 0.002938 | -0.34006 | 332 |  |  |
| -0.33844 | 423.3865 | 0.003093 | -0.33896 | 333 |  |  |
| -0.33737 | 424.4547 | 0.003257 | -0.33795 | 334 |  |  |
| -0.3363 | 425.5228 | 0.00343 | -0.33698 | 335 |  |  |
| -0.33524 | 426.5909 | 0.00361 | -0.33594 | 336 |  |  |
| -0.33417 | 427.659 | 0.003796 | -0.33487 | 337 |  |  |
| -0.3331 | 428.7271 | 0.003978 | -0.3338 | 338 |  |  |
| -0.33203 | 429.7952 | 0.004155 | -0.33282 | 339 |  |  |
| -0.33096 | 430.8633 | 0.00432 | -0.33182 | 340 |  |  |
| -0.3299 | 431.9315 | 0.004469 | -0.33066 | 341 |  |  |
| -0.32883 | 432.9996 | 0.004602 | -0.32971 | 342 |  |  |
| -0.32776 | 434.0677 | 0.004722 | -0.32864 | 343 |  |  |
| -0.32669 | 435.1358 | 0.00483 | -0.32758 | 344 |  |  |
| -0.32562 | 436.2039 | 0.004928 | -0.32666 | 345 |  |  |
| -0.32455 | 437.272 | 0.005018 | -0.32553 | 346 |  |  |
| -0.32349 | 438.3402 | 0.005103 | -0.32452 | 347 |  |  |
| -0.32242 | 439.4083 | 0.005183 | -0.32346 | 348 |  |  |
| -0.32135 | 440.4764 | 0.005258 | -0.32236 | 349 |  |  |
| -0.32028 | 441.5445 | 0.00533 | -0.32138 | 350 |  |  |
| -0.31921 | 442.6126 | 0.005399 | -0.32028 | 351 |  |  |
| -0.31815 | 443.6807 | 0.005466 | -0.31915 | 352 |  |  |
| -0.31708 | 444.7488 | 0.005531 | -0.31815 | 353 |  |  |
| -0.31601 | 445.817 | 0.005594 | -0.31714 | 354 |  |  |
| -0.31494 | 446.8851 | 0.005655 | -0.31604 | 355 |  |  |
| -0.31387 | 447.9532 | 0.005716 | -0.31506 | 356 |  |  |
| -0.31281 | 449.0213 | 0.005776 | -0.31396 | 357 |  |  |
| -0.31174 | 450.0894 | 0.005835 | -0.31287 | 358 |  |  |
| -0.31067 | 451.1575 | 0.005893 | -0.31186 | 359 |  |  |
| -0.3096 | 452.2256 | 0.005951 | -0.31076 | 360 |  |  |
| -0.30853 | 453.2938 | 0.006007 | -0.30969 | 361 |  |  |
| -0.30746 | 454.3619 | 0.006064 | -0.30869 | 362 |  |  |
| -0.3064 | 455.43 | 0.006119 | -0.30765 | 363 |  |  |
| -0.30533 | 456.4981 | 0.006175 | -0.30652 | 364 |  |  |
| -0.30426 | 457.5662 | 0.00623 | -0.30551 | 365 |  |  |
| -0.30319 | 458.6343 | 0.006285 | -0.30447 | 366 |  |  |
| -0.30212 | 459.7025 | 0.006339 | -0.30341 | 367 |  |  |
| -0.30106 | 460.7706 | 0.006393 | -0.30234 | 368 |  |  |
| -0.29999 | 461.8387 | 0.006445 | -0.3013 | 369 |  |  |
| -0.29892 | 462.9068 | 0.006497 | -0.30026 | 370 |  |  |
| -0.29785 | 463.9749 | 0.006549 | -0.29922 | 371 |  |  |
| -0.29678 | 465.043 | 0.0066 | -0.29807 | 372 |  |  |
| -0.29572 | 466.1111 | 0.00665 | -0.29703 | 373 |  |  |
| -0.29465 | 467.1793 | 0.006699 | -0.29599 | 374 |  |  |
| -0.29358 | 468.2474 | 0.006747 | -0.29498 | 375 |  |  |
| -0.29251 | 469.3155 | 0.006793 | -0.29391 | 376 |  |  |
| -0.29144 | 470.3836 | 0.006838 | -0.29282 | 377 |  |  |
| -0.29037 | 471.4517 | 0.006882 | -0.29187 | 378 |  |  |
| -0.28931 | 472.5198 | 0.006924 | -0.29077 | 379 |  |  |
| -0.28824 | 473.5879 | 0.006965 | -0.28964 | 380 |  |  |
| -0.28717 | 474.6561 | 0.007005 | -0.2886 | 381 |  |  |
| -0.2861 | 475.7242 | 0.007043 | -0.2876 | 382 |  |  |
| -0.28503 | 476.7923 | 0.00708 | -0.28653 | 383 |  |  |
| -0.28397 | 477.8604 | 0.007116 | -0.28543 | 384 |  |  |
| -0.2829 | 478.9285 | 0.00715 | -0.28442 | 385 |  |  |
| -0.28183 | 479.9966 | 0.007183 | -0.28333 | 386 |  |  |
| -0.28076 | 481.0648 | 0.007215 | -0.28223 | 387 |  |  |
| -0.27969 | 482.1329 | 0.007246 | -0.28122 | 388 |  |  |
| -0.27863 | 483.201 | 0.007276 | -0.28018 | 389 |  |  |
| -0.27756 | 484.2691 | 0.007306 | -0.27908 | 390 |  |  |
| -0.27649 | 485.3372 | 0.007333 | -0.27805 | 391 |  |  |
| -0.27542 | 486.4053 | 0.00736 | -0.27704 | 392 |  |  |
| -0.27435 | 487.4734 | 0.007386 | -0.27588 | 393 |  |  |
| -0.27328 | 488.5416 | 0.007411 | -0.2749 | 394 |  |  |
| -0.27222 | 489.6097 | 0.007436 | -0.27371 | 395 |  |  |
| -0.27115 | 490.6778 | 0.00746 | -0.2728 | 396 |  |  |
| -0.27008 | 491.7459 | 0.007483 | -0.27164 | 397 |  |  |
| -0.26901 | 492.814 | 0.007505 | -0.27057 | 398 |  |  |
| -0.26794 | 493.8821 | 0.007527 | -0.2695 | 399 |  |  |
| -0.26688 | 494.9502 | 0.007548 | -0.26852 | 400 |  |  |
| -0.26581 | 496.0184 | 0.007569 | -0.26733 | 401 |  |  |
| -0.26474 | 497.0865 | 0.007588 | -0.26633 | 402 |  |  |
| -0.26367 | 498.1546 | 0.007608 | -0.26529 | 403 |  |  |
| -0.2626 | 499.2227 | 0.007627 | -0.26422 | 404 |  |  |
| -0.26154 | 500.2908 | 0.007645 | -0.26318 | 405 |  |  |
| -0.26047 | 501.3589 | 0.007663 | -0.26208 | 406 |  |  |
| -0.2594 | 502.4271 | 0.007681 | -0.26105 | 407 |  |  |
| -0.25833 | 503.4952 | 0.007699 | -0.25995 | 408 |  |  |
| -0.25726 | 504.5633 | 0.007716 | -0.25891 | 409 |  |  |
| -0.2562 | 505.6314 | 0.007733 | -0.25787 | 410 |  |  |
| -0.25513 | 506.6995 | 0.007748 | -0.25677 | 411 |  |  |
| -0.25406 | 507.7676 | 0.007765 | -0.25571 | 412 |  |  |
| -0.25299 | 508.8357 | 0.00778 | -0.25467 | 413 |  |  |
| -0.25192 | 509.9039 | 0.007796 | -0.25357 | 414 |  |  |
| -0.25085 | 510.972 | 0.007811 | -0.25253 | 415 |  |  |
| -0.24979 | 512.0401 | 0.007825 | -0.25143 | 416 |  |  |
| -0.24872 | 513.1082 | 0.007839 | -0.2504 | 417 |  |  |
| -0.24765 | 514.1763 | 0.007853 | -0.24939 | 418 |  |  |
| -0.24658 | 515.2444 | 0.007867 | -0.24826 | 419 |  |  |
| -0.24551 | 516.3125 | 0.00788 | -0.24719 | 420 |  |  |
| -0.24445 | 517.3807 | 0.007892 | -0.24615 | 421 |  |  |
| -0.24338 | 518.4488 | 0.007905 | -0.24509 | 422 |  |  |
| -0.24231 | 519.5169 | 0.007917 | -0.24402 | 423 |  |  |
| -0.24124 | 520.585 | 0.007931 | -0.24301 | 424 |  |  |
| -0.24017 | 521.6531 | 0.007943 | -0.24191 | 425 |  |  |
| -0.23911 | 522.7212 | 0.007956 | -0.24088 | 426 |  |  |
| -0.23804 | 523.7894 | 0.00797 | -0.23972 | 427 |  |  |
| -0.23697 | 524.8575 | 0.007982 | -0.23868 | 428 |  |  |
| -0.2359 | 525.9256 | 0.007997 | -0.23758 | 429 |  |  |
| -0.23483 | 526.9937 | 0.008008 | -0.23654 | 430 |  |  |
| -0.23376 | 528.0618 | 0.008021 | -0.23553 | 431 |  |  |
| -0.2327 | 529.1299 | 0.008032 | -0.23438 | 432 |  |  |
| -0.23163 | 530.198 | 0.008044 | -0.23331 | 433 |  |  |
| -0.23056 | 531.2662 | 0.008058 | -0.2323 | 434 |  |  |
| -0.22949 | 532.3343 | 0.008068 | -0.2312 | 435 |  |  |
| -0.22842 | 533.4024 | 0.008079 | -0.23026 | 436 |  |  |
| -0.22736 | 534.4705 | 0.00809 | -0.22922 | 437 |  |  |
| -0.22629 | 535.5386 | 0.008099 | -0.22809 | 438 |  |  |
| -0.22522 | 536.6067 | 0.008111 | -0.22702 | 439 |  |  |
| -0.22415 | 537.6748 | 0.008121 | -0.22595 | 440 |  |  |
| -0.22308 | 538.743 | 0.008131 | -0.22485 | 441 |  |  |
| -0.22202 | 539.8111 | 0.008141 | -0.22382 | 442 |  |  |
| -0.22095 | 540.8792 | 0.008152 | -0.22278 | 443 |  |  |
| -0.21988 | 541.9473 | 0.008163 | -0.22159 | 444 |  |  |
| -0.21881 | 543.0154 | 0.008174 | -0.22061 | 445 |  |  |
| -0.21774 | 544.0835 | 0.008183 | -0.21951 | 446 |  |  |
| -0.21667 | 545.1517 | 0.008194 | -0.21854 | 447 |  |  |
| -0.21561 | 546.2198 | 0.008206 | -0.21738 | 448 |  |  |
| -0.21454 | 547.2879 | 0.008214 | -0.21637 | 449 |  |  |
| -0.21347 | 548.356 | 0.008224 | -0.21527 | 450 |  |  |
| -0.2124 | 549.4241 | 0.008233 | -0.21423 | 451 |  |  |
| -0.21133 | 550.4922 | 0.008242 | -0.2131 | 452 |  |  |
| -0.21027 | 551.5603 | 0.008253 | -0.21216 | 453 |  |  |
| -0.2092 | 552.6285 | 0.008261 | -0.21097 | 454 |  |  |
| -0.20813 | 553.6966 | 0.008269 | -0.20993 | 455 |  |  |
| -0.20706 | 554.7647 | 0.008277 | -0.20889 | 456 |  |  |
| -0.20599 | 555.8328 | 0.008286 | -0.20776 | 457 |  |  |
| -0.20493 | 556.9009 | 0.008294 | -0.20673 | 458 |  |  |
| -0.20386 | 557.969 | 0.008301 | -0.20566 | 459 |  |  |
| -0.20279 | 559.0371 | 0.00831 | -0.20462 | 460 |  |  |
| -0.20172 | 560.1053 | 0.008321 | -0.20349 | 461 |  |  |
| -0.20065 | 561.1734 | 0.008329 | -0.20245 | 462 |  |  |
| -0.19958 | 562.2415 | 0.008337 | -0.20139 | 463 |  |  |
| -0.19852 | 563.3096 | 0.008347 | -0.20035 | 464 |  |  |
| -0.19745 | 564.3777 | 0.008357 | -0.19934 | 465 |  |  |
| -0.19638 | 565.4458 | 0.008364 | -0.19821 | 466 |  |  |
| -0.19531 | 566.514 | 0.008373 | -0.19705 | 467 |  |  |
| -0.19424 | 567.5821 | 0.008382 | -0.19611 | 468 |  |  |

Sample: *Wormwood*, Concentration (ppm): 800, Immersion time: 24h

| Potential applied(V) | Time (s) | WE(1).  Current (A) | WE(1).  Potential (V) | Index |  |  |
| --- | --- | --- | --- | --- | --- | --- |
| -0.69778 | 68.64937 | -9.3E-05 | -0.69577 | 1 |  |  |
| -0.69672 | 69.71748 | -0.00093 | -0.69467 | 2 |  |  |
| -0.69565 | 70.7856 | -0.00717 | -0.69424 | 3 |  |  |
| -0.69458 | 71.85371 | -0.00713 | -0.69305 | 4 |  |  |
| -0.69351 | 72.92183 | -0.0071 | -0.69199 | 5 |  |  |
| -0.69244 | 73.98994 | -0.00707 | -0.69101 | 6 |  |  |
| -0.69138 | 75.05806 | -0.00705 | -0.68982 | 7 |  |  |
| -0.69031 | 76.12617 | -0.00702 | -0.68872 | 8 |  |  |
| -0.68924 | 77.19429 | -0.007 | -0.68774 | 9 |  |  |
| -0.68817 | 78.2624 | -0.00697 | -0.68668 | 10 |  |  |
| -0.6871 | 79.33052 | -0.00695 | -0.68576 | 11 |  |  |
| -0.68604 | 80.39863 | -0.00693 | -0.68466 | 12 |  |  |
| -0.68497 | 81.46675 | -0.00691 | -0.68353 | 13 |  |  |
| -0.6839 | 82.53486 | -0.00689 | -0.68246 | 14 |  |  |
| -0.68283 | 83.60298 | -0.00686 | -0.6813 | 15 |  |  |
| -0.68176 | 84.67109 | -0.00684 | -0.68036 | 16 |  |  |
| -0.68069 | 85.73921 | -0.00682 | -0.67914 | 17 |  |  |
| -0.67963 | 86.80732 | -0.0068 | -0.67813 | 18 |  |  |
| -0.67856 | 87.87544 | -0.00678 | -0.67712 | 19 |  |  |
| -0.67749 | 88.94355 | -0.00676 | -0.67612 | 20 |  |  |
| -0.67642 | 90.01167 | -0.00674 | -0.67511 | 21 |  |  |
| -0.67535 | 91.07978 | -0.00672 | -0.67392 | 22 |  |  |
| -0.67429 | 92.1479 | -0.0067 | -0.67288 | 23 |  |  |
| -0.67322 | 93.21601 | -0.00668 | -0.67184 | 24 |  |  |
| -0.67215 | 94.28413 | -0.00666 | -0.67075 | 25 |  |  |
| -0.67108 | 95.35224 | -0.00664 | -0.66968 | 26 |  |  |
| -0.67001 | 96.42036 | -0.00662 | -0.66864 | 27 |  |  |
| -0.66895 | 97.48847 | -0.0066 | -0.66763 | 28 |  |  |
| -0.66788 | 98.55659 | -0.00658 | -0.6665 | 29 |  |  |
| -0.66681 | 99.6247 | -0.00656 | -0.66553 | 30 |  |  |
| -0.66574 | 100.6928 | -0.00654 | -0.66437 | 31 |  |  |
| -0.66467 | 101.7609 | -0.00652 | -0.66339 | 32 |  |  |
| -0.6636 | 102.829 | -0.0065 | -0.66229 | 33 |  |  |
| -0.66254 | 103.8972 | -0.00648 | -0.66119 | 34 |  |  |
| -0.66147 | 104.9653 | -0.00646 | -0.66019 | 35 |  |  |
| -0.6604 | 106.0334 | -0.00644 | -0.65915 | 36 |  |  |
| -0.65933 | 107.1015 | -0.00642 | -0.65808 | 37 |  |  |
| -0.65826 | 108.1696 | -0.0064 | -0.65692 | 38 |  |  |
| -0.6572 | 109.2377 | -0.00638 | -0.65604 | 39 |  |  |
| -0.65613 | 110.3059 | -0.00636 | -0.65479 | 40 |  |  |
| -0.65506 | 111.374 | -0.00634 | -0.65384 | 41 |  |  |
| -0.65399 | 112.4421 | -0.00632 | -0.6528 | 42 |  |  |
| -0.65292 | 113.5102 | -0.0063 | -0.65167 | 43 |  |  |
| -0.65186 | 114.5783 | -0.00628 | -0.65054 | 44 |  |  |
| -0.65079 | 115.6464 | -0.00626 | -0.64954 | 45 |  |  |
| -0.64972 | 116.7145 | -0.00624 | -0.64841 | 46 |  |  |
| -0.64865 | 117.7827 | -0.00622 | -0.64746 | 47 |  |  |
| -0.64758 | 118.8508 | -0.0062 | -0.64639 | 48 |  |  |
| -0.64651 | 119.9189 | -0.00618 | -0.64529 | 49 |  |  |
| -0.64545 | 120.987 | -0.00616 | -0.64423 | 50 |  |  |
| -0.64438 | 122.0551 | -0.00614 | -0.64319 | 51 |  |  |
| -0.64331 | 123.1232 | -0.00612 | -0.64218 | 52 |  |  |
| -0.64224 | 124.1913 | -0.0061 | -0.64099 | 53 |  |  |
| -0.64117 | 125.2595 | -0.00608 | -0.63998 | 54 |  |  |
| -0.64011 | 126.3276 | -0.00606 | -0.63892 | 55 |  |  |
| -0.63904 | 127.3957 | -0.00604 | -0.63788 | 56 |  |  |
| -0.63797 | 128.4638 | -0.00602 | -0.63672 | 57 |  |  |
| -0.6369 | 129.5319 | -0.006 | -0.63571 | 58 |  |  |
| -0.63583 | 130.6 | -0.00598 | -0.63474 | 59 |  |  |
| -0.63477 | 131.6682 | -0.00596 | -0.63364 | 60 |  |  |
| -0.6337 | 132.7363 | -0.00594 | -0.63257 | 61 |  |  |
| -0.63263 | 133.8044 | -0.00591 | -0.63147 | 62 |  |  |
| -0.63156 | 134.8725 | -0.00589 | -0.63049 | 63 |  |  |
| -0.63049 | 135.9406 | -0.00587 | -0.62927 | 64 |  |  |
| -0.62943 | 137.0087 | -0.00585 | -0.62833 | 65 |  |  |
| -0.62836 | 138.0768 | -0.00583 | -0.62723 | 66 |  |  |
| -0.62729 | 139.145 | -0.00581 | -0.6261 | 67 |  |  |
| -0.62622 | 140.2131 | -0.00578 | -0.62515 | 68 |  |  |
| -0.62515 | 141.2812 | -0.00576 | -0.62396 | 69 |  |  |
| -0.62408 | 142.3493 | -0.00574 | -0.62302 | 70 |  |  |
| -0.62302 | 143.4174 | -0.00572 | -0.62189 | 71 |  |  |
| -0.62195 | 144.4855 | -0.00569 | -0.62091 | 72 |  |  |
| -0.62088 | 145.5536 | -0.00567 | -0.61981 | 73 |  |  |
| -0.61981 | 146.6218 | -0.00565 | -0.61871 | 74 |  |  |
| -0.61874 | 147.6899 | -0.00562 | -0.61761 | 75 |  |  |
| -0.61768 | 148.758 | -0.0056 | -0.61664 | 76 |  |  |
| -0.61661 | 149.8261 | -0.00557 | -0.61557 | 77 |  |  |
| -0.61554 | 150.8942 | -0.00555 | -0.61447 | 78 |  |  |
| -0.61447 | 151.9623 | -0.00552 | -0.6134 | 79 |  |  |
| -0.6134 | 153.0305 | -0.0055 | -0.61237 | 80 |  |  |
| -0.61234 | 154.0986 | -0.00547 | -0.6113 | 81 |  |  |
| -0.61127 | 155.1667 | -0.00544 | -0.61023 | 82 |  |  |
| -0.6102 | 156.2348 | -0.00542 | -0.60922 | 83 |  |  |
| -0.60913 | 157.3029 | -0.00539 | -0.60809 | 84 |  |  |
| -0.60806 | 158.371 | -0.00536 | -0.60709 | 85 |  |  |
| -0.60699 | 159.4391 | -0.00534 | -0.60596 | 86 |  |  |
| -0.60593 | 160.5073 | -0.00531 | -0.60498 | 87 |  |  |
| -0.60486 | 161.5754 | -0.00528 | -0.60385 | 88 |  |  |
| -0.60379 | 162.6435 | -0.00525 | -0.60278 | 89 |  |  |
| -0.60272 | 163.7116 | -0.00522 | -0.60175 | 90 |  |  |
| -0.60165 | 164.7797 | -0.00519 | -0.60059 | 91 |  |  |
| -0.60059 | 165.8478 | -0.00515 | -0.5997 | 92 |  |  |
| -0.59952 | 166.9159 | -0.00512 | -0.59851 | 93 |  |  |
| -0.59845 | 167.9841 | -0.00509 | -0.59744 | 94 |  |  |
| -0.59738 | 169.0522 | -0.00505 | -0.59653 | 95 |  |  |
| -0.59631 | 170.1203 | -0.00502 | -0.59537 | 96 |  |  |
| -0.59525 | 171.1884 | -0.00498 | -0.59433 | 97 |  |  |
| -0.59418 | 172.2565 | -0.00494 | -0.59326 | 98 |  |  |
| -0.59311 | 173.3246 | -0.0049 | -0.59225 | 99 |  |  |
| -0.59204 | 174.3928 | -0.00486 | -0.59109 | 100 |  |  |
| -0.59097 | 175.4609 | -0.00481 | -0.59012 | 101 |  |  |
| -0.5899 | 176.529 | -0.00477 | -0.58893 | 102 |  |  |
| -0.58884 | 177.5971 | -0.00472 | -0.58804 | 103 |  |  |
| -0.58777 | 178.6652 | -0.00467 | -0.58707 | 104 |  |  |
| -0.5867 | 179.7333 | -0.00461 | -0.58588 | 105 |  |  |
| -0.58563 | 180.8014 | -0.00456 | -0.58487 | 106 |  |  |
| -0.58456 | 181.8696 | -0.0045 | -0.58377 | 107 |  |  |
| -0.5835 | 182.9377 | -0.00443 | -0.58276 | 108 |  |  |
| -0.58243 | 184.0058 | -0.00437 | -0.58167 | 109 |  |  |
| -0.58136 | 185.0739 | -0.0043 | -0.58063 | 110 |  |  |
| -0.58029 | 186.142 | -0.00422 | -0.57959 | 111 |  |  |
| -0.57922 | 187.2101 | -0.00414 | -0.57852 | 112 |  |  |
| -0.57816 | 188.2782 | -0.00406 | -0.57751 | 113 |  |  |
| -0.57709 | 189.3464 | -0.00398 | -0.57645 | 114 |  |  |
| -0.57602 | 190.4145 | -0.00389 | -0.57535 | 115 |  |  |
| -0.57495 | 191.4826 | -0.00381 | -0.57428 | 116 |  |  |
| -0.57388 | 192.5507 | -0.00372 | -0.5733 | 117 |  |  |
| -0.57281 | 193.6188 | -0.00363 | -0.57227 | 118 |  |  |
| -0.57175 | 194.6869 | -0.00354 | -0.57114 | 119 |  |  |
| -0.57068 | 195.7551 | -0.00346 | -0.57007 | 120 |  |  |
| -0.56961 | 196.8232 | -0.00337 | -0.56906 | 121 |  |  |
| -0.56854 | 197.8913 | -0.00329 | -0.56802 | 122 |  |  |
| -0.56747 | 198.9594 | -0.0032 | -0.56711 | 123 |  |  |
| -0.56641 | 200.0275 | -0.00312 | -0.56586 | 124 |  |  |
| -0.56534 | 201.0956 | -0.00304 | -0.56494 | 125 |  |  |
| -0.56427 | 202.1637 | -0.00297 | -0.56387 | 126 |  |  |
| -0.5632 | 203.2319 | -0.00289 | -0.56287 | 127 |  |  |
| -0.56213 | 204.3 | -0.00282 | -0.56177 | 128 |  |  |
| -0.56107 | 205.3681 | -0.00275 | -0.5607 | 129 |  |  |
| -0.56 | 206.4362 | -0.00267 | -0.55969 | 130 |  |  |
| -0.55893 | 207.5043 | -0.00261 | -0.55865 | 131 |  |  |
| -0.55786 | 208.5724 | -0.00254 | -0.5575 | 132 |  |  |
| -0.55679 | 209.6405 | -0.00247 | -0.5564 | 133 |  |  |
| -0.55573 | 210.7087 | -0.00241 | -0.55533 | 134 |  |  |
| -0.55466 | 211.7768 | -0.00235 | -0.55441 | 135 |  |  |
| -0.55359 | 212.8449 | -0.00229 | -0.55341 | 136 |  |  |
| -0.55252 | 213.913 | -0.00223 | -0.55225 | 137 |  |  |
| -0.55145 | 214.9811 | -0.00217 | -0.55124 | 138 |  |  |
| -0.55038 | 216.0492 | -0.00211 | -0.5502 | 139 |  |  |
| -0.54932 | 217.1174 | -0.00206 | -0.54919 | 140 |  |  |
| -0.54825 | 218.1855 | -0.00201 | -0.548 | 141 |  |  |
| -0.54718 | 219.2536 | -0.00195 | -0.54703 | 142 |  |  |
| -0.54611 | 220.3217 | -0.0019 | -0.5459 | 143 |  |  |
| -0.54504 | 221.3898 | -0.00185 | -0.54492 | 144 |  |  |
| -0.54398 | 222.4579 | -0.00181 | -0.54385 | 145 |  |  |
| -0.54291 | 223.526 | -0.00176 | -0.54276 | 146 |  |  |
| -0.54184 | 224.5942 | -0.00171 | -0.54163 | 147 |  |  |
| -0.54077 | 225.6623 | -0.00167 | -0.54065 | 148 |  |  |
| -0.5397 | 226.7304 | -0.00163 | -0.53964 | 149 |  |  |
| -0.53864 | 227.7985 | -0.00158 | -0.53857 | 150 |  |  |
| -0.53757 | 228.8666 | -0.00154 | -0.53745 | 151 |  |  |
| -0.5365 | 229.9347 | -0.0015 | -0.53641 | 152 |  |  |
| -0.53543 | 231.0028 | -0.00146 | -0.5354 | 153 |  |  |
| -0.53436 | 232.071 | -0.00143 | -0.53418 | 154 |  |  |
| -0.53329 | 233.1391 | -0.00139 | -0.53314 | 155 |  |  |
| -0.53223 | 234.2072 | -0.00135 | -0.53204 | 156 |  |  |
| -0.53116 | 235.2753 | -0.00132 | -0.53107 | 157 |  |  |
| -0.53009 | 236.3434 | -0.00128 | -0.52994 | 158 |  |  |
| -0.52902 | 237.4115 | -0.00125 | -0.52896 | 159 |  |  |
| -0.52795 | 238.4797 | -0.00122 | -0.52789 | 160 |  |  |
| -0.52689 | 239.5478 | -0.00119 | -0.52664 | 161 |  |  |
| -0.52582 | 240.6159 | -0.00115 | -0.52573 | 162 |  |  |
| -0.52475 | 241.684 | -0.00112 | -0.52475 | 163 |  |  |
| -0.52368 | 242.7521 | -0.00109 | -0.52365 | 164 |  |  |
| -0.52261 | 243.8202 | -0.00107 | -0.52261 | 165 |  |  |
| -0.52155 | 244.8883 | -0.00104 | -0.52161 | 166 |  |  |
| -0.52048 | 245.9565 | -0.00101 | -0.52063 | 167 |  |  |
| -0.51941 | 247.0246 | -0.00098 | -0.51935 | 168 |  |  |
| -0.51834 | 248.0927 | -0.00096 | -0.5184 | 169 |  |  |
| -0.51727 | 249.1608 | -0.00093 | -0.51727 | 170 |  |  |
| -0.5162 | 250.2289 | -0.00091 | -0.5163 | 171 |  |  |
| -0.51514 | 251.297 | -0.00088 | -0.51508 | 172 |  |  |
| -0.51407 | 252.3651 | -0.00086 | -0.51416 | 173 |  |  |
| -0.513 | 253.4333 | -0.00084 | -0.51297 | 174 |  |  |
| -0.51193 | 254.5014 | -0.00082 | -0.51202 | 175 |  |  |
| -0.51086 | 255.5695 | -0.00079 | -0.51089 | 176 |  |  |
| -0.5098 | 256.6376 | -0.00077 | -0.50974 | 177 |  |  |
| -0.50873 | 257.7057 | -0.00075 | -0.50879 | 178 |  |  |
| -0.50766 | 258.7738 | -0.00073 | -0.50772 | 179 |  |  |
| -0.50659 | 259.842 | -0.00071 | -0.50668 | 180 |  |  |
| -0.50552 | 260.9101 | -0.00069 | -0.50552 | 181 |  |  |
| -0.50446 | 261.9782 | -0.00067 | -0.50443 | 182 |  |  |
| -0.50339 | 263.0463 | -0.00066 | -0.50333 | 183 |  |  |
| -0.50232 | 264.1144 | -0.00064 | -0.50241 | 184 |  |  |
| -0.50125 | 265.1825 | -0.00062 | -0.5014 | 185 |  |  |
| -0.50018 | 266.2506 | -0.0006 | -0.50009 | 186 |  |  |
| -0.49911 | 267.3188 | -0.00059 | -0.49911 | 187 |  |  |
| -0.49805 | 268.3869 | -0.00057 | -0.49811 | 188 |  |  |
| -0.49698 | 269.455 | -0.00055 | -0.4971 | 189 |  |  |
| -0.49591 | 270.5231 | -0.00054 | -0.49597 | 190 |  |  |
| -0.49484 | 271.5912 | -0.00052 | -0.49496 | 191 |  |  |
| -0.49377 | 272.6593 | -0.00051 | -0.4938 | 192 |  |  |
| -0.49271 | 273.7274 | -0.00049 | -0.49274 | 193 |  |  |
| -0.49164 | 274.7956 | -0.00048 | -0.49182 | 194 |  |  |
| -0.49057 | 275.8637 | -0.00047 | -0.49063 | 195 |  |  |
| -0.4895 | 276.9318 | -0.00045 | -0.48962 | 196 |  |  |
| -0.48843 | 277.9999 | -0.00044 | -0.48831 | 197 |  |  |
| -0.48737 | 279.068 | -0.00043 | -0.48746 | 198 |  |  |
| -0.4863 | 280.1361 | -0.00041 | -0.48642 | 199 |  |  |
| -0.48523 | 281.2043 | -0.0004 | -0.48514 | 200 |  |  |
| -0.48416 | 282.2724 | -0.00039 | -0.48431 | 201 |  |  |
| -0.48309 | 283.3405 | -0.00038 | -0.48312 | 202 |  |  |
| -0.48203 | 284.4086 | -0.00037 | -0.48218 | 203 |  |  |
| -0.48096 | 285.4767 | -0.00035 | -0.48105 | 204 |  |  |
| -0.47989 | 286.5448 | -0.00034 | -0.47992 | 205 |  |  |
| -0.47882 | 287.6129 | -0.00033 | -0.47894 | 206 |  |  |
| -0.47775 | 288.6811 | -0.00032 | -0.47775 | 207 |  |  |
| -0.47668 | 289.7492 | -0.00031 | -0.47672 | 208 |  |  |
| -0.47562 | 290.8173 | -0.0003 | -0.4758 | 209 |  |  |
| -0.47455 | 291.8854 | -0.00029 | -0.47461 | 210 |  |  |
| -0.47348 | 292.9535 | -0.00028 | -0.47357 | 211 |  |  |
| -0.47241 | 294.0216 | -0.00027 | -0.47238 | 212 |  |  |
| -0.47134 | 295.0897 | -0.00026 | -0.47144 | 213 |  |  |
| -0.47028 | 296.1579 | -0.00025 | -0.47049 | 214 |  |  |
| -0.46921 | 297.226 | -0.00024 | -0.46936 | 215 |  |  |
| -0.46814 | 298.2941 | -0.00023 | -0.46817 | 216 |  |  |
| -0.46707 | 299.3622 | -0.00022 | -0.46719 | 217 |  |  |
| -0.466 | 300.4303 | -0.00022 | -0.46625 | 218 |  |  |
| -0.46494 | 301.4984 | -0.00021 | -0.46518 | 219 |  |  |
| -0.46387 | 302.5666 | -0.0002 | -0.46402 | 220 |  |  |
| -0.4628 | 303.6347 | -0.00019 | -0.46292 | 221 |  |  |
| -0.46173 | 304.7028 | -0.00018 | -0.46176 | 222 |  |  |
| -0.46066 | 305.7709 | -0.00017 | -0.46066 | 223 |  |  |
| -0.45959 | 306.839 | -0.00017 | -0.45966 | 224 |  |  |
| -0.45853 | 307.9071 | -0.00016 | -0.45847 | 225 |  |  |
| -0.45746 | 308.9752 | -0.00015 | -0.45758 | 226 |  |  |
| -0.45639 | 310.0434 | -0.00014 | -0.4566 | 227 |  |  |
| -0.45532 | 311.1115 | -0.00013 | -0.45544 | 228 |  |  |
| -0.45425 | 312.1796 | -0.00013 | -0.45432 | 229 |  |  |
| -0.45319 | 313.2477 | -0.00012 | -0.45325 | 230 |  |  |
| -0.45212 | 314.3158 | -0.00011 | -0.45224 | 231 |  |  |
| -0.45105 | 315.3839 | -0.0001 | -0.45114 | 232 |  |  |
| -0.44998 | 316.452 | -9.6E-05 | -0.45007 | 233 |  |  |
| -0.44891 | 317.5202 | -8.8E-05 | -0.44901 | 234 |  |  |
| -0.44785 | 318.5883 | -8.1E-05 | -0.44797 | 235 |  |  |
| -0.44678 | 319.6564 | -7.4E-05 | -0.44687 | 236 |  |  |
| -0.44571 | 320.7245 | -6.6E-05 | -0.44583 | 237 |  |  |
| -0.44464 | 321.7926 | -5.9E-05 | -0.44455 | 238 |  |  |
| -0.44357 | 322.8607 | -5.1E-05 | -0.44363 | 239 |  |  |
| -0.4425 | 323.9289 | -4.4E-05 | -0.44263 | 240 |  |  |
| -0.44144 | 324.997 | -3.6E-05 | -0.44135 | 241 |  |  |
| -0.44037 | 326.0651 | -2.8E-05 | -0.44061 | 242 |  |  |
| -0.4393 | 327.1332 | -2.1E-05 | -0.43933 | 243 |  |  |
| -0.43823 | 328.2013 | -1.4E-05 | -0.43835 | 244 |  |  |
| -0.43716 | 329.2694 | -6.7E-06 | -0.43738 | 245 |  |  |
| -0.4361 | 330.3375 | 7.45E-07 | -0.43622 | 246 |  |  |
| -0.43503 | 331.4057 | 8.39E-06 | -0.43509 | 247 |  |  |
| -0.43396 | 332.4738 | 1.55E-05 | -0.43423 | 248 |  |  |
| -0.43289 | 333.5419 | 2.35E-05 | -0.43307 | 249 |  |  |
| -0.43182 | 334.61 | 3.09E-05 | -0.43188 | 250 |  |  |
| -0.43076 | 335.6781 | 3.87E-05 | -0.43109 | 251 |  |  |
| -0.42969 | 336.7462 | 4.72E-05 | -0.42993 | 252 |  |  |
| -0.42862 | 337.8143 | 5.51E-05 | -0.42883 | 253 |  |  |
| -0.42755 | 338.8825 | 6.36E-05 | -0.42776 | 254 |  |  |
| -0.42648 | 339.9506 | 7.19E-05 | -0.42676 | 255 |  |  |
| -0.42542 | 341.0187 | 8.03E-05 | -0.42572 | 256 |  |  |
| -0.42435 | 342.0868 | 8.87E-05 | -0.42453 | 257 |  |  |
| -0.42328 | 343.1549 | 9.74E-05 | -0.42346 | 258 |  |  |
| -0.42221 | 344.223 | 0.000106 | -0.42236 | 259 |  |  |
| -0.42114 | 345.2912 | 0.000115 | -0.42154 | 260 |  |  |
| -0.42007 | 346.3593 | 0.000124 | -0.42035 | 261 |  |  |
| -0.41901 | 347.4274 | 0.000134 | -0.4194 | 262 |  |  |
| -0.41794 | 348.4955 | 0.000143 | -0.41821 | 263 |  |  |
| -0.41687 | 349.5636 | 0.000153 | -0.41727 | 264 |  |  |
| -0.4158 | 350.6317 | 0.000163 | -0.41617 | 265 |  |  |
| -0.41473 | 351.6998 | 0.000173 | -0.41498 | 266 |  |  |
| -0.41367 | 352.768 | 0.000183 | -0.41403 | 267 |  |  |
| -0.4126 | 353.8361 | 0.000194 | -0.4129 | 268 |  |  |
| -0.41153 | 354.9042 | 0.000205 | -0.41202 | 269 |  |  |
| -0.41046 | 355.9723 | 0.000216 | -0.41086 | 270 |  |  |
| -0.40939 | 357.0404 | 0.000228 | -0.40979 | 271 |  |  |
| -0.40833 | 358.1085 | 0.000239 | -0.40887 | 272 |  |  |
| -0.40726 | 359.1766 | 0.000252 | -0.4079 | 273 |  |  |
| -0.40619 | 360.2448 | 0.000264 | -0.40668 | 274 |  |  |
| -0.40512 | 361.3129 | 0.000277 | -0.40555 | 275 |  |  |
| -0.40405 | 362.381 | 0.000291 | -0.40454 | 276 |  |  |
| -0.40298 | 363.4491 | 0.000304 | -0.40359 | 277 |  |  |
| -0.40192 | 364.5172 | 0.000319 | -0.40201 | 278 |  |  |
| -0.40085 | 365.5853 | 0.000334 | -0.40097 | 279 |  |  |
| -0.39978 | 366.6535 | 0.000349 | -0.3999 | 280 |  |  |
| -0.39871 | 367.7216 | 0.000365 | -0.39883 | 281 |  |  |
| -0.39764 | 368.7897 | 0.000381 | -0.39783 | 282 |  |  |
| -0.39658 | 369.8578 | 0.000398 | -0.39664 | 283 |  |  |
| -0.39551 | 370.9259 | 0.000416 | -0.39554 | 284 |  |  |
| -0.39444 | 371.994 | 0.000434 | -0.3945 | 285 |  |  |
| -0.39337 | 373.0621 | 0.000452 | -0.3934 | 286 |  |  |
| -0.3923 | 374.1303 | 0.000472 | -0.39236 | 287 |  |  |
| -0.39124 | 375.1984 | 0.000492 | -0.39133 | 288 |  |  |
| -0.39017 | 376.2665 | 0.000513 | -0.39029 | 289 |  |  |
| -0.3891 | 377.3346 | 0.000535 | -0.38934 | 290 |  |  |
| -0.38803 | 378.4027 | 0.000558 | -0.38824 | 291 |  |  |
| -0.38696 | 379.4708 | 0.000582 | -0.38724 | 292 |  |  |
| -0.38589 | 380.5389 | 0.000607 | -0.38614 | 293 |  |  |
| -0.38483 | 381.6071 | 0.000632 | -0.38498 | 294 |  |  |
| -0.38376 | 382.6752 | 0.000659 | -0.38412 | 295 |  |  |
| -0.38269 | 383.7433 | 0.000687 | -0.38284 | 296 |  |  |
| -0.38162 | 384.8114 | 0.000717 | -0.3819 | 297 |  |  |
| -0.38055 | 385.8795 | 0.000747 | -0.38074 | 298 |  |  |
| -0.37949 | 386.9476 | 0.000779 | -0.37979 | 299 |  |  |
| -0.37842 | 388.0158 | 0.000812 | -0.37863 | 300 |  |  |
| -0.37735 | 389.0839 | 0.000848 | -0.37756 | 301 |  |  |
| -0.37628 | 390.152 | 0.000884 | -0.37662 | 302 |  |  |
| -0.37521 | 391.2201 | 0.000923 | -0.37543 | 303 |  |  |
| -0.37415 | 392.2882 | 0.000963 | -0.37442 | 304 |  |  |
| -0.37308 | 393.3563 | 0.001005 | -0.37344 | 305 |  |  |
| -0.37201 | 394.4244 | 0.001049 | -0.37234 | 306 |  |  |
| -0.37094 | 395.4926 | 0.001095 | -0.37122 | 307 |  |  |
| -0.36987 | 396.5607 | 0.001144 | -0.37021 | 308 |  |  |
| -0.3688 | 397.6288 | 0.001195 | -0.36905 | 309 |  |  |
| -0.36774 | 398.6969 | 0.00125 | -0.36804 | 310 |  |  |
| -0.36667 | 399.765 | 0.001306 | -0.36694 | 311 |  |  |
| -0.3656 | 400.8331 | 0.001366 | -0.36591 | 312 |  |  |
| -0.36453 | 401.9012 | 0.001429 | -0.36487 | 313 |  |  |
| -0.36346 | 402.9694 | 0.001494 | -0.36374 | 314 |  |  |
| -0.3624 | 404.0375 | 0.001563 | -0.3627 | 315 |  |  |
| -0.36133 | 405.1056 | 0.001636 | -0.36172 | 316 |  |  |
| -0.36026 | 406.1737 | 0.001714 | -0.36063 | 317 |  |  |
| -0.35919 | 407.2418 | 0.001794 | -0.35965 | 318 |  |  |
| -0.35812 | 408.3099 | 0.00188 | -0.35846 | 319 |  |  |
| -0.35706 | 409.3781 | 0.001971 | -0.35739 | 320 |  |  |
| -0.35599 | 410.4462 | 0.002065 | -0.35632 | 321 |  |  |
| -0.35492 | 411.5143 | 0.002163 | -0.35519 | 322 |  |  |
| -0.35385 | 412.5824 | 0.002267 | -0.35428 | 323 |  |  |
| -0.35278 | 413.6505 | 0.002377 | -0.35324 | 324 |  |  |
| -0.35172 | 414.7186 | 0.002493 | -0.35211 | 325 |  |  |
| -0.35065 | 415.7867 | 0.002616 | -0.35117 | 326 |  |  |
| -0.34958 | 416.8549 | 0.002747 | -0.3501 | 327 |  |  |
| -0.34851 | 417.923 | 0.002885 | -0.34894 | 328 |  |  |
| -0.34744 | 418.9911 | 0.003031 | -0.3479 | 329 |  |  |
| -0.34637 | 420.0592 | 0.003185 | -0.34695 | 330 |  |  |
| -0.34531 | 421.1273 | 0.003347 | -0.34589 | 331 |  |  |
| -0.34424 | 422.1954 | 0.003515 | -0.34479 | 332 |  |  |
| -0.34317 | 423.2635 | 0.003688 | -0.34381 | 333 |  |  |
| -0.3421 | 424.3317 | 0.003863 | -0.34274 | 334 |  |  |
| -0.34103 | 425.3998 | 0.004034 | -0.3418 | 335 |  |  |
| -0.33997 | 426.4679 | 0.004197 | -0.34076 | 336 |  |  |
| -0.3389 | 427.536 | 0.004348 | -0.33963 | 337 |  |  |
| -0.33783 | 428.6041 | 0.004484 | -0.33868 | 338 |  |  |
| -0.33676 | 429.6722 | 0.004606 | -0.33752 | 339 |  |  |
| -0.33569 | 430.7404 | 0.004716 | -0.33652 | 340 |  |  |
| -0.33463 | 431.8085 | 0.004816 | -0.33548 | 341 |  |  |
| -0.33356 | 432.8766 | 0.004908 | -0.33447 | 342 |  |  |
| -0.33249 | 433.9447 | 0.004993 | -0.33344 | 343 |  |  |
| -0.33142 | 435.0128 | 0.005073 | -0.33234 | 344 |  |  |
| -0.33035 | 436.0809 | 0.005148 | -0.33139 | 345 |  |  |
| -0.32928 | 437.149 | 0.005219 | -0.33026 | 346 |  |  |
| -0.32822 | 438.2172 | 0.005288 | -0.32922 | 347 |  |  |
| -0.32715 | 439.2853 | 0.005354 | -0.32822 | 348 |  |  |
| -0.32608 | 440.3534 | 0.005417 | -0.32718 | 349 |  |  |
| -0.32501 | 441.4215 | 0.005479 | -0.32617 | 350 |  |  |
| -0.32394 | 442.4896 | 0.005538 | -0.32495 | 351 |  |  |
| -0.32288 | 443.5577 | 0.005597 | -0.32394 | 352 |  |  |
| -0.32181 | 444.6258 | 0.005655 | -0.32291 | 353 |  |  |
| -0.32074 | 445.694 | 0.005712 | -0.32187 | 354 |  |  |
| -0.31967 | 446.7621 | 0.005768 | -0.32092 | 355 |  |  |
| -0.3186 | 447.8302 | 0.005823 | -0.31982 | 356 |  |  |
| -0.31754 | 448.8983 | 0.005877 | -0.31873 | 357 |  |  |
| -0.31647 | 449.9664 | 0.005932 | -0.31763 | 358 |  |  |
| -0.3154 | 451.0345 | 0.005986 | -0.31665 | 359 |  |  |
| -0.31433 | 452.1027 | 0.006039 | -0.31555 | 360 |  |  |
| -0.31326 | 453.1708 | 0.006092 | -0.31442 | 361 |  |  |
| -0.31219 | 454.2389 | 0.006145 | -0.31342 | 362 |  |  |
| -0.31113 | 455.307 | 0.006197 | -0.31244 | 363 |  |  |
| -0.31006 | 456.3751 | 0.006249 | -0.31131 | 364 |  |  |
| -0.30899 | 457.4432 | 0.0063 | -0.3103 | 365 |  |  |
| -0.30792 | 458.5113 | 0.00635 | -0.30911 | 366 |  |  |
| -0.30685 | 459.5795 | 0.0064 | -0.30823 | 367 |  |  |
| -0.30579 | 460.6476 | 0.006449 | -0.30707 | 368 |  |  |
| -0.30472 | 461.7157 | 0.006497 | -0.306 | 369 |  |  |
| -0.30365 | 462.7838 | 0.006544 | -0.30496 | 370 |  |  |
| -0.30258 | 463.8519 | 0.00659 | -0.30374 | 371 |  |  |
| -0.30151 | 464.92 | 0.006635 | -0.30283 | 372 |  |  |
| -0.30045 | 465.9881 | 0.006679 | -0.30176 | 373 |  |  |
| -0.29938 | 467.0563 | 0.006722 | -0.30069 | 374 |  |  |
| -0.29831 | 468.1244 | 0.006763 | -0.29971 | 375 |  |  |
| -0.29724 | 469.1925 | 0.006804 | -0.29849 | 376 |  |  |
| -0.29617 | 470.2606 | 0.006843 | -0.29755 | 377 |  |  |
| -0.2951 | 471.3287 | 0.006881 | -0.29654 | 378 |  |  |
| -0.29404 | 472.3968 | 0.006918 | -0.29547 | 379 |  |  |
| -0.29297 | 473.465 | 0.006953 | -0.29434 | 380 |  |  |
| -0.2919 | 474.5331 | 0.006988 | -0.29343 | 381 |  |  |
| -0.29083 | 475.6012 | 0.007021 | -0.2923 | 382 |  |  |
| -0.28976 | 476.6693 | 0.007054 | -0.29117 | 383 |  |  |
| -0.2887 | 477.7374 | 0.007085 | -0.29016 | 384 |  |  |
| -0.28763 | 478.8055 | 0.007115 | -0.28906 | 385 |  |  |
| -0.28656 | 479.8736 | 0.007144 | -0.28799 | 386 |  |  |
| -0.28549 | 480.9418 | 0.007172 | -0.28693 | 387 |  |  |
| -0.28442 | 482.0099 | 0.0072 | -0.28595 | 388 |  |  |
| -0.28336 | 483.078 | 0.007226 | -0.28485 | 389 |  |  |
| -0.28229 | 484.1461 | 0.007251 | -0.28391 | 390 |  |  |
| -0.28122 | 485.2142 | 0.007275 | -0.28271 | 391 |  |  |
| -0.28015 | 486.2823 | 0.007298 | -0.28168 | 392 |  |  |
| -0.27908 | 487.3504 | 0.007321 | -0.28064 | 393 |  |  |
| -0.27802 | 488.4186 | 0.007343 | -0.27951 | 394 |  |  |
| -0.27695 | 489.4867 | 0.007365 | -0.27844 | 395 |  |  |
| -0.27588 | 490.5548 | 0.007386 | -0.27744 | 396 |  |  |
| -0.27481 | 491.6229 | 0.007406 | -0.2764 | 397 |  |  |
| -0.27374 | 492.691 | 0.007426 | -0.27524 | 398 |  |  |
| -0.27267 | 493.7591 | 0.007445 | -0.2742 | 399 |  |  |
| -0.27161 | 494.8273 | 0.007464 | -0.27316 | 400 |  |  |
| -0.27054 | 495.8954 | 0.007483 | -0.27209 | 401 |  |  |
| -0.26947 | 496.9635 | 0.007501 | -0.27109 | 402 |  |  |
| -0.2684 | 498.0316 | 0.007518 | -0.26987 | 403 |  |  |
| -0.26733 | 499.0997 | 0.007536 | -0.26886 | 404 |  |  |
| -0.26627 | 500.1678 | 0.007553 | -0.26782 | 405 |  |  |
| -0.2652 | 501.2359 | 0.00757 | -0.26685 | 406 |  |  |
| -0.26413 | 502.3041 | 0.007586 | -0.26572 | 407 |  |  |
| -0.26306 | 503.3722 | 0.007603 | -0.26459 | 408 |  |  |
| -0.26199 | 504.4403 | 0.007619 | -0.26349 | 409 |  |  |
| -0.26093 | 505.5084 | 0.007635 | -0.26245 | 410 |  |  |
| -0.25986 | 506.5765 | 0.007651 | -0.2616 | 411 |  |  |
| -0.25879 | 507.6446 | 0.007666 | -0.26047 | 412 |  |  |
| -0.25772 | 508.7127 | 0.007681 | -0.25931 | 413 |  |  |
| -0.25665 | 509.7809 | 0.007695 | -0.2583 | 414 |  |  |
| -0.25558 | 510.849 | 0.00771 | -0.25723 | 415 |  |  |
| -0.25452 | 511.9171 | 0.007724 | -0.25616 | 416 |  |  |
| -0.25345 | 512.9852 | 0.007738 | -0.2551 | 417 |  |  |
| -0.25238 | 514.0533 | 0.007753 | -0.25406 | 418 |  |  |
| -0.25131 | 515.1214 | 0.007767 | -0.25311 | 419 |  |  |
| -0.25024 | 516.1896 | 0.007781 | -0.25177 | 420 |  |  |
| -0.24918 | 517.2577 | 0.007795 | -0.25082 | 421 |  |  |
| -0.24811 | 518.3258 | 0.007808 | -0.24969 | 422 |  |  |
| -0.24704 | 519.3939 | 0.007821 | -0.24863 | 423 |  |  |
| -0.24597 | 520.462 | 0.007834 | -0.24771 | 424 |  |  |
| -0.2449 | 521.5301 | 0.007847 | -0.24649 | 425 |  |  |
| -0.24384 | 522.5982 | 0.00786 | -0.24548 | 426 |  |  |
| -0.24277 | 523.6664 | 0.007872 | -0.24448 | 427 |  |  |
| -0.2417 | 524.7345 | 0.007884 | -0.2435 | 428 |  |  |
| -0.24063 | 525.8026 | 0.007897 | -0.2424 | 429 |  |  |
| -0.23956 | 526.8707 | 0.007909 | -0.24118 | 430 |  |  |
| -0.23849 | 527.9388 | 0.007921 | -0.24008 | 431 |  |  |
| -0.23743 | 529.0069 | 0.007933 | -0.2392 | 432 |  |  |
| -0.23636 | 530.075 | 0.007945 | -0.23804 | 433 |  |  |
| -0.23529 | 531.1432 | 0.007957 | -0.237 | 434 |  |  |
| -0.23422 | 532.2113 | 0.007967 | -0.23602 | 435 |  |  |
| -0.23315 | 533.2794 | 0.007978 | -0.23486 | 436 |  |  |
| -0.23209 | 534.3475 | 0.007989 | -0.23373 | 437 |  |  |
| -0.23102 | 535.4156 | 0.007999 | -0.23273 | 438 |  |  |
| -0.22995 | 536.4837 | 0.00801 | -0.23175 | 439 |  |  |
| -0.22888 | 537.5519 | 0.008022 | -0.23056 | 440 |  |  |
| -0.22781 | 538.62 | 0.008033 | -0.22952 | 441 |  |  |
| -0.22675 | 539.6881 | 0.008043 | -0.22839 | 442 |  |  |
| -0.22568 | 540.7562 | 0.008054 | -0.22739 | 443 |  |  |
| -0.22461 | 541.8243 | 0.008063 | -0.22632 | 444 |  |  |
| -0.22354 | 542.8924 | 0.008075 | -0.22543 | 445 |  |  |
| -0.22247 | 543.9605 | 0.008084 | -0.22418 | 446 |  |  |
| -0.22141 | 545.0287 | 0.008094 | -0.22321 | 447 |  |  |
| -0.22034 | 546.0968 | 0.008104 | -0.22202 | 448 |  |  |
| -0.21927 | 547.1649 | 0.008113 | -0.22098 | 449 |  |  |
| -0.2182 | 548.233 | 0.008122 | -0.21994 | 450 |  |  |
| -0.21713 | 549.3011 | 0.008134 | -0.21884 | 451 |  |  |
| -0.21606 | 550.3692 | 0.008144 | -0.21793 | 452 |  |  |
| -0.215 | 551.4373 | 0.008154 | -0.21674 | 453 |  |  |
| -0.21393 | 552.5055 | 0.008163 | -0.21567 | 454 |  |  |
| -0.21286 | 553.5736 | 0.008173 | -0.2146 | 455 |  |  |
| -0.21179 | 554.6417 | 0.008181 | -0.21353 | 456 |  |  |
| -0.21072 | 555.7098 | 0.008189 | -0.21246 | 457 |  |  |
| -0.20966 | 556.7779 | 0.008197 | -0.21161 | 458 |  |  |
| -0.20859 | 557.846 | 0.008206 | -0.21033 | 459 |  |  |
| -0.20752 | 558.9142 | 0.008214 | -0.20929 | 460 |  |  |
| -0.20645 | 559.9823 | 0.008222 | -0.20816 | 461 |  |  |
| -0.20538 | 561.0504 | 0.008231 | -0.20706 | 462 |  |  |
| -0.20432 | 562.1185 | 0.008241 | -0.20599 | 463 |  |  |
| -0.20325 | 563.1866 | 0.008249 | -0.20499 | 464 |  |  |
| -0.20218 | 564.2547 | 0.008257 | -0.20392 | 465 |  |  |
| -0.20111 | 565.3228 | 0.008267 | -0.20294 | 466 |  |  |
| -0.20004 | 566.391 | 0.008276 | -0.20193 | 467 |  |  |
| -0.19897 | 567.4591 | 0.008286 | -0.20081 | 468 |  |  |
| -0.19791 | 568.5272 | 0.008295 | -0.19974 | 469 |  |  |

Sample: *Maurorum*, Concentration (ppm): 600, Immersion time: 24h

| Potential applied(V) | Time (s) | WE(1).  Current (A) | WE(1).  Potential (V) | Index |  |  |
| --- | --- | --- | --- | --- | --- | --- |
| -0.7106 | 68.62636 | -0.00093 | -0.70856 | 1 |  |  |
| -0.70953 | 69.69448 | -0.00763 | -0.70795 | 2 |  |  |
| -0.70847 | 70.76259 | -0.00759 | -0.70685 | 3 |  |  |
| -0.7074 | 71.83071 | -0.00756 | -0.70578 | 4 |  |  |
| -0.70633 | 72.89882 | -0.00753 | -0.70471 | 5 |  |  |
| -0.70526 | 73.96694 | -0.0075 | -0.70377 | 6 |  |  |
| -0.70419 | 75.03505 | -0.00748 | -0.70264 | 7 |  |  |
| -0.70313 | 76.10317 | -0.00746 | -0.70151 | 8 |  |  |
| -0.70206 | 77.17128 | -0.00744 | -0.70047 | 9 |  |  |
| -0.70099 | 78.2394 | -0.00741 | -0.6994 | 10 |  |  |
| -0.69992 | 79.30751 | -0.00739 | -0.69833 | 11 |  |  |
| -0.69885 | 80.37563 | -0.00737 | -0.69727 | 12 |  |  |
| -0.69778 | 81.44374 | -0.00735 | -0.6962 | 13 |  |  |
| -0.69672 | 82.51186 | -0.00733 | -0.69516 | 14 |  |  |
| -0.69565 | 83.57997 | -0.00731 | -0.69412 | 15 |  |  |
| -0.69458 | 84.64809 | -0.00729 | -0.69302 | 16 |  |  |
| -0.69351 | 85.7162 | -0.00727 | -0.69189 | 17 |  |  |
| -0.69244 | 86.78432 | -0.00725 | -0.69092 | 18 |  |  |
| -0.69138 | 87.85243 | -0.00723 | -0.68997 | 19 |  |  |
| -0.69031 | 88.92055 | -0.00721 | -0.68869 | 20 |  |  |
| -0.68924 | 89.98866 | -0.00719 | -0.68765 | 21 |  |  |
| -0.68817 | 91.05678 | -0.00717 | -0.68661 | 22 |  |  |
| -0.6871 | 92.12489 | -0.00716 | -0.68561 | 23 |  |  |
| -0.68604 | 93.19301 | -0.00714 | -0.68454 | 24 |  |  |
| -0.68497 | 94.26112 | -0.00712 | -0.68344 | 25 |  |  |
| -0.6839 | 95.32924 | -0.0071 | -0.6824 | 26 |  |  |
| -0.68283 | 96.39735 | -0.00708 | -0.68143 | 27 |  |  |
| -0.68176 | 97.46547 | -0.00706 | -0.68027 | 28 |  |  |
| -0.68069 | 98.53358 | -0.00704 | -0.67911 | 29 |  |  |
| -0.67963 | 99.6017 | -0.00702 | -0.67822 | 30 |  |  |
| -0.67856 | 100.6698 | -0.00701 | -0.67706 | 31 |  |  |
| -0.67749 | 101.7379 | -0.00699 | -0.67609 | 32 |  |  |
| -0.67642 | 102.806 | -0.00697 | -0.67499 | 33 |  |  |
| -0.67535 | 103.8742 | -0.00695 | -0.67386 | 34 |  |  |
| -0.67429 | 104.9423 | -0.00693 | -0.67288 | 35 |  |  |
| -0.67322 | 106.0104 | -0.00691 | -0.67181 | 36 |  |  |
| -0.67215 | 107.0785 | -0.00689 | -0.67072 | 37 |  |  |
| -0.67108 | 108.1466 | -0.00688 | -0.66968 | 38 |  |  |
| -0.67001 | 109.2147 | -0.00686 | -0.66855 | 39 |  |  |
| -0.66895 | 110.2828 | -0.00684 | -0.66751 | 40 |  |  |
| -0.66788 | 111.351 | -0.00682 | -0.66653 | 41 |  |  |
| -0.66681 | 112.4191 | -0.0068 | -0.66541 | 42 |  |  |
| -0.66574 | 113.4872 | -0.00678 | -0.66437 | 43 |  |  |
| -0.66467 | 114.5553 | -0.00677 | -0.66333 | 44 |  |  |
| -0.6636 | 115.6234 | -0.00675 | -0.66232 | 45 |  |  |
| -0.66254 | 116.6915 | -0.00673 | -0.66107 | 46 |  |  |
| -0.66147 | 117.7597 | -0.00671 | -0.66019 | 47 |  |  |
| -0.6604 | 118.8278 | -0.00669 | -0.65909 | 48 |  |  |
| -0.65933 | 119.8959 | -0.00667 | -0.65793 | 49 |  |  |
| -0.65826 | 120.964 | -0.00665 | -0.65689 | 50 |  |  |
| -0.6572 | 122.0321 | -0.00664 | -0.65588 | 51 |  |  |
| -0.65613 | 123.1002 | -0.00662 | -0.65479 | 52 |  |  |
| -0.65506 | 124.1683 | -0.0066 | -0.65381 | 53 |  |  |
| -0.65399 | 125.2365 | -0.00658 | -0.65253 | 54 |  |  |
| -0.65292 | 126.3046 | -0.00656 | -0.65158 | 55 |  |  |
| -0.65186 | 127.3727 | -0.00654 | -0.65057 | 56 |  |  |
| -0.65079 | 128.4408 | -0.00652 | -0.64948 | 57 |  |  |
| -0.64972 | 129.5089 | -0.0065 | -0.64841 | 58 |  |  |
| -0.64865 | 130.577 | -0.00648 | -0.6474 | 59 |  |  |
| -0.64758 | 131.6451 | -0.00647 | -0.64633 | 60 |  |  |
| -0.64651 | 132.7133 | -0.00645 | -0.64526 | 61 |  |  |
| -0.64545 | 133.7814 | -0.00643 | -0.64423 | 62 |  |  |
| -0.64438 | 134.8495 | -0.00641 | -0.64307 | 63 |  |  |
| -0.64331 | 135.9176 | -0.00639 | -0.64206 | 64 |  |  |
| -0.64224 | 136.9857 | -0.00637 | -0.64099 | 65 |  |  |
| -0.64117 | 138.0538 | -0.00635 | -0.63998 | 66 |  |  |
| -0.64011 | 139.122 | -0.00633 | -0.63879 | 67 |  |  |
| -0.63904 | 140.1901 | -0.00631 | -0.63788 | 68 |  |  |
| -0.63797 | 141.2582 | -0.00629 | -0.63672 | 69 |  |  |
| -0.6369 | 142.3263 | -0.00627 | -0.63568 | 70 |  |  |
| -0.63583 | 143.3944 | -0.00625 | -0.63458 | 71 |  |  |
| -0.63477 | 144.4625 | -0.00623 | -0.63348 | 72 |  |  |
| -0.6337 | 145.5306 | -0.00621 | -0.63251 | 73 |  |  |
| -0.63263 | 146.5988 | -0.00619 | -0.63147 | 74 |  |  |
| -0.63156 | 147.6669 | -0.00617 | -0.63043 | 75 |  |  |
| -0.63049 | 148.735 | -0.00615 | -0.62936 | 76 |  |  |
| -0.62943 | 149.8031 | -0.00613 | -0.6283 | 77 |  |  |
| -0.62836 | 150.8712 | -0.00611 | -0.62723 | 78 |  |  |
| -0.62729 | 151.9393 | -0.00609 | -0.62625 | 79 |  |  |
| -0.62622 | 153.0074 | -0.00607 | -0.62509 | 80 |  |  |
| -0.62515 | 154.0756 | -0.00604 | -0.62399 | 81 |  |  |
| -0.62408 | 155.1437 | -0.00602 | -0.62299 | 82 |  |  |
| -0.62302 | 156.2118 | -0.006 | -0.62192 | 83 |  |  |
| -0.62195 | 157.2799 | -0.00598 | -0.62076 | 84 |  |  |
| -0.62088 | 158.348 | -0.00596 | -0.61981 | 85 |  |  |
| -0.61981 | 159.4161 | -0.00594 | -0.61868 | 86 |  |  |
| -0.61874 | 160.4843 | -0.00591 | -0.61758 | 87 |  |  |
| -0.61768 | 161.5524 | -0.00589 | -0.61652 | 88 |  |  |
| -0.61661 | 162.6205 | -0.00587 | -0.61551 | 89 |  |  |
| -0.61554 | 163.6886 | -0.00585 | -0.61441 | 90 |  |  |
| -0.61447 | 164.7567 | -0.00582 | -0.6134 | 91 |  |  |
| -0.6134 | 165.8248 | -0.0058 | -0.61234 | 92 |  |  |
| -0.61234 | 166.8929 | -0.00578 | -0.61127 | 93 |  |  |
| -0.61127 | 167.9611 | -0.00575 | -0.61014 | 94 |  |  |
| -0.6102 | 169.0292 | -0.00573 | -0.60913 | 95 |  |  |
| -0.60913 | 170.0973 | -0.00571 | -0.60806 | 96 |  |  |
| -0.60806 | 171.1654 | -0.00568 | -0.60706 | 97 |  |  |
| -0.60699 | 172.2335 | -0.00566 | -0.60599 | 98 |  |  |
| -0.60593 | 173.3016 | -0.00563 | -0.60486 | 99 |  |  |
| -0.60486 | 174.3697 | -0.00561 | -0.60385 | 100 |  |  |
| -0.60379 | 175.4379 | -0.00558 | -0.60272 | 101 |  |  |
| -0.60272 | 176.506 | -0.00555 | -0.60168 | 102 |  |  |
| -0.60165 | 177.5741 | -0.00553 | -0.60065 | 103 |  |  |
| -0.60059 | 178.6422 | -0.0055 | -0.59952 | 104 |  |  |
| -0.59952 | 179.7103 | -0.00547 | -0.59845 | 105 |  |  |
| -0.59845 | 180.7784 | -0.00545 | -0.59744 | 106 |  |  |
| -0.59738 | 181.8466 | -0.00542 | -0.59637 | 107 |  |  |
| -0.59631 | 182.9147 | -0.00539 | -0.59534 | 108 |  |  |
| -0.59525 | 183.9828 | -0.00536 | -0.59421 | 109 |  |  |
| -0.59418 | 185.0509 | -0.00533 | -0.5932 | 110 |  |  |
| -0.59311 | 186.119 | -0.0053 | -0.59213 | 111 |  |  |
| -0.59204 | 187.1871 | -0.00527 | -0.59113 | 112 |  |  |
| -0.59097 | 188.2552 | -0.00523 | -0.59003 | 113 |  |  |
| -0.5899 | 189.3234 | -0.0052 | -0.5889 | 114 |  |  |
| -0.58884 | 190.3915 | -0.00517 | -0.58786 | 115 |  |  |
| -0.58777 | 191.4596 | -0.00513 | -0.58682 | 116 |  |  |
| -0.5867 | 192.5277 | -0.00509 | -0.58569 | 117 |  |  |
| -0.58563 | 193.5958 | -0.00506 | -0.58469 | 118 |  |  |
| -0.58456 | 194.6639 | -0.00502 | -0.58359 | 119 |  |  |
| -0.5835 | 195.732 | -0.00498 | -0.58258 | 120 |  |  |
| -0.58243 | 196.8002 | -0.00494 | -0.58154 | 121 |  |  |
| -0.58136 | 197.8683 | -0.00489 | -0.58051 | 122 |  |  |
| -0.58029 | 198.9364 | -0.00484 | -0.57941 | 123 |  |  |
| -0.57922 | 200.0045 | -0.0048 | -0.57834 | 124 |  |  |
| -0.57816 | 201.0726 | -0.00475 | -0.5773 | 125 |  |  |
| -0.57709 | 202.1407 | -0.00469 | -0.57629 | 126 |  |  |
| -0.57602 | 203.2089 | -0.00463 | -0.5752 | 127 |  |  |
| -0.57495 | 204.277 | -0.00457 | -0.57419 | 128 |  |  |
| -0.57388 | 205.3451 | -0.00451 | -0.57315 | 129 |  |  |
| -0.57281 | 206.4132 | -0.00444 | -0.57199 | 130 |  |  |
| -0.57175 | 207.4813 | -0.00437 | -0.57101 | 131 |  |  |
| -0.57068 | 208.5494 | -0.00429 | -0.56995 | 132 |  |  |
| -0.56961 | 209.6175 | -0.00421 | -0.56885 | 133 |  |  |
| -0.56854 | 210.6857 | -0.00413 | -0.56784 | 134 |  |  |
| -0.56747 | 211.7538 | -0.00404 | -0.56683 | 135 |  |  |
| -0.56641 | 212.8219 | -0.00395 | -0.56573 | 136 |  |  |
| -0.56534 | 213.89 | -0.00386 | -0.56473 | 137 |  |  |
| -0.56427 | 214.9581 | -0.00376 | -0.5636 | 138 |  |  |
| -0.5632 | 216.0262 | -0.00367 | -0.56265 | 139 |  |  |
| -0.56213 | 217.0943 | -0.00358 | -0.56155 | 140 |  |  |
| -0.56107 | 218.1625 | -0.00348 | -0.56055 | 141 |  |  |
| -0.56 | 219.2306 | -0.00339 | -0.55945 | 142 |  |  |
| -0.55893 | 220.2987 | -0.0033 | -0.55832 | 143 |  |  |
| -0.55786 | 221.3668 | -0.00321 | -0.55734 | 144 |  |  |
| -0.55679 | 222.4349 | -0.00313 | -0.55627 | 145 |  |  |
| -0.55573 | 223.503 | -0.00304 | -0.55527 | 146 |  |  |
| -0.55466 | 224.5712 | -0.00296 | -0.55426 | 147 |  |  |
| -0.55359 | 225.6393 | -0.00288 | -0.55313 | 148 |  |  |
| -0.55252 | 226.7074 | -0.0028 | -0.55222 | 149 |  |  |
| -0.55145 | 227.7755 | -0.00273 | -0.55109 | 150 |  |  |
| -0.55038 | 228.8436 | -0.00265 | -0.55017 | 151 |  |  |
| -0.54932 | 229.9117 | -0.00258 | -0.54904 | 152 |  |  |
| -0.54825 | 230.9798 | -0.00251 | -0.54794 | 153 |  |  |
| -0.54718 | 232.048 | -0.00244 | -0.54684 | 154 |  |  |
| -0.54611 | 233.1161 | -0.00237 | -0.54587 | 155 |  |  |
| -0.54504 | 234.1842 | -0.00231 | -0.54489 | 156 |  |  |
| -0.54398 | 235.2523 | -0.00224 | -0.54382 | 157 |  |  |
| -0.54291 | 236.3204 | -0.00218 | -0.54269 | 158 |  |  |
| -0.54184 | 237.3885 | -0.00212 | -0.54166 | 159 |  |  |
| -0.54077 | 238.4566 | -0.00206 | -0.54062 | 160 |  |  |
| -0.5397 | 239.5248 | -0.00201 | -0.53955 | 161 |  |  |
| -0.53864 | 240.5929 | -0.00195 | -0.53848 | 162 |  |  |
| -0.53757 | 241.661 | -0.0019 | -0.53735 | 163 |  |  |
| -0.5365 | 242.7291 | -0.00184 | -0.53635 | 164 |  |  |
| -0.53543 | 243.7972 | -0.00179 | -0.53528 | 165 |  |  |
| -0.53436 | 244.8653 | -0.00174 | -0.53424 | 166 |  |  |
| -0.53329 | 245.9335 | -0.00169 | -0.53317 | 167 |  |  |
| -0.53223 | 247.0016 | -0.00165 | -0.53217 | 168 |  |  |
| -0.53116 | 248.0697 | -0.0016 | -0.53113 | 169 |  |  |
| -0.53009 | 249.1378 | -0.00156 | -0.53003 | 170 |  |  |
| -0.52902 | 250.2059 | -0.00151 | -0.52896 | 171 |  |  |
| -0.52795 | 251.274 | -0.00147 | -0.52795 | 172 |  |  |
| -0.52689 | 252.3421 | -0.00143 | -0.52673 | 173 |  |  |
| -0.52582 | 253.4103 | -0.00139 | -0.52573 | 174 |  |  |
| -0.52475 | 254.4784 | -0.00135 | -0.52478 | 175 |  |  |
| -0.52368 | 255.5465 | -0.00131 | -0.52365 | 176 |  |  |
| -0.52261 | 256.6146 | -0.00127 | -0.52255 | 177 |  |  |
| -0.52155 | 257.6827 | -0.00124 | -0.52155 | 178 |  |  |
| -0.52048 | 258.7508 | -0.0012 | -0.52045 | 179 |  |  |
| -0.51941 | 259.8189 | -0.00117 | -0.51935 | 180 |  |  |
| -0.51834 | 260.8871 | -0.00113 | -0.5184 | 181 |  |  |
| -0.51727 | 261.9552 | -0.0011 | -0.51724 | 182 |  |  |
| -0.5162 | 263.0233 | -0.00107 | -0.5162 | 183 |  |  |
| -0.51514 | 264.0914 | -0.00104 | -0.51514 | 184 |  |  |
| -0.51407 | 265.1595 | -0.00101 | -0.51401 | 185 |  |  |
| -0.513 | 266.2276 | -0.00098 | -0.51306 | 186 |  |  |
| -0.51193 | 267.2958 | -0.00095 | -0.5119 | 187 |  |  |
| -0.51086 | 268.3639 | -0.00092 | -0.51093 | 188 |  |  |
| -0.5098 | 269.432 | -0.00089 | -0.50983 | 189 |  |  |
| -0.50873 | 270.5001 | -0.00087 | -0.50873 | 190 |  |  |
| -0.50766 | 271.5682 | -0.00084 | -0.50769 | 191 |  |  |
| -0.50659 | 272.6363 | -0.00082 | -0.50665 | 192 |  |  |
| -0.50552 | 273.7044 | -0.00079 | -0.50558 | 193 |  |  |
| -0.50446 | 274.7726 | -0.00077 | -0.50449 | 194 |  |  |
| -0.50339 | 275.8407 | -0.00074 | -0.50345 | 195 |  |  |
| -0.50232 | 276.9088 | -0.00072 | -0.50241 | 196 |  |  |
| -0.50125 | 277.9769 | -0.0007 | -0.50131 | 197 |  |  |
| -0.50018 | 279.045 | -0.00068 | -0.50015 | 198 |  |  |
| -0.49911 | 280.1131 | -0.00065 | -0.4993 | 199 |  |  |
| -0.49805 | 281.1812 | -0.00063 | -0.49811 | 200 |  |  |
| -0.49698 | 282.2494 | -0.00061 | -0.49704 | 201 |  |  |
| -0.49591 | 283.3175 | -0.00059 | -0.49597 | 202 |  |  |
| -0.49484 | 284.3856 | -0.00057 | -0.4949 | 203 |  |  |
| -0.49377 | 285.4537 | -0.00056 | -0.4938 | 204 |  |  |
| -0.49271 | 286.5218 | -0.00054 | -0.49277 | 205 |  |  |
| -0.49164 | 287.5899 | -0.00052 | -0.49179 | 206 |  |  |
| -0.49057 | 288.6581 | -0.0005 | -0.49069 | 207 |  |  |
| -0.4895 | 289.7262 | -0.00048 | -0.4895 | 208 |  |  |
| -0.48843 | 290.7943 | -0.00047 | -0.48849 | 209 |  |  |
| -0.48737 | 291.8624 | -0.00045 | -0.48743 | 210 |  |  |
| -0.4863 | 292.9305 | -0.00043 | -0.48633 | 211 |  |  |
| -0.48523 | 293.9986 | -0.00042 | -0.48535 | 212 |  |  |
| -0.48416 | 295.0667 | -0.0004 | -0.48425 | 213 |  |  |
| -0.48309 | 296.1349 | -0.00039 | -0.48312 | 214 |  |  |
| -0.48203 | 297.203 | -0.00037 | -0.48209 | 215 |  |  |
| -0.48096 | 298.2711 | -0.00036 | -0.48111 | 216 |  |  |
| -0.47989 | 299.3392 | -0.00034 | -0.47995 | 217 |  |  |
| -0.47882 | 300.4073 | -0.00033 | -0.47888 | 218 |  |  |
| -0.47775 | 301.4754 | -0.00031 | -0.47784 | 219 |  |  |
| -0.47668 | 302.5435 | -0.0003 | -0.47678 | 220 |  |  |
| -0.47562 | 303.6117 | -0.00029 | -0.47565 | 221 |  |  |
| -0.47455 | 304.6798 | -0.00027 | -0.47461 | 222 |  |  |
| -0.47348 | 305.7479 | -0.00026 | -0.47357 | 223 |  |  |
| -0.47241 | 306.816 | -0.00025 | -0.4725 | 224 |  |  |
| -0.47134 | 307.8841 | -0.00023 | -0.4715 | 225 |  |  |
| -0.47028 | 308.9522 | -0.00022 | -0.4704 | 226 |  |  |
| -0.46921 | 310.0204 | -0.00021 | -0.46933 | 227 |  |  |
| -0.46814 | 311.0885 | -0.0002 | -0.46817 | 228 |  |  |
| -0.46707 | 312.1566 | -0.00018 | -0.46719 | 229 |  |  |
| -0.466 | 313.2247 | -0.00017 | -0.46613 | 230 |  |  |
| -0.46494 | 314.2928 | -0.00016 | -0.46503 | 231 |  |  |
| -0.46387 | 315.3609 | -0.00015 | -0.46399 | 232 |  |  |
| -0.4628 | 316.429 | -0.00014 | -0.46292 | 233 |  |  |
| -0.46173 | 317.4972 | -0.00013 | -0.46182 | 234 |  |  |
| -0.46066 | 318.5653 | -0.00011 | -0.46072 | 235 |  |  |
| -0.45959 | 319.6334 | -0.0001 | -0.45969 | 236 |  |  |
| -0.45853 | 320.7015 | -9.1E-05 | -0.45871 | 237 |  |  |
| -0.45746 | 321.7696 | -8E-05 | -0.45755 | 238 |  |  |
| -0.45639 | 322.8377 | -6.9E-05 | -0.45648 | 239 |  |  |
| -0.45532 | 323.9058 | -5.7E-05 | -0.45541 | 240 |  |  |
| -0.45425 | 324.974 | -4.6E-05 | -0.45441 | 241 |  |  |
| -0.45319 | 326.0421 | -3.4E-05 | -0.45322 | 242 |  |  |
| -0.45212 | 327.1102 | -2.3E-05 | -0.45227 | 243 |  |  |
| -0.45105 | 328.1783 | -1.2E-05 | -0.45117 | 244 |  |  |
| -0.44998 | 329.2464 | -1.1E-06 | -0.4502 | 245 |  |  |
| -0.44891 | 330.3145 | 9.86E-06 | -0.44907 | 246 |  |  |
| -0.44785 | 331.3827 | 2.1E-05 | -0.44794 | 247 |  |  |
| -0.44678 | 332.4508 | 3.16E-05 | -0.44702 | 248 |  |  |
| -0.44571 | 333.5189 | 4.41E-05 | -0.44574 | 249 |  |  |
| -0.44464 | 334.587 | 5.58E-05 | -0.44479 | 250 |  |  |
| -0.44357 | 335.6551 | 6.73E-05 | -0.44394 | 251 |  |  |
| -0.4425 | 336.7232 | 7.9E-05 | -0.44263 | 252 |  |  |
| -0.44144 | 337.7913 | 9.07E-05 | -0.44171 | 253 |  |  |
| -0.44037 | 338.8595 | 0.000102 | -0.44077 | 254 |  |  |
| -0.4393 | 339.9276 | 0.000114 | -0.43961 | 255 |  |  |
| -0.43823 | 340.9957 | 0.000126 | -0.43857 | 256 |  |  |
| -0.43716 | 342.0638 | 0.000139 | -0.43747 | 257 |  |  |
| -0.4361 | 343.1319 | 0.000151 | -0.43634 | 258 |  |  |
| -0.43503 | 344.2 | 0.000164 | -0.43533 | 259 |  |  |
| -0.43396 | 345.2681 | 0.000177 | -0.43442 | 260 |  |  |
| -0.43289 | 346.3363 | 0.00019 | -0.43326 | 261 |  |  |
| -0.43182 | 347.4044 | 0.000204 | -0.43225 | 262 |  |  |
| -0.43076 | 348.4725 | 0.000217 | -0.43115 | 263 |  |  |
| -0.42969 | 349.5406 | 0.000231 | -0.43011 | 264 |  |  |
| -0.42862 | 350.6087 | 0.000246 | -0.42905 | 265 |  |  |
| -0.42755 | 351.6768 | 0.00026 | -0.42804 | 266 |  |  |
| -0.42648 | 352.745 | 0.000275 | -0.427 | 267 |  |  |
| -0.42542 | 353.8131 | 0.00029 | -0.42599 | 268 |  |  |
| -0.42435 | 354.8812 | 0.000306 | -0.42499 | 269 |  |  |
| -0.42328 | 355.9493 | 0.000322 | -0.42343 | 270 |  |  |
| -0.42221 | 357.0174 | 0.000338 | -0.42236 | 271 |  |  |
| -0.42114 | 358.0855 | 0.000355 | -0.42133 | 272 |  |  |
| -0.42007 | 359.1536 | 0.000372 | -0.42017 | 273 |  |  |
| -0.41901 | 360.2218 | 0.00039 | -0.41925 | 274 |  |  |
| -0.41794 | 361.2899 | 0.000408 | -0.41809 | 275 |  |  |
| -0.41687 | 362.358 | 0.000427 | -0.41699 | 276 |  |  |
| -0.4158 | 363.4261 | 0.000447 | -0.41599 | 277 |  |  |
| -0.41473 | 364.4942 | 0.000466 | -0.41492 | 278 |  |  |
| -0.41367 | 365.5623 | 0.000487 | -0.41379 | 279 |  |  |
| -0.4126 | 366.6304 | 0.000508 | -0.41278 | 280 |  |  |
| -0.41153 | 367.6986 | 0.00053 | -0.41168 | 281 |  |  |
| -0.41046 | 368.7667 | 0.000553 | -0.41068 | 282 |  |  |
| -0.40939 | 369.8348 | 0.000576 | -0.40958 | 283 |  |  |
| -0.40833 | 370.9029 | 0.000601 | -0.4086 | 284 |  |  |
| -0.40726 | 371.971 | 0.000625 | -0.4075 | 285 |  |  |
| -0.40619 | 373.0391 | 0.000651 | -0.40649 | 286 |  |  |
| -0.40512 | 374.1073 | 0.000679 | -0.40533 | 287 |  |  |
| -0.40405 | 375.1754 | 0.000706 | -0.40433 | 288 |  |  |
| -0.40298 | 376.2435 | 0.000735 | -0.40323 | 289 |  |  |
| -0.40192 | 377.3116 | 0.000765 | -0.40216 | 290 |  |  |
| -0.40085 | 378.3797 | 0.000797 | -0.40109 | 291 |  |  |
| -0.39978 | 379.4478 | 0.000828 | -0.40005 | 292 |  |  |
| -0.39871 | 380.5159 | 0.000862 | -0.3989 | 293 |  |  |
| -0.39764 | 381.5841 | 0.000898 | -0.39783 | 294 |  |  |
| -0.39658 | 382.6522 | 0.000934 | -0.39679 | 295 |  |  |
| -0.39551 | 383.7203 | 0.000972 | -0.39581 | 296 |  |  |
| -0.39444 | 384.7884 | 0.001012 | -0.39468 | 297 |  |  |
| -0.39337 | 385.8565 | 0.001053 | -0.39365 | 298 |  |  |
| -0.3923 | 386.9246 | 0.001097 | -0.39261 | 299 |  |  |
| -0.39124 | 387.9927 | 0.001142 | -0.3916 | 300 |  |  |
| -0.39017 | 389.0609 | 0.001189 | -0.39041 | 301 |  |  |
| -0.3891 | 390.129 | 0.001239 | -0.38947 | 302 |  |  |
| -0.38803 | 391.1971 | 0.001291 | -0.38828 | 303 |  |  |
| -0.38696 | 392.2652 | 0.001345 | -0.38724 | 304 |  |  |
| -0.38589 | 393.3333 | 0.001402 | -0.38626 | 305 |  |  |
| -0.38483 | 394.4014 | 0.001463 | -0.38516 | 306 |  |  |
| -0.38376 | 395.4696 | 0.001526 | -0.38412 | 307 |  |  |
| -0.38269 | 396.5377 | 0.001591 | -0.38293 | 308 |  |  |
| -0.38162 | 397.6058 | 0.00166 | -0.38205 | 309 |  |  |
| -0.38055 | 398.6739 | 0.001732 | -0.38089 | 310 |  |  |
| -0.37949 | 399.742 | 0.001808 | -0.37994 | 311 |  |  |
| -0.37842 | 400.8101 | 0.001886 | -0.37872 | 312 |  |  |
| -0.37735 | 401.8782 | 0.001968 | -0.37769 | 313 |  |  |
| -0.37628 | 402.9464 | 0.002055 | -0.37674 | 314 |  |  |
| -0.37521 | 404.0145 | 0.002146 | -0.37564 | 315 |  |  |
| -0.37415 | 405.0826 | 0.002242 | -0.37463 | 316 |  |  |
| -0.37308 | 406.1507 | 0.002343 | -0.37344 | 317 |  |  |
| -0.37201 | 407.2188 | 0.002448 | -0.37244 | 318 |  |  |
| -0.37094 | 408.2869 | 0.00256 | -0.37137 | 319 |  |  |
| -0.36987 | 409.355 | 0.002677 | -0.37039 | 320 |  |  |
| -0.3688 | 410.4232 | 0.002801 | -0.36932 | 321 |  |  |
| -0.36774 | 411.4913 | 0.00293 | -0.36819 | 322 |  |  |
| -0.36667 | 412.5594 | 0.003066 | -0.36725 | 323 |  |  |
| -0.3656 | 413.6275 | 0.00321 | -0.36633 | 324 |  |  |
| -0.36453 | 414.6956 | 0.003359 | -0.36514 | 325 |  |  |
| -0.36346 | 415.7637 | 0.003515 | -0.36411 | 326 |  |  |
| -0.3624 | 416.8319 | 0.003675 | -0.36316 | 327 |  |  |
| -0.36133 | 417.9 | 0.003837 | -0.36206 | 328 |  |  |
| -0.36026 | 418.9681 | 0.003998 | -0.36096 | 329 |  |  |
| -0.35919 | 420.0362 | 0.004152 | -0.35995 | 330 |  |  |
| -0.35812 | 421.1043 | 0.004297 | -0.35901 | 331 |  |  |
| -0.35706 | 422.1724 | 0.004429 | -0.35788 | 332 |  |  |
| -0.35599 | 423.2405 | 0.00455 | -0.35687 | 333 |  |  |
| -0.35492 | 424.3087 | 0.004659 | -0.35587 | 334 |  |  |
| -0.35385 | 425.3768 | 0.004757 | -0.35477 | 335 |  |  |
| -0.35278 | 426.4449 | 0.004848 | -0.3537 | 336 |  |  |
| -0.35172 | 427.513 | 0.004931 | -0.35269 | 337 |  |  |
| -0.35065 | 428.5811 | 0.005009 | -0.35159 | 338 |  |  |
| -0.34958 | 429.6492 | 0.005082 | -0.35059 | 339 |  |  |
| -0.34851 | 430.7173 | 0.005151 | -0.34958 | 340 |  |  |
| -0.34744 | 431.7855 | 0.005216 | -0.34845 | 341 |  |  |
| -0.34637 | 432.8536 | 0.005279 | -0.34747 | 342 |  |  |
| -0.34531 | 433.9217 | 0.005339 | -0.34637 | 343 |  |  |
| -0.34424 | 434.9898 | 0.005397 | -0.34531 | 344 |  |  |
| -0.34317 | 436.0579 | 0.005453 | -0.34418 | 345 |  |  |
| -0.3421 | 437.126 | 0.005507 | -0.34317 | 346 |  |  |
| -0.34103 | 438.1942 | 0.00556 | -0.34213 | 347 |  |  |
| -0.33997 | 439.2623 | 0.005612 | -0.34113 | 348 |  |  |
| -0.3389 | 440.3304 | 0.005663 | -0.33997 | 349 |  |  |
| -0.33783 | 441.3985 | 0.005713 | -0.33899 | 350 |  |  |
| -0.33676 | 442.4666 | 0.005762 | -0.33798 | 351 |  |  |
| -0.33569 | 443.5347 | 0.00581 | -0.33688 | 352 |  |  |
| -0.33463 | 444.6028 | 0.005857 | -0.33582 | 353 |  |  |
| -0.33356 | 445.671 | 0.005903 | -0.33478 | 354 |  |  |
| -0.33249 | 446.7391 | 0.005949 | -0.33368 | 355 |  |  |
| -0.33142 | 447.8072 | 0.005995 | -0.33261 | 356 |  |  |
| -0.33035 | 448.8753 | 0.00604 | -0.33154 | 357 |  |  |
| -0.32928 | 449.9434 | 0.006085 | -0.33047 | 358 |  |  |
| -0.32822 | 451.0115 | 0.006129 | -0.32938 | 359 |  |  |
| -0.32715 | 452.0796 | 0.006174 | -0.32837 | 360 |  |  |
| -0.32608 | 453.1478 | 0.006217 | -0.32736 | 361 |  |  |
| -0.32501 | 454.2159 | 0.006261 | -0.32629 | 362 |  |  |
| -0.32394 | 455.284 | 0.006304 | -0.32526 | 363 |  |  |
| -0.32288 | 456.3521 | 0.006348 | -0.32422 | 364 |  |  |
| -0.32181 | 457.4202 | 0.00639 | -0.32312 | 365 |  |  |
| -0.32074 | 458.4883 | 0.006433 | -0.32211 | 366 |  |  |
| -0.31967 | 459.5565 | 0.006475 | -0.32092 | 367 |  |  |
| -0.3186 | 460.6246 | 0.006517 | -0.31995 | 368 |  |  |
| -0.31754 | 461.6927 | 0.006559 | -0.31891 | 369 |  |  |
| -0.31647 | 462.7608 | 0.006599 | -0.31784 | 370 |  |  |
| -0.3154 | 463.8289 | 0.00664 | -0.3168 | 371 |  |  |
| -0.31433 | 464.897 | 0.006681 | -0.31577 | 372 |  |  |
| -0.31326 | 465.9651 | 0.006721 | -0.31476 | 373 |  |  |
| -0.31219 | 467.0333 | 0.006761 | -0.31357 | 374 |  |  |
| -0.31113 | 468.1014 | 0.0068 | -0.31256 | 375 |  |  |
| -0.31006 | 469.1695 | 0.006838 | -0.31155 | 376 |  |  |
| -0.30899 | 470.2376 | 0.006876 | -0.31046 | 377 |  |  |
| -0.30792 | 471.3057 | 0.006912 | -0.3093 | 378 |  |  |
| -0.30685 | 472.3738 | 0.006949 | -0.30823 | 379 |  |  |
| -0.30579 | 473.4419 | 0.006984 | -0.30722 | 380 |  |  |
| -0.30472 | 474.5101 | 0.007019 | -0.30621 | 381 |  |  |
| -0.30365 | 475.5782 | 0.007053 | -0.30511 | 382 |  |  |
| -0.30258 | 476.6463 | 0.007086 | -0.30405 | 383 |  |  |
| -0.30151 | 477.7144 | 0.007119 | -0.30304 | 384 |  |  |
| -0.30045 | 478.7825 | 0.00715 | -0.30191 | 385 |  |  |
| -0.29938 | 479.8506 | 0.007181 | -0.30093 | 386 |  |  |
| -0.29831 | 480.9188 | 0.007211 | -0.29974 | 387 |  |  |
| -0.29724 | 481.9869 | 0.007241 | -0.29871 | 388 |  |  |
| -0.29617 | 483.055 | 0.00727 | -0.29767 | 389 |  |  |
| -0.2951 | 484.1231 | 0.007298 | -0.29663 | 390 |  |  |
| -0.29404 | 485.1912 | 0.007326 | -0.29562 | 391 |  |  |
| -0.29297 | 486.2593 | 0.007353 | -0.29459 | 392 |  |  |
| -0.2919 | 487.3274 | 0.00738 | -0.29346 | 393 |  |  |
| -0.29083 | 488.3956 | 0.007406 | -0.29242 | 394 |  |  |
| -0.28976 | 489.4637 | 0.007431 | -0.29129 | 395 |  |  |
| -0.2887 | 490.5318 | 0.007456 | -0.29025 | 396 |  |  |
| -0.28763 | 491.5999 | 0.00748 | -0.28925 | 397 |  |  |
| -0.28656 | 492.668 | 0.007504 | -0.28815 | 398 |  |  |
| -0.28549 | 493.7361 | 0.007527 | -0.28711 | 399 |  |  |
| -0.28442 | 494.8042 | 0.00755 | -0.28598 | 400 |  |  |
| -0.28336 | 495.8724 | 0.007572 | -0.28485 | 401 |  |  |
| -0.28229 | 496.9405 | 0.007594 | -0.28384 | 402 |  |  |
| -0.28122 | 498.0086 | 0.007616 | -0.28278 | 403 |  |  |
| -0.28015 | 499.0767 | 0.007637 | -0.28177 | 404 |  |  |
| -0.27908 | 500.1448 | 0.007658 | -0.28064 | 405 |  |  |
| -0.27802 | 501.2129 | 0.007678 | -0.27966 | 406 |  |  |
| -0.27695 | 502.2811 | 0.007698 | -0.27853 | 407 |  |  |
| -0.27588 | 503.3492 | 0.007717 | -0.27753 | 408 |  |  |
| -0.27481 | 504.4173 | 0.007736 | -0.2764 | 409 |  |  |
| -0.27374 | 505.4854 | 0.007755 | -0.27539 | 410 |  |  |
| -0.27267 | 506.5535 | 0.007773 | -0.27438 | 411 |  |  |
| -0.27161 | 507.6216 | 0.007792 | -0.27319 | 412 |  |  |
| -0.27054 | 508.6897 | 0.00781 | -0.27219 | 413 |  |  |
| -0.26947 | 509.7579 | 0.007827 | -0.27112 | 414 |  |  |
| -0.2684 | 510.826 | 0.007844 | -0.27011 | 415 |  |  |
| -0.26733 | 511.8941 | 0.007861 | -0.26901 | 416 |  |  |
| -0.26627 | 512.9622 | 0.007878 | -0.26794 | 417 |  |  |
| -0.2652 | 514.0303 | 0.007895 | -0.26685 | 418 |  |  |
| -0.26413 | 515.0984 | 0.007911 | -0.26575 | 419 |  |  |
| -0.26306 | 516.1665 | 0.007927 | -0.26477 | 420 |  |  |
| -0.26199 | 517.2347 | 0.007943 | -0.26367 | 421 |  |  |
| -0.26093 | 518.3028 | 0.007958 | -0.26254 | 422 |  |  |
| -0.25986 | 519.3709 | 0.007975 | -0.26163 | 423 |  |  |
| -0.25879 | 520.439 | 0.007992 | -0.26047 | 424 |  |  |
| -0.25772 | 521.5071 | 0.008006 | -0.25928 | 425 |  |  |
| -0.25665 | 522.5752 | 0.008021 | -0.2583 | 426 |  |  |
| -0.25558 | 523.6434 | 0.008037 | -0.25729 | 427 |  |  |
| -0.25452 | 524.7115 | 0.008052 | -0.25626 | 428 |  |  |
| -0.25345 | 525.7796 | 0.008066 | -0.25516 | 429 |  |  |
| -0.25238 | 526.8477 | 0.00808 | -0.25412 | 430 |  |  |
| -0.25131 | 527.9158 | 0.008093 | -0.25302 | 431 |  |  |
| -0.25024 | 528.9839 | 0.008107 | -0.25198 | 432 |  |  |
| -0.24918 | 530.052 | 0.00812 | -0.25085 | 433 |  |  |
| -0.24811 | 531.1202 | 0.008133 | -0.24991 | 434 |  |  |
| -0.24704 | 532.1883 | 0.008146 | -0.24881 | 435 |  |  |
| -0.24597 | 533.2564 | 0.00816 | -0.24777 | 436 |  |  |
| -0.2449 | 534.3245 | 0.008172 | -0.24664 | 437 |  |  |
| -0.24384 | 535.3926 | 0.008184 | -0.24561 | 438 |  |  |
| -0.24277 | 536.4607 | 0.008196 | -0.24457 | 439 |  |  |
| -0.2417 | 537.5288 | 0.008211 | -0.24347 | 440 |  |  |
| -0.24063 | 538.597 | 0.008224 | -0.24249 | 441 |  |  |
| -0.23956 | 539.6651 | 0.008234 | -0.2413 | 442 |  |  |
| -0.23849 | 540.7332 | 0.008246 | -0.24036 | 443 |  |  |
| -0.23743 | 541.8013 | 0.008257 | -0.23923 | 444 |  |  |
| -0.23636 | 542.8694 | 0.008268 | -0.23822 | 445 |  |  |
| -0.23529 | 543.9375 | 0.008279 | -0.23709 | 446 |  |  |
| -0.23422 | 545.0057 | 0.008289 | -0.23605 | 447 |  |  |
| -0.23315 | 546.0738 | 0.0083 | -0.23492 | 448 |  |  |
| -0.23209 | 547.1419 | 0.00831 | -0.23383 | 449 |  |  |
| -0.23102 | 548.21 | 0.008321 | -0.23288 | 450 |  |  |
| -0.22995 | 549.2781 | 0.008333 | -0.23184 | 451 |  |  |
| -0.22888 | 550.3462 | 0.008343 | -0.23071 | 452 |  |  |
| -0.22781 | 551.4143 | 0.008354 | -0.22968 | 453 |  |  |
| -0.22675 | 552.4825 | 0.008364 | -0.22861 | 454 |  |  |
| -0.22568 | 553.5506 | 0.008373 | -0.22748 | 455 |  |  |
| -0.22461 | 554.6187 | 0.008382 | -0.22641 | 456 |  |  |
| -0.22354 | 555.6868 | 0.008392 | -0.22537 | 457 |  |  |
| -0.22247 | 556.7549 | 0.008401 | -0.22433 | 458 |  |  |
| -0.22141 | 557.823 | 0.00841 | -0.22324 | 459 |  |  |
| -0.22034 | 558.8911 | 0.008416 | -0.22223 | 460 |  |  |
| -0.21927 | 559.9593 | 0.008425 | -0.2211 | 461 |  |  |
| -0.2182 | 561.0274 | 0.008433 | -0.22012 | 462 |  |  |
| -0.21713 | 562.0955 | 0.008442 | -0.21893 | 463 |  |  |
| -0.21606 | 563.1636 | 0.008449 | -0.21796 | 464 |  |  |
| -0.215 | 564.2317 | 0.008458 | -0.21683 | 465 |  |  |
| -0.21393 | 565.2998 | 0.008467 | -0.21585 | 466 |  |  |
| -0.21286 | 566.368 | 0.008476 | -0.21472 | 467 |  |  |
| -0.21179 | 567.4361 | 0.008485 | -0.21362 | 468 |  |  |
| -0.21072 | 568.5042 | 0.008492 | -0.21252 | 469 |  |  |

Sample: *Maurorum*, Concentration (ppm): 800, Immersion time: 24h

| Potential applied(V) | Time (s) | WE(1).  Current (A) | WE(1).  Potential (V) | Index |  |  |
| --- | --- | --- | --- | --- | --- | --- |
| -0.71045 | 68.71137 | -0.00093 | -0.70828 | 1 |  |  |
| -0.70938 | 69.77949 | -0.00766 | -0.7077 | 2 |  |  |
| -0.70831 | 70.8476 | -0.00763 | -0.70673 | 3 |  |  |
| -0.70724 | 71.91572 | -0.00759 | -0.7056 | 4 |  |  |
| -0.70618 | 72.98383 | -0.00757 | -0.70456 | 5 |  |  |
| -0.70511 | 74.05195 | -0.00754 | -0.70352 | 6 |  |  |
| -0.70404 | 75.12006 | -0.00751 | -0.70251 | 7 |  |  |
| -0.70297 | 76.18818 | -0.00749 | -0.70135 | 8 |  |  |
| -0.7019 | 77.25629 | -0.00746 | -0.70032 | 9 |  |  |
| -0.70084 | 78.32441 | -0.00744 | -0.6994 | 10 |  |  |
| -0.69977 | 79.39252 | -0.00741 | -0.69821 | 11 |  |  |
| -0.6987 | 80.46064 | -0.00739 | -0.69714 | 12 |  |  |
| -0.69763 | 81.52875 | -0.00737 | -0.69614 | 13 |  |  |
| -0.69656 | 82.59687 | -0.00734 | -0.69513 | 14 |  |  |
| -0.6955 | 83.66498 | -0.00732 | -0.69388 | 15 |  |  |
| -0.69443 | 84.7331 | -0.0073 | -0.69284 | 16 |  |  |
| -0.69336 | 85.80121 | -0.00728 | -0.69189 | 17 |  |  |
| -0.69229 | 86.86933 | -0.00725 | -0.69083 | 18 |  |  |
| -0.69122 | 87.93744 | -0.00723 | -0.68967 | 19 |  |  |
| -0.69016 | 89.00556 | -0.00721 | -0.68857 | 20 |  |  |
| -0.68909 | 90.07367 | -0.00719 | -0.68762 | 21 |  |  |
| -0.68802 | 91.14179 | -0.00717 | -0.68655 | 22 |  |  |
| -0.68695 | 92.2099 | -0.00715 | -0.68546 | 23 |  |  |
| -0.68588 | 93.27802 | -0.00712 | -0.68439 | 24 |  |  |
| -0.68481 | 94.34613 | -0.0071 | -0.68329 | 25 |  |  |
| -0.68375 | 95.41425 | -0.00708 | -0.68225 | 26 |  |  |
| -0.68268 | 96.48236 | -0.00706 | -0.68118 | 27 |  |  |
| -0.68161 | 97.55048 | -0.00704 | -0.68011 | 28 |  |  |
| -0.68054 | 98.61859 | -0.00702 | -0.67914 | 29 |  |  |
| -0.67947 | 99.68671 | -0.007 | -0.6781 | 30 |  |  |
| -0.67841 | 100.7548 | -0.00698 | -0.67685 | 31 |  |  |
| -0.67734 | 101.8229 | -0.00696 | -0.67587 | 32 |  |  |
| -0.67627 | 102.8911 | -0.00694 | -0.67493 | 33 |  |  |
| -0.6752 | 103.9592 | -0.00692 | -0.67374 | 34 |  |  |
| -0.67413 | 105.0273 | -0.0069 | -0.6727 | 35 |  |  |
| -0.67307 | 106.0954 | -0.00688 | -0.67169 | 36 |  |  |
| -0.672 | 107.1635 | -0.00686 | -0.67056 | 37 |  |  |
| -0.67093 | 108.2316 | -0.00684 | -0.66956 | 38 |  |  |
| -0.66986 | 109.2997 | -0.00682 | -0.66843 | 39 |  |  |
| -0.66879 | 110.3679 | -0.0068 | -0.66739 | 40 |  |  |
| -0.66772 | 111.436 | -0.00678 | -0.66638 | 41 |  |  |
| -0.66666 | 112.5041 | -0.00676 | -0.66516 | 42 |  |  |
| -0.66559 | 113.5722 | -0.00674 | -0.66418 | 43 |  |  |
| -0.66452 | 114.6403 | -0.00672 | -0.66321 | 44 |  |  |
| -0.66345 | 115.7084 | -0.0067 | -0.66208 | 45 |  |  |
| -0.66238 | 116.7765 | -0.00668 | -0.66113 | 46 |  |  |
| -0.66132 | 117.8447 | -0.00666 | -0.65997 | 47 |  |  |
| -0.66025 | 118.9128 | -0.00664 | -0.65884 | 48 |  |  |
| -0.65918 | 119.9809 | -0.00662 | -0.65787 | 49 |  |  |
| -0.65811 | 121.049 | -0.0066 | -0.65686 | 50 |  |  |
| -0.65704 | 122.1171 | -0.00658 | -0.65573 | 51 |  |  |
| -0.65598 | 123.1852 | -0.00656 | -0.65466 | 52 |  |  |
| -0.65491 | 124.2534 | -0.00654 | -0.65356 | 53 |  |  |
| -0.65384 | 125.3215 | -0.00652 | -0.6524 | 54 |  |  |
| -0.65277 | 126.3896 | -0.0065 | -0.65146 | 55 |  |  |
| -0.6517 | 127.4577 | -0.00648 | -0.65039 | 56 |  |  |
| -0.65063 | 128.5258 | -0.00646 | -0.64932 | 57 |  |  |
| -0.64957 | 129.5939 | -0.00644 | -0.64819 | 58 |  |  |
| -0.6485 | 130.662 | -0.00642 | -0.64709 | 59 |  |  |
| -0.64743 | 131.7302 | -0.0064 | -0.64624 | 60 |  |  |
| -0.64636 | 132.7983 | -0.00638 | -0.64505 | 61 |  |  |
| -0.64529 | 133.8664 | -0.00636 | -0.6441 | 62 |  |  |
| -0.64423 | 134.9345 | -0.00634 | -0.64294 | 63 |  |  |
| -0.64316 | 136.0026 | -0.00632 | -0.642 | 64 |  |  |
| -0.64209 | 137.0707 | -0.0063 | -0.64084 | 65 |  |  |
| -0.64102 | 138.1388 | -0.00628 | -0.6398 | 66 |  |  |
| -0.63995 | 139.207 | -0.00625 | -0.63876 | 67 |  |  |
| -0.63889 | 140.2751 | -0.00623 | -0.63766 | 68 |  |  |
| -0.63782 | 141.3432 | -0.00621 | -0.63669 | 69 |  |  |
| -0.63675 | 142.4113 | -0.00619 | -0.6355 | 70 |  |  |
| -0.63568 | 143.4794 | -0.00617 | -0.63452 | 71 |  |  |
| -0.63461 | 144.5475 | -0.00615 | -0.63348 | 72 |  |  |
| -0.63354 | 145.6157 | -0.00613 | -0.63242 | 73 |  |  |
| -0.63248 | 146.6838 | -0.00611 | -0.63132 | 74 |  |  |
| -0.63141 | 147.7519 | -0.00608 | -0.63031 | 75 |  |  |
| -0.63034 | 148.82 | -0.00606 | -0.62921 | 76 |  |  |
| -0.62927 | 149.8881 | -0.00604 | -0.6282 | 77 |  |  |
| -0.6282 | 150.9562 | -0.00602 | -0.62708 | 78 |  |  |
| -0.62714 | 152.0243 | -0.006 | -0.62589 | 79 |  |  |
| -0.62607 | 153.0925 | -0.00597 | -0.62491 | 80 |  |  |
| -0.625 | 154.1606 | -0.00595 | -0.6239 | 81 |  |  |
| -0.62393 | 155.2287 | -0.00593 | -0.62292 | 82 |  |  |
| -0.62286 | 156.2968 | -0.00591 | -0.6217 | 83 |  |  |
| -0.6218 | 157.3649 | -0.00588 | -0.62064 | 84 |  |  |
| -0.62073 | 158.433 | -0.00586 | -0.61966 | 85 |  |  |
| -0.61966 | 159.5011 | -0.00584 | -0.61859 | 86 |  |  |
| -0.61859 | 160.5693 | -0.00581 | -0.61755 | 87 |  |  |
| -0.61752 | 161.6374 | -0.00579 | -0.61649 | 88 |  |  |
| -0.61646 | 162.7055 | -0.00576 | -0.61545 | 89 |  |  |
| -0.61539 | 163.7736 | -0.00574 | -0.61432 | 90 |  |  |
| -0.61432 | 164.8417 | -0.00572 | -0.61325 | 91 |  |  |
| -0.61325 | 165.9098 | -0.00569 | -0.61224 | 92 |  |  |
| -0.61218 | 166.978 | -0.00566 | -0.61111 | 93 |  |  |
| -0.61111 | 168.0461 | -0.00564 | -0.61002 | 94 |  |  |
| -0.61005 | 169.1142 | -0.00561 | -0.60904 | 95 |  |  |
| -0.60898 | 170.1823 | -0.00559 | -0.60788 | 96 |  |  |
| -0.60791 | 171.2504 | -0.00556 | -0.6069 | 97 |  |  |
| -0.60684 | 172.3185 | -0.00553 | -0.60583 | 98 |  |  |
| -0.60577 | 173.3866 | -0.0055 | -0.60471 | 99 |  |  |
| -0.60471 | 174.4548 | -0.00548 | -0.60379 | 100 |  |  |
| -0.60364 | 175.5229 | -0.00545 | -0.60257 | 101 |  |  |
| -0.60257 | 176.591 | -0.00542 | -0.6015 | 102 |  |  |
| -0.6015 | 177.6591 | -0.00539 | -0.60059 | 103 |  |  |
| -0.60043 | 178.7272 | -0.00536 | -0.59952 | 104 |  |  |
| -0.59937 | 179.7953 | -0.00533 | -0.59845 | 105 |  |  |
| -0.5983 | 180.8634 | -0.00529 | -0.59726 | 106 |  |  |
| -0.59723 | 181.9316 | -0.00526 | -0.59628 | 107 |  |  |
| -0.59616 | 182.9997 | -0.00523 | -0.59525 | 108 |  |  |
| -0.59509 | 184.0678 | -0.00519 | -0.59406 | 109 |  |  |
| -0.59402 | 185.1359 | -0.00516 | -0.59308 | 110 |  |  |
| -0.59296 | 186.204 | -0.00512 | -0.59204 | 111 |  |  |
| -0.59189 | 187.2721 | -0.00508 | -0.59091 | 112 |  |  |
| -0.59082 | 188.3403 | -0.00504 | -0.59003 | 113 |  |  |
| -0.58975 | 189.4084 | -0.005 | -0.58884 | 114 |  |  |
| -0.58868 | 190.4765 | -0.00496 | -0.58786 | 115 |  |  |
| -0.58762 | 191.5446 | -0.00491 | -0.58673 | 116 |  |  |
| -0.58655 | 192.6127 | -0.00486 | -0.58569 | 117 |  |  |
| -0.58548 | 193.6808 | -0.00481 | -0.58463 | 118 |  |  |
| -0.58441 | 194.7489 | -0.00476 | -0.58362 | 119 |  |  |
| -0.58334 | 195.8171 | -0.00471 | -0.58246 | 120 |  |  |
| -0.58228 | 196.8852 | -0.00465 | -0.58145 | 121 |  |  |
| -0.58121 | 197.9533 | -0.00459 | -0.58032 | 122 |  |  |
| -0.58014 | 199.0214 | -0.00452 | -0.57932 | 123 |  |  |
| -0.57907 | 200.0895 | -0.00445 | -0.57825 | 124 |  |  |
| -0.578 | 201.1576 | -0.00438 | -0.57733 | 125 |  |  |
| -0.57693 | 202.2257 | -0.0043 | -0.57623 | 126 |  |  |
| -0.57587 | 203.2939 | -0.00421 | -0.57516 | 127 |  |  |
| -0.5748 | 204.362 | -0.00413 | -0.57416 | 128 |  |  |
| -0.57373 | 205.4301 | -0.00404 | -0.57306 | 129 |  |  |
| -0.57266 | 206.4982 | -0.00394 | -0.57187 | 130 |  |  |
| -0.57159 | 207.5663 | -0.00385 | -0.57104 | 131 |  |  |
| -0.57053 | 208.6344 | -0.00375 | -0.56989 | 132 |  |  |
| -0.56946 | 209.7026 | -0.00366 | -0.56888 | 133 |  |  |
| -0.56839 | 210.7707 | -0.00356 | -0.56784 | 134 |  |  |
| -0.56732 | 211.8388 | -0.00347 | -0.56683 | 135 |  |  |
| -0.56625 | 212.9069 | -0.00337 | -0.56564 | 136 |  |  |
| -0.56519 | 213.975 | -0.00328 | -0.56473 | 137 |  |  |
| -0.56412 | 215.0431 | -0.0032 | -0.56363 | 138 |  |  |
| -0.56305 | 216.1112 | -0.00311 | -0.56265 | 139 |  |  |
| -0.56198 | 217.1794 | -0.00302 | -0.56152 | 140 |  |  |
| -0.56091 | 218.2475 | -0.00294 | -0.56055 | 141 |  |  |
| -0.55984 | 219.3156 | -0.00286 | -0.55948 | 142 |  |  |
| -0.55878 | 220.3837 | -0.00278 | -0.55841 | 143 |  |  |
| -0.55771 | 221.4518 | -0.00271 | -0.55728 | 144 |  |  |
| -0.55664 | 222.5199 | -0.00263 | -0.5564 | 145 |  |  |
| -0.55557 | 223.588 | -0.00256 | -0.55524 | 146 |  |  |
| -0.5545 | 224.6562 | -0.00249 | -0.55426 | 147 |  |  |
| -0.55344 | 225.7243 | -0.00242 | -0.55325 | 148 |  |  |
| -0.55237 | 226.7924 | -0.00236 | -0.55219 | 149 |  |  |
| -0.5513 | 227.8605 | -0.00229 | -0.55099 | 150 |  |  |
| -0.55023 | 228.9286 | -0.00223 | -0.55008 | 151 |  |  |
| -0.54916 | 229.9967 | -0.00217 | -0.54895 | 152 |  |  |
| -0.5481 | 231.0649 | -0.00211 | -0.54788 | 153 |  |  |
| -0.54703 | 232.133 | -0.00205 | -0.54694 | 154 |  |  |
| -0.54596 | 233.2011 | -0.00199 | -0.54578 | 155 |  |  |
| -0.54489 | 234.2692 | -0.00194 | -0.5448 | 156 |  |  |
| -0.54382 | 235.3373 | -0.00189 | -0.5437 | 157 |  |  |
| -0.54276 | 236.4054 | -0.00183 | -0.54276 | 158 |  |  |
| -0.54169 | 237.4735 | -0.00178 | -0.54153 | 159 |  |  |
| -0.54062 | 238.5417 | -0.00173 | -0.54047 | 160 |  |  |
| -0.53955 | 239.6098 | -0.00169 | -0.53946 | 161 |  |  |
| -0.53848 | 240.6779 | -0.00164 | -0.53833 | 162 |  |  |
| -0.53741 | 241.746 | -0.0016 | -0.53732 | 163 |  |  |
| -0.53635 | 242.8141 | -0.00155 | -0.53625 | 164 |  |  |
| -0.53528 | 243.8822 | -0.00151 | -0.53528 | 165 |  |  |
| -0.53421 | 244.9503 | -0.00147 | -0.53418 | 166 |  |  |
| -0.53314 | 246.0185 | -0.00143 | -0.53311 | 167 |  |  |
| -0.53207 | 247.0866 | -0.00139 | -0.53198 | 168 |  |  |
| -0.53101 | 248.1547 | -0.00135 | -0.53107 | 169 |  |  |
| -0.52994 | 249.2228 | -0.00131 | -0.52997 | 170 |  |  |
| -0.52887 | 250.2909 | -0.00128 | -0.52881 | 171 |  |  |
| -0.5278 | 251.359 | -0.00124 | -0.52783 | 172 |  |  |
| -0.52673 | 252.4272 | -0.00121 | -0.5267 | 173 |  |  |
| -0.52567 | 253.4953 | -0.00117 | -0.52563 | 174 |  |  |
| -0.5246 | 254.5634 | -0.00114 | -0.5246 | 175 |  |  |
| -0.52353 | 255.6315 | -0.00111 | -0.52347 | 176 |  |  |
| -0.52246 | 256.6996 | -0.00108 | -0.52249 | 177 |  |  |
| -0.52139 | 257.7677 | -0.00105 | -0.52142 | 178 |  |  |
| -0.52032 | 258.8358 | -0.00102 | -0.52039 | 179 |  |  |
| -0.51926 | 259.904 | -0.00099 | -0.51926 | 180 |  |  |
| -0.51819 | 260.9721 | -0.00096 | -0.51822 | 181 |  |  |
| -0.51712 | 262.0402 | -0.00094 | -0.51718 | 182 |  |  |
| -0.51605 | 263.1083 | -0.00091 | -0.51608 | 183 |  |  |
| -0.51498 | 264.1764 | -0.00088 | -0.51511 | 184 |  |  |
| -0.51392 | 265.2445 | -0.00086 | -0.51398 | 185 |  |  |
| -0.51285 | 266.3126 | -0.00084 | -0.51285 | 186 |  |  |
| -0.51178 | 267.3808 | -0.00081 | -0.51187 | 187 |  |  |
| -0.51071 | 268.4489 | -0.00079 | -0.51071 | 188 |  |  |
| -0.50964 | 269.517 | -0.00077 | -0.50983 | 189 |  |  |
| -0.50858 | 270.5851 | -0.00075 | -0.50861 | 190 |  |  |
| -0.50751 | 271.6532 | -0.00072 | -0.50748 | 191 |  |  |
| -0.50644 | 272.7213 | -0.0007 | -0.50641 | 192 |  |  |
| -0.50537 | 273.7895 | -0.00068 | -0.50543 | 193 |  |  |
| -0.5043 | 274.8576 | -0.00066 | -0.50439 | 194 |  |  |
| -0.50323 | 275.9257 | -0.00065 | -0.5033 | 195 |  |  |
| -0.50217 | 276.9938 | -0.00063 | -0.50232 | 196 |  |  |
| -0.5011 | 278.0619 | -0.00061 | -0.50119 | 197 |  |  |
| -0.50003 | 279.13 | -0.00059 | -0.5 | 198 |  |  |
| -0.49896 | 280.1981 | -0.00057 | -0.49911 | 199 |  |  |
| -0.49789 | 281.2663 | -0.00056 | -0.49802 | 200 |  |  |
| -0.49683 | 282.3344 | -0.00054 | -0.49698 | 201 |  |  |
| -0.49576 | 283.4025 | -0.00052 | -0.49588 | 202 |  |  |
| -0.49469 | 284.4706 | -0.00051 | -0.49487 | 203 |  |  |
| -0.49362 | 285.5387 | -0.00049 | -0.49374 | 204 |  |  |
| -0.49255 | 286.6068 | -0.00048 | -0.49265 | 205 |  |  |
| -0.49149 | 287.6749 | -0.00046 | -0.49155 | 206 |  |  |
| -0.49042 | 288.7431 | -0.00045 | -0.49048 | 207 |  |  |
| -0.48935 | 289.8112 | -0.00044 | -0.48944 | 208 |  |  |
| -0.48828 | 290.8793 | -0.00042 | -0.48822 | 209 |  |  |
| -0.48721 | 291.9474 | -0.00041 | -0.4873 | 210 |  |  |
| -0.48615 | 293.0155 | -0.0004 | -0.48618 | 211 |  |  |
| -0.48508 | 294.0836 | -0.00038 | -0.4852 | 212 |  |  |
| -0.48401 | 295.1518 | -0.00037 | -0.4841 | 213 |  |  |
| -0.48294 | 296.2199 | -0.00036 | -0.48297 | 214 |  |  |
| -0.48187 | 297.288 | -0.00035 | -0.48196 | 215 |  |  |
| -0.4808 | 298.3561 | -0.00034 | -0.48087 | 216 |  |  |
| -0.47974 | 299.4242 | -0.00032 | -0.47974 | 217 |  |  |
| -0.47867 | 300.4923 | -0.00031 | -0.47879 | 218 |  |  |
| -0.4776 | 301.5604 | -0.0003 | -0.47766 | 219 |  |  |
| -0.47653 | 302.6286 | -0.00029 | -0.47662 | 220 |  |  |
| -0.47546 | 303.6967 | -0.00028 | -0.47565 | 221 |  |  |
| -0.4744 | 304.7648 | -0.00027 | -0.4744 | 222 |  |  |
| -0.47333 | 305.8329 | -0.00026 | -0.47336 | 223 |  |  |
| -0.47226 | 306.901 | -0.00025 | -0.47238 | 224 |  |  |
| -0.47119 | 307.9691 | -0.00024 | -0.47131 | 225 |  |  |
| -0.47012 | 309.0372 | -0.00023 | -0.47021 | 226 |  |  |
| -0.46906 | 310.1054 | -0.00022 | -0.46918 | 227 |  |  |
| -0.46799 | 311.1735 | -0.00021 | -0.46805 | 228 |  |  |
| -0.46692 | 312.2416 | -0.0002 | -0.46701 | 229 |  |  |
| -0.46585 | 313.3097 | -0.00019 | -0.466 | 230 |  |  |
| -0.46478 | 314.3778 | -0.00018 | -0.46494 | 231 |  |  |
| -0.46371 | 315.4459 | -0.00017 | -0.46387 | 232 |  |  |
| -0.46265 | 316.5141 | -0.00017 | -0.46271 | 233 |  |  |
| -0.46158 | 317.5822 | -0.00016 | -0.46173 | 234 |  |  |
| -0.46051 | 318.6503 | -0.00015 | -0.46066 | 235 |  |  |
| -0.45944 | 319.7184 | -0.00014 | -0.45953 | 236 |  |  |
| -0.45837 | 320.7865 | -0.00013 | -0.4585 | 237 |  |  |
| -0.45731 | 321.8546 | -0.00012 | -0.45746 | 238 |  |  |
| -0.45624 | 322.9227 | -0.00011 | -0.45636 | 239 |  |  |
| -0.45517 | 323.9909 | -0.00011 | -0.45517 | 240 |  |  |
| -0.4541 | 325.059 | -9.7E-05 | -0.45416 | 241 |  |  |
| -0.45303 | 326.1271 | -8.8E-05 | -0.45316 | 242 |  |  |
| -0.45197 | 327.1952 | -8E-05 | -0.45215 | 243 |  |  |
| -0.4509 | 328.2633 | -7.1E-05 | -0.45102 | 244 |  |  |
| -0.44983 | 329.3314 | -6.3E-05 | -0.44989 | 245 |  |  |
| -0.44876 | 330.3995 | -5.4E-05 | -0.44873 | 246 |  |  |
| -0.44769 | 331.4677 | -4.6E-05 | -0.44778 | 247 |  |  |
| -0.44662 | 332.5358 | -3.7E-05 | -0.44672 | 248 |  |  |
| -0.44556 | 333.6039 | -2.8E-05 | -0.44556 | 249 |  |  |
| -0.44449 | 334.672 | -2E-05 | -0.44455 | 250 |  |  |
| -0.44342 | 335.7401 | -1.2E-05 | -0.44351 | 251 |  |  |
| -0.44235 | 336.8082 | -3.4E-06 | -0.44241 | 252 |  |  |
| -0.44128 | 337.8764 | 4.88E-06 | -0.44141 | 253 |  |  |
| -0.44022 | 338.9445 | 1.35E-05 | -0.44049 | 254 |  |  |
| -0.43915 | 340.0126 | 2.22E-05 | -0.43924 | 255 |  |  |
| -0.43808 | 341.0807 | 3.07E-05 | -0.4382 | 256 |  |  |
| -0.43701 | 342.1488 | 3.96E-05 | -0.43729 | 257 |  |  |
| -0.43594 | 343.2169 | 4.95E-05 | -0.43619 | 258 |  |  |
| -0.43488 | 344.285 | 5.86E-05 | -0.43506 | 259 |  |  |
| -0.43381 | 345.3532 | 6.8E-05 | -0.43417 | 260 |  |  |
| -0.43274 | 346.4213 | 7.76E-05 | -0.43307 | 261 |  |  |
| -0.43167 | 347.4894 | 8.72E-05 | -0.43201 | 262 |  |  |
| -0.4306 | 348.5575 | 9.75E-05 | -0.43091 | 263 |  |  |
| -0.42953 | 349.6256 | 0.000107 | -0.42984 | 264 |  |  |
| -0.42847 | 350.6937 | 0.000117 | -0.42886 | 265 |  |  |
| -0.4274 | 351.7618 | 0.000128 | -0.4278 | 266 |  |  |
| -0.42633 | 352.83 | 0.000139 | -0.4267 | 267 |  |  |
| -0.42526 | 353.8981 | 0.00015 | -0.42557 | 268 |  |  |
| -0.42419 | 354.9662 | 0.000161 | -0.42456 | 269 |  |  |
| -0.42313 | 356.0343 | 0.000172 | -0.42352 | 270 |  |  |
| -0.42206 | 357.1024 | 0.000184 | -0.42252 | 271 |  |  |
| -0.42099 | 358.1705 | 0.000196 | -0.42154 | 272 |  |  |
| -0.41992 | 359.2387 | 0.000208 | -0.42035 | 273 |  |  |
| -0.41885 | 360.3068 | 0.000221 | -0.41922 | 274 |  |  |
| -0.41779 | 361.3749 | 0.000233 | -0.41833 | 275 |  |  |
| -0.41672 | 362.443 | 0.000247 | -0.41714 | 276 |  |  |
| -0.41565 | 363.5111 | 0.00026 | -0.41614 | 277 |  |  |
| -0.41458 | 364.5792 | 0.000274 | -0.4151 | 278 |  |  |
| -0.41351 | 365.6473 | 0.000289 | -0.41397 | 279 |  |  |
| -0.41245 | 366.7155 | 0.000304 | -0.41296 | 280 |  |  |
| -0.41138 | 367.7836 | 0.000319 | -0.41168 | 281 |  |  |
| -0.41031 | 368.8517 | 0.000335 | -0.41049 | 282 |  |  |
| -0.40924 | 369.9198 | 0.000352 | -0.40939 | 283 |  |  |
| -0.40817 | 370.9879 | 0.000368 | -0.40833 | 284 |  |  |
| -0.4071 | 372.056 | 0.000386 | -0.40726 | 285 |  |  |
| -0.40604 | 373.1241 | 0.000404 | -0.40622 | 286 |  |  |
| -0.40497 | 374.1923 | 0.000423 | -0.40509 | 287 |  |  |
| -0.4039 | 375.2604 | 0.000443 | -0.40405 | 288 |  |  |
| -0.40283 | 376.3285 | 0.000463 | -0.40295 | 289 |  |  |
| -0.40176 | 377.3966 | 0.000484 | -0.40198 | 290 |  |  |
| -0.4007 | 378.4647 | 0.000506 | -0.40097 | 291 |  |  |
| -0.39963 | 379.5328 | 0.000529 | -0.3999 | 292 |  |  |
| -0.39856 | 380.601 | 0.000553 | -0.39886 | 293 |  |  |
| -0.39749 | 381.6691 | 0.000578 | -0.39774 | 294 |  |  |
| -0.39642 | 382.7372 | 0.000604 | -0.3967 | 295 |  |  |
| -0.39536 | 383.8053 | 0.00063 | -0.39554 | 296 |  |  |
| -0.39429 | 384.8734 | 0.000658 | -0.39462 | 297 |  |  |
| -0.39322 | 385.9415 | 0.000687 | -0.39349 | 298 |  |  |
| -0.39215 | 387.0096 | 0.000717 | -0.3924 | 299 |  |  |
| -0.39108 | 388.0778 | 0.000748 | -0.39133 | 300 |  |  |
| -0.39001 | 389.1459 | 0.000779 | -0.39032 | 301 |  |  |
| -0.38895 | 390.214 | 0.000813 | -0.38928 | 302 |  |  |
| -0.38788 | 391.2821 | 0.000848 | -0.38815 | 303 |  |  |
| -0.38681 | 392.3502 | 0.000885 | -0.38708 | 304 |  |  |
| -0.38574 | 393.4183 | 0.000924 | -0.38599 | 305 |  |  |
| -0.38467 | 394.4864 | 0.000965 | -0.38498 | 306 |  |  |
| -0.38361 | 395.5546 | 0.001007 | -0.38388 | 307 |  |  |
| -0.38254 | 396.6227 | 0.001052 | -0.38278 | 308 |  |  |
| -0.38147 | 397.6908 | 0.001098 | -0.38184 | 309 |  |  |
| -0.3804 | 398.7589 | 0.001146 | -0.38077 | 310 |  |  |
| -0.37933 | 399.827 | 0.001197 | -0.37958 | 311 |  |  |
| -0.37827 | 400.8951 | 0.001251 | -0.37854 | 312 |  |  |
| -0.3772 | 401.9632 | 0.001307 | -0.37762 | 313 |  |  |
| -0.37613 | 403.0314 | 0.001367 | -0.3764 | 314 |  |  |
| -0.37506 | 404.0995 | 0.001428 | -0.37537 | 315 |  |  |
| -0.37399 | 405.1676 | 0.001494 | -0.37427 | 316 |  |  |
| -0.37292 | 406.2357 | 0.001563 | -0.37326 | 317 |  |  |
| -0.37186 | 407.3038 | 0.001635 | -0.37213 | 318 |  |  |
| -0.37079 | 408.3719 | 0.001711 | -0.37119 | 319 |  |  |
| -0.36972 | 409.4401 | 0.001792 | -0.37 | 320 |  |  |
| -0.36865 | 410.5082 | 0.001875 | -0.36899 | 321 |  |  |
| -0.36758 | 411.5763 | 0.001964 | -0.36792 | 322 |  |  |
| -0.36652 | 412.6444 | 0.002058 | -0.36697 | 323 |  |  |
| -0.36545 | 413.7125 | 0.002157 | -0.36581 | 324 |  |  |
| -0.36438 | 414.7806 | 0.002261 | -0.36472 | 325 |  |  |
| -0.36331 | 415.8487 | 0.00237 | -0.36371 | 326 |  |  |
| -0.36224 | 416.9169 | 0.002486 | -0.36255 | 327 |  |  |
| -0.36118 | 417.985 | 0.002608 | -0.36157 | 328 |  |  |
| -0.36011 | 419.0531 | 0.002738 | -0.36066 | 329 |  |  |
| -0.35904 | 420.1212 | 0.002874 | -0.35953 | 330 |  |  |
| -0.35797 | 421.1893 | 0.003018 | -0.35858 | 331 |  |  |
| -0.3569 | 422.2574 | 0.00317 | -0.35748 | 332 |  |  |
| -0.35583 | 423.3255 | 0.00333 | -0.35641 | 333 |  |  |
| -0.35477 | 424.3937 | 0.003499 | -0.35544 | 334 |  |  |
| -0.3537 | 425.4618 | 0.003672 | -0.35446 | 335 |  |  |
| -0.35263 | 426.5299 | 0.003848 | -0.35339 | 336 |  |  |
| -0.35156 | 427.598 | 0.004023 | -0.35239 | 337 |  |  |
| -0.35049 | 428.6661 | 0.00419 | -0.35132 | 338 |  |  |
| -0.34943 | 429.7342 | 0.004347 | -0.35031 | 339 |  |  |
| -0.34836 | 430.8024 | 0.004488 | -0.34924 | 340 |  |  |
| -0.34729 | 431.8705 | 0.004615 | -0.34824 | 341 |  |  |
| -0.34622 | 432.9386 | 0.00473 | -0.34717 | 342 |  |  |
| -0.34515 | 434.0067 | 0.004833 | -0.34619 | 343 |  |  |
| -0.34409 | 435.0748 | 0.004927 | -0.345 | 344 |  |  |
| -0.34302 | 436.1429 | 0.005015 | -0.34406 | 345 |  |  |
| -0.34195 | 437.211 | 0.005096 | -0.34299 | 346 |  |  |
| -0.34088 | 438.2792 | 0.005172 | -0.34192 | 347 |  |  |
| -0.33981 | 439.3473 | 0.005245 | -0.34085 | 348 |  |  |
| -0.33875 | 440.4154 | 0.005314 | -0.33978 | 349 |  |  |
| -0.33768 | 441.4835 | 0.005381 | -0.33871 | 350 |  |  |
| -0.33661 | 442.5516 | 0.005444 | -0.33765 | 351 |  |  |
| -0.33554 | 443.6197 | 0.005505 | -0.33664 | 352 |  |  |
| -0.33447 | 444.6878 | 0.005566 | -0.33563 | 353 |  |  |
| -0.3334 | 445.756 | 0.005625 | -0.33453 | 354 |  |  |
| -0.33234 | 446.8241 | 0.005681 | -0.33344 | 355 |  |  |
| -0.33127 | 447.8922 | 0.005738 | -0.33243 | 356 |  |  |
| -0.3302 | 448.9603 | 0.005793 | -0.33148 | 357 |  |  |
| -0.32913 | 450.0284 | 0.005847 | -0.33032 | 358 |  |  |
| -0.32806 | 451.0965 | 0.005901 | -0.32922 | 359 |  |  |
| -0.327 | 452.1647 | 0.005954 | -0.32828 | 360 |  |  |
| -0.32593 | 453.2328 | 0.006006 | -0.32712 | 361 |  |  |
| -0.32486 | 454.3009 | 0.006058 | -0.32614 | 362 |  |  |
| -0.32379 | 455.369 | 0.006111 | -0.3251 | 363 |  |  |
| -0.32272 | 456.4371 | 0.006162 | -0.32401 | 364 |  |  |
| -0.32166 | 457.5052 | 0.006213 | -0.32306 | 365 |  |  |
| -0.32059 | 458.5733 | 0.006264 | -0.32196 | 366 |  |  |
| -0.31952 | 459.6415 | 0.006315 | -0.32083 | 367 |  |  |
| -0.31845 | 460.7096 | 0.006365 | -0.31967 | 368 |  |  |
| -0.31738 | 461.7777 | 0.006415 | -0.3187 | 369 |  |  |
| -0.31631 | 462.8458 | 0.006465 | -0.31769 | 370 |  |  |
| -0.31525 | 463.9139 | 0.006514 | -0.31659 | 371 |  |  |
| -0.31418 | 464.982 | 0.006563 | -0.31555 | 372 |  |  |
| -0.31311 | 466.0501 | 0.006611 | -0.31448 | 373 |  |  |
| -0.31204 | 467.1183 | 0.006659 | -0.31348 | 374 |  |  |
| -0.31097 | 468.1864 | 0.006705 | -0.31235 | 375 |  |  |
| -0.30991 | 469.2545 | 0.006751 | -0.31131 | 376 |  |  |
| -0.30884 | 470.3226 | 0.006795 | -0.31021 | 377 |  |  |
| -0.30777 | 471.3907 | 0.006839 | -0.30905 | 378 |  |  |
| -0.3067 | 472.4588 | 0.006881 | -0.30807 | 379 |  |  |
| -0.30563 | 473.527 | 0.006923 | -0.30704 | 380 |  |  |
| -0.30457 | 474.5951 | 0.006963 | -0.30597 | 381 |  |  |
| -0.3035 | 475.6632 | 0.007001 | -0.30508 | 382 |  |  |
| -0.30243 | 476.7313 | 0.007039 | -0.30383 | 383 |  |  |
| -0.30136 | 477.7994 | 0.007076 | -0.30283 | 384 |  |  |
| -0.30029 | 478.8675 | 0.00711 | -0.3017 | 385 |  |  |
| -0.29922 | 479.9356 | 0.007144 | -0.30075 | 386 |  |  |
| -0.29816 | 481.0038 | 0.007177 | -0.29962 | 387 |  |  |
| -0.29709 | 482.0719 | 0.007208 | -0.29865 | 388 |  |  |
| -0.29602 | 483.14 | 0.007238 | -0.29761 | 389 |  |  |
| -0.29495 | 484.2081 | 0.007267 | -0.29651 | 390 |  |  |
| -0.29388 | 485.2762 | 0.007296 | -0.29535 | 391 |  |  |
| -0.29282 | 486.3443 | 0.007323 | -0.29443 | 392 |  |  |
| -0.29175 | 487.4124 | 0.00735 | -0.29324 | 393 |  |  |
| -0.29068 | 488.4806 | 0.007375 | -0.29227 | 394 |  |  |
| -0.28961 | 489.5487 | 0.0074 | -0.29114 | 395 |  |  |
| -0.28854 | 490.6168 | 0.007424 | -0.29013 | 396 |  |  |
| -0.28748 | 491.6849 | 0.007448 | -0.28909 | 397 |  |  |
| -0.28641 | 492.753 | 0.007471 | -0.28796 | 398 |  |  |
| -0.28534 | 493.8211 | 0.007493 | -0.28687 | 399 |  |  |
| -0.28427 | 494.8893 | 0.007515 | -0.28589 | 400 |  |  |
| -0.2832 | 495.9574 | 0.007537 | -0.28476 | 401 |  |  |
| -0.28214 | 497.0255 | 0.007559 | -0.28375 | 402 |  |  |
| -0.28107 | 498.0936 | 0.00758 | -0.28268 | 403 |  |  |
| -0.28 | 499.1617 | 0.0076 | -0.28171 | 404 |  |  |
| -0.27893 | 500.2298 | 0.007619 | -0.28055 | 405 |  |  |
| -0.27786 | 501.2979 | 0.007639 | -0.27954 | 406 |  |  |
| -0.27679 | 502.3661 | 0.007658 | -0.2785 | 407 |  |  |
| -0.27573 | 503.4342 | 0.007678 | -0.2774 | 408 |  |  |
| -0.27466 | 504.5023 | 0.007697 | -0.27628 | 409 |  |  |
| -0.27359 | 505.5704 | 0.007716 | -0.27527 | 410 |  |  |
| -0.27252 | 506.6385 | 0.007734 | -0.27417 | 411 |  |  |
| -0.27145 | 507.7066 | 0.007753 | -0.27301 | 412 |  |  |
| -0.27039 | 508.7747 | 0.007771 | -0.27209 | 413 |  |  |
| -0.26932 | 509.8429 | 0.007788 | -0.27097 | 414 |  |  |
| -0.26825 | 510.911 | 0.007806 | -0.26993 | 415 |  |  |
| -0.26718 | 511.9791 | 0.007823 | -0.2688 | 416 |  |  |
| -0.26611 | 513.0472 | 0.00784 | -0.26779 | 417 |  |  |
| -0.26505 | 514.1153 | 0.007857 | -0.26675 | 418 |  |  |
| -0.26398 | 515.1834 | 0.007874 | -0.26566 | 419 |  |  |
| -0.26291 | 516.2516 | 0.007891 | -0.26453 | 420 |  |  |
| -0.26184 | 517.3197 | 0.007907 | -0.26349 | 421 |  |  |
| -0.26077 | 518.3878 | 0.007924 | -0.26236 | 422 |  |  |
| -0.2597 | 519.4559 | 0.00794 | -0.26147 | 423 |  |  |
| -0.25864 | 520.524 | 0.007956 | -0.26038 | 424 |  |  |
| -0.25757 | 521.5921 | 0.007972 | -0.25931 | 425 |  |  |
| -0.2565 | 522.6602 | 0.007988 | -0.25821 | 426 |  |  |
| -0.25543 | 523.7284 | 0.008003 | -0.25717 | 427 |  |  |
| -0.25436 | 524.7965 | 0.008018 | -0.25604 | 428 |  |  |
| -0.2533 | 525.8646 | 0.008033 | -0.25504 | 429 |  |  |
| -0.25223 | 526.9327 | 0.008048 | -0.25394 | 430 |  |  |
| -0.25116 | 528.0008 | 0.008063 | -0.2529 | 431 |  |  |
| -0.25009 | 529.0689 | 0.008077 | -0.25186 | 432 |  |  |
| -0.24902 | 530.137 | 0.008092 | -0.25076 | 433 |  |  |
| -0.24796 | 531.2052 | 0.008106 | -0.24966 | 434 |  |  |
| -0.24689 | 532.2733 | 0.00812 | -0.24866 | 435 |  |  |
| -0.24582 | 533.3414 | 0.008134 | -0.24759 | 436 |  |  |
| -0.24475 | 534.4095 | 0.008148 | -0.24649 | 437 |  |  |
| -0.24368 | 535.4776 | 0.008161 | -0.24542 | 438 |  |  |
| -0.24261 | 536.5457 | 0.008174 | -0.24442 | 439 |  |  |
| -0.24155 | 537.6139 | 0.008188 | -0.24329 | 440 |  |  |
| -0.24048 | 538.682 | 0.008201 | -0.24228 | 441 |  |  |
| -0.23941 | 539.7501 | 0.008214 | -0.24127 | 442 |  |  |
| -0.23834 | 540.8182 | 0.008226 | -0.24017 | 443 |  |  |
| -0.23727 | 541.8863 | 0.008239 | -0.23907 | 444 |  |  |
| -0.23621 | 542.9544 | 0.008251 | -0.23807 | 445 |  |  |
| -0.23514 | 544.0225 | 0.008263 | -0.237 | 446 |  |  |
| -0.23407 | 545.0907 | 0.008276 | -0.2359 | 447 |  |  |
| -0.233 | 546.1588 | 0.008288 | -0.23483 | 448 |  |  |
| -0.23193 | 547.2269 | 0.0083 | -0.23376 | 449 |  |  |
| -0.23087 | 548.295 | 0.008312 | -0.23273 | 450 |  |  |
| -0.2298 | 549.3631 | 0.008323 | -0.23166 | 451 |  |  |
| -0.22873 | 550.4312 | 0.008334 | -0.23053 | 452 |  |  |
| -0.22766 | 551.4993 | 0.008344 | -0.22946 | 453 |  |  |
| -0.22659 | 552.5675 | 0.008354 | -0.22849 | 454 |  |  |
| -0.22552 | 553.6356 | 0.008366 | -0.22736 | 455 |  |  |
| -0.22446 | 554.7037 | 0.008376 | -0.22635 | 456 |  |  |
| -0.22339 | 555.7718 | 0.008387 | -0.22519 | 457 |  |  |
| -0.22232 | 556.8399 | 0.008398 | -0.22427 | 458 |  |  |
| -0.22125 | 557.908 | 0.008409 | -0.22308 | 459 |  |  |
| -0.22018 | 558.9762 | 0.008418 | -0.22202 | 460 |  |  |
| -0.21912 | 560.0443 | 0.008427 | -0.22095 | 461 |  |  |
| -0.21805 | 561.1124 | 0.008438 | -0.21994 | 462 |  |  |
| -0.21698 | 562.1805 | 0.008447 | -0.21887 | 463 |  |  |
| -0.21591 | 563.2486 | 0.008455 | -0.21774 | 464 |  |  |
| -0.21484 | 564.3167 | 0.008463 | -0.21674 | 465 |  |  |
| -0.21378 | 565.3848 | 0.008473 | -0.2157 | 466 |  |  |
| -0.21271 | 566.453 | 0.008482 | -0.21457 | 467 |  |  |
| -0.21164 | 567.5211 | 0.008491 | -0.21353 | 468 |  |  |
| -0.21057 | 568.5892 | 0.0085 | -0.2124 | 469 |  |  |

B: impedance test

Sample: Blank (no inhibitor), Immersion time: 15 min

| Index |  | Frequency(Hz) | Z' (Ω) | -Z'' (Ω) | Z (Ω) | -Phase(°) | Time (s) |
| --- | --- | --- | --- | --- | --- | --- | --- |
| 1 |  | 100000 | 0.999053 | 2.193867 | 2.410634 | 65.51622 | 9.219053 |
| 2 |  | 79433 | 0.789243 | 1.518874 | 1.711691 | 62.54254 | 10.38907 |
| 3 |  | 63096 | 0.599918 | 1.081345 | 1.236611 | 60.97899 | 11.78409 |
| 4 |  | 50119 | 0.462425 | 0.799836 | 0.92389 | 59.96566 | 13.14412 |
| 5 |  | 39811 | 0.368125 | 0.608081 | 0.710829 | 58.8098 | 14.40415 |
| 6 |  | 31623 | 0.30646 | 0.472723 | 0.563369 | 57.04519 | 15.70917 |
| 7 |  | 25119 | 0.267055 | 0.374283 | 0.45979 | 54.49171 | 16.98419 |
| 8 |  | 19953 | 0.242691 | 0.301464 | 0.387014 | 51.16446 | 18.23921 |
| 9 |  | 15849 | 0.228336 | 0.246786 | 0.336216 | 47.2238 | 19.53924 |
| 10 |  | 12589 | 0.224086 | 0.207369 | 0.305313 | 42.78109 | 20.80927 |
| 11 |  | 10000 | 0.215814 | 0.177515 | 0.279441 | 39.43855 | 22.9793 |
| 12 |  | 7943.3 | 0.25672 | 0.175666 | 0.311069 | 34.38271 | 24.14932 |
| 13 |  | 6309.6 | 0.254278 | 0.163438 | 0.302273 | 32.73103 | 25.45435 |
| 14 |  | 5011.9 | 0.26429 | 0.163833 | 0.310951 | 31.79467 | 26.70937 |
| 15 |  | 3981.1 | 0.263004 | 0.166668 | 0.311367 | 32.3628 | 27.9444 |
| 16 |  | 3162.3 | 0.265417 | 0.178128 | 0.319649 | 33.86656 | 29.22442 |
| 17 |  | 2511.9 | 0.255951 | 0.189137 | 0.318251 | 36.46284 | 30.59445 |
| 18 |  | 1995.3 | 0.263349 | 0.217213 | 0.341371 | 39.51612 | 31.82947 |
| 19 |  | 1584.9 | 0.258262 | 0.243631 | 0.355042 | 43.33016 | 33.03449 |
| 20 |  | 1258.9 | 0.256319 | 0.278733 | 0.37867 | 47.39877 | 34.23951 |
| 21 |  | 1000 | 0.246397 | 0.31127 | 0.396989 | 51.63539 | 35.50454 |
| 22 |  | 794.33 | 0.257955 | 0.3767 | 0.456556 | 55.59754 | 36.87956 |
| 23 |  | 630.96 | 0.273613 | 0.45971 | 0.534974 | 59.23945 | 38.13958 |
| 24 |  | 501.19 | 0.290886 | 0.559231 | 0.63036 | 62.51866 | 39.43961 |
| 25 |  | 398.11 | 0.313586 | 0.684359 | 0.752784 | 65.38185 | 40.67963 |
| 26 |  | 316.23 | 0.342871 | 0.839274 | 0.90661 | 67.77839 | 41.94465 |
| 27 |  | 251.19 | 0.380376 | 1.029713 | 1.097723 | 69.72578 | 43.24968 |
| 28 |  | 199.53 | 0.428214 | 1.263312 | 1.333913 | 71.27534 | 44.5197 |
| 29 |  | 158.49 | 0.490086 | 1.549231 | 1.6249 | 72.44569 | 45.81473 |
| 30 |  | 125.89 | 0.570164 | 1.899018 | 1.982764 | 73.28807 | 47.17975 |
| 31 |  | 100 | 0.674148 | 2.324668 | 2.420446 | 73.82797 | 48.52478 |
| 32 |  | 79.433 | 0.813049 | 2.857107 | 2.97054 | 74.11519 | 49.8998 |
| 33 |  | 63.096 | 0.992354 | 3.490472 | 3.628796 | 74.12936 | 51.30483 |
| 34 |  | 50.119 | 1.22983 | 4.261292 | 4.43521 | 73.9016 | 52.73986 |
| 35 |  | 39.811 | 1.546252 | 5.191763 | 5.41713 | 73.415 | 61.81002 |
| 36 |  | 31.623 | 1.973207 | 6.309357 | 6.610714 | 72.63329 | 63.38505 |
| 37 |  | 25.119 | 2.551526 | 7.646931 | 8.06138 | 71.54787 | 64.93008 |
| 38 |  | 19.953 | 3.338591 | 9.216138 | 9.802213 | 70.08683 | 66.78012 |
| 39 |  | 15.849 | 4.413731 | 11.04682 | 11.89594 | 68.22094 | 68.68515 |
| 40 |  | 12.589 | 5.870861 | 13.1216 | 14.37509 | 65.89532 | 70.59519 |
| 41 |  | 10 | 7.820736 | 15.38915 | 17.26239 | 63.06042 | 72.83023 |
| 42 |  | 7.9433 | 10.36182 | 17.75927 | 20.5611 | 59.73815 | 75.09527 |
| 43 |  | 6.3096 | 13.59845 | 20.05712 | 24.23233 | 55.86328 | 77.38531 |
| 44 |  | 5.0119 | 17.54284 | 22.08001 | 28.20068 | 51.53242 | 79.77036 |
| 45 |  | 3.9811 | 22.1224 | 23.55751 | 32.31651 | 46.79944 | 82.1354 |
| 46 |  | 3.1623 | 27.09641 | 24.23703 | 36.35449 | 41.8118 | 84.62045 |
| 47 |  | 2.5119 | 32.18493 | 24.03266 | 40.16763 | 36.7489 | 87.11049 |
| 48 |  | 1.9953 | 37.0511 | 22.97753 | 43.5976 | 31.80544 | 89.80054 |
| 49 |  | 1.5849 | 41.38489 | 21.23055 | 46.51285 | 27.15793 | 92.45059 |
| 50 |  | 1.2589 | 45.00559 | 19.00237 | 48.85277 | 22.89056 | 95.32564 |
| 51 |  | 1 | 47.93975 | 16.61747 | 50.73815 | 19.11798 | 98.5907 |
| 52 |  | 0.79433 | 50.16409 | 14.21542 | 52.13937 | 15.82159 | 101.7658 |
| 53 |  | 0.63096 | 51.82565 | 11.95903 | 53.18756 | 12.99384 | 105.2558 |
| 54 |  | 0.50119 | 53.06191 | 9.96416 | 53.98936 | 10.63536 | 109.0059 |
| 55 |  | 0.39811 | 53.95007 | 8.195152 | 54.56895 | 8.637344 | 113.181 |
| 56 |  | 0.31623 | 54.57799 | 6.679746 | 54.98524 | 6.977671 | 117.9061 |
| 57 |  | 0.25119 | 54.97913 | 5.471739 | 55.25074 | 5.683585 | 123.2212 |
| 58 |  | 0.19953 | 55.36042 | 4.348653 | 55.53095 | 4.491456 | 129.3613 |
| 59 |  | 0.15849 | 55.4885 | 3.51146 | 55.59949 | 3.621001 | 136.7114 |
| 60 |  | 0.12589 | 55.61814 | 2.688995 | 55.68311 | 2.767949 | 145.1966 |
| 61 |  | 0.1 | 55.75588 | 2.03313 | 55.79294 | 2.088357 | 155.9268 |
| 62 |  | 0.079433 | 55.76636 | 1.545844 | 55.78778 | 1.587833 | 168.132 |
| 63 |  | 0.063096 | 55.6869 | 1.137879 | 55.69852 | 1.170591 | 182.9373 |
| 64 |  | 0.050119 | 55.48249 | 0.741468 | 55.48745 | 0.765655 | 201.0126 |
| 65 |  | 0.039811 | 55.26196 | 0.303403 | 55.26279 | 0.314566 | 223.278 |
| 66 |  | 0.031623 | 55.02766 | -0.16423 | 55.02791 | -0.171 | 250.6785 |
| 67 |  | 0.025119 | 54.72971 | -0.60854 | 54.73309 | -0.63705 | 284.5741 |
| 68 |  | 0.019953 | 54.30879 | -1.03697 | 54.31869 | -1.09387 | 326.6849 |
| 69 |  | 0.015849 | 53.85104 | -1.45687 | 53.87074 | -1.54968 | 379.0659 |
| 70 |  | 0.012589 | 53.24921 | -1.86012 | 53.28169 | -2.00067 | 444.4721 |
| 71 |  | 0.01 | 52.42789 | -2.27333 | 52.47715 | -2.48285 | 526.2336 |

Sample: Blank (no inhibitor), Immersion time: 2h

| Index | Frequency(Hz) | Z' (Ω) | -Z'' (Ω) | Z (Ω) | -Phase(°) | Time (s) |
| --- | --- | --- | --- | --- | --- | --- |
| 1 | 100000 | 0.974061 | 2.257599 | 2.45877 | 66.66179 | 8.890054 |
| 2 | 79433 | 0.773766 | 1.567687 | 1.748244 | 63.73035 | 10.01507 |
| 3 | 63096 | 0.592787 | 1.122092 | 1.269049 | 62.15309 | 11.3701 |
| 4 | 50119 | 0.459275 | 0.833114 | 0.951321 | 61.13316 | 12.73512 |
| 5 | 39811 | 0.368326 | 0.63587 | 0.734844 | 59.91856 | 14.09515 |
| 6 | 31623 | 0.307995 | 0.495033 | 0.583026 | 58.11133 | 15.36517 |
| 7 | 25119 | 0.269603 | 0.392341 | 0.476043 | 55.50449 | 16.62519 |
| 8 | 19953 | 0.245705 | 0.316271 | 0.400497 | 52.15702 | 17.89022 |
| 9 | 15849 | 0.231509 | 0.259328 | 0.347631 | 48.24389 | 19.15024 |
| 10 | 12589 | 0.225324 | 0.217354 | 0.313072 | 43.96856 | 20.41527 |
| 11 | 10000 | 0.21871 | 0.186824 | 0.287641 | 40.50418 | 22.58031 |
| 12 | 7943.3 | 0.249768 | 0.181339 | 0.308654 | 35.98073 | 23.75533 |
| 13 | 6309.6 | 0.253093 | 0.17096 | 0.305424 | 34.03834 | 25.01535 |
| 14 | 5011.9 | 0.260395 | 0.170225 | 0.311098 | 33.17331 | 26.28037 |
| 15 | 3981.1 | 0.261363 | 0.174223 | 0.314109 | 33.68727 | 27.53039 |
| 16 | 3162.3 | 0.262205 | 0.185456 | 0.321162 | 35.27155 | 28.76542 |
| 17 | 2511.9 | 0.254675 | 0.198453 | 0.322867 | 37.92712 | 30.03544 |
| 18 | 1995.3 | 0.259451 | 0.226784 | 0.344595 | 41.15655 | 31.29047 |
| 19 | 1584.9 | 0.255775 | 0.256489 | 0.362226 | 45.07997 | 32.50549 |
| 20 | 1258.9 | 0.253886 | 0.294827 | 0.389077 | 49.26715 | 33.71551 |
| 21 | 1000 | 0.249215 | 0.337548 | 0.41958 | 53.56122 | 34.98054 |
| 22 | 794.33 | 0.26075 | 0.410031 | 0.485918 | 57.5466 | 36.33556 |
| 23 | 630.96 | 0.276216 | 0.501366 | 0.572419 | 61.14843 | 37.60058 |
| 24 | 501.19 | 0.293764 | 0.612225 | 0.679056 | 64.36689 | 38.86061 |
| 25 | 398.11 | 0.317515 | 0.752202 | 0.816471 | 67.11472 | 40.10563 |
| 26 | 316.23 | 0.348341 | 0.924936 | 0.988356 | 69.36309 | 41.37065 |
| 27 | 251.19 | 0.3891 | 1.138667 | 1.203312 | 71.13389 | 42.65568 |
| 28 | 199.53 | 0.442511 | 1.400889 | 1.469118 | 72.46979 | 43.9207 |
| 29 | 158.49 | 0.512975 | 1.721435 | 1.796241 | 73.40632 | 45.21572 |
| 30 | 125.89 | 0.607359 | 2.115267 | 2.200736 | 73.97959 | 46.56075 |
| 31 | 100 | 0.736568 | 2.60619 | 2.708276 | 74.21851 | 47.86077 |
| 32 | 79.433 | 0.908116 | 3.19147 | 3.318155 | 74.11658 | 49.2108 |
| 33 | 63.096 | 1.140168 | 3.898599 | 4.061903 | 73.69814 | 50.62082 |
| 34 | 50.119 | 1.455793 | 4.748219 | 4.966378 | 72.95458 | 51.92585 |
| 35 | 39.811 | 1.887269 | 5.762355 | 6.06354 | 71.8655 | 60.99602 |
| 36 | 31.623 | 2.480057 | 6.958985 | 7.387703 | 70.38484 | 62.55604 |
| 37 | 25.119 | 3.288392 | 8.35297 | 8.97695 | 68.51146 | 64.06607 |
| 38 | 19.953 | 4.390389 | 9.928576 | 10.85597 | 66.14512 | 65.88611 |
| 39 | 15.849 | 5.867897 | 11.65569 | 13.04942 | 63.27766 | 67.76614 |
| 40 | 12.589 | 7.807369 | 13.45438 | 15.55556 | 59.87407 | 69.66618 |
| 41 | 10 | 10.27483 | 15.19118 | 18.33969 | 55.92683 | 71.90622 |
| 42 | 7.9433 | 13.28539 | 16.70579 | 21.34443 | 51.5063 | 74.17626 |
| 43 | 6.3096 | 16.77715 | 17.78914 | 24.45253 | 46.67695 | 76.4463 |
| 44 | 5.0119 | 20.56412 | 18.25726 | 27.49928 | 41.59934 | 78.83635 |
[truncated: 332,863 more chars]
